# Co-Consumption of Methanol and Succinate by *Methylobacterium extorquens* AM1

**DOI:** 10.1371/journal.pone.0048271

**Published:** 2012-11-01

**Authors:** Rémi Peyraud, Patrick Kiefer, Philipp Christen, Jean-Charles Portais, Julia A. Vorholt

**Affiliations:** 1 Institute of Microbiology, ETH Zurich, Zurich, Switzerland; 2 Université de Toulouse, INSA, UPS, INP, Toulouse, France; 3 INRA, UMR792 Ingénierie des Systèmes Biologiques et des Procédés, Toulouse, France; 4 CNRS, UMR5504, Toulouse, France; University of Nottingham, United Kingdom

## Abstract

*Methylobacterium extorquens* AM1 is a facultative methylotrophic Alphaproteobacterium and has been subject to intense study under pure methylotrophic as well as pure heterotrophic growth conditions in the past. Here, we investigated the metabolism of *M. extorquens* AM1 under mixed substrate conditions, i.e., in the presence of methanol plus succinate. We found that both substrates were co-consumed, and the carbon conversion was two-thirds from succinate and one-third from methanol relative to mol carbon. ^13^C-methanol labeling and liquid chromatography mass spectrometry analyses revealed the different fates of the carbon from the two substrates. Methanol was primarily oxidized to CO_2_ for energy generation. However, a portion of the methanol entered biosynthetic reactions via reactions specific to the one-carbon carrier tetrahydrofolate. In contrast, succinate was primarily used to provide precursor metabolites for bulk biomass production. This work opens new perspectives on the role of methylotrophy when substrates are simultaneously available, a situation prevailing under environmental conditions.

## Introduction

Bacteria often live in environments containing diverse substrates [1]. One of such habitat is the phyllosphere, where facultative methylotrophic bacteria are found to be ubiquitous and abundant [2], [3], [4]. These methylotrophic bacteria belong to the genus *Methylobacterium* and are known to metabolize methanol but also a limited number of alternative carbon substrates, such as organic acids and alcohols. Plant leaf surfaces release diverse carbon sources, mainly sugars and organic acids, at low amounts (µM range) [5], [6], [7], and these sources are heterogeneously located and result of leaching through the cuticle [8]. In addition to these substrates, volatile carbon substrates, particularly the plant cell wall metabolism byproduct methanol, are released via the stomata. Methanol emission peaks in the morning, when the stomata first open [9]. There is evidence that methanol is consumed by *Methylobacterium* and contributes to the epiphytic fitness of the organism [6], [10]. However, in addition to the peak of methanol emission in the morning, *Methylobacterium* should adapt its metabolism to use additional carbon sources during the rest of the day when methanol emission is low or during the night when stomata are closed and methanol is consequently no longer available [9]. Accordingly, additional carbon sources were suggested to be relevant for the efficient colonization of plant surfaces *in situ* [10].

*M. extorquens* AM1 is a model methylotrophic organism, and a number of novel enzymes and pathways involved in methanol dissimilation and assimilation were shown to operate in this organism [11], [12], [13], [14]. In the past, a number of studies reported metabolic differences between methylotrophic growth conditions (i.e., methanol as sole source of carbon and energy) and multicarbon growth conditions (i.e., succinate as sole source of carbon and energy). These investigations include transcriptomic [15], proteomic [16], [17], and metabolomic studies [18], [19]. The core of the central metabolism of *M. extorquens* AM1 was described to encompass 85 biochemical reactions that are strongly reprogrammed upon adaptation to nutrient changes [20]. Indeed, metabolic pathways such as the tetrahydromethanopterin-dependent oxidation pathway, the serine cycle, and the ethylmalonyl-CoA pathway are essential during growth on methanol but dispensable for growth on organic acids (although some individual enzymes may still be required). In contrast, a complete TCA cycle and pyruvate dehydrogenase, which provide energy during growth on organic acid, are not required during C1 growth [20], [21], [22].

All of these metabolic pathways are strongly connected via a dense network of reactions that interconvert key C2–C3–C4 metabolites (i.e., acetyl-CoA, phosphoenolpyruvate, pyruvate, oxaloacetate, and malate). Due to the occurrence of sequences of reactions that generate substrate cycles, this network of reactions is highly flexible and allows the efficient switching of the central metabolism towards the utilization of alternative substrates [20], [21], [22]. Recently, the adaptation involved in the transition from succinate to methanol utilization via a systems-level approach was investigated [23]. The study revealed that a significant amount of methanol is quickly oxidized to formate in the early stage of the transition but that the first steps of the assimilation processes are repressed. Thereafter, assimilation starts only when the entire set of required enzymes is expressed [23]. Such a transition between succinate and methanol [23] might mimic a diauxic shift, which is a well-described mechanism of catabolic repression during mixed substrate conditions. Indeed, diauxic growth is based on an important genetic regulation phenomenon that was uncovered decades ago as a strategy for bacteria to address the availability of two substrates [24], [25], [26]. In essence, one substrate is utilized exclusively, and it is only upon exhaustion of the “preferred” substrate, often the substrate supporting the higher growth rate, that the genes for enzymes required for the second substrate are induced. Several regulation mechanisms were described to trigger catabolic repression, like the phosphotransferase system (PTS), riboswitches, or regulators such as Crp/CyaA [26].

The metabolic profile of *M. extorquens* AM1 in the presence of a multicarbon compound(s) in addition to methanol has not been thoroughly investigated. Preliminary enzyme activity data in cell extracts indicated that methanol dehydrogenase and enzymes of the assimilatory serine cycle were detectable in *M. extorquens* AM1 cell extracts when cells were incubated overnight with methanol plus succinate [27]. An intermediate level of some of the enzymes activities compared to their levels under pure methanol or succinate conditions suggests the presence of a dedicated metabolism adapted to mixotrophic conditions. However, no information on cell growth or substrate utilization during the incubation with both substrates was included in this earlier study. From another perspective, the co-consumption of methanol and thiosulfate by several *Methylobacterium* species, including *M. extorquens* AM1, has been reported [28]. Thiosulfate was effectively used as an additional energy source and appeared to enhance growth capacity. These results indicate that *M. extorquens* AM1 is able to utilize at least two very different energy sources of inorganic and organic nature.

In this study, we investigated the metabolism of *M. extorquens* AM1 under a mixed carbon substrate condition, i.e., methanol plus succinate, to address whether diauxic growth or co-consumption occurs.

## Results

### Characterization of *M. extorquens* AM1 Growth on Methanol Plus Succinate

**Figure 1:**
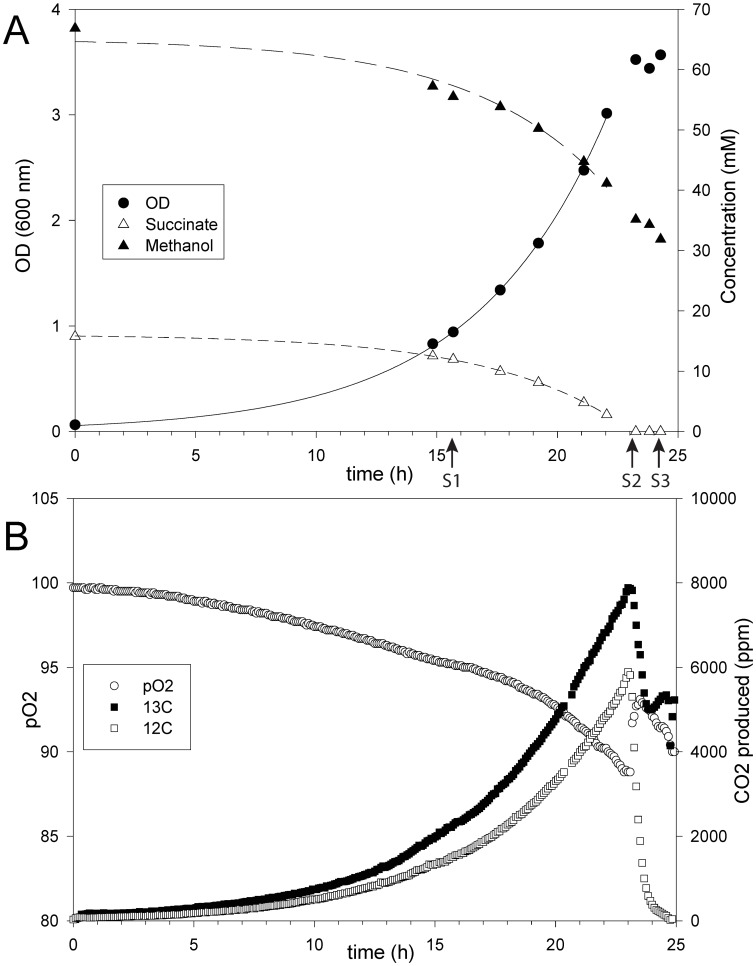
Monitoring of *M. extorquens* AM1 growth parameters in batch-culture with 60 mM methanol plus 15 mM succinate. A. Monitoring of Optical Density (OD) at 600 nm (black circle), methanol consumption (black triangle) and succinate (white triangle) consumption. B. Monitoring of oxygen partial pressure (pO_2_) (white circle), ^13^C (black square) and ^12^C (white square) CO_2_ production in exhaust gas. Metabolite sampling was performed at three timepoints as indicated in A: mid-co-consumption phase (Sampling time 1, S1), end co-consumption phase (Sampling time 2, S2), and transition phase (Sampling time 3, S3). The figure shows data of one replicate.

To infer the adaptation of *M. extorquens* AM1 to conditions under which both succinate and methanol are available, growth experiments were performed on minimal medium with equivalent C-mol of methanol and succinate, i.e., 60 mM and 15 mM, respectively. A growth rate of 0.18±0.01 h^−1^ was observed under this condition, which is roughly similar to that under either pure succinate growth (0.20±0.01 h^−1^) or pure methanol growth conditions (0.17±0.01 h^−1^) (Table 1). Both methanol and succinate were consumed during the exponential growth phase (Figure 1A) indicating the co-consumption of the two compounds. For each substrate, the consumption rate under mixed substrate conditions was lower than that observed under pure culture conditions. Succinate utilization dropped by 34%, and methanol utilization dropped by 70% (Table 1). Notably, the sum of the two consumption rates (in moles of carbon) was similar to the amount of substrate consumed under pure conditions, approximately 17 C-mmol·g^−1^·h^−1^ (Table 1). The relatively higher contribution of succinate to growth under mixed substrate conditions (approximately 72% C-mol consumed) shows that succinate was the predominant substrate when both substrates were simultaneously available. The biomass yield obtained was similar, i.e., approximately 10.6 g.C-mol^−1^, under the different conditions (Table 1). Growth ceased once succinate was fully consumed, and growth resumed after a transition phase of approximately 1.5 h.

**Table 1:** Growth parameters of *M. extorquens* AM1 cells growing in batch-culture in minimal medium with 120 mM methanol, or 15 mM succinate, or 60 mM methanol plus 15 mM succinate.

Growth parameters	succinate (15 mM)	succinate (15 mM) + methanol (60 mM)	methanol (120 mM)
Growth rate (h^−1^)	0,20±0.01	0.18±0.01	0.17±0.01
succinate uptake rate (C-mmol.g^−1^.h^−1^)	18.9±1.8	12.5±1.9	
methanol uptake rate (C-mmol.g^−1^.h^−1^)		4.8–4.9**	15.9±1.2
CO2 production rate (mmol.g^−1^.h^−1^)	8.3±1.3	7.7±2.1	6.7±1.8
Biomass Yield (g.g^−1^)	0.36±0.02	0.36–0.35**	0.33±0.03
(g/C-mol)	10.5±0.7	10.8–10.7**	10.7±1.1
**^13^C-Labelling**		**succinate(^12^C) + methanol(^13^C)**	
**^13^**C CO_2_ production rate (mmol.g^−1^.h^−1^)		5.2*	
**^12^**C CO_2_ production rate (mmol.g^−1^1.h^−1^)		3.6*	

Average and standard deviations (2σ) of 3 biological replicates, except: * one replicate (^13^C labeling experiment), ** two replicates.

To follow the metabolic fate of methanol and succinate during mixed substrate conditions, we performed an experiment with ^13^C-labeled methanol (>99% ^13^C) and succinate at a natural abundance of ^13^C (1.1%). The determination of ^13^C-CO_2_ production in the exhaust gas of the bioreactor revealed that the methanol consumed was almost entirely converted to CO_2_ (Figure 1B and Table 1), indicating that the methanol was used mainly for catabolic purposes. A portion of the released CO_2_ was produced from succinate; 41% of the CO_2_ was ^12^C labeled and represents 29% of the carbon of the consumed succinate. The proportions of both ^13^C- and ^12^C-CO_2_ produced were stable over time until all succinate was depleted. The ^12^C-CO_2_ production was then abolished, but the dissimilation of methanol remained; consequently, the ^13^C-CO_2_ production became exclusive.

### Incorporation of ^13^C Methanol into Amino Acids and Selected Metabolites during Mixed Substrate Conditions Revealed by LC-MS

**Figure 2:**
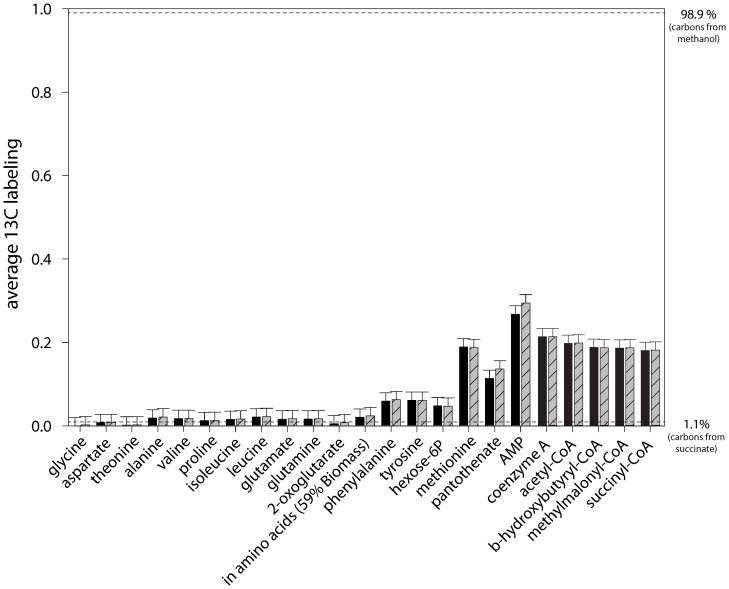
Average ^13^C labeling in intra-cellular metabolites measured by LC-MS during the growth of *M. extorquens* AM1 upon co-consumption with ^13^C (>99%) methanol and natural abundance (1.1% ^13^C) succinate. Metabolite quenching, extraction and measurements were performed specifically for each class of metabolite, i.e., amino acids, polar compounds, and coenzyme A thioesters, as described in the materials and methods. Average ^13^C labeling. (black): Sample collected during mid-co-consumption phase (Sampling time 1 in Figure 1), (gray hatched): sample collected at the end of the co-consumption phase (Sampling time 2 in Figure 1).

**Figure 3:**
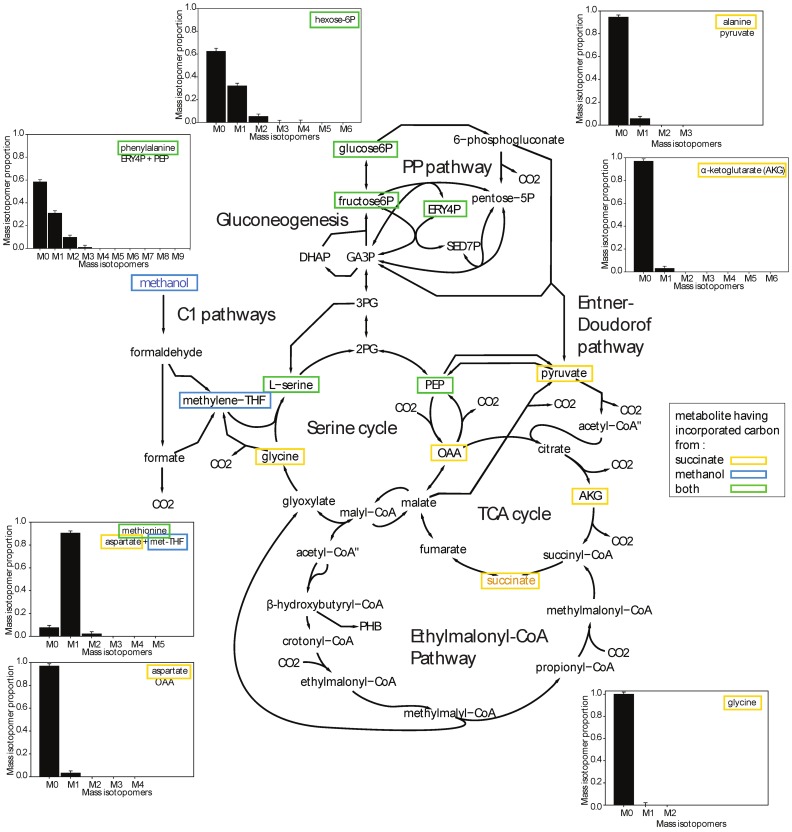
Central metabolic network map displaying selected mass isotopomer distributions of the central metabolites measured by LC-MS of *M. extorquens* AM1 growth upon co-consumption with ^13^C (>99%) methanol and natural abundance (1.1% ^13^C) succinate. The precursors of the amino acids measured in the central metabolism and directly measured metabolites are indicated in boxes. The box colors indicate substrate-specific carbon incorporation: orange from succinate, blue from methanol, green from both. Mass isotopomer data correspond to samples collected during the mid-co-consumption phase (Sampling time 1 in Figure 1).

**Figure 4:**
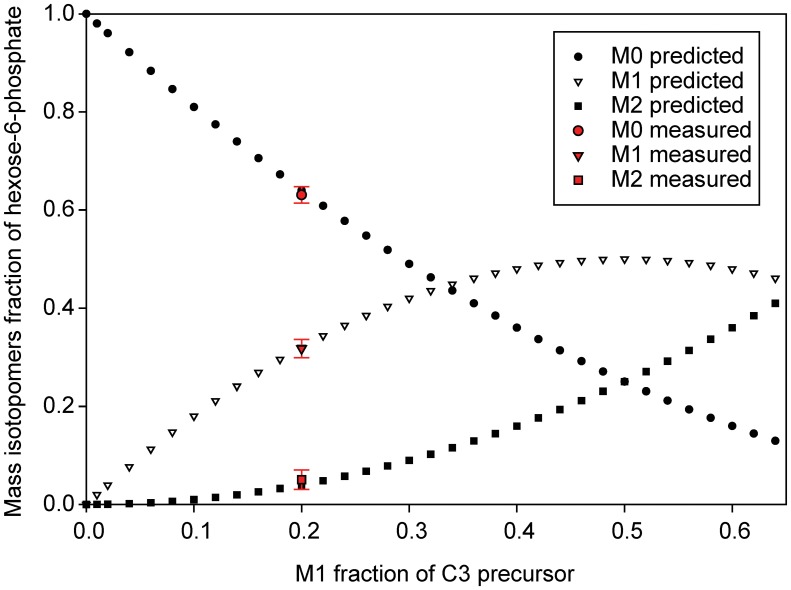
Prediction of the mass isotopomer distribution of hexose-phosphate depending of the M1 mass isotopomers fraction into C3 precursors of gluconeogenesis. Black, predicted values; red, measured values. The mass isotopomer fraction of hexose-phosphate measurements were 0.63±0.017 for M0, 0.32±0.019 M1, 0.05±0.020 M2, and 0.002±0.020 M3. These values correspond to the probabilistic recombination of two C3-units from gluconeogenesis assuming that 20% of the C3 units have incorporated one ^13^C carbon and 80% of the units are not labeled.

As outlined above, the majority of methanol was found to be catabolized to CO_2_. To confirm that little methanol was assimilated into biomass and to elucidate the metabolic fate of the one-carbon substrate, we analyzed the labeling pattern of intracellular metabolites by LC-MS. Sampling for metabolites was performed at three different timepoints during growth with ^13^C methanol and naturally labeled succinate. The first sample was collected in the middle of the first exponential growth phase, and the second sample was harvested just at the complete exhaustion of succinate in the medium. A third sample was harvested to monitor the extent of methanol assimilation 90 min after succinate depletion. The average ^13^C labeling (AL_13C_), which corresponds to the percentage of ^13^C carbon incorporated into the metabolites, was calculated for each compound (see Materials and Methods). This value reflects the mean proportion of methanol-derived carbon in the molecule: an AL_13C_ of 1.1% means that all carbon atoms originate from succinate and an AL_13C_ of 99% means that all carbon atoms originate from methanol. The AL_13C_ values at the first two timepoints were identical, indicating that the metabolism was stable until the depletion of succinate (Figure 2). The AL_13C_ values ranged from 1% to 27% depending on the metabolite, indicating that the dominant carbon source for biosynthesis was indeed succinate, consistent with the measured consumption and production rates (Table 1). However, remarkable differences in the ^13^C fraction of the metabolites were observed. Whereas the AL_13C_ of most amino acids was approximately 2.0%, hexose-phosphate (5%), phenylalanine (6%), tyrosine (6%), and especially methionine (19%) showed higher AL_13C_ values (Figure 2). An analysis of the mass isotopomer distribution of methionine revealed that 90% of the methionine contained one labeled carbon (Figure 3 and Figure S1). Methionine is generated from aspartate plus 5-methyltetrahydrofolate (M-THF). Because no label incorporation was observed in aspartate (1% ±2), the ^13^C label found in methionine indicates that up to 90% ±2 of its precursor, M-THF, was produced from assimilated methanol. As mentioned above, phenylalanine, tyrosine and hexose-phosphate also showed a small but significant incorporation of the label from methanol, which resulted in an increased abundance of the mass isotopomers M1 and M2. These compounds are synthesized by gluconeogenesis. Four metabolites generated from succinate and methanol represent potential precursors for gluconeogenesis: oxaloacetate, pyruvate, glycine, and 5,10-methylenetetrahydrofolate (Me-THF). Because no significant labeling was found in alanine (a derivative of pyruvate), aspartate (a derivative of oxaloacetate) or glycine, Me-THF (which condenses with glycine to form serine) can explain the introduction of the label into the gluconeogenesis pathway. Indeed, monolabeled phosphoglycerate was generated, which can lead to the incorporation of the label into phosphoenolpyruvate, the precursor of phenylalanine and tyrosine, and/or via gluconeogenesis into hexose-phosphate. An analysis of the hexose-phosphate mass isotopomer fractions indicated that 20% of monolabeled 2-phosphoglycerate is generated from the serine cycle, i.e., glycine plus Me-THF condensation (Figure 4). This observation indicates that serine is produced at least partially (>20%) from the condensation of glycine plus C1 compounds under mixed substrate conditions.

Two additional metabolites involving the incorporation of C1 precursors showed elevated AL_13C_ values: pantothenate (11±2%) and adenosine monophosphate (AMP) (27±2%). Pantothenic acid is generated from one Me-THF and pyruvate, and in the case of AMP biosynthesis, two formyl-THF molecules and one CO_2_ are incorporated into the purine part of the molecule. To investigate whether the observed AL_13C_ can be explained by the incorporation of C1 compounds originating from methanol, AL_13C_ values were calculated assuming that: i. tetrahydrofolate activated C1 precursors were mainly derived from methanol (90% ^13^C according to the labeling state of methionine, see above); ii. CO_2_ contains 59% ^13^C (Table 1); and iii. all other carbon atoms originate from naturally labeled succinate (1.1% ^13^C). The calculated AL_13C_ values were 19% (methionine), 11% (pantothenic acid) and 25% (AMP), and in agreement with the experimentally determined values. Thus, these results indicate that the incorporation of ^13^C is limited to metabolites that require C1 precursors for formation.

To validate the approach taken and to demonstrate the enhanced assimilation of methanol into intracellular metabolites after succinate depletion, additional samples were taken at 90 minutes after succinate was consumed. Indeed, ^13^C incorporation increased in all amino acids (Figures S2, S3), indicating that cells began to use methanol as a carbon sources in all biosynthesis processes after succinate depletion.

### Incorporation of ^13^C Methanol into CoA Esters and their Precursors during Mixed Substrate Conditions

The ethylmalonyl-CoA pathway is critical for providing anaplerotic support to the serine cycle during growth on methanol by glyoxylate regeneration [12], [29]. To monitor the operation of the ethylmalonyl-CoA pathway during growth on methanol plus succinate, the CoA-esters were extracted and analyzed by LC-MS at different timepoints. Only four of the 12 CoA esters involved in the ethylmalonyl-CoA pathway, as well as free coenzyme A (CoA), could be detected. The key intermediates crotonyl-CoA and ethylmalonyl-CoA were not detectable. Free CoA and all detected CoA esters showed significant incorporation of the label (Figure 2). The highest AL_13C_ was found for free Coenzyme A (22%), followed by Acetyl-CoA (20%). All CoA esters of C2 and C4 organic acids had AL_13C_ values of approximately 19%. In addition, all CoA esters showed very similar mass isotopomer distributions (Figure S4). The decrease of the average labeling with increasing number of carbon atoms in the organic acid moiety indicates that labeled carbon atoms are located in the CoA part rather than in the esterified acids. C4 β-hydroxybutyryl-CoA and C4 methylmalonyl-CoA showed very similar AL_13C_, and no significant change in the MID of methylmalonyl-CoA due to the incorporation of ^13^C-labeled CO_2_ via the ethylmalonyl-CoA pathway (note that the CO_2_ in the reactor is 59% labeled) was observed. Thus, it can be deduced that the ethylmalonyl-CoA pathway was not operating during the co-consumption of succinate and methanol, which is consistent with the failure to detect key intermediates of the pathway that are detectable during methylotrophic growth [12].

The incorporation of ^13^C label from ^13^C methanol seems to be restricted to metabolites for which biosynthesis requires C1 precursors. We used simulations to determine whether the observed ^13^C labeling into the CoA moiety also originated from the C1 precursors and was thus consistent with the CoA biosynthetic pathway (Figure S5). The carbon atoms of CoA originate from AMP, pantothenic acid, and carbon 2 and 3 of cysteine, which are derived from carbon 2 and 3 of serine. As mentioned above, two metabolic origins of the serine carbons are possible, the condensation of glycine plus a C1 precursor (in position 3 of serine) via the serine cycle or a C3 precursor from gluconeogenesis. The AL_13C_ values calculated based on the measured AL_13C_ of AMP and pantothenic acid were 18.6% if 20% of serine had incorporated ^13^C carbon into position 3 and 21.9% if 90% of serine had incorporated one-^13^C carbons (the labeling state of the C1 precursor). The measured AL_13C_ of coenzyme A was 21.6%, indicating that almost all serine molecules might have contained ^13^C carbon from C1 units. These results support the theory that serine is predominantly produced from glycine and Me-THF during growth in the presence of methanol plus succinate and that one-carbon-units for biosynthetic purposes are produced from methanol.

### Energetic Contributions of Methanol and Succinate into Metabolism

**Figure 5:**
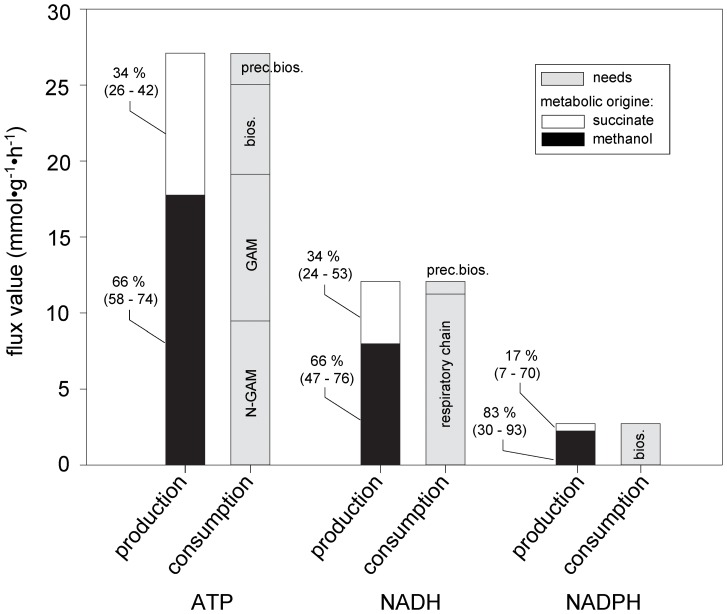
Metabolic contribution of succinate and methanol in energetics (ATP, NADH, NADPH) during co-consumption calculated by metabolic flux analysis through the genome-scale metabolic network of *M. extorquens* AM1 [**20**]. Methanol and succinate contributions are indicated in black and white, respectively, for a feasible solution under the constraints of the measured experimental values (methanol uptake rate, succinate uptake rate, growth rate, CO_2_ production rate). The % of their contribution to all needs is indicated at the left part of the bars; the values under the brackets are the lower and upper limits of their contributions calculated by the Flux Variability Analysis. N-GAM: non-growth-associated maintenance; GAM: growth-associated maintenance; Bios.: biomass biosynthesis; Prec. Bios.: precursor biosynthesis through the central metabolic network.

The above data showed that methanol and succinate were co-consumed but that their carbon atoms had distinct metabolic fates. To better understand the cell physiology and especially the energetic contributions of both substrates during co-consumption, we performed a flux variability analysis using the genome-scale network available for *M. extorquens* AM1 [20] to determine the flux solution space through the network during co-consumption. Details of the simulations are given in Tables S1, S2. A solution could be found under the standard deviation of the measured constraints (growth rate, substrate uptake rates and CO_2_ production rate), indicating that the carbon balance is closed. The calculated methanol and succinate contributions to ATP, NADH, and NADPH flux production for a feasible solution are displayed in Figure 5, and detailed values can be found in Table S3. Note that no single solution exists; rather, multiple satisfactory flux distributions can be predicted. Consequently, a flux variability analysis was performed to analyze the flux solution space under the measured constraints (see Table S4 for detailed results). The calculated flux distribution predicted that methanol oxidation provided 58–74% of the ATP, showing that methanol is the main energy source. ATP is produced exclusively by oxidative phosphorylation, which is fueled by NADH for 73% of reactions, methanol dehydrogenase (cytochrome-dependent) for 17% of reactions, and succinate dehydrogenase (ubiquinone-dependent) for 10% of reactions (see Table S5 for detailed calculations). Methanol dissimilation contributes to 66% of NADH generation (min 0.46 and max 0.76), whereas succinate oxidation, i.e., oxidative operation of the TCA cycle plus pyruvate dehydrogenase, and the pentose phosphate pathway supply only 9% of the NADH needs. Nonetheless, an additional 25% of NADH molecules are produced from succinate during biomass precursor formation, which is higher than the 7% required for their production and thus results in net energy production. Interestingly, some of the NADH can be converted to NADPH via transhydrogenase (0–3.28 mmol·g^−1^·h^−1^), indicating that an excess of NADH may result from co-consumption.

## Discussion

The data reported here show that *M. extorquens* AM1 co-consumes succinate and methanol under mixed substrate conditions. This result is something of a surprise in light of prior knowledge about the adaptation capacity of this model methylotroph. Indeed, the two pure culture conditions that were studied intensively in the past, methanol versus succinate, rely on the use of specific metabolic pathways on one hand and on driving metabolic fluxes in opposite direction through common enzyme steps, like the serine cycle, on the other hand. Consequently, rerouting the metabolic fluxes in a backward direction through some reactions involves a strong reprogramming of the central metabolism to achieve the metabolic switch between succinate and methanol [23]. In the past, this fundamental difference in central metabolic pathway usage between succinate growth and methanol growth conditions provided the basis of the successful identification of the genes involved under both conditions [30], and these became the two standard conditions under which to study the metabolism under methylotrophic and non-methylotrophic conditions in multiple biochemical and omics experiments [15], [17], [18], [19], [31]. Here, we showed that *M. extorquens* AM1 co-consumes methanol in addition to succinate, and a specific metabolic network takes place that relies on the previously demonstrated modularity [14] and flexibility [20], [21], [22] of the central metabolism of the model methylotroph. ^13^C-labeling revealed the partitioning of methanol and succinate to specific metabolic pathways and functions upon co-consumption. Methanol was mainly used to fulfill energy requirements; however, a portion of the consumed methanol entered biosynthetic pathways. The most promiscuous pathways were the biosynthetic pathways linked to one-carbon metabolism, such as purine biosynthesis from tetrahydrofolate derivatives; in addition, few carbons entered gluconeogenesis via C1 precursors and the first step of the serine cycle. In contrast, under mixed substrate conditions, succinate was used primarily to fulfill the carbon requirements of the cell and to supply roughly one-third of the energy requirements. Half of this energy is produced concomitantly with biomass precursor formation.

The mechanism allowing the observed partitioning through the central metabolism involved the repression of methanol assimilation, not at the level of C1 activation but at the first step of the serine cycle. In addition, CO_2_ assimilation via the phosphoenolpyruvate carboxylase and ethylmalonyl-CoA pathways, which represent 50% of the carbon assimilated into the biomass during pure methylotrophic growth conditions [20], appears to be blocked. Establishing this state of the network requires the coordinated control of several key points of the central metabolism by the cell. The regulation at the enzyme activity level was in part revealed by the enzyme activity measurements performed by Dunstan *et al.* [27]. In this study, the methanol dehydrogenase activity of *M. extorquens* AM1 grown in the presence of methanol and succinate was found to be significant, and no catabolic repression of the encoding gene occurred. Secondly, lower activities for the first enzymes of the anabolic serine cycle (i.e., serine-glyoxylate aminotransferase, hydroxypyruvate reductase and glycerate kinase) compared to methylotrophic growth were found [27], although its activities were significantly higher than under pure succinate incubation. These observations corroborate and explain the observed flux partitioning at the first step of the serine cycle, where methanol assimilation flow is mainly, but not entirely, restricted to C1-precursor biosynthesis. This specific regulation, which takes place during co-consumption of methanol plus succinate, is likely to rely on an active process for the activation and repression of a specific set of enzymes. The regulation of genes for enzymes involved in C1 dissimilation and C1 assimilation are at least partially known and are dependent on distinct regulators [32]. Some C1 dissimilation genes appear to be under the control of the methanol concentration [33], indicating that methanol utilization is not catabolically repressed by the presence of succinate. Note that this mechanism is crucial for methanol and formaldehyde detoxification. The genes responsible for C1 assimilation are clustered in 2 main operons and are regulated by a Lys-R-type transcriptional regulator (QscR) [32], [34], [35]. QscR is known to be expressed at low levels under both methanol and succinate conditions and responds to physiological signals. Indeed, the DNA binding of the regulator is enhanced by the presence of formyl-H_4_F [35] but reduced by NADP^+^, acetyl-CoA, and weakly reduced by glyoxylate [34]. On one hand, the formyl-H_4_F concentration is expected to increase under methanol utilization, which could enhance C1 precursor biosynthesis; on the other hand, a change in the energetic state of the cell (NADP+/NADPH) [23], [31] and the concentrations of acetyl-CoA and glyoxylate, two key metabolites of the serine cycle, could induce a decrease in enzyme activities [31]. In addition, the regulator of the main enzyme of the ethylmalonyl-CoA pathway, crotonyl-CoA carboxylase/reductase, was recently identified [36]. A complex mechanism of regulation of the genes encoding the metabolic enzymes involved in C1 dissimilation and assimilation allows these processes to be decoupled, for instance, during co-consumption (this study) or during a substrate switch [23]. In fact, the mechanism of C1 pathway regulation in *M. extorquens* AM1, in addition to the high modularity of the central metabolic pathways, is crucial to enable the co-consumption of a C1 substrate (methanol) and a C4 organic acid (succinate).

The metabolic strategy of co-consumption contrasts with the intensively studied phenomenon of diauxic and catabolic repression [24], [26], [37]. Several hypotheses could explain co-consumption by *M. extorquens* AM1 rather than the successive use of succinate and methanol. First, it could be a strategy of resource management under environmental conditions. Indeed, methanol is only transiently released by plants during the diurnal cycle [9], and it is a volatile compound; therefore its co-consumption would lead to a delay in the exhaustion of succinate (or other organic acids) when methanol is available and assure the cell of prolonged substrate availability. In addition, except during the peak of emission in the morning, methanol is emitted at a low rate during the day. This might not be sufficient to sustain growth, such that an additional carbon substrate would consequently be required. Another point of consideration is that the growth rates during pure succinate and pure methanol growth are relatively similar, and mixed substrate conditions result in comparable growth. Likewise, *Corynebacterium glutamicum* exhibits identical growth rates during growth on glucose and acetate and co-consumes both substrates at the same growth rate under mixed substrate conditions [38]. Thus, both *Methylobacterium* and *Corynebacterium* reduce the uptake rates of both substrates under mixed substrate conditions. In contrast, *Bacillus subtilis*, which is able to grow on a mixture of glucose and malate, exhibits a higher growth rate during co-consumption than during pure culture conditions [39]. Interestingly, for *Bacillus subtilis,* both glucose and malate catabolically repress several other substrates [39]. Thus, similar growth rates on different substrates might lead to the convergence of substrate utilization rather than diauxie. The latter is the case for *Escherichia coli* and *Azotobacter vinelandii,* which exhibit different growth rates on glucose and acetate, respectively [40], [41]. Mixed substrate conditions result in a diauxic shift in which by *E. coli* consumes glucose first and *A. vinelandii* switches from acetate to glucose metabolization.

Nonetheless, the co-consumption of methanol and succinate in *M. extorquens* could be a strategy to optimize substrate utilization because methanol theoretically has a higher C-mol yield of energy units (ATP, NADH, and NADPH) as well as C1 units (∼ 150%) compared to succinate but only a slightly higher C-mol yield for other biomass production (115%), based on yield calculations using a genome-scale model [20]. Thus, the partitioning observed constrains methanol to its most efficient utilization pattern. However, similar biomass yields were observed experimentally under each condition (methanol, succinate, and methanol plus succinate), indicating that no substantial gain resulted from the substrate partitioning.

Addressing the role of co-consumption challenges our current ability to measure cell physiology in situ (*in planta*). The results presented here for the co-consumption of methanol plus a C4 organic acid by *M. extorquens* AM1 might suggest that heterotrophy could be as important as methylotrophy for leaf colonization capacity by *Methylobacterium* and is in line with the finding that methylotrophic bacteria in the phyllosphere are facultative methylotrophs rather than obligate methylotrophs [3], [42]. Thus, co-consumption might be a prevailing strategy under environmental conditions, where substrate availability is scarce, transient and diverse.

## Materials and Methods

### Chemicals

[^13^C] methanol (99%) was purchased from Cambridge Isotope Laboratories; all other chemicals were purchased from Sigma (St. Louis, MO, USA). The acetonitrile, formic acid, and ammonium used as HPLC solvents were of LC-MS grade.

### Medium Composition, Culture Conditions, and Growth Parameter Measurement

The minimal medium used to grow *M. extorquens* AM1 contained 1.62 g·L^−1^ NH_4_Cl, 0.2 g·L^−1^ MgSO_4_, 0.22 g·L^−1^ K_2_HPO_4_, 0.13 g·L^−1^ NaH_2_PO_4_·2H_2_O, and the following trace elements: 15 mg·L^−1^ Na_2_EDTA_2_·H_2_O, 4.5 mg·L^−1^ ZnSO_4_·7H_2_O, 3 mg·L^−1^ CoCl_2_·6H_2_O, 0.6 mg·L^−1^ MnCl_2_, 1 mg·L^−1^ H_3_BO_3_, 3.0 mg·L^−1^ CaCl_2_, 0.4 mg·L^−1^ Na_2_MoO_4_·2H_2_O, 3 mg·L^−1^ FeSO_4_·7H_2_O, and 0.3 mg·L^−1^ CuSO_4_ 5H_2_O. Batch-culture was carried out in a 500-mL bioreactor (Infors-HT) at 28°C and at 1000 rpm, aerated with compressed air at 0.1 L·min^−1^. The pH was kept constant at 7.0 by the addition of 1 M NH_4_OH or 0.5 M H_2_SO_4_. Cells were grown in 400 mL of medium containing a mixture of 60 mM methanol plus 15 mM succinic acid (equivalent C-moles of each carbon source). The partial pressure of dissolved oxygen was monitored using polarographic oxygen sensors (InPro 6800, Mettler-Toledo). The methanol concentration was determined by GC-flame ionization detection (GC-FID) (GC 6850, Agilent Technologies; column: DB-Wax, J&W Scientific). The succinate concentration was determined by HPLC-UV-DAD (column: Phenomenex Rezex ROA-organic acid H+7.8 mm) using tartaric acid as an internal standard. The ^13^C enrichment of CO_2_ in the exhaust gas was measured using two infrared sensors (BCP-CO2, BlueSens), one sensitive to ^12^C CO_2_ and the other sensitive to ^13^C CO_2_. The calibration of each sensor and the specific correction of ^12^C and ^13^C signals were performed as recommended by the company. Cell dry weight (CDW) was determined upon growth on each substrate (methanol, methanol plus succinate, succinate). The results of 7 cultures were not significantly different, and an overall CDW value was found to be 0.269±0.013 (2σ).

### Sampling, Quenching, and Extraction of Intracellular Metabolites

CoA-ester sampling was performed as follows: a volume of 1 mL of culture was directly injected into 4.5 mL of −20°C cold acidified acetonitrile containing 0.1 M formic acid on a Vortex [12]. The extraction was performed with the sample incubated for 15 min on ice and subsequently freeze-dried and stored at −20°C until analysis. Prior to analysis, dried samples were dissolved in 300 µL of 25 mM ammonium formate buffer (pH 3.5, 2% MeOH). The suspension was centrifuged (14,000 *g*, 2 min, −5°C), and the supernatant was filtered through a Sartorius Minisart filter (pore size 0.2 µm) before analysis. Amino acids and central metabolites were sampled as described previously [19]. In brief, 1 mL of culture was sampled by fast filtration and washed with 5 ml medium. The filters (RC Sartorius Minisart, pore size 0.2 µm) were directly transferred into shot bottles containing 8 ml of boiling water for quenching and extraction. The extracts were cooled on ice and filtered via a RC Sartorius Minisart filter (pore size 0.2 µm) and then chilled with liquid nitrogen. All samples were lyophilized immediately and stored at −20°C. Dried samples were dissolved in 100 µl double-distilled water and diluted 30/70 (v/v) with acetonitrile prior to analysis.

### LC-MS Analysis

LC-MS analysis was performed using a Rheos 2200 HPLC system (Flux Instruments) coupled to an LTQ Orbitrap mass spectrometer (Thermo Fisher Scientific) and equipped with an electrospray ionization probe. CoA-thioesters were separated using a previously described procedure [12], with slight modifications [43]. Polar intracellular metabolites were separated on a pHILIC column (150×2.0 mm, particle size 5 µm; Sequant, Umea, Sweden) as previously described [19]. Phosphorylated hexoses were not separated; thus, the data given are an average of the hexose-phosphate pool.

The LC-MS system was equilibrated for 6 min under initial elution conditions between two successive analyses. The LC was coupled to the mass spectrometer. The sheath gas flow rate was 40, the auxiliary gas flow rate was 30, the tube lens was 80 V, the capillary voltage was 35 V, and the ion spray voltage was 4.3 kV. MS analysis was performed in the FTMS positive mode to analyze CoA esters and amino acids and in the negative FTMS mode for all other compounds at a resolution of 60,000 (m/z 400).

### Data Analysis

The incorporation of ^13^C label into metabolites during the ^13^C-labeling experiment was calculated from the analysis of the mass isotopomer distribution (MID) in the mass spectra. The resolution of 60,000 (m/z 400) allowed the separation of carbon, nitrogen and oxygen mass isotopomers; therefore, only carbon MIDs were considered, and correction for naturally occurring isotopes of the other elements was not required for most metabolites. However, the mass resolution of the Orbitrap decreases with increasing m/z values. In case of co-esters (m/z >800), analysis yielded mass resolution below 42,000, resulting in incomplete separation of the isotopes requiring correction for the contributions of N, O and S to MID. The standard deviations (STD) of the measurements were considered to be at least 2%, higher than found over the 3 technical replicates (average STD in amino acids: 0.53%). This was likely due to systematic error resulting from lower linearity of the LTQ-Orbitrap, which was evaluated to be lower than 2% in the intensity range considered. The average ^13^C labeling AL_13C_ was calculated as follows:
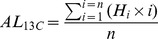
n = number of carbon atoms, H_i_ relative abundance of the monoisotopic mass + i fraction.

### Calculation of Methanol and Succinate Contribution to Energetics

Feasibility analysis and Flux variability analysis was performed using the genome-scale metabolic network of *M. extorquens* AM1 [20]. Calculations were performed using CellNetAnalyser [44] based on the *M. extorquens* AM1 biomass composition determined during methylotrophic growth [20] and under the constraints (upper and lower flux limits were fixed corresponding to 2 standard deviations) of the measured fluxes (uptake and production rate, growth rate); see Table S1 for details. All exchange fluxes of carbon sources except methanol and succinate were set to 0, and a network reduction step was applied as described by Peyraud *et al.* [20] to make accurate predictions. Therefore, the identified methylotrophic sub-network [20] was amended by the opening reactions specifically used under succinate conditions (Table S2). The contributions of methanol and succinate to cell energetics were calculated from a feasible solution (Table S3) under the given constraints, and their deviation was performed by Flux Variability Analysis (Table S4); see Table S5 for calculation results.

## Supporting Information

Figure S1 **Mass isotopomers distribution of central metabolites measured by LC-MS of** ***M. extorquens*** **AM1 upon co-consumption with ^13^C (>99%) methanol and natural abundance (1.1% ^13^C) succinate.** Mass isotopomer data in black correspond to samples collected during mid-co-consumption phase (Sampling time 1, see Fig. 1), and in red to samples collected at the end of the co-consumption phase (Sampling time 2).
(PDF)

Figure S2 **Average ^13^C labeling in intra-cellular metabolites measured by LC-MS of** ***M. extorquens*** **AM1 at 90 minutes after succinate exhaustion (Sampling time 3 on** **Fig. 1** **).**
(PDF)

Figure S3 **Mass isotopomers distribution of central metabolites measured by LC-MS of** ***M. extorquens*** **AM1 at 90 minutes after succinate exhaustion (Sampling time 3 of** **Fig. 1** **).**
(PDF)

Figure S4 **Mass isotopomers distribution in CoA thioesters measured by LC-MS during growth of** ***M.extorquens*** **AM1 upon co-consumption with ^13^C (>99%) methanol and natural abundance (1.1% ^13^C) succinate.** Metabolite quenching, extraction and measurements were performed as described in material and methods. Mass isotopomer data correspond to sample collected during mid-co-consumption phase (Sampling time 1, see Fig. 1).
(PDF)

Figure S5 **Scheme of the Coenzyme A biosynthesis in** ***M. extorquens*** **AM1.** Identified ^13^C carbon entry points are indicated by colored cycles. Red, C1-precursor from tetrahydrofolate pathway; yellow, CO_2_; green, C3 carbon of serine.
(PDF)

Table S1 **Lists of parameters used for flux balance analysis and flux variability analysis.**
(PDF)

Table S2 **List of the reactions reduced for flux balance analysis upon co-consumption condition.**
(PDF)

Table S3 **Fluxes solution of the feasibility analysis.**
(PDF)

Table S4 **Fluxes solution of the flux variability analysis.**
(PDF)

Table S5 **Details of energetics calculations from flux balance analysis.**
(PDF)

## References

[1] Egli T, Mason CA (1991) Mixed substrates and mixed cultures. Biotechnology 18: 173–201. 1909913 10.1016/b978-0-7506-9188-8.50015-2

[2] Corpe WA, Rheem S (1989) Ecology of the methylotrophic bacteria on living leaf surfaces. FEMS Microbiology Letters 62: 243–249.

[3] Delmotte N, Knief C, Chaffron S, Innerebner G, Roschitzki B, et al (2009) Community proteogenomics reveals insights into the physiology of phyllosphere bacteria. Proc Natl Acad Sci U S A 106: 16428–16433. 19805315 10.1073/pnas.0905240106 PMC2738620

[4] Knief C, Delmotte N, Chaffron S, Stark M, Innerebner G, et al (2012) Metaproteogenomic analysis of microbial communities in the phyllosphere and rhizosphere of rice. ISME J 6: 1378–1390. 22189496 10.1038/ismej.2011.192 PMC3379629

[5] Lindow SE, Brandl MT (2003) Microbiology of the phyllosphere. Appl Environ Microbiol 69: 1875–1883. 12676659 10.1128/AEM.69.4.1875-1883.2003 PMC154815

[6] Abanda-Nkpwatt D, Musch M, Tschiersch J, Boettner M, Schwab W (2006) Molecular interaction between *Methylobacterium extorquens* and seedlings: growth promotion, methanol consumption, and localization of the methanol emission site. J Exp Bot 57: 4025–4032. 17043084 10.1093/jxb/erl173

[7] Miller WG, Brandl MT, Quinones B, Lindow SE (2001) Biological sensor for sucrose availability: relative sensitivities of various reporter genes. Appl Environ Microbiol 67: 1308–1317. 11229926 10.1128/AEM.67.3.1308-1317.2001 PMC92729

[8] Leveau JH, Lindow SE (2001) Appetite of an epiphyte: quantitative monitoring of bacterial sugar consumption in the phyllosphere. Proc Natl Acad Sci U S A 98: 3446–3453. 11248098 10.1073/pnas.061629598 PMC30673

[9] Huve K, Christ MM, Kleist E, Uerlings R, Niinemets U, et al (2007) Simultaneous growth and emission measurements demonstrate an interactive control of methanol release by leaf expansion and stomata. J Exp Bot 58: 1783–1793. 17374874 10.1093/jxb/erm038

[10] Sy A, Timmers AC, Knief C, Vorholt JA (2005) Methylotrophic metabolism is advantageous for *Methylobacterium extorquens* during colonization of *Medicago truncatula* under competitive conditions. Appl Environ Microbiol 71: 7245–7252. 16269765 10.1128/AEM.71.11.7245-7252.2005 PMC1287603

[11] Chistoserdova L, Vorholt JA, Thauer RK, Lidstrom ME (1998) C1 transfer enzymes and coenzymes linking methylotrophic bacteria and methanogenic Archaea. Science 281: 99–102. 9651254 10.1126/science.281.5373.99

[12] Peyraud R, Kiefer P, Christen P, Massou S, Portais JC, et al (2009) Demonstration of the ethylmalonyl-CoA pathway by using 13C metabolomics. Proc Natl Acad Sci U S A 106: 4846–4851. 19261854 10.1073/pnas.0810932106 PMC2660752

[13] Anthony C (2011) How half a century of research was required to understand bacterial growth on C1 and C2 compounds; the story of the serine cycle and the ethylmalonyl-CoA pathway. Sci Prog 94: 109–137. 21805909 10.3184/003685011X13044430633960 PMC10365475

[14] Chistoserdova L (2011) Modularity of methylotrophy, revisited. Environ Microbiol 13: 2603–2622. 21443740 10.1111/j.1462-2920.2011.02464.x

[15] Okubo Y, Skovran E, Guo X, Sivam D, Lidstrom ME (2007) Implementation of microarrays for Methylobacterium extorquens AM1. Omics 11: 325–340. 18092906 10.1089/omi.2007.0027

[16] Laukel M, Rossignol M, Borderies G, Volker U, Vorholt JA (2004) Comparison of the proteome of *Methylobacterium extorquens* AM1 grown under methylotrophic and nonmethylotrophic conditions. Proteomics 4: 1247–1264. 15188393 10.1002/pmic.200300713

[17] Bosch G, Skovran E, Xia Q, Wang T, Taub F, et al (2008) Comprehensive proteomics of *Methylobacterium extorquens* AM1 metabolism under single carbon and nonmethylotrophic conditions. Proteomics 8: 3494–3505. 18686303 10.1002/pmic.200800152 PMC2707879

[18] Guo X, Lidstrom ME (2008) Metabolite profiling analysis of *Methylobacterium extorquens* AM1 by comprehensive two-dimensional gas chromatography coupled with time-of-flight mass spectrometry. Biotechnol Bioeng 99: 929–940. 17879968 10.1002/bit.21652

[19] Kiefer P, Portais JC, Vorholt JA (2008) Quantitative metabolome analysis using liquid chromatography-high-resolution mass spectrometry. Anal Biochem 382: 94–100. 18694716 10.1016/j.ab.2008.07.010

[20] Peyraud R, Schneider K, Kiefer P, Massou S, Vorholt JA, et al (2011) Genome-scale reconstruction and system level investigation of the metabolic network of *Methylobacterium extorquens* AM1. BMC Syst Biol 5: 189. 22074569 10.1186/1752-0509-5-189 PMC3227643

[21] Van Dien SJ, Okubo Y, Hough MT, Korotkova N, Taitano T, et al (2003) Reconstruction of C(3) and C(4) metabolism in *Methylobacterium extorquens* AM1 using transposon mutagenesis. Microbiology 149: 601–609. 12634329 10.1099/mic.0.25955-0

[22] Schneider K, Peyraud R, Kiefer P, Christen P, Delmotte N, et al (2012) The ethylmalonyl-CoA pathway is used in place of the glyoxylate cycle by *Methylobacterium extorquens* AM1 during growth on acetate. J Biol Chem 287: 757–766. 22105076 10.1074/jbc.M111.305219 PMC3249130

[23] Skovran E, Crowther GJ, Guo X, Yang S, Lidstrom ME (2010) A systems biology approach uncovers cellular strategies used by *Methylobacterium extorquens* AM1 during the switch from multi- to single-carbon growth. PLoS One 5: e14091. 21124828 10.1371/journal.pone.0014091 PMC2991311

[24] Beckwith JR (1967) Regulation of the lac operon. Recent studies on the regulation of lactose metabolism in *Escherichia coli* support the operon model. Science 156: 597–604. 5337175 10.1126/science.156.3775.597

[25] Jacob F, Monod J (1961) Genetic regulatory mechanisms in the synthesis of proteins. J Mol Biol 3: 318–356. 13718526 10.1016/s0022-2836(61)80072-7

[26] Deutscher J (2008) The mechanisms of carbon catabolite repression in bacteria. Curr Opin Microbiol 11: 87–93. 18359269 10.1016/j.mib.2008.02.007

[27] Dunstan PM, Anthony C, Drabble WT (1972) Microbial metabolism of C 1 and C 2 compounds. The role of glyoxylate, glycollate and acetate in the growth of *Pseudomonas* AM1 on ethanol and on C 1 compounds. Biochem J 128: 107–115. 5085544 10.1042/bj1280107 PMC1173575

[28] Anandham R, Indiragandhi P, Madhaiyan M, Chung J, Ryu KY, et al (2009) Thiosulfate Oxidation and mixotrophic growth of *Methylobacterium goesingense* and *Methylobacterium fujisawaense* . J Microbiol Biotechnol 19: 17–22. 19190404

[29] Erb TJ, Berg IA, Brecht V, Muller M, Fuchs G, et al (2007) Synthesis of C5-dicarboxylic acids from C2-units involving crotonyl-CoA carboxylase/reductase: the ethylmalonyl-CoA pathway. Proc Natl Acad Sci U S A 104: 10631–10636. 17548827 10.1073/pnas.0702791104 PMC1965564

[30] Chistoserdova L, Chen SW, Lapidus A, Lidstrom ME (2003) Methylotrophy in *Methylobacterium extorquens* AM1 from a genomic point of view. J Bacteriol 185: 2980–2987. 12730156 10.1128/JB.185.10.2980-2987.2003 PMC154073

[31] Guo X, Lidstrom ME (2006) Physiological analysis of *Methylobacterium extorquens* AM1 grown in continuous and batch cultures. Arch Microbiol 186: 139–149. 16821027 10.1007/s00203-006-0131-7

[32] Vuilleumier S, Chistoserdova L, Lee MC, Bringel F, Lajus A, et al (2009) *Methylobacterium* genome sequences: a reference blueprint to investigate microbial metabolism of C1 compounds from natural and industrial sources. PLoS One 4: e5584. 19440302 10.1371/journal.pone.0005584 PMC2680597

[33] Springer AL, Auman AJ, Lidstrom ME (1998) Sequence and characterization of *mxaB*, a response regulator involved in regulation of methanol oxidation, and of *mxaW*, a methanol-regulated gene in *Methylobacterium extorquens* AM1. FEMS Microbiol Lett 160: 119–124. 9495022 10.1111/j.1574-6968.1998.tb12900.x

[34] Kalyuzhnaya MG, Lidstrom ME (2003) QscR, a LysR-type transcriptional regulator and CbbR homolog, is involved in regulation of the serine cycle genes in *Methylobacterium extorquens* AM1. J Bacteriol 185: 1229–1235. 12562792 10.1128/JB.185.4.1229-1235.2003 PMC142849

[35] Kalyuzhnaya MG, Lidstrom ME (2005) QscR-mediated transcriptional activation of serine cycle genes in *Methylobacterium extorquens* AM1. J Bacteriol 187: 7511–7517. 16237034 10.1128/JB.187.21.7511-7517.2005 PMC1272982

[36] Hu B, Lidstrom M (2012) CcrR, a TetR family transcriptional regulator, activates the transcription of a gene of the Ethylmalonyl coenzyme a pathway in *Methylobacterium extorquens* AM1. J Bacteriol 194: 2802–2808. 22447902 10.1128/JB.00061-12 PMC3370644

[37] Monod J (1949) The growth of bacterial cultures. Annu Rev Microbiol 3: 371–394.

[38] Wendisch VF, de Graaf AA, Sahm H, Eikmanns BJ (2000) Quantitative determination of metabolic fluxes during coutilization of two carbon sources: comparative analyses with *Corynebacterium glutamicum* during growth on acetate and/or glucose. J Bacteriol 182: 3088–3096. 10809686 10.1128/jb.182.11.3088-3096.2000 PMC94493

[39] Kleijn RJ, Buescher JM, Le Chat L, Jules M, Aymerich S, et al (2010) Metabolic fluxes during strong carbon catabolite repression by malate in *Bacillus subtilis* . J Biol Chem 285: 1587–1596. 19917605 10.1074/jbc.M109.061747 PMC2804316

[40] Wolfe AJ (2005) The acetate switch. Microbiol Mol Biol Rev 69: 12–50. 15755952 10.1128/MMBR.69.1.12-50.2005 PMC1082793

[41] Tauchert K, Jahn A, Oelze J (1990) Control of diauxic growth of *Azotobacter vinelandii* on acetate and glucose. J Bacteriol 172: 6447–6451. 2228968 10.1128/jb.172.11.6447-6451.1990 PMC526832

[42] Knief C, Frances L, Vorholt JA (2010) Competitiveness of diverse *Methylobacterium* strains in the phyllosphere of *Arabidopsis thaliana* and identification of representative models, including *M. extorquens* PA1. Microb Ecol 60: 440–452. 20700590 10.1007/s00248-010-9725-3

[43] Kuntze K, Kiefer P, Baumann S, Seifert J, von Bergen M, et al (2011) Enzymes involved in the anaerobic degradation of meta-substituted halobenzoates. Mol Microbiol 82: 758–769. 22010634 10.1111/j.1365-2958.2011.07856.x

[44] Klamt S, Saez-Rodriguez J, Gilles ED (2007) Structural and functional analysis of cellular networks with CellNetAnalyzer. BMC Syst Biol 1: 2. 17408509 10.1186/1752-0509-1-2 PMC1847467

