# Supplementary figures and images for: Co-Consumption of Methanol and Succinate by Methylobacterium extorquens AM1
Source: PLoS One. 2012 Nov 1;7(11):e48271. doi: 10.1371/journal.pone.0048271 (PMC3486813; doi:10.1371/journal.pone.0048271)

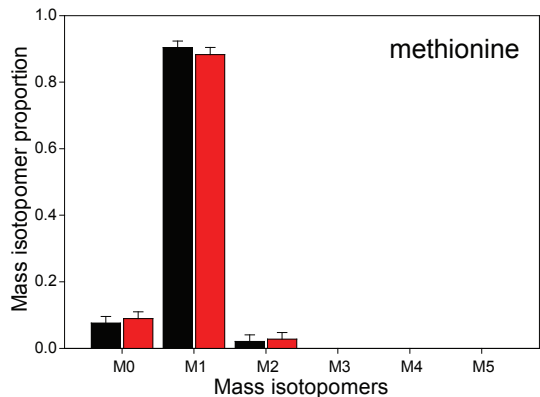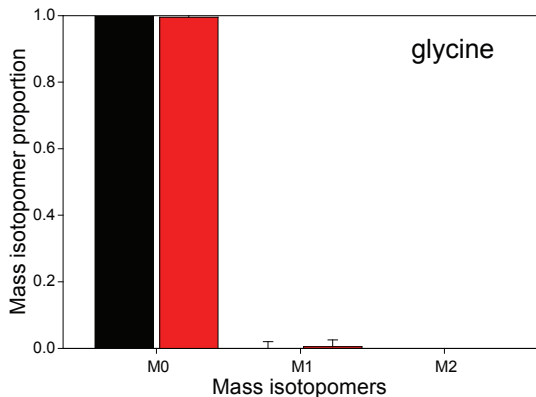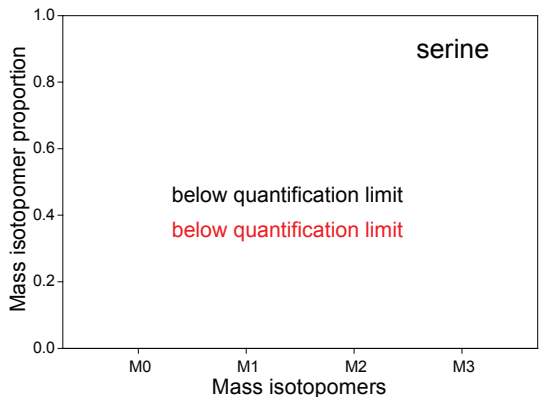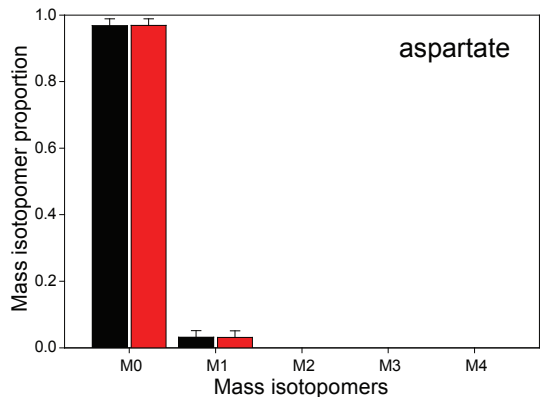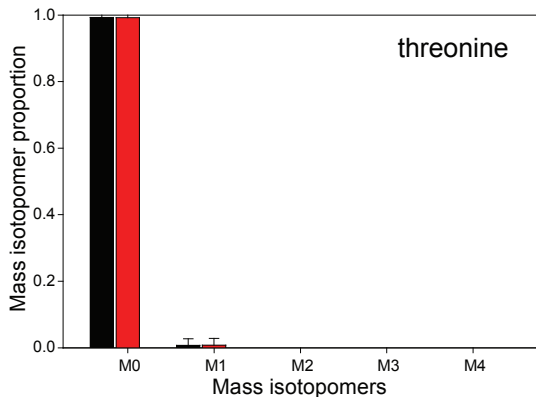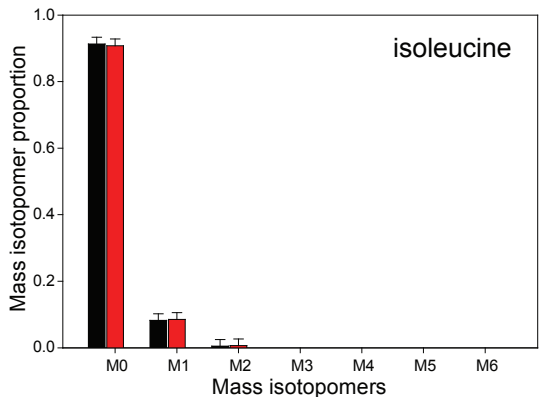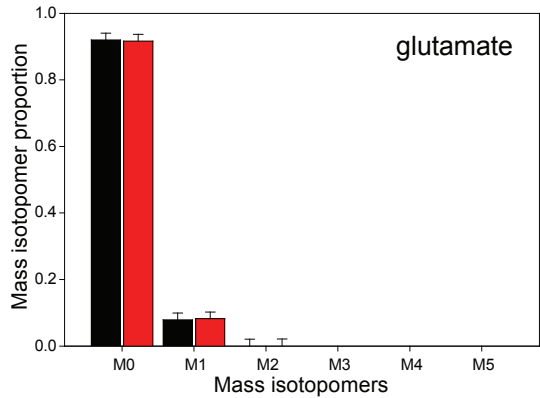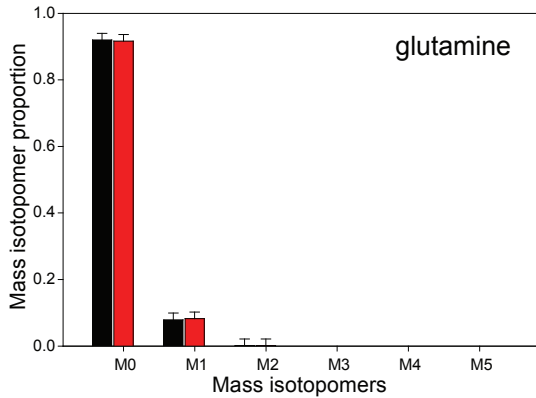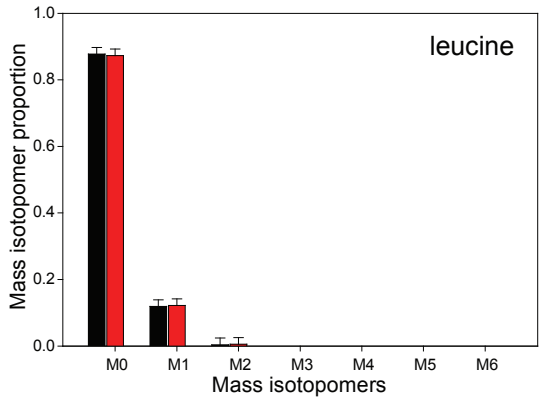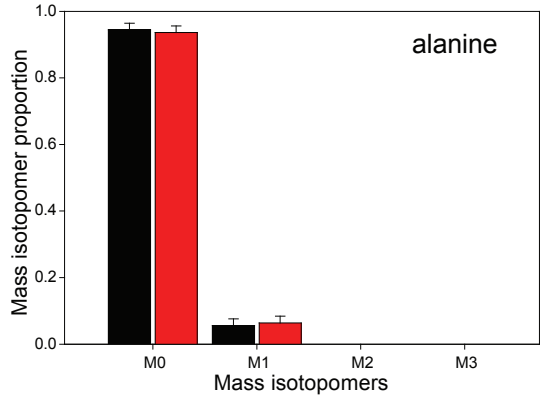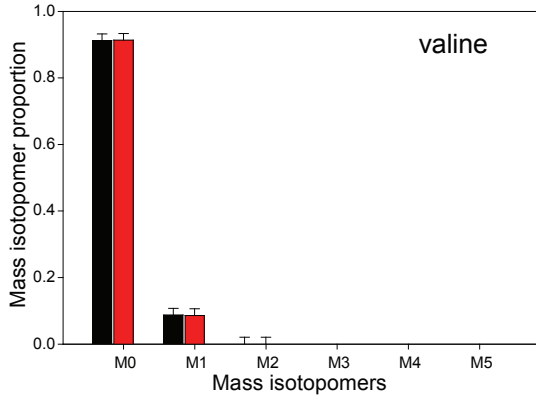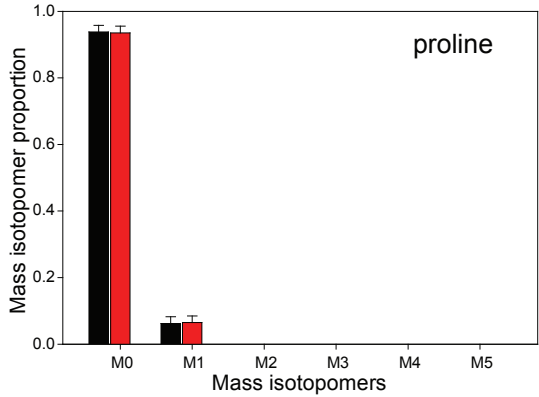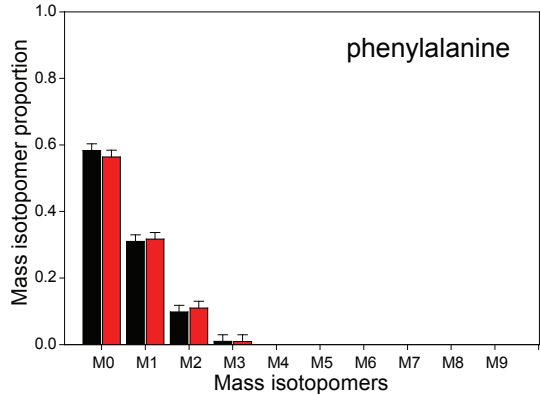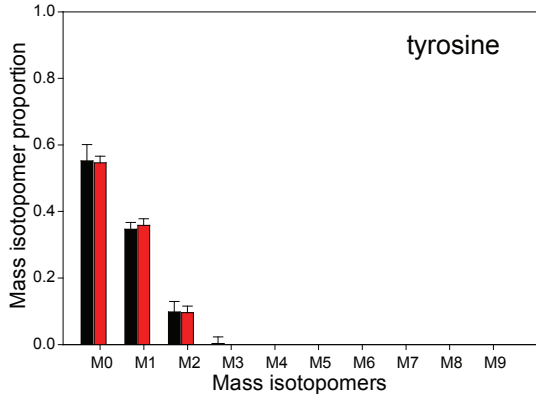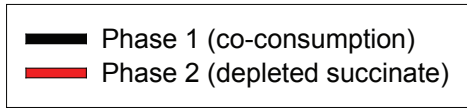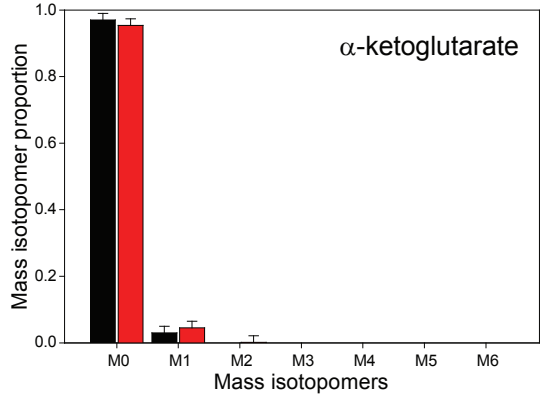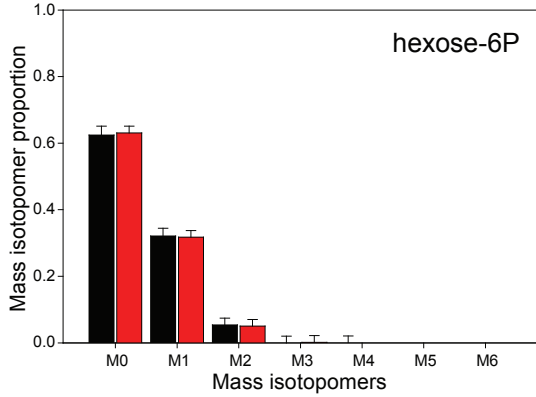

Supplement: Figure S1 — Mass isotopomers distribution of central metabolites measured by LC-MS of M. extorquens AM1 upon co-consumption with 13C (>99%) methanol and natural abundance (1.1% 13C) succinate. Mass isotopomer data in black correspond to samples collected during mid-co-consumption phase (Sampling time 1, see Fig. 1), and in red to samples collected at the end of the co-consumption phase (Sampling time 2). (PDF) [file pone.0048271.s001.pdf]

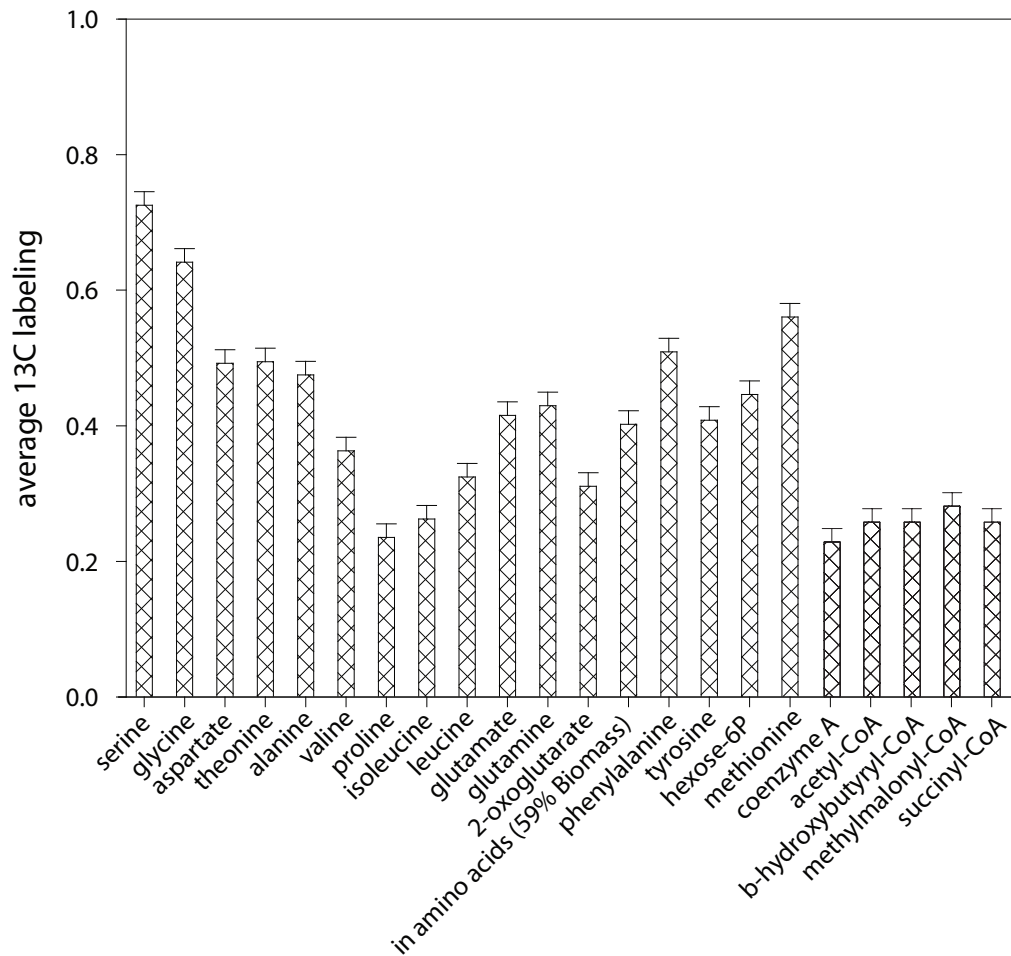

Supplement: Figure S2 — Average 13C labeling in intra-cellular metabolites measured by LC-MS of M. extorquens AM1 at 90 minutes after succinate exhaustion (Sampling time 3 on Fig. 1 ). (PDF) [file pone.0048271.s002.pdf]

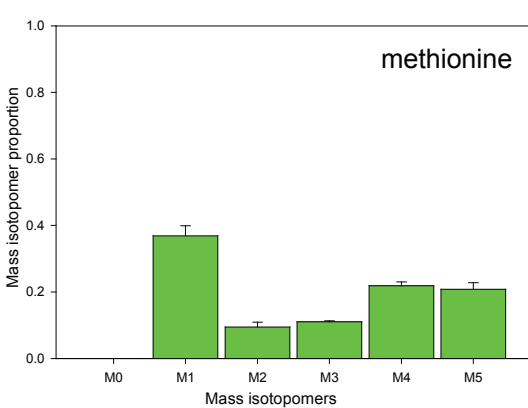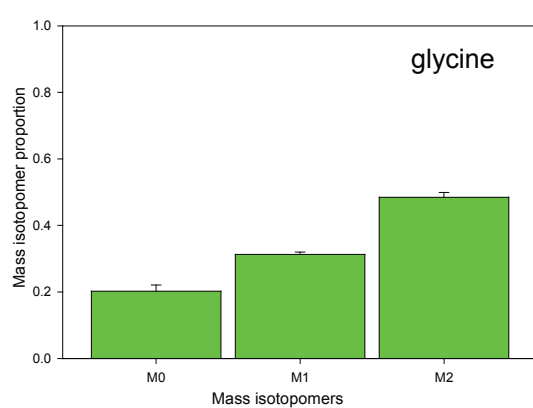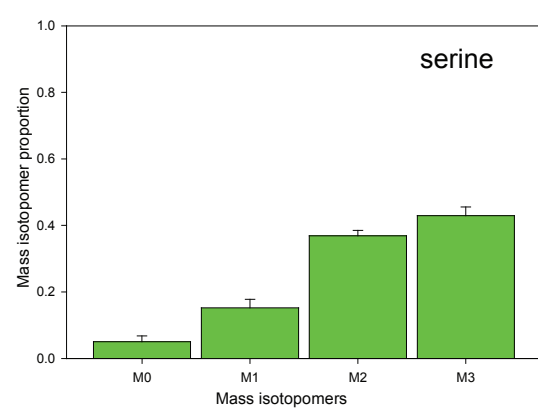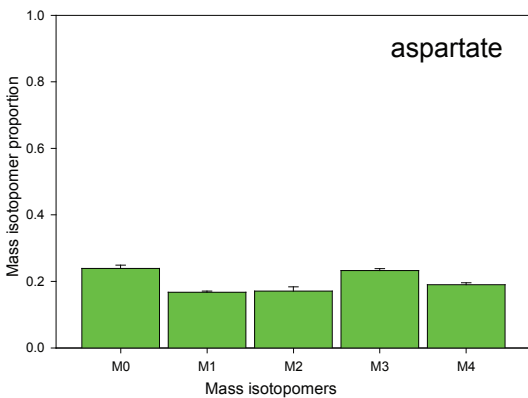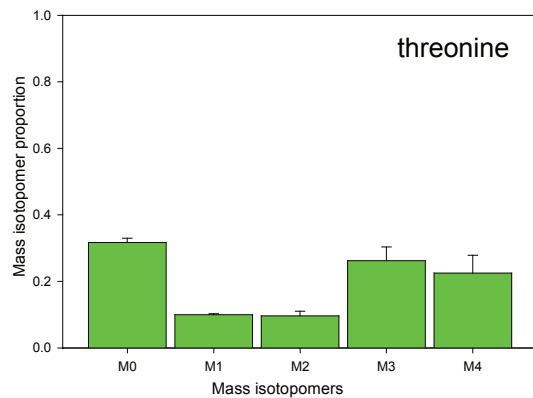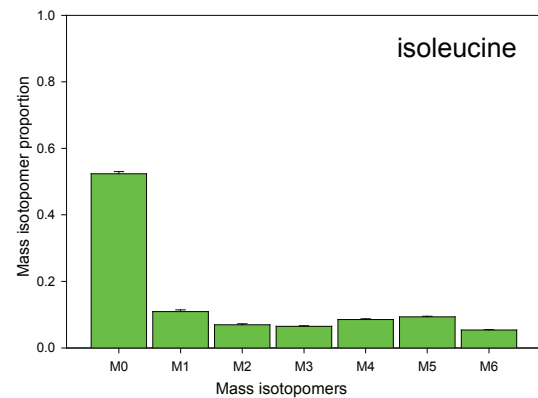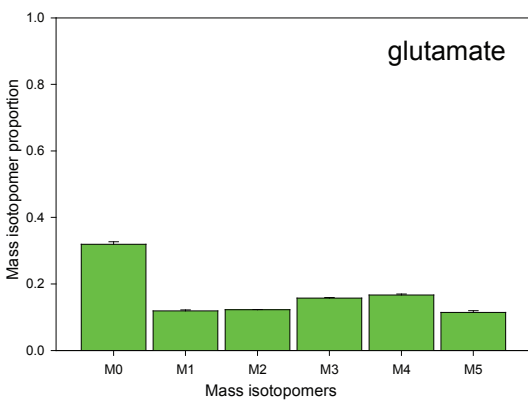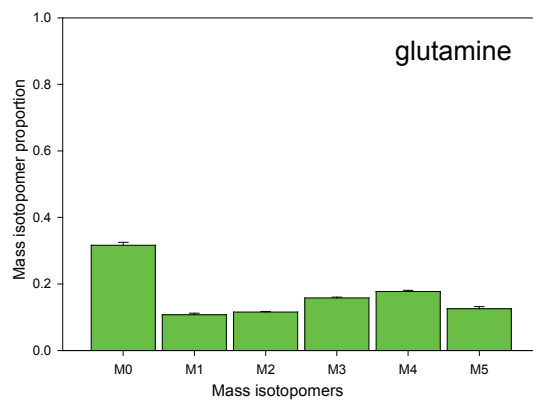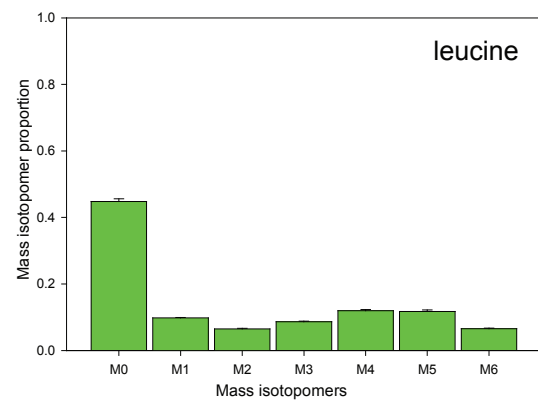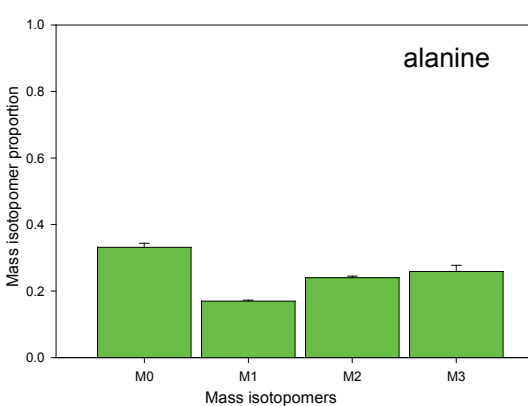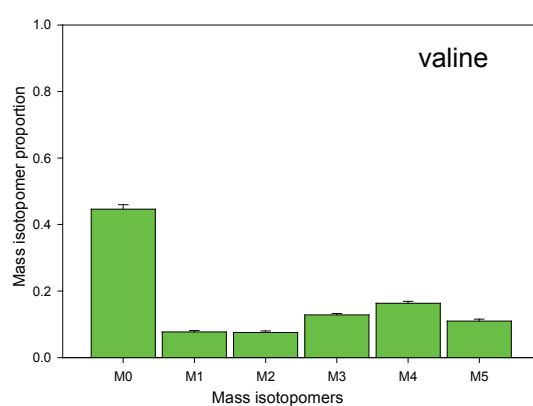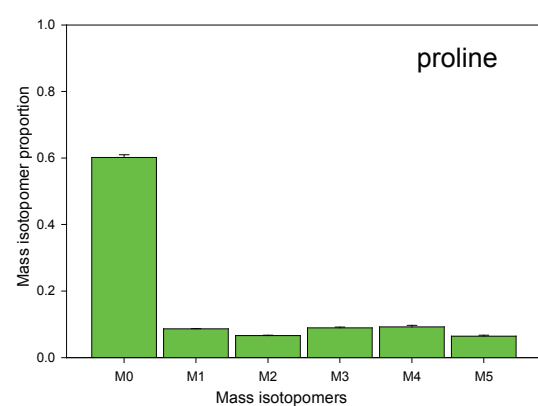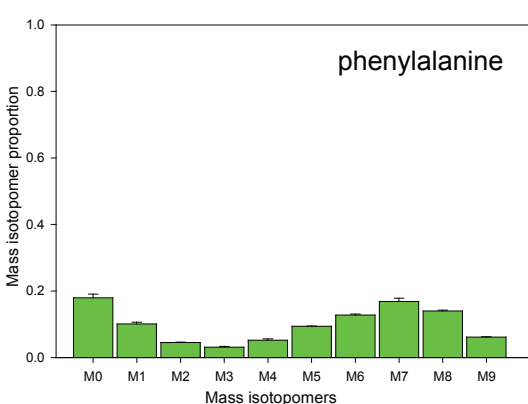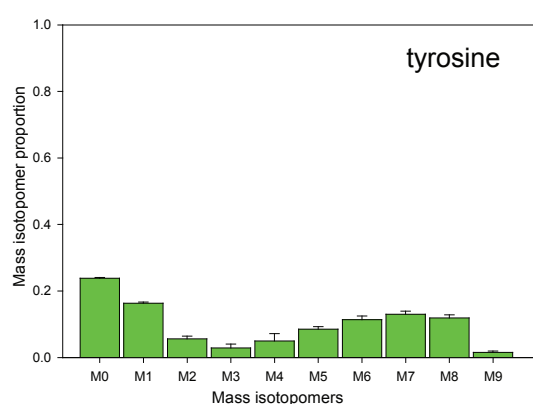

Supplement: Figure S3 — Mass isotopomers distribution of central metabolites measured by LC-MS of M. extorquens AM1 at 90 minutes after succinate exhaustion (Sampling time 3 of Fig. 1 ). (PDF) [file pone.0048271.s003.pdf]

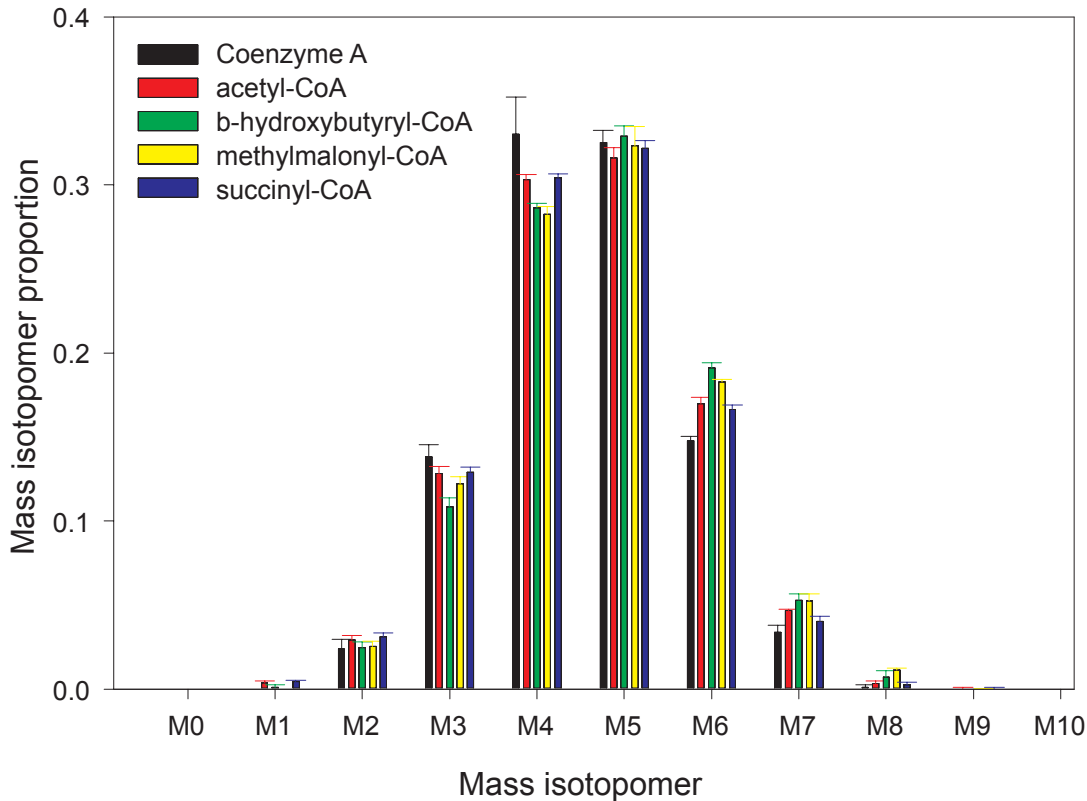

Supplement: Figure S4 — Mass isotopomers distribution in CoA thioesters measured by LC-MS during growth of M.extorquens AM1 upon co-consumption with 13C (>99%) methanol and natural abundance (1.1% 13C) succinate. Metabolite quenching, extraction and measurements were performed as described in material and methods. Mass isotopomer data correspond to sample collected during mid-co-consumption phase (Sampling time 1, see Fig. 1). (PDF) [file pone.0048271.s004.pdf]

# Coenzyme A biosynthesis

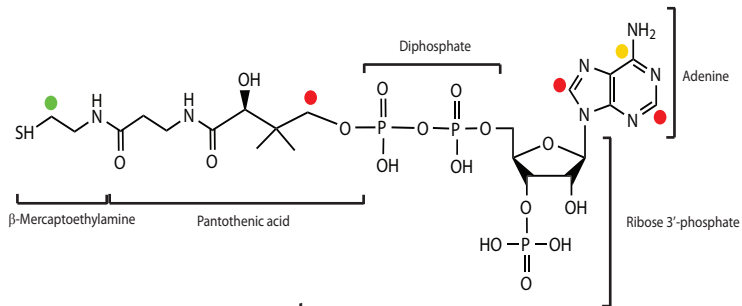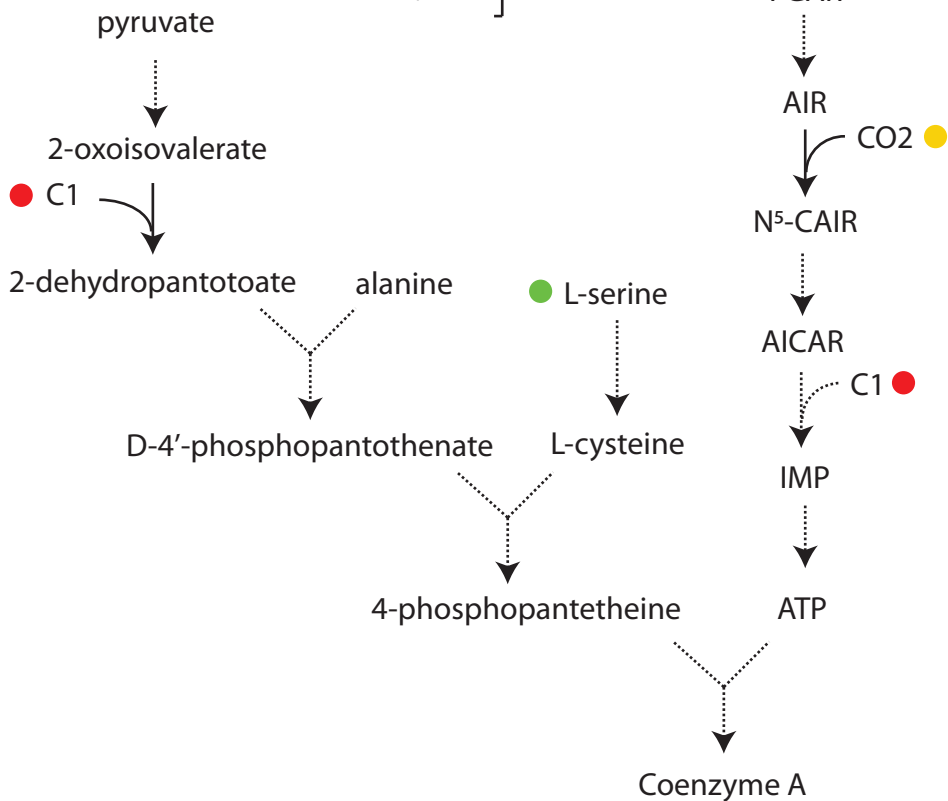

Supplement: Figure S5 — Scheme of the Coenzyme A biosynthesis in M. extorquens AM1. Identified 13C carbon entry points are indicated by colored cycles. Red, C1-precursor from tetrahydrofolate pathway; yellow, CO2; green, C3 carbon of serine. (PDF) [file pone.0048271.s005.pdf]
