# Supplementary material for: Co-Consumption of Methanol and Succinate by Methylobacterium extorquens AM1
Source: PLoS One. 2012 Nov 1;7(11):e48271. doi: 10.1371/journal.pone.0048271 (PMC3486813; doi:10.1371/journal.pone.0048271)
Supplement: Table S1 — Lists of parameters used for flux balance analysis and flux variability analysis. (PDF) [file pone.0048271.s006.pdf]

Simulation information

**biomass composition data**	methanol growth; Peyraud et al. (2011) BMC system biology
**Genome scale network**	iRP911; Peyraud et al. (2011) BMC system biology
**reduced network**	identified methylotrophyc network from Peyraud et al. (2011) BMC system biology modified as display in the SI Table S2.
	additional reduction: R-0015 was set irreversible and R-0245 was fixed to 0 in accordance with the 13C-labelling experiment where C1-units are generated from methanol; R-0991 was set to 0 : NADPH electron sink.

Constraints

		experimental data (mmol.g-1.h-1)				feasible solution under the constraints		
	reaction Identifier	value	std	min	max	value	free value	Δ experimetanl value
succinate uptake	EX-0009	3.13	0.48	2.65	3.60	2.67		0.46
methanol uptake	EX-0001	4,94*	0,44*	4.50	5.38	5.20		0.26
CO2 production from methanol	R-0011 + R-0095	5.20				5.10		0.10
CO2 production (total)	EX-0004	7.70	2.10	5.60	9.80	8.64		0.94
growth rate	mue	0.18	0.01	0.17	0.19	0.18		fixed

* Data of the 13C-labelling experiment were used because only 2 replicates were available.

** Data of the 13C-labelling experiment; a 13C/12C CO2 output ratio of 1.44 was fixed by linking R-0011 plus R-0095 values, via A-13CCO2 in results file, corresponding to 59% of the CO2 ouput (EX-0004)
