# Supplementary material for: Co-Consumption of Methanol and Succinate by Methylobacterium extorquens AM1
Source: PLoS One. 2012 Nov 1;7(11):e48271. doi: 10.1371/journal.pone.0048271 (PMC3486813; doi:10.1371/journal.pone.0048271)
Supplement: Table S2 — List of the reactions reduced for flux balance analysis upon co-consumption condition. (PDF) [file pone.0048271.s007.pdf]

						CRITERIA FOR NETWORK REDUCTION					
				Methylotrophic network	Succinate utilisation network	Proteomic: differential expression methanol vs succinate	Transcriptomic: Differential expression methanol vs succinate	Differential Enzyme activity between methanol and succinate growth	Presence of alternate reaction or alternate pathway	Comment	Co-consumption (methaol + succiante) network
REACTION LIST						Threshold : 2	Threshold : 2	Threshold : 2			
Reaction Identifier	Name	Equation	Subsystem	0: excluded; 1: included	0: excluded; 1: included	-1: lower expressed; 0: same level; 1: higher express; -: not measured	-1: lower expression; 0: same expression; 1: higer expression; -: not measured	-1: lower activity; 0: same activity; 1: higer activity; -: not measured	1: presence of alternate; -: no alternate reported		0: excluded; 1: included
EX-0001	exchange flux : methanol	= methanol[e]	exchange with environment	1	0	-	-	-	-	methanol uptake	1
EX-0002	exchange flux : oxygen	= O2[e]	exchange with environment	1	1	-	-	-	-		1
EX-0003	exchange flux : nitrogen	= NH3[e]	exchange with environment	1	1	-	-	-	-		1
EX-0004	exchange flux : CO2	= CO2[e]	exchange with environment	1	1	-	-	-	-		1
EX-0007	exchange flux : formaldehyde	= formaldehyde[e]	exchange with environment	0	0	-	-	-	-		0
EX-0008	exchange flux : phosphate	= phosphate[e]	exchange with environment	1	1	-	-	-	-		1
EX-0009	exchange flux : succinate	= succinate[e]	exchange with environment	0	1	-	-	-	-	succiante uptake	1
EX-0010	exchange flux : fumarate	= fumarate[e]	exchange with environment	0	0	-	-	-	-		0
EX-0011	exchange flux : citrate	= citrate[e]	exchange with environment	0	0	-	-	-	-		0
EX-0012	exchange flux : glycolate	= glycolate[e]	exchange with environment	0	0	-	-	-	-		0
EX-0013	exchange flux : formate	= formate[e]	exchange with environment	0	0	-	-	-	-		0
EX-0014	exchange flux : oxalate	= oxalate[e]	exchange with environment	0	0	-	-	-	-		0
EX-0015	exchange flux : acetate	= acetate[e]	exchange with environment	0	0	-	-	-	-		0
EX-0016	exchange flux : D-gluconate	= D-gluconate[e]	exchange with environment	0	0	-	-	-	-		0
EX-0017	exchange flux : putrescine	= putrescine[e]	exchange with environment	0	0	-	-	-	-		0
EX-0019	exchange flux : 2-oxoglutarate	= 2-oxoglutarate[e]	exchange with environment	0	0	-	-	-	-		0
EX-0020	exchange flux : SO42-	= SO42-[e]	exchange with environment	1	1	-	-	-	-		1
EX-0021	exchange flux : Na+	= Na+[e]	exchange with environment	1	1	-	-	-	-		1
EX-0022	exchange flux : thiosulfate	= thiosulfate[e]	exchange with environment	0	0	-	-	-	-		0
EX-0023	exchange flux : nitrate	= NO3-[e]	exchange with environment	0	0	-	-	-	-		0
EX-0024	exchange flux : L-glutamate	= L-glutamate[e]	exchange with environment	0	0	-	-	-	-		0
EX-0025	exchange flux : L-aspartate	= L-aspartate[e]	exchange with environment	0	0	-	-	-	-		0
EX-0026	exchange flux : H2O	= H2O[e]	exchange with environment	1	1	-	-	-	-		1
EX-0027	exchange flux : ethanolamine	= ethanolamine[e]	exchange with environment	0	0	-	-	-	-		0
EX-0028	exchange flux : (S)-malate	= (S)-malate[e]	exchange with environment	0	0	-	-	-	-		0
EX-0029	exchange flux : pyruvate	= pyruvate[e]	exchange with environment	0	0	-	-	-	-		0
EX-0030	exchange flux : D-glucose	= D-glucose[e]	exchange with environment	0	0	-	-	-	-		0
EX-0031	exchange flux : D-ribose	= D-ribose[e]	exchange with environment	0	0	-	-	-	-		0
EX-0032	exchange flux : 5-dehydro-D-gluconate	= 5-dehydro-D-gluconate[e]	exchange with environment	0	0	-	-	-	-		0
EX-0033	exchange flux : Co2+	= Co2+[e]	exchange with environment	1	1	-	-	-	-		1
EX-0034	Biomass flux : L-rhamnose	dTDP-a-L-rhamnose + H2O = L-rhamnose + dTDP	biomass and maintenance equation	1	1	-	-	-	-		1
EX-0035	Biomass flux : N-acetyl-D-glucosamine	UDP-N-acetyl-D-glucosamine + H2O = N-acetyl-D-glucosamine + U	biomass and maintenance equation	1	1	-	-	-	-		1
EX-0036	exchange flux : proton	= H+[e]	exchange with environment	1	1	-	-	-	-		1
EX-0037	exchange flux : H2O2	= H2O2[e]	exchange with environment	0	0	-	-	-	-		0
EX-0038	exchange flux : Fe2+	= Fe2+[e]	exchange with environment	1	1	-	-	-	-		1
EX-0039	exchange flux : molybdate	= molybdate[e]	exchange with environment	1	1	-	-	-	-		1
EX-0040	exchange flux : potassium	= K+[e]	exchange with environment	1	1	-	-	-	-		1
EX-0041	exchange flux : magnesium	= Mg2+[e]	exchange with environment	1	1	-	-	-	-		1
EX-0042	exchange flux : calcium	= Ca2+[e]	exchange with environment	1	1	-	-	-	-		1
EX-0043	exchange flux : copper	= Cu2+[e]	exchange with environment	1	1	-	-	-	-		1
EX-0044	exchange flux : manganese	= Mn2+[e]	exchange with environment	1	1	-	-	-	-		1
EX-0045	exchange flux : zinc	= Zn2+[e]	exchange with environment	1	1	-	-	-	-		1
EX-0046	exchange flux : chloride	= Cl-[e]	exchange with environment	1	1	-	-	-	-		1
EX-0047	exchange flux : light	= photon	exchange with environment	0	0	-	-	-	-		0
EX-0048	exchange flux : D-arabinose	= D-arabinose[e]	exchange with environment	0	0	-	-	-	-		0
EX-0049	exchange flux : L-arabinose	= L-arabinose[e]	exchange with environment	0	0	-	-	-	-		0
EX-0050	exchange flux : D-arabinonate	= D-arabinonate[e]	exchange with environment	0	0	-	-	-	-		0
EX-0051	exchange flux : L-arabinonate	= L-arabinonate[e]	exchange with environment	0	0	-	-	-	-		0
EX-0052	exchange flux : L-lyxose	= L-lyxose[e]	exchange with environment	0	0	-	-	-	-		0
EX-0053	exchange flux : D-xylose	= D-xylose[e]	exchange with environment	0	0	-	-	-	-		0
EX-0054	exchange flux : D-xylonate	= D-xylonate[e]	exchange with environment	0	0	-	-	-	-		0
EX-0055	exchange flux : L-lyxonate	= L-lyxonate[e]	exchange with environment	0	0	-	-	-	-		0
EX-0056	exchange flux : D-ribonate	= D-ribonate[e]	exchange with environment	0	0	-	-	-	-		0
EX-0057	exchange flux : 2-deoxy-D-ribonate	= 2-deoxy-D-ribonate[e]	exchange with environment	0	0	-	-	-	-		0
EX-0058	exchange flux : acetoacetate	= acetoacetate[e]	exchange with environment	0	0	-	-	-	-		0
EX-0059	exchange flux : propanoate	= propanoate[e]	exchange with environment	0	0	-	-	-	-		0
EX-0060	exchange flux : 3-O-b-D-galactopyranosyl-D-arabinos	= 3-O-b-D-galactopyranosyl-D-arabinose[e]	exchange with environment	0	0	-	-	-	-		0
EX-0061	exchange flux : 3-O-b-D-galactopyranosyl-D-arabinor	= 3-O-b-D-galactopyranosyl-D-arabinonate[e]	exchange with environment	0	0	-	-	-	-		0
EX-0062	exchange flux : decanoate	= decanoate[e]	exchange with environment	0	0	-	-	-	-		0
EX-0063	exchange flux : 2-deoxy-D-ribose	= 2-deoxy-D-ribose[e]	exchange with environment	0	0	-	-	-	-		0
EX-0064	exchange flux : methylamine	= methylamine[e]	exchange with environment	0	0	-	-	-	-		0
EX-0065	exchange flux : ethylamine	= ethylamine[e]	exchange with environment	0	0	-	-	-	-		0
EX-0066	exchange flux : ethanol	= ethanol[e]	exchange with environment	0	0	-	-	-	-		0
EX-0067	exchange flux : L-tartrate	= L-tartrate[e]	exchange with environment	0	0	-	-	-	-		0
EX-0068	exchange flux : glycerone	= glycerone[e]	exchange with environment	0	0	-	-	-	-		0
R-0001	methanol dehydrogenase	methanol[p] + 2 oxidized-cytochrome-c = formaldehyde[p] + 2 H+	methanol oxidation to formaldehyd	1	1	0	0	1	-		1
R-0002	H4F-dependent formaldehyde activating	formaldehyde + tetrahydrofolate = 5,10-methylenetetrahydrofola	tetrahydrofolate-independ pathway	0	0	-	-	-	1		0
R-0003	H4MPT-dependent formaldehyde activating	formaldehyde + tetrahydromethanopterin = 5,10-methylenetetra	tetrahydromethanopterin-depend p	1	1	0	1	1	-		1
R-0004	methylenetetrahydromethanopterin dehydrogenase	5,10-methylenetetrahydromethanopterin + NADP+ = 5,10-methe	tetrahydromethanopterin-depend p	1	1	1	0	0	-		1
R-0005	methylenetetrahydromethanopterin dehydrogenase	5,10-methylenetetrahydromethanopterin + NAD+ = 5,10-methen	tetrahydromethanopterin-depend p	1	1	0	0	0	-		1
R-0006	methenyltetrahydrometanopterin cyclohydrolase	5,10-methenyltetrahydromethanopterin + H2O = 5-formyltetrahy	tetrahydromethanopterin-depend p	1	1	0	0	0	-		1
R-0007	formylmethanofuran:H4MPT formyltransferase	methanofuran + 5-formyltetrahydromethanopterin = tetrahydror	tetrahydromethanopterin-depend p	1	1	0	0	0	-		1
R-0008	formyl-MFS hydrolysis	formylmethanofuran + H2O = formate + methanofuran	tetrahydromethanopterin-depend p	1	1	0	0	-	-		1
R-0011	formate dehydrogenase	formate + NAD+ = CO2 + NADH + H+	formate oxidation to CO2	1	1	1	0	1	-		1
R-0012	formate-tetrahydrofolate ligase	formate + ATP + tetrahydrofolate = 10-formyltetrahydrofolate + A	tetrahydrofolate-depend pathway	1	1	1	1	0	-		1
R-0013	methenyltetrahydrofolate cyclohydrolase	10-formyltetrahydrofolate + H+ = 5,10-methenyltetrahydrofolate	tetrahydrofolate-depend pathway	1	1	1	1	0	-		1
R-0014	methylenetetrahydrofolate dehydrogenase	5,10-methenyltetrahydrofolate + NADPH = 5,10-methylenetetrah	tetrahydrofolate-depend pathway	1	1	1	1	0	-		1
R-0015	serine hydroxymethyltransferase	5,10-methylenetetrahydrofolate + glycine + H2O = tetrahydrofola	serine cycle	1	1	0	0	1	-		1
R-0016	L-serine-glyoxylate aminotransferase	L-serine + glyoxylate = hydroxypyruvate + glycine	serine cycle	1	1	1	0	1	-		1
R-0017	hydroxypyruvate reductase	hydroxypyruvate + NADH + H+ = D-glycerate + NAD+	serine cycle	1	1	1	1	1	1		1
R-0018	glycerate kinase	D-glycerate + ATP = 2-phospho-D-glycerate + ADP	serine cycle	1	1	1	1	1	-		1
R-0019	enolase	2-phospho-D-glycerate = phosphoenolpyruvate + H2O	serine cycle	1	1	0	0	0	-		1
R-0020	phosphoenolpyruvate carboxylase	phosphoenolpyruvate + CO2 + H2O = oxaloacetate + phosphate	serine cycle	1	1	1	0	1	-		1
R-0021	malate dehydrogenase	(S)-malate + NAD+ = oxaloacetate + NADH + H+	serine cycle	1	1	0	0	-1	1		1
R-0022	malyl-CoA synthetase	(S)-malate + CoA + ATP = (S)-malyl-CoA + ADP + phosphate	serine cycle	1	1	1	1	1	-		1
R-0023	malyl-CoA lyase	(S)-malyl-CoA = acetyl-CoA + glyoxylate	serine cycle	1	1	1	0	1	-		1
R-0024	acetyl-CoA C-acetyltransferase	2 acetyl-CoA = CoA + acetoacetyl-CoA	Ethylmalonyl-CoA pathway	1	1	0	0	-1	-		1
R-0025	acetoacetyl-CoA reductase 1	acetoacetyl-CoA + NADPH + H+ = (R)-3-hydroxybutanoyl-CoA + N/	Ethylmalonyl-CoA pathway	1	1	0	0	0	1		1
R-0026	poly(3-hydroxyalkanoate) depolymerase	poly-beta-hydroxybutyrate(n) + H2O = poly-beta-hydroxybutyrat	PHB degradation	0	0	0	0	-	-		0
R-0027	poly(3-hydroxyalkanoate) polymerase	(R)-3-hydroxybutanoyl-CoA + poly-beta-hydroxybutyrate(n-1) = p	PHB biosynthesis	1	1	0	0	-	-		1
R-0028	D-beta-hydroxybutyrate dehydrogenase	(R)-3-hydroxybutanoate + NAD+ = acetoacetate + NADH + H+	PHB degradation	0	0	0	-1	-	-		0
R-0029	succinyl-CoA:3-oxo-acid CoA-transferase	succinyl-CoA + acetoacetate = succinate + acetoacetyl-CoA	PHB degradation	0	0	1	1	-	1		0
R-0031	3-hydroxybutyryl-CoA dehydratase	(S)-3-hydroxybutanoyl-CoA = crotonyl-CoA + H2O	fatty acid b-oxidation	0	0	0	0	-	1		0
R-0032	crotonyl-CoA reductase/carboxylase	crotonyl-CoA + NADPH + H+ + CO2 = (2S)-ethylmalonyl-CoA + NAD	Ethylmalonyl-CoA pathway	1	1	0	0	1	1		1
R-0033	crotonyl-CoA reductase	crotonyl-CoA + NADPH + H+ = butyryl-CoA + NADP+	Ethylmalonyl-CoA pathway	0	0	0	0	-	1		0
R-0034	butyryl-CoA carboxylase	ATP + butyryl-CoA + HCO3- + H+ = ADP + phosphate + (2S)-ethylm	Ethylmalonyl-CoA pathway	0	0	0	0	-	1		0
R-0035	ethylmalonyl-CoA mutase	(2R)-ethylmalonyl-CoA = (2S)-methylsuccinyl-CoA	Ethylmalonyl-CoA pathway	1	1	0	0	1	-		1
R-0036	mesaconyl-CoA hydratase	mesaconyl-CoA + H2O = erythro-b-methylmalyl-CoA	Ethylmalonyl-CoA pathway	1	1	0	0	1	-		1
R-0037	methylsuccinyl-CoA dehydrogenase	(2S)-methylsuccinyl-CoA + oxidized-ETF = mesaconyl-CoA + reduc	Ethylmalonyl-CoA pathway	1	1	0	0	1	-		1
R-0038	b-methylmalyl-CoA lyase	erythro-b-methylmalyl-CoA = propionyl-CoA + glyoxylate	Ethylmalonyl-CoA pathway	1	1	1	1	1	-		1
R-0044	propionyl-CoA carboxylase 1	ATP + propionyl-CoA + HCO3- + H+ = ADP + phosphate + (S)-methy	Ethylmalonyl-CoA pathway	1	1	0	0	0	-		1
R-0045	methylmalonyl-CoA epimerase	(S)-methylmalonyl-CoA = (R)-methylmalonyl-CoA	Ethylmalonyl-CoA pathway	1	1	0	0	-	-		1
R-0046	methylmalonyl-CoA mutase	(R)-methylmalonyl-CoA = succinyl-CoA	Ethylmalonyl-CoA pathway	1	1	0	0	1	-		1
R-0047	citrate synthase	acetyl-CoA + H2O + oxaloacetate = citrate + CoA	TCA cycle	1	1	0	-1	0	-		1
R-0048	aconitate hydratase	citrate = threo-D-isocitrate	TCA cycle	1	1	0	0	-	-		1
R-0049	isocitrate dehydrogenase	threo-D-isocitrate + NADP+ = 2-oxoglutarate + CO2 + NADPH + H+	TCA cycle	1	1	-1	-1	0	-		1
R-0050	2-oxoglutarate dehydrogenase	2-oxoglutarate + CoA + NAD+ = succinyl-CoA + CO2 + NADH + H+	TCA cycle	1	1	-1	0	-1	-		1
R-0051	succinyl-CoA hydrolase	succinyl-CoA + H2O = CoA + succinate	TCA cycle	1	1	-	-	-1	1		1
R-0052	succinate dehydrogenase	succinate + UQ = fumarate + UQH2	TCA cycle	1	1	-1	-1	0	-		1
R-0053	fumarate hydratase	fumarate + H2O = (S)-malate	TCA cycle	1	1	-1	0	-	-		1
R-0054	malate dehydrogenase (decarboxylating)	(S)-malate + NAD+ = pyruvate + CO2 + NADH + H+	pyruvate metabolism	1	1	-1	-1	0	1		1
R-0055	pyruvate dehydrogenase	pyruvate + CoA + NAD+ = acetyl-CoA + CO2 + NADH + H+	pyruvate metabolism	1	1	-1	-1	-1	1		1
R-0056	oxaloacetate decarboxylase	oxaloacetate = pyruvate + CO2	pyruvate metabolism	1	1	0	0	-	1		1
R-0057	phosphoenolpyruvate carboxykinase	ATP + oxaloacetate = ADP + phosphoenolpyruvate + CO2	gluconeogenesis	1	1	-1	0	-	-		1
R-0058	phosphoglyceromutase	2-phospho-D-glycerate = 3-phospho-D-glycerate	gluconeogenesis	1	1	-1	0	-	-		1
R-0059	phosphoglycerate kinase	ATP + 3-phospho-D-glycerate = ADP + 3-phospho-D-glyceroylphos	gluconeogenesis	1	1	0	0	-	-		1
R-0060	glyceraldehyde-3-phosphate dehydrogenase	D-glyceraldehyde-3-phosphate + phosphate + NAD+ = 3-phospho	gluconeogenesis	1	1	0	0	-	-		1
R-0061	triosephosphate isomerase	D-glyceraldehyde-3-phosphate = glycerone-phosphate	gluconeogenesis	1	1	0	0	-	-		1
R-0062	fructose-bisphosphate aldolase	glycerone-phosphate + D-glyceraldehyde-3-phosphate = D-fructo	gluconeogenesis	1	1	0	0	-	-		1
R-0063	fructose-1,6-bisphosphatase	D-fructose-1,6-bisphosphate + H2O = D-fructose-6-phosphate + p	gluconeogenesis	1	1	0	0	-	-		1
R-0064	phosphoglucose isomerase	D-fructose-6-phosphate = D-glucose-6-phosphate	gluconeogenesis	1	1	0	0	-	-		1
R-0065	glucokinase	ATP + D-glucose = ADP + D-glucose-6-phosphate	starch and sucrose metabolism	0	0	0	0	-	-		0
R-0066	glucose-6-phosphate 1-dehydrogenase	D-glucose-6-phosphate + NADP+ = D-glucono-1,5-lactone-6-phos	pentose phosphate pathway	1	1	0	0	-	-		1
R-0067	6-phosphogluconolactonase	D-glucono-1,5-lactone-6-phosphate + H2O = 6-phospho-D-glucon	pentose phosphate pathway	1	1	0	0	-	-		1
R-0068	6-phosphogluconate dehydrogenase	6-phospho-D-gluconate + NADP+ = D-ribulose-5-phosphate + CO2	pentose phosphate pathway	1	1	0	0	-	-		1
R-0069	D-ribulose-5-phosphate 3-epimerase	D-ribulose-5-phosphate = D-xylulose-5-phosphate	pentose phosphate pathway	1	1	-1	0	-	-		1
R-0070	ribose-5-phosphate isomerase	D-ribose-5-phosphate = D-ribulose-5-phosphate	pentose phosphate pathway	1	1	0	0	-	-		1
R-0071	D-xylulose 5-phosphate phosphoketolase	D-xylulose-5-phosphate + phosphate = acetylphosphate + D-glyce	pentose phosphate pathway	1	1	0	0	-	-		1
R-0072	D-fructose 6-phosphate phosphoketolase	D-fructose-6-phosphate + phosphate = acetylphosphate + D-eryth	pentose phosphate pathway	1	1	0	0	-	-		1
R-0073	transaldolase	sedoheptulose-7-phosphate + D-glyceraldehyde-3-phosphate = D	pentose phosphate pathway	1	1	0	0	-	-		1
R-0074	transketolase	sedoheptulose-7-phosphate + D-glyceraldehyde-3-phosphate = D	pentose phosphate pathway	1	1	0	0	-	-		1
R-0075	phosphoribosylpyrophosphate synthetase	ATP + D-ribose-5-phosphate = AMP + 5-phosphoribosyl-1-pyroph	pentose phosphate pathway	1	1	0	0	-	-		1
R-0076	phosphopentomutase	alpha-D-ribose-1-phosphate = D-ribose-5-phosphate	salvage pathways of purine and pyri	0	0	0	0	-	-		0
R-0077	1-deoxy-D-xylulose-5-phosphate synthase	pyruvate + D-glyceraldehyde-3-phosphate = 1-deoxy-D-xylulose-5	carotenoids biosynthesis	1	1	0	0	-	-		1
R-0078	2-deoxyribose-5-phosphate aldolase	2-deoxy-D-ribose-5-phosphate = D-glyceraldehyde-3-phosphate +	deoxyribonucleosides degradation	1	1	-	-	-	-		1
R-0079	6-phosphogluconate dehydratase	6-phospho-D-gluconate = 2-dehydro-3-deoxy-D-gluconate-6-phos	entner-Doudoroff pathway	1	1	-1	0	-	-		1
R-0080	2-keto-3-deoxygluconate 6-phosphate aldolase	2-dehydro-3-deoxy-D-gluconate-6-phosphate = pyruvate + D-glyc	entner-Doudoroff pathway	1	1	0	0	-	-		1
R-0081	2-keto-4-hydroxyglutarate aldolase	4-hydroxy-2-oxoglutarate = pyruvate + glyoxylate	glyoxylate and dicarboxylate metab	0	0	0	0	-	-		0
R-0082	bisphosphoglycerate mutase	3-phospho-D-glyceroylphosphate = 2,3-bisphospho-D-glycerate	gluconeogenesis	1	1	-1	0	-	-		1
R-0083	cob(I)alamin adenosyltransferase	ATP + cobinamide = triphosphate + adenosylcobinamide	adenosylcobalamin salvage pathwa	0	0	0	0	-	-		0
R-0086	glyoxylate reductase	glyoxylate + NADH + H+ = glycolate + NAD+	glyoxylate and dicarboxylate metab	1	1	0	0	1	-		1
R-0089	succinyl-CoA synthetase	ATP + succinate + CoA = ADP + phosphate + succinyl-CoA	TCA cycle	0	0	0	0	-	1		0
R-0090	malate dehydrogenase, FAD/NAD(P)-binding domair	(S)-malate + UQ = oxaloacetate + UQH2	TCA cycle	0	1	-1	-1	-	1	set usable upon succiante growth	1
R-0094	pyruvate phosphate dikinase	ATP + pyruvate + phosphate = AMP + phosphoenolpyruvate + dip	pyruvate metabolism	1	1	0	0	-	-		1
R-0095	formate dehydrogenase (cytochrome-linked) peripla	formate[p] + 2 oxidized-cytochrome-c = CO2[p] + 2 reduced-cytoc	formate oxidation to CO2	1	1	0	0	-	1		1
R-0097	(R)-3-hydroxybutyryl-CoA dehydratase	(R)-3-hydroxybutanoyl-CoA = crotonyl-CoA + H2O	Ethylmalonyl-CoA pathway	1	1	-	-	0	1		1
R-0098	ethylmalonyl-CoA epimerase	(2S)-ethylmalonyl-CoA = (2R)-ethylmalonyl-CoA	Ethylmalonyl-CoA pathway	1	1	0	0	-	-		1
R-0099	pyruvate kinase	ATP + pyruvate = ADP + phosphoenolpyruvate	pyruvate metabolism	1	1	0	0	-	-		1
R-0101	acetyl-CoA synthetase	ATP + acetate + CoA = AMP + diphosphate + acetyl-CoA	acetate metabolism	1	1	0	0	-	1		1
R-0102	S-lactoylglutathione hydrolase	S-lactoylglutathione + H2O = glutathione + D-lactate	methylglyoxal degradation	0	0	0	0	-	-		0
R-0105	pyruvate dehydrogenase/oxidase	pyruvate + H2O + 2 oxidized-cytochrome-c = acetate + CO2 + 2 rec	pyruvate metabolism	1	1	0	0	-	1		1
R-0106	phosphate acetyltransferase 1	acetyl-CoA + phosphate = CoA + acetylphosphate	acetate metabolism	0	0	0	0	-	1		0
R-0107	acetate kinase	acetate + ATP = acetylphosphate + ADP	acetate metabolism	0	0	1	0	-	1		0
R-0108	propionate kinase	propanoate + ATP = ADP + propionylphosphate	propanoate metabolism	0	0	0	0	-	-		0
R-0109	lactaldehyde dehydrogenase	(S)-lactaldehyde + NAD+ + H2O = (S)-lactate + NADH + 2 H+	L-lactaldehyde degradation	0	0	0	1	-	-		0
R-0110	glycolaldehyde dehydrogenase	glycolaldehyde + NAD+ + H2O = glycolate + NADH + H+	glyoxylate and dicarboxylate metab	1	1	0	1	-	-		1
R-0111	lactoylglutathione lyase	S-lactoylglutathione = glutathione + methylglyoxal	methylglyoxal degradation	0	0	0	0	-	-		0
R-0112	phosphopentomutase 2	deoxyribose-1-phosphate = 2-deoxy-D-ribose-5-phosphate	deoxyribonucleosides degradation	1	1	0	0	-	-		1
R-0113	amino acid transferase (leucine)	4-methyl-2-oxopentanoate + L-glutamate = L-leucine + 2-oxoglut	valine, leucine, isoleucine biosynthe	1	1	0	0	-	-		1
R-0114	amino acid transferase (valine)	3-methyl-2-oxobutanoate + L-glutamate = L-valine + 2-oxoglutara	valine, leucine, isoleucine biosynthe	1	1	0	0	-	-		1
R-0115	amino acid transferase (isoleucine)	2-keto-3-methyl-valerate + L-glutamate = L-isoleucine + 2-oxoglut	valine, leucine, isoleucine biosynthe	1	1	0	0	-	-		1
R-0116	valine tRNA synthetase	ATP + L-valine + tRNA(Val) = AMP + diphosphate + L-valyl-tRNA(V	valine, leucine, isoleucine biosynthe	1	1	0	0	-	-		1
R-0117	leucine tRNA synthetase	ATP + L-leucine + tRNA(Leu) = AMP + diphosphate + L-leucyl-tRNA	valine, leucine, isoleucine biosynthe	1	1	0	0	-	-		1
R-0118	isoleucine tRNA synthetase	ATP + L-isoleucine + tRNA(Ile) = AMP + diphosphate + L-isoleucyl-	valine, leucine, isoleucine biosynthe	1	1	0	0	-	-		1
R-0119	2-isopropylmalate synthase	acetyl-CoA + 3-methyl-2-oxobutanoate + H2O = (2S)-2-isopropylm	valine, leucine, isoleucine biosynthe	1	1	0	0	-	-		1
R-0120	3-isopropylmalate dehydrogenase	3-isopropylmalate + NAD+ = 2-isopropyl-3-oxosuccinate + NADH	valine, leucine, isoleucine biosynthe	1	1	-	-	-	-		1
R-0121	2-isopropyl-3-oxosuccinate decarboxylase	2-isopropyl-3-oxosuccinate = 4-methyl-2-oxopentanoate + CO2	valine, leucine, isoleucine biosynthe	1	1	-	-	-	-		1
R-0122	2,3-dihydroxy-isovalerate dehydratase	2,3-dihydroxy-isovalerate = 3-methyl-2-oxobutanoate + H2O	valine, leucine, isoleucine biosynthe	1	1	0	0	-	-		1
R-0123	2,3-dihydroxy-3-methylvalerate dehydratase	2,3-dihydroxy-3-methylvalerate = 2-keto-3-methyl-valerate + H2C	valine, leucine, isoleucine biosynthe	1	1	0	0	-	-		1
R-0124	acetolactate synthase	2 pyruvate = 2-acetolactate + CO2	valine, leucine, isoleucine biosynthe	1	1	0	0	-	-		1
R-0125	2-aceto-2-hydroxy-butyrate synthase	pyruvate + 2-oxobutanoate + H+ = 2-aceto-2-hydroxy-butyrate + (	valine, leucine, isoleucine biosynthe	1	1	0	0	-	-		1
R-0126	2-acetolactate reductoisomerase	2-acetolactate + NADPH + H+ = 2,3-dihydroxy-isovalerate + NADP	valine, leucine, isoleucine biosynthe	1	1	0	0	-	-		1
R-0127	2-aceto-2-hydroxy-butyrate reductoisomerase	2-aceto-2-hydroxy-butyrate + NADPH + H+ = 2,3-dihydroxy-3-met	valine, leucine, isoleucine biosynthe	1	1	0	0	-	-		1
R-0128	3-isopropylmalate isomerase	isopropylmaleate + H2O = 3-isopropylmalate	valine, leucine, isoleucine biosynthe	1	1	0	0	-	-		1
R-0129	2-isopropylmalate isomerase	isopropylmaleate + H2O = (2S)-2-isopropylmalate	valine, leucine, isoleucine biosynthe	1	1	0	0	-	-		1
R-0130	phosphoglucomutase	D-glucose-1-phosphate = D-glucose-6-phosphate	sugar nucleotide biosynthesis	1	1	0	0	-	-		1
R-0131	phosphomannomutase	D-mannose-1-phosphate = D-mannose-6-phosphate	sugar nucleotide biosynthesis	1	1	0	0	-	-		1
R-0132	beta phosphoglucomutase	b-D-glucose-1-phosphate = b-D-glucose-6-phosphate	sugar metabolism	1	1	-	0	-	-		1
R-0133	glucose-1-phosphate uridylyltransferase	UTP + D-glucose-1-phosphate = diphosphate + UDP-glucose	sugar nucleotide biosynthesis	1	1	0	0	-	-		1
R-0134	UDP-glucose 4-epimerase	UDP-glucose = UDP-galactose	sugar nucleotide biosynthesis	1	1	0	0	-	-		1
R-0135	UDP-glucose-6-dehydrogenase	UDP-glucose + 2 NAD+ + H2O = UDP-glucuronate + 2 NADH + 2 H+	sugar nucleotide biosynthesis	1	1	0	0	-	-		1
R-0136	GDP-L-fucose synthase	GDP-4-dehydro-6-deoxy-D-mannose + NADPH + H+ = GDP-fucose	sugar nucleotide biosynthesis	1	1	0	0	-	-		1
R-0137	mannose-1-phosphate guanylyltransferase	GTP + D-mannose-1-phosphate = GDP-mannose + diphosphate	sugar nucleotide biosynthesis	1	1	0	0	-	-		1
R-0138	mannose-6-phosphate isomerase	D-fructose-6-phosphate = D-mannose-6-phosphate	sugar nucleotide biosynthesis	1	1	0	0	-	-		1
R-0139	GDP-mannose 3,5-epimerase 1	GDP-mannose = GDP-galactose	sugar nucleotide biosynthesis	1	1	0	0	-	1		1
R-0140	GDP-mannose 3,5-epimerase 2	GDP-mannose = GDP-gulose	sugar nucleotide biosynthesis	1	1	0	0	-	1		1
R-0141	GDP-mannose 3,5-epimerase 3	GDP-gulose = GDP-galactose	sugar nucleotide biosynthesis	0	0	0	0	-	1		0
R-0142	GDP-mannose 4,6-dehydratase	GDP-mannose = GDP-4-dehydro-6-deoxy-D-mannose + H2O	sugar nucleotide biosynthesis	1	1	-	-	-	-		1
R-0143	gluconate 2-dehydrogenase (periplasmic)	D-gluconate[p] + 2 oxidized-cytochrome-c = 2-dehydro-D-glucona	sugar oxidation	0	0	1	1	-	-		0
R-0145	gluconokinase	ATP + D-gluconate = ADP + 6-phospho-D-gluconate	sugar metabolism	0	0	-	-	-	-		0
R-0146	galactose mutarotase	D-galactose[p] = b-D-galactose[p]	sugar metabolism	0	0	-	0	-	-		0
R-0147	aldose sugar dehydrogenase	D-glucose[p] + 2 oxidized-cytochrome-c = glucono-d-lactone[p] +	sugar oxidation	0	0	0	0	-	-		0
R-0148	L-fuculose-1-phosphate aldolase	L-fuculose-1-phosphate = glycerone-phosphate + (S)-lactaldehyde	sugar degradation	0	0	0	0	-	-		0
R-0149	trehalose-6-phosphate synthase	UDP-glucose + D-glucose-6-phosphate = UDP + trehalose-6-phosp	starch and sucrose metabolism	1	1	0	0	-	-		1
R-0150	trehalose-6-phosphate phosphatase	trehalose-6-phosphate + H2O = trehalose + phosphate	starch and sucrose metabolism	1	1	0	0	-	-		1
R-0151	maltose alpha-D-glucosyltransferase	maltose = trehalose	starch and sucrose metabolism	1	1	0	0	-	-		1
R-0152	maltooligosyltrehalose trehalohydrolase	maltooligosyl-trehalose = maltodextrin + trehalose	starch and sucrose metabolism	0	0	0	0	-	-		0
R-0153	cellulose synthase	UDP-glucose + (1,4-beta-D-glucosyl)(n) = UDP + (1,4-beta-D-glucos	starch and sucrose metabolism	1	1	1	0	-	-		1
R-0154	glycogen synthase	ADP-glucose + (1,4-alpha-D-glucosyl)(n) = ADP + (1,4-alpha-D-gluc	starch and sucrose metabolism	1	1	0	0	-	-		1
R-0155	1,4-alpha-glucan branching enzyme	(1,4-alpha-D-glucosyl)(n+1) = glycogen	starch and sucrose metabolism	1	1	0	0	-	-		1
R-0156	4-a-glucanotransferase	limit-dextrin = limit-dextrin-with-short-branches	starch and sucrose metabolism	1	1	0	0	-	-		1
R-0157	amylo-a-1,6-glucosidase	limit-dextrin-with-short-branches + H2O = debranched-limit-dext	starch and sucrose metabolism	1	1	0	0	-	-		1

R-0158	glycogen phosphorylase	glycogen + phosphate = limit-dextrin + D-glucose-1-phosphate	starch and sucrose metabolism	1	1	-	-	-	-		1
R-0159	glucoamylase	debranched-limit-dextrin + n H2O = n b-D-glucose	starch and sucrose metabolism	1	1	0	0	-	-		1
R-0160	alpha-D-glucose-1-phosphate cytidylyltransferase	CTP + D-glucose-1-phosphate = diphosphate + CDP-glucose	sugar nucleotide biosynthesis	1	1	-	-	-	-		1
R-0161	cdp-glucose 4,6-dehydratase	CDP-glucose = CDP-4-dehydro-6-deoxy-D-glucose + H2O	sugar nucleotide biosynthesis	1	1	0	0	-	-		1
R-0162	CDP-4-dehydro-6-deoxy-D-gulose 4-reductase	CDP-4-dehydro-6-deoxy-D-glucose + NADPH + H+ = CDP-4-dehydr	sugar nucleotide biosynthesis	1	1	-	-	-	-		1
R-0163	dTDP-4-dehydrorhamnose 3,5-epimerase	dTDP-4-dehydro-6-deoxy-D-glucose = dTDP-4-dehydro-6-deoxy-L-	starch and sucrose metabolism	1	1	0	0	-	-		1
R-0164	dTDP-glucose 4,6-dehydratase	dTDP-glucose = dTDP-4-dehydro-6-deoxy-D-glucose + H2O	starch and sucrose metabolism	1	1	0	0	-	-		1
R-0165	dTDP-4-dehydrorhamnose reductase	dTDP-4-dehydro-6-deoxy-L-mannose + NADPH + H+ = dTDP-a-L-rh	starch and sucrose metabolism	1	1	0	0	-	-		1
R-0166	glucose-1-phosphate thymidylyltransferase	dTTP + D-glucose-1-phosphate = diphosphate + dTDP-glucose	starch and sucrose metabolism	1	1	0	0	-	-		1
R-0167	endo-1,4-D-glucanase	(1,4-alpha-D-glucosyl)(n+1) + H2O = (1,4-alpha-D-glucosyl)(x) + sh	starch and sucrose metabolism	1	1	0	0	-	-		1
R-0168	shikimate 5-dehydrogenase	shikimate + NADP+ = 3-dehydroshikimate + NADPH + H+	phenylalanine, tyrosine, tryptophan	1	1	0	0	-	-		1
R-0169	prephenate dehydratase	prephenate = phenylpyruvate + H2O + CO2	phenylalanine, tyrosine, tryptophan	1	1	0	0	-	-		1
R-0170	malonyl-CoA decarboxylase	malonyl-CoA = acetyl-CoA + CO2	malonate degradation	0	0	0	0	-	-		0
R-0171	acetyl-CoA carboxylase	ATP + acetyl-CoA + HCO3- + H+ = ADP + phosphate + malonyl-CoA	fatty acid biosynthesis	1	1	0	0	-	-		1
R-0174	propionyl-CoA synthetase	ATP + propanoate + CoA = AMP + diphosphate + propionyl-CoA	propanoate metabolism	0	0	0	0	-	-		0
R-0175	malonate decarboxylase	malonate = acetate + CO2	malonate degradation	0	0	0	0	-	-		0
R-0176	phosphate acetyltransferase 2	propionyl-CoA + phosphate = propionylphosphate + CoA	propanoate metabolism	0	0	0	0	-	-		0
R-0177	2-dehydro-3-deoxyphosphoheptonate aldolase	phosphoenolpyruvate + D-erythrose-4-phosphate + H2O = 3-deo	phenylalanine, tyrosine, tryptophan	1	1	0	0	-	-		1
R-0178	5-enolpyruvylshikimate-3-phosphate synthetase	phosphoenolpyruvate + shikimate-3-phosphate = phosphate + 5-	phenylalanine, tyrosine, tryptophan	1	1	0	0	-	-		1
R-0179	anthranilate phosphoribosyltransferase	anthranilate + 5-phosphoribosyl-1-pyrophosphate = N-(5-phosph	phenylalanine, tyrosine, tryptophan	1	1	0	0	-	-		1
R-0180	phosphoribosylanthranilate isomerase	N-(5-phospho-beta-D-ribosyl)-anthranilate = 1-(2-carboxyphenyla	phenylalanine, tyrosine, tryptophan	1	1	0	0	-	-		1
R-0181	anthranilate synthase	chorismate + L-glutamine = anthranilate + pyruvate + L-glutamate	phenylalanine, tyrosine, tryptophan	1	1	0	0	-	-		1
R-0182	tryptophan synthase	L-serine + indole-3-glycerol-phosphate = L-tryptophan + D-glycera	phenylalanine, tyrosine, tryptophan	0	0	0	0	-	1		0
R-0183	tryptophan synthase A	indole-3-glycerol-phosphate = indole + D-glyceraldehyde-3-phosp	phenylalanine, tyrosine, tryptophan	1	1	0	0	-	1		1
R-0184	tryptophan synthase B	indole + L-serine = L-tryptophan + H2O	phenylalanine, tyrosine, tryptophan	1	1	0	0	-	1		1
R-0185	chorismate mutase	chorismate = prephenate	phenylalanine, tyrosine, tryptophan	1	1	0	0	-	-		1
R-0186	aminodeoxychorismate synthase	chorismate + L-glutamine = 4-amino-4-deoxychorismate + L-gluta	phenylalanine, tyrosine, tryptophan	1	1	0	0	-	-		1
R-0187	tyrosine tRNA synthetase	ATP + L-tyrosine + tRNA(Tyr) = AMP + diphosphate + L-tyrosyl-tRN	phenylalanine, tyrosine, tryptophan	1	1	0	0	-	-		1
R-0188	phenylalanine tRNA synthetase	ATP + L-phenylalanine + tRNA(Phe) = AMP + diphosphate + L-phe	phenylalanine, tyrosine, tryptophan	1	1	0	0	-	-		1
R-0189	chorismate synthase	5-enolpyruvylshikimate-3-phosphate = chorismate + phosphate	phenylalanine, tyrosine, tryptophan	1	1	0	0	-	-		1
R-0190	3-dehydroquinate dehydratase	3-dehydroquinate = 3-dehydroshikimate + H2O	phenylalanine, tyrosine, tryptophan	1	1	1	0	-	-		1
R-0191	indole-3-glycerol phosphate synthase	1-(2-carboxyphenylamino)-1-deoxy-D-ribulose-5-phosphate = ind	phenylalanine, tyrosine, tryptophan	1	1	0	0	-	-		1
R-0192	cyclohexadienyl dehydrogenase	L-arogenate + NAD+ = L-tyrosine + CO2 + NADH + H+	phenylalanine, tyrosine, tryptophan	1	1	-	-	-	-		1
R-0193	histidinol-phosphate aminotransferase	imidazol-acetol-phosphate + L-glutamate = L-histidinol-phosphat	histidine biosynthesis	1	1	0	0	-	-		1
R-0194	shikimate kinase	shikimate + ATP = shikimate-3-phosphate + ADP	phenylalanine, tyrosine, tryptophan	1	1	0	0	-	-		1
R-0195	3-dehydroquinate synthase	3-deoxy-D-arabino-heptulosonate-7-phosphate = 3-dehydroquin	phenylalanine, tyrosine, tryptophan	1	1	0	0	-	-		1
R-0196	UDP-glucuronate 5'-epimerase	UDP-glucuronate = UDP-L-iduronate	sugar nucleotide biosynthesis	1	1	0	0	-	-		1
R-0197	homoserine O-succinyltransferase	succinyl-CoA + L-homoserine = CoA + O-succinyl-L-homoserine	methionine biosynthesis	0	0	-	0	-	1		0
R-0198	5'-methylthioadenosine phosphorylase	(S)-methyl-5'-thioadenosine + phosphate = adenine + (S)-methyl-	methionine salvage pathway	1	1	-1	0	-	-		1
R-0199	spermidine synthase	(S)-adenosylmethioninamine + putrescine = (S)-methyl-5'-thioade	polyamine biosynthesis	1	1	0	0	-	-		1
R-0200	S-adenosylhomocysteine hydrolase	(S)-adenosyl-L-homocysteine + H2O = L-homocysteine + adenosin	methionine biosynthesis	1	1	0	0	-	-		1
R-0201	O-acetylhomoserine sulfhydrylase	O-acetyl-L-homoserine + H2S = L-homocysteine + acetate	methionine biosynthesis	1	1	0	0	-	1		1
R-0202	cystathionine gamma-synthase	O-succinyl-L-homoserine + L-cysteine = L-cystathionine + succinat	methionine biosynthesis	0	0	0	0	-	1		0
R-0203	cystathionine beta-lyase	L-cystathionine + H2O = L-homocysteine + NH3 + pyruvate	methionine biosynthesis	0	0	0	0	-	1		0
R-0204	methionyl-tRNA synthetase	ATP + L-methionine + tRNA(Met) = AMP + diphosphate + L-methi	methionine biosynthesis	1	1	0	0	-	-		1
R-0205	methionine adenosyltransferase	ATP + L-methionine + H2O = phosphate + diphosphate + (S)-aden	methionine biosynthesis	1	1	0	0	-	-		1
R-0206	homoserine O-acetyltransferase	acetyl-CoA + L-homoserine = CoA + O-acetyl-L-homoserine	methionine biosynthesis	1	1	0	0	-	1		1
R-0207	homocysteine transmethylase	5-methyltetrahydrofolate + L-homocysteine = tetrahydrofolate +	methionine biosynthesis	1	1	-1	0	-	-		1
R-0208	5,10-methylenetetrahydrofolate reductase	5,10-methylenetetrahydrofolate + NADPH + H+ = 5-methyltetrahy	methionine biosynthesis	1	1	0	0	-	-		1
R-0209	10-formyltetrahydrofolate:L-methionyl-tRNA(fMet) N	10-formyltetrahydrofolate + L-methionyl-tRNA(fMet) = tetrahydro	methionine biosynthesis	1	1	0	0	-	-		1
R-0210	2,3,4,5-tetrahydropyridine-2-carboxylate N-succinylt	succinyl-CoA + tetrahydrodipicolinate + H2O = CoA + N-succinyl-2	lysine biosynthesis	1	1	0	0	-	-		1
R-0211	N-succinyl-L-diaminopimelate desuccinylase	N-succinyl-L,L-2,6-diaminopimelate + H2O = LL-2,6-diaminohepta	lysine biosynthesis	1	1	0	0	-	-		1
R-0212	diaminopimelate decarboxylase	meso-diaminoheptanedioate = L-lysine + CO2	lysine biosynthesis	1	1	0	0	-	-		1
R-0213	diaminopimelate epimerase	LL-2,6-diaminoheptanedioate = meso-diaminoheptanedioate	lysine biosynthesis	1	1	0	0	-	-		1
R-0214	dihydrodipicolinate synthase	L-aspartate-4-semialdehyde + pyruvate = dihydrodipicolinate + 2	lysine biosynthesis	1	1	0	0	-	-		1
R-0215	lysyl tRNA synthetase	ATP + L-lysine + tRNA(Lys) = AMP + diphosphate + L-lysyl-tRNA(Lys	lysine biosynthesis	1	1	0	0	-	-		1
R-0216	dihydrodipicolinate reductase	dihydrodipicolinate + NADPH + H+ = tetrahydrodipicolinate + NA	lysine biosynthesis	1	1	0	0	-	-		1
R-0217	acetylornithine aminotransferase	N-acetyl-L-ornithine + 2-oxoglutarate = L-glutamate + N-acetyl-L-	arginine and proline metabolism	1	1	0	0	-	-		1
R-0218	succinylornithine transaminase	N2-succinyl-L-ornithine + 2-oxoglutarate = L-glutamate + N2-succi	arginine and proline degradation	0	0	0	0	-	-		0
R-0219	histidinal dehydrogenase	histidinal + NAD+ + H2O = L-histidine + NADH + H+	histidine biosynthesis	1	1	0	0	-	-		1
R-0220	histidinol dehydrogenase	L-histidinol + NAD+ = histidinal + NADH + H+	histidine biosynthesis	1	1	0	0	-	-		1
R-0221	imidazoleglycerol-phosphate dehydratase	D-erythro-imidazole-glycerol-phosphate = imidazol-acetol-phosp	histidine biosynthesis	1	1	0	0	-	-		1
R-0222	histidyl-tRNA synthetase	ATP + L-histidine + tRNA(His) = AMP + diphosphate + L-histidyl-tR	histidine biosynthesis	1	1	0	0	-	-		1
R-0223	imidazole glycerol phosphate synthase	phosphoribulosylformimino-AICAR-phosphate + L-glutamine = L-	histidine biosynthesis	1	1	0	0	-	-		1
R-0224	phosphoribosyl-ATP pyrophosphatase	phosphoribosyl-ATP + H2O = phosphoribosyl-AMP + diphosphate	histidine biosynthesis	1	1	0	0	-	-		1
R-0225	phosphoribosyl-AMP cyclohydrolase	phosphoribosyl-AMP + H2O = phosphoribosylformimino-AICAR-p	histidine biosynthesis	1	1	0	0	-	-		1
R-0226	N-(5'-phospho-L-ribosyl-formimino)-5-amino-1-(5'-pl	phosphoribosylformimino-AICAR-phosphate = phosphoribulosylf	histidine biosynthesis	1	1	0	0	-	-		1
R-0227	ATP phosphoribosyltransferase	ATP + 5-phosphoribosyl-1-pyrophosphate = phosphoribosyl-ATP -	histidine biosynthesis	1	1	0	0	-	-		1
R-0228	oxalyl-CoA decarboxylase	oxalyl-CoA = formyl-CoA + CO2	oxalate metabolism	0	0	0	0	-	-		0
R-0229	formyl-CoA transferase	formyl-CoA + oxalate = formate + oxalyl-CoA	oxalate metabolism	0	0	0	0	-	-		0
R-0230	hydroxypyruvate isomerase	hydroxypyruvate = tartronate-semialdehyde	tartronate-semialdehyde metabolis	0	0	0	0	-	1		0
R-0231	glyoxylate dehydrogenase	glyoxylate + CoA + NADP+ = oxalyl-CoA + NADPH + H+	oxalate metabolism	0	0	-	-	-	-		0
R-0232	phosphoglycolate phosphatase	2-phosphoglycolate + H2O = glycolate + phosphate	glyoxylate and dicarboxylate metab	0	1	-1	0	-	-	set usable upon succiante growth	1
R-0233	2-hydroxy-3-oxopropionate reductase (NAD)	D-glycerate + NAD+ = tartronate-semialdehyde + NADH + H+	tartronate-semialdehyde metabolis	0	0	0	0	-	1		0
R-0234	2-hydroxy-3-oxopropionate reductase (NADP)	D-glycerate + NADP+ = tartronate-semialdehyde + NADPH + H+	tartronate-semialdehyde metabolis	0	0	0	0	-	1		0
R-0235	formamide amidohydrolase	formamide + H2O = formate + NH3	histidine degradation	0	0	0	0	-	-		0
R-0236	5-aminolevulinate synthase	succinyl-CoA + glycine = 5-aminolevulinate + CoA + CO2	tetrapyrrole biosynthesis II	1	1	0	0	-	-		1
R-0237	porphobilinogen synthase	2 5-aminolevulinate = porphobilinogen + 2 H2O	tetrapyrrole biosynthesis II	1	1	0	0	-	-		1
R-0238	porphobilinogen deaminase	4 porphobilinogen + H2O = 4 NH3 + hydroxymethylbilane	tetrapyrrole biosynthesis II	1	1	0	0	-	-		1
R-0239	uroporphyrinogen III synthase	hydroxymethylbilane = uroporphyrinogen-III + H2O	tetrapyrrole biosynthesis II	1	1	0	0	-	-		1
R-0240	sarcosine oxidase	sarcosine + O2 + H2O = H2O2 + formaldehyde + glycine	sarcosine degradation	0	0	0	0	-	-		0
R-0241	aspartate-semialdehyde dehydrogenase	L-aspartyl-4-phosphate + NADPH + H+ = phosphate + L-aspartate-	L-threonine biosynthesis	1	1	0	0	-	-		1
R-0242	glycine amidinotransferase	glycine + L-arginine = guanidinoacetate + L-ornithine	creatinine biosynthesis	1	1	0	0	-	-		1
R-0243	glycine decarboxylase	H-Gcv-protein-(lipoyl)lysine + glycine = H-protein-S-(aminomethy	glycine, serine metabolism	1	1	-1	-1	-	-		1
R-0244	H-protein-lipoamide dehydrogenase	H-Gcv-protein-(dihydrolipoyl)lysine + NAD+ = H-Gcv-protein-(lipo	glycine, serine metabolism	1	1	-1	-1	-	-		1
R-0245	H-protein-aminomethyltransferase	H-protein-S-(aminomethyldihydrolipoyl)lysine + tetrahydrofolate	glycine, serine metabolism	1	1	-1	0	-	-		1
R-0249	threonine synthase	O-phospho-L-homoserine + H2O = L-threonine + phosphate	L-threonine biosynthesis	1	1	0	0	-	-		1
R-0251	phosphoserine phosphatase (D)	O-phospho-D-serine + H2O = D-serine + phosphate	glycine, serine metabolism	1	1	0	0	-	-		1
R-0252	L-threonine aldolase	L-threonine = glycine + acetaldehyde	glycine, serine, threonine degradati	0	0	0	0	-	-		0
R-0253	L-allo-threonine aldolase	L-allo-threonine = glycine + acetaldehyde	unassigned	1	1	0	0	-	-		1
R-0254	phenylserine aldolase	L-threo-3-phenylserine = benzaldehyde + glycine	unassigned	1	1	0	0	-	-		1
R-0255	threonine deaminase	L-threonine = 2-oxobutanoate + NH3 + H+	valine, leucine, isoleucine biosynthe	1	1	0	0	-	-		1
R-0256	L-serine deaminase	L-serine = pyruvate + NH3	glycine, serine, threonine degradati	0	0	0	0	-	-		0
R-0257	homoserine dehydrogenase	L-aspartate-4-semialdehyde + NADPH + H+ = L-homoserine + NAD	L-threonine biosynthesis	1	1	0	0	-	-		1
R-0258	threonine tRNA synthetase	ATP + L-threonine + tRNA(Thr) = AMP + diphosphate + L-threonyl-	L-threonine biosynthesis	1	1	-	-	-	-		1
R-0259	serine tRNA synthetase	ATP + L-serine + tRNA(Ser) = AMP + diphosphate + L-seryl-tRNA(Se	glycine, serine metabolism	1	1	0	0	-	-		1
R-0260	homoserine kinase	L-homoserine + ATP = O-phospho-L-homoserine + ADP	L-threonine biosynthesis	1	1	0	0	-	-		1
R-0261	aspartokinase	L-aspartate + ATP = L-aspartyl-4-phosphate + ADP	L-threonine biosynthesis	1	1	0	0	-	-		1
R-0262	glycine tRNA synthetase	ATP + glycine + tRNA(Gly) = AMP + diphosphate + glycyl-tRNA(Gly	glycine, serine metabolism	1	1	0	0	-	-		1
R-0263	D-3-phosphoglycerate dehydrogenase	3-phospho-D-glycerate + NAD+ = 3-phospho-hydroxypyruvate + N	glycine, serine metabolism	1	1	-1	0	-	-		1
R-0264	phosphoserine aminotransferase	L-glutamate + 3-phospho-hydroxypyruvate = 3-phospho-L-serine	glycine, serine metabolism	1	1	0	0	-	-		1
R-0265	phosphoserine phosphatase	3-phospho-L-serine + H2O = L-serine + phosphate	glycine, serine metabolism	1	1	0	0	-	-		1
R-0266	phosphohydroxythreonine aminotransferase	2-oxo-3-hydroxy-4-phosphobutanoate + L-glutamate = 4-(phosph	pyridoxal-5'-phosphate biosynthesis	1	1	0	0	-	-		1
R-0267	a-ketoglutarate reductase	2-hydroxyglutarate + NAD+ = 2-oxoglutarate + NADH + H+	unassigned	0	1	-1	-1	-	-	set usable upon succiante growth	1
R-0268	glycerol-3-phosphate dehydrogenase	glycerone-phosphate + NADPH + H+ = sn-glycerol-3-phosphate + I	glycerophospholipid metabolism	1	1	0	0	-	-		1
R-0270	ethanolamine ammonia-lyase	ethanolamine = acetaldehyde + NH3	ethanolamine utilization	0	0	0	0	-	-		0
R-0272	CDP-diacylglycerol synthetase (n-16:0)	CTP + 1,2-dihexadecanoyl-sn-glycerol-3-phosphate = diphosphate	glycerophospholipid metabolism	1	1	0	0	-	-		1
R-0273	CDP-diacylglycerol synthetase (n-16:1)	CTP + 1,2-dihexadec-9-enoyl-sn-glycerol-3-phosphate = diphosph	glycerophospholipid metabolism	1	1	0	0	-	-		1
R-0274	CDP-diacylglycerol synthetase (n-18:0)	CTP + 1,2-dioctadecanoyl-sn-glycerol-3-phosphate = diphosphate	glycerophospholipid metabolism	1	1	0	0	-	-		1
R-0275	CDP-diacylglycerol synthetase (n-18:1)	CTP + 1,2-dioctadec-9-enoyl-sn-glycerol-3-phosphate = diphosph	glycerophospholipid metabolism	1	1	0	0	-	-		1
R-0276	phosphatidylethanolamine-N-methyltransferase (n-	(S)-adenosyl-L-methionine + phosphatidylethanolamine(dihexad	glycerophospholipid metabolism	1	1	-1	0	-	-		1
R-0277	phosphatidylethanolamine-N-methyltransferase (n-	(S)-adenosyl-L-methionine + phosphatidylethanolamine(dihexad	glycerophospholipid metabolism	1	1	-1	0	-	-		1
R-0278	phosphatidylethanolamine-N-methyltransferase (n-	(S)-adenosyl-L-methionine + phosphatidylethanolamine(dioctade	glycerophospholipid metabolism	1	1	-1	0	-	-		1
R-0279	phosphatidylethanolamine-N-methyltransferase (n-	(S)-adenosyl-L-methionine + phosphatidylethanolamine(dioctade	glycerophospholipid metabolism	1	1	-1	0	-	-		1
R-0280	phosphatidylserine decarboxylase (n-16:0)	phosphatidyl-L-serine(dihexadecanoyl) = phosphatidylethanolam	glycerophospholipid metabolism	1	1	0	0	-	-		1
R-0281	phosphatidylserine decarboxylase (n-16:1)	phosphatidyl-L-serine(dihexadec-9-enoyl) = phosphatidylethanol	glycerophospholipid metabolism	1	1	0	0	-	-		1
R-0282	phosphatidylserine decarboxylase (n-18:0)	phosphatidyl-L-serine(dioctadecanoyl) = phosphatidylethanolam	glycerophospholipid metabolism	1	1	0	0	-	-		1
R-0283	phosphatidylserine decarboxylase (n-18:1)	phosphatidyl-L-serine(dioctadec-9-enoyl) = phosphatidylethanol	glycerophospholipid metabolism	1	1	0	0	-	-		1
R-0284	phosphatidylserine synthase (n-16:0)	CDP-1,2-dihexadecanoyl-glycerol + L-serine = CMP + phosphatidy	glycerophospholipid metabolism	1	1	-	-	-	-		1
R-0285	phosphatidylserine synthase (n-16:1)	CDP-1,2-dihexadec-9-enoyl-glycerol + L-serine = CMP + phosphati	glycerophospholipid metabolism	1	1	-	-	-	-		1
R-0286	phosphatidylserine synthase (n-18:0)	CDP-1,2-dioctadecanoyl-glycerol + L-serine = CMP + phosphatidyl	glycerophospholipid metabolism	1	1	-	-	-	-		1
R-0287	phosphatidylserine synthase (n-18:1)	CDP-1,2-dioctadec-9-enoyl-glycerol + L-serine = CMP + phosphati	glycerophospholipid metabolism	1	1	-	-	-	-		1
R-0289	phosphatidylglycerophosphate synthetase (n-16:0)	CDP-1,2-dihexadecanoyl-glycerol + sn-glycerol-3-phosphate = CM	glycerophospholipid metabolism	1	1	-	0	-	-		1
R-0290	phosphatidylglycerophosphate synthetase (n-16:1)	CDP-1,2-dihexadec-9-enoyl-glycerol + sn-glycerol-3-phosphate = C	glycerophospholipid metabolism	1	1	-	0	-	-		1
R-0291	phosphatidylglycerophosphate synthetase (n-18:0)	CDP-1,2-dioctadecanoyl-glycerol + sn-glycerol-3-phosphate = CM	glycerophospholipid metabolism	1	1	-	0	-	-		1
R-0292	phosphatidylglycerophosphate synthetase (n-18:1)	CDP-1,2-dioctadec-9-enoyl-glycerol + sn-glycerol-3-phosphate = C	glycerophospholipid metabolism	1	1	-	0	-	-		1
R-0294	sn-Glycero-3-phosphocholine glycerophosphohydrol	sn-glycero-3-phosphocholine + H2O = sn-glycerol-3-phosphate + (	glycerophospholipid degradation	0	0	0	0	-	-		0
R-0295	sn-Glycero-3-phosphoethanolamine glycerophosphc	sn-glycero-3-phosphoethanolamine + H2O = sn-glycerol-3-phosph	glycerophospholipid degradation	0	0	0	0	-	-		0
R-0298	lysophospholipase C1 (n-16:0)	1-hexadecanoyl-sn-glycero-3-phosphocholine + H2O = sn-glycero-	glycerophospholipid degradation	0	0	0	0	-	-		0
R-0299	lysophospholipase C2 (n-16:0)	2-hexadecanoyl-sn-glycero-3-phosphocholine + H2O = sn-glycero-	glycerophospholipid degradation	0	0	0	0	-	-		0
R-0300	lysophospholipase C1 (n-16:1)	1-hexadec-9-enoyl-sn-glycero-3-phosphocholine + H2O = sn-glyce	glycerophospholipid degradation	0	0	0	0	-	-		0
R-0301	lysophospholipase C2 (n-16:1)	2-hexadec-9-enoyl-sn-glycero-3-phosphocholine + H2O = sn-glyce	glycerophospholipid degradation	0	0	0	0	-	-		0
R-0302	lysophospholipase C1 (n-18:0)	1-octadecanoyl-sn-glycero-3-phosphocholine + H2O = sn-glycero-	glycerophospholipid degradation	0	0	0	0	-	-		0
R-0303	lysophospholipase C2 (n-18:0)	2-octadecanoyl-sn-glycero-3-phosphocholine + H2O = sn-glycero-	glycerophospholipid degradation	0	0	0	0	-	-		0
R-0304	lysophospholipase C1 (n-18:1)	1-octadec-9-enoyl-sn-glycero-3-phosphocholine + H2O = sn-glyce	glycerophospholipid degradation	0	0	0	0	-	-		0
R-0305	lysophospholipase C2 (n-18:1)	2-octadec-9-enoyl-sn-glycero-3-phosphocholine + H2O = sn-glyce	glycerophospholipid degradation	0	0	0	0	-	-		0
R-0308	lysophospholipase E1 (n-16:0)	1-hexadecanoyl-sn-glycero-3-phosphoethanolamine + H2O = sn-g	glycerophospholipid degradation	0	0	0	0	-	-		0
R-0309	lysophospholipase E2 (n-16:0)	2-hexadecanoyl-sn-glycero-3-phosphoethanolamine + H2O = sn-g	glycerophospholipid degradation	0	0	0	0	-	-		0
R-0310	lysophospholipase E1 (n-16:1)	1-hexadec-9-enoyl-sn-glycero-3-phosphoethanolamine + H2O = s	glycerophospholipid degradation	0	0	0	0	-	-		0
R-0311	lysophospholipase E2 (n-16:1)	2-hexadec-9-enoyl-sn-glycero-3-phosphoethanolamine + H2O = s	glycerophospholipid degradation	0	0	0	0	-	-		0
R-0312	lysophospholipase E1 (n-18:0)	1-octadecanoyl-sn-glycero-3-phosphoethanolamine + H2O = sn-g	glycerophospholipid degradation	0	0	0	0	-	-		0
R-0313	lysophospholipase E2 (n-18:0)	2-octadecanoyl-sn-glycero-3-phosphoethanolamine + H2O = sn-g	glycerophospholipid degradation	0	0	0	0	-	-		0
R-0314	lysophospholipase E1 (n-18:1)	1-octadec-9-enoyl-sn-glycero-3-phosphoethanolamine + H2O = sr	glycerophospholipid degradation	0	0	0	0	-	-		0
R-0315	lysophospholipase E2 (n-18:1)	2-octadec-9-enoyl-sn-glycero-3-phosphoethanolamine + H2O = sr	glycerophospholipid degradation	0	0	0	0	-	-		0
R-0317	2-acylglycerophosphoethanolamine acyltransferase	hexadecanoyl-[acyl-carrier-protein] + 2-hexadecanoyl-sn-glycero-	glycerophospholipid metabolism	1	1	0	0	-	-		1
R-0318	2-acylglycerophosphoethanolamine acyltransferase	hexadec-9-enoyl-[acyl-carrier-protein] + 2-hexadec-9-enoyl-sn-gly	glycerophospholipid metabolism	1	1	0	0	-	-		1
R-0319	2-acylglycerophosphoethanolamine acyltransferase	octadecanoyl-[acyl-carrier-protein] + 2-octadecanoyl-sn-glycero-3	glycerophospholipid metabolism	1	1	0	0	-	-		1
R-0320	2-acylglycerophosphoethanolamine acyltransferase	octadec-9-enoyl-[acyl-carrier-protein] + 2-octadec-9-enoyl-sn-gly	glycerophospholipid metabolism	1	1	0	0	-	-		1
R-0322	acyl-acyl carrier protein synthetase (n-16:0)	ATP + hexadecanoate + [acyl-carrier-protein] = AMP + diphosphat	glycerophospholipid metabolism	1	1	0	0	-	-		1
R-0323	acyl-acyl carrier protein synthetase (n-16:1)	ATP + 9-hexadecenoate + [acyl-carrier-protein] = AMP + diphosph	glycerophospholipid metabolism	1	1	0	0	-	-		1
R-0324	acyl-acyl carrier protein synthetase (n-18:0)	ATP + octadecanoate + [acyl-carrier-protein] = AMP + diphosphat	glycerophospholipid metabolism	1	1	0	0	-	-		1
R-0325	acyl-acyl carrier protein synthetase (n-18:1)	ATP + 9-octadecenoate + [acyl-carrier-protein] = AMP + diphosph	glycerophospholipid metabolism	1	1	0	0	-	-		1
R-0327	diacylglycerol kinase (n-16:0)	ATP + 1,2-dihexadecanoyl-glycerol = ADP + 1,2-dihexadecanoyl-sr	glycerophospholipid metabolism	1	1	1	0	-	-		1
R-0328	diacylglycerol kinase (n-16:1)	ATP + 1,2-dihexadec-9-enoyl-glycerol = ADP + 1,2-dihexadec-9-en	glycerophospholipid metabolism	1	1	1	0	-	-		1
R-0329	diacylglycerol kinase (n-18:0)	ATP + 1,2-dioctadecanoyl-glycerol = ADP + 1,2-dioctadecanoyl-sn	glycerophospholipid metabolism	1	1	1	0	-	-		1
R-0330	diacylglycerol kinase (n-18:1)	ATP + 1,2-dioctadec-9-enoyl-glycerol = ADP + 1,2-dioctadec-9-enc	glycerophospholipid metabolism	1	1	1	0	-	-		1
R-0331	dihydroxyacetone kinase	ATP + glycerone = ADP + glycerone-phosphate	dihydroxyacetone degradation	0	0	0	0	-	-		0
R-0332	glycerol kinase	ATP + glycerol = ADP + sn-glycerol-3-phosphate	glycerol degradation	0	0	-	-	-	-		0
R-0334	lysophosphatidic acid acyltransferase (n-16:0)	hexadecanoyl-[acyl-carrier-protein] + 1-hexadecanoyl-sn-glycerol	glycerophospholipid metabolism	1	1	0	0	-	-		1
R-0335	lysophosphatidic acid acyltransferase (n-16:1)	hexadec-9-enoyl-[acyl-carrier-protein] + 1-hexadec-9-enoyl-sn-gly	glycerophospholipid metabolism	1	1	0	0	-	-		1
R-0336	lysophosphatidic acid acyltransferase (n-18:0)	octadecanoyl-[acyl-carrier-protein] + 1-octadecanoyl-sn-glycerol-	glycerophospholipid metabolism	1	1	0	0	-	-		1
R-0337	lysophosphatidic acid acyltransferase (n-18:1)	octadec-9-enoyl-[acyl-carrier-protein] + 1-octadec-9-enoyl-sn-gly	glycerophospholipid metabolism	1	1	0	0	-	-		1
R-0338	malonyl-CoA-[acyl-carrier-protein] transacylase	malonyl-CoA + [acyl-carrier-protein] = CoA + malonyl-[acyl-carrier	fatty acid biosynthesis	1	1	0	0	-	-		1
R-0339	acetyl-CoA:ACP transacylase	acetyl-CoA + [acyl-carrier-protein] = acetyl-[acyl-carrier-protein] +	fatty acid biosynthesis	1	1	0	0	-	-		1
R-0340	3-oxoacyl-[acyl-carrier-protein] synthase (n-4:0)	acetyl-[acyl-carrier-protein] + malonyl-[acyl-carrier-protein] = ace	fatty acid biosynthesis	1	1	0	0	-	-		1
R-0341	3-oxoacyl-[acyl-carrier-protein] synthase (n-6:0)	butyryl-[acyl-carrier-protein] + malonyl-[acyl-carrier-protein] = 3-	fatty acid biosynthesis	1	1	0	0	-	-		1
R-0342	3-oxoacyl-[acyl-carrier-protein] synthase (n-8:0)	hexanoyl-[acyl-carrier-protein] + malonyl-[acyl-carrier-protein] =	fatty acid biosynthesis	1	1	0	0	-	-		1
R-0343	3-oxoacyl-[acyl-carrier-protein] synthase (n-10:0)	octanoyl-[acyl-carrier-protein] + malonyl-[acyl-carrier-protein] =	fatty acid biosynthesis	1	1	0	0	-	-		1
R-0344	3-oxoacyl-[acyl-carrier-protein] synthase (n-12:0)	decanoyl-[acyl-carrier-protein] + malonyl-[acyl-carrier-protein] =	fatty acid biosynthesis	1	1	0	0	-	-		1
R-0345	3-oxoacyl-[acyl-carrier-protein] synthase (n-14:0)	dodecanoyl-[acyl-carrier-protein] + malonyl-[acyl-carrier-protein]	fatty acid biosynthesis	1	1	0	0	-	-		1
R-0346	3-oxoacyl-[acyl-carrier-protein] synthase (n-16:0)	tetradecanoyl-[acyl-carrier-protein] + malonyl-[acyl-carrier-prote	fatty acid biosynthesis	1	1	0	0	-	-		1
R-0347	3-oxoacyl-[acyl-carrier-protein] synthase (n-18:0)	hexadecanoyl-[acyl-carrier-protein] + malonyl-[acyl-carrier-prote	fatty acid biosynthesis	1	1	0	0	-	-		1
R-0348	3-oxoacyl-[acyl-carrier-protein] synthase (n-12:1)	cis-dec-3-enoyl-[acyl-carrier-protein] + malonyl-[acyl-carrier-prot	fatty acid biosynthesis	1	1	0	0	-	-		1
R-0349	3-oxoacyl-[acyl-carrier-protein] synthase (n-14:1)	cis-dodec-5-enoyl-[acyl-carrier-protein] + malonyl-[acyl-carrier-pr	fatty acid biosynthesis	1	1	0	0	-	-		1
R-0350	3-oxoacyl-[acyl-carrier-protein] synthase (n-16:1)	cis-tetradec-7-enoyl-[acyl-carrier-protein] + malonyl-[acyl-carrier-	fatty acid biosynthesis	1	1	0	0	-	-		1
R-0351	3-oxoacyl-[acyl-carrier-protein] synthase (n-18:1)	hexadec-9-enoyl-[acyl-carrier-protein] + malonyl-[acyl-carrier-pro	fatty acid biosynthesis	1	1	0	0	-	-		1
R-0352	3-oxoacyl-[acyl-carrier-protein] reductase (n-4:0)	acetoacetyl-[acyl-carrier-protein] + NADPH + H+ = (3R)-3-hydroxyl	fatty acid biosynthesis	1	1	0	0	-	-		1
R-0353	3-oxoacyl-[acyl-carrier-protein] reductase (n-6:0)	3-oxohexanoyl-[acyl-carrier-protein] + NADPH + H+ = (3R)-3-hydrc	fatty acid biosynthesis	1	1	0	0	-	-		1
R-0354	3-oxoacyl-[acyl-carrier-protein] reductase (n-8:0)	3-oxooctanoyl-[acyl-carrier-protein] + NADPH + H+ = (3R)-3-hydro	fatty acid biosynthesis	1	1	0	0	-	-		1
R-0355	3-oxoacyl-[acyl-carrier-protein] reductase (n-10:0)	3-oxodecanoyl-[acyl-carrier-protein] + NADPH + H+ = (3R)-3-hydrc	fatty acid biosynthesis	1	1	0	0	-	-		1
R-0356	3-oxoacyl-[acyl-carrier-protein] reductase (n-12:0)	3-oxododecanoyl-[acyl-carrier-protein] + NADPH + H+ = (3R)-3-hy	fatty acid biosynthesis	1	1	0	0	-	-		1
R-0358	3-oxoacyl-[acyl-carrier-protein] reductase (n-14:0)	3-oxotetradecanoyl-[acyl-carrier-protein] + NADPH + H+ = (3R)-3-I	fatty acid biosynthesis	1	1	0	0	-	-		1
R-0359	3-oxoacyl-[acyl-carrier-protein] reductase (n-16:0)	3-oxohexadecanoyl-[acyl-carrier-protein] + NADPH + H+ = (3R)-3-I	fatty acid biosynthesis	1	1	0	0	-	-		1
R-0360	3-oxoacyl-[acyl-carrier-protein] reductase (n-18:0)	3-oxooctadecanoyl-[acyl-carrier-protein] + NADPH + H+ = (3R)-3-h	fatty acid biosynthesis	1	1	0	0	-	-		1
R-0361	3-oxoacyl-[acyl-carrier-protein] reductase (n-12:1)	3-oxo-cis-dodec-5-enoyl-[acyl-carrier-protein] + NADPH + H+ = (3F	fatty acid biosynthesis	1	1	0	0	-	-		1
R-0362	3-oxoacyl-[acyl-carrier-protein] reductase (n-14:1)	3-oxo-cis-tetradec-7-enoyl-[acyl-carrier-protein] + NADPH + H+ = (	fatty acid biosynthesis	1	1	0	0	-	-		1
R-0363	3-oxoacyl-[acyl-carrier-protein] reductase (n-16:1)	3-oxo-cis-hexadec-9-enoyl-[acyl-carrier-protein] + NADPH + H+ = (	fatty acid biosynthesis	1	1	0	0	-	-		1
R-0364	3-oxoacyl-[acyl-carrier-protein] reductase (n-18:1)	3-oxo-cis-octadec-11-enoyl-[acyl-carrier-protein] + NADPH + H+ =	fatty acid biosynthesis	1	1	0	0	-	-		1
R-0365	B-hydroxydecanoyl thioester dehydrase	(3R)-3-hydroxydecanoyl-[acyl-carrier-protein] = trans-dec-2-enoyl	fatty acid biosynthesis	1	1	0	0	-	-		1
R-0366	trans-2-decenoyl-ACP isomerase	trans-dec-2-enoyl-[acyl-carrier-protein] = cis-dec-3-enoyl-[acyl-ca	fatty acid biosynthesis	1	1	0	0	-	-		1
R-0367	3-hydroxyacyl-[acyl-carrier-protein] dehydratase (n-4	(3R)-3-hydroxybutanoyl-[acyl-carrier-protein] = crotonyl-[acyl-car	fatty acid biosynthesis	1	1	0	0	-	-		1
R-0368	3-hydroxyacyl-[acyl-carrier-protein] dehydratase (n-6	(3R)-3-hydroxyhexanoyl-[acyl-carrier-protein] = trans-hex-2-enoyl	fatty acid biosynthesis	1	1	0	0	-	-		1
R-0369	3-hydroxyacyl-[acyl-carrier-protein] dehydratase (n-8	(3R)-3-hydroxyoctanoyl-[acyl-carrier-protein] = trans-oct-2-enoyl-	fatty acid biosynthesis	1	1	0	0	-	-		1
R-0370	3-hydroxyacyl-[acyl-carrier-protein] dehydratase (n-1	(3R)-3-hydroxydodecanoyl-[acyl-carrier-protein] = trans-dodec-2-	fatty acid biosynthesis	1	1	0	0	-	-		1
R-0371	3-hydroxyacyl-[acyl-carrier-protein] dehydratase (n-1	(3R)-3-hydroxytetradecanoyl-[acyl-carrier-protein] = trans-tetrade	fatty acid biosynthesis	1	1	0	0	-	-		1
R-0372	3-hydroxyacyl-[acyl-carrier-protein] dehydratase (n-1	(3R)-3-hydroxyhexadecanoyl-[acyl-carrier-protein] = trans-hexade	fatty acid biosynthesis	1	1	0	0	-	-		1
R-0373	3-hydroxyacyl-[acyl-carrier-protein] dehydratase (n-1	(3R)-3-hydroxyoctadecanoyl-[acyl-carrier-protein] = trans-octade	fatty acid biosynthesis	1	1	0	0	-	-		1
R-0374	3-hydroxyacyl-[acyl-carrier-protein] dehydratase (n-1	(3R)-3-hydroxy-cis-dodec-5-enoyl-[acyl-carrier-protein] = trans-3-	fatty acid biosynthesis	1	1	0	0	-	-		1
R-0375	3-hydroxyacyl-[acyl-carrier-protein] dehydratase (n-1	(3R)-3-hydroxy-cis-tetradec-7-enoyl-[acyl-carrier-protein] = trans-	fatty acid biosynthesis	1	1	0	0	-	-		1
R-0376	3-hydroxyacyl-[acyl-carrier-protein] dehydratase (n-1	(3R)-3-hydroxy-cis-hexadec-9-enoyl-[acyl-carrier-protein] = trans-	fatty acid biosynthesis	1	1	0	0	-	-		1
R-0377	3-hydroxyacyl-[acyl-carrier-protein] dehydratase (n-1	(3R)-3-hydroxy-cis-octadec-11-enoyl-[acyl-carrier-protein] = trans	fatty acid biosynthesis	1	1	0	0	-	-		1
R-0378	enoyl-[acyl-carrier-protein] reductase (n-4:0)	crotonyl-[acyl-carrier-protein] + NADPH + H+ = butyryl-[acyl-carrie	fatty acid biosynthesis	1	1	0	0	-	-		1
R-0379	enoyl-[acyl-carrier-protein] reductase (n-6:0)	trans-hex-2-enoyl-[acyl-carrier-protein] + NADPH + H+ = hexanoyl	fatty acid biosynthesis	1	1	0	0	-	-		1
R-0380	enoyl-[acyl-carrier-protein] reductase (n-8:0)	trans-oct-2-enoyl-[acyl-carrier-protein] + NADPH + H+ = octanoyl-	fatty acid biosynthesis	1	1	0	0	-	-		1
R-0381	enoyl-[acyl-carrier-protein] reductase (n-10:0)	trans-dec-2-enoyl-[acyl-carrier-protein] + NADPH + H+ = decanoyl	fatty acid biosynthesis	1	1	0	0	-	-		1
R-0382	enoyl-[acyl-carrier-protein] reductase (n-12:0)	trans-dodec-2-enoyl-[acyl-carrier-protein] + NADPH + H+ = dodec	fatty acid biosynthesis	1	1	0	0	-	-		1
R-0383	enoyl-[acyl-carrier-protein] reductase (n-14:0)	trans-tetradec-2-enoyl-[acyl-carrier-protein] + NADPH + H+ = tetr	fatty acid biosynthesis	1	1	0	0	-	-		1
R-0384	enoyl-[acyl-carrier-protein] reductase (n-16:0)	trans-hexadec-2-enoyl-[acyl-carrier-protein] + NADPH + H+ = hexa	fatty acid biosynthesis	1	1	0	0	-	-		1
R-0385	enoyl-[acyl-carrier-protein] reductase (n-18:0)	trans-octadec-2-enoyl-[acyl-carrier-protein] + NADPH + H+ = octa	fatty acid biosynthesis	1	1	0	0	-	-		1
R-0386	enoyl-[acyl-carrier-protein] reductase (n-12:1)	trans-3-cis-5-dodecenoyl-[acyl-carrier-protein] + NADPH + H+ = ci	fatty acid biosynthesis	1	1	0	0	-	-		1
R-0387	enoyl-[acyl-carrier-protein] reductase (n-14:1)	trans-3-cis-7-tetradecenoyl-[acyl-carrier-protein] + NADPH + H+ =	fatty acid biosynthesis	1	1	0	0	-	-		1
R-0388	enoyl-[acyl-carrier-protein] reductase (n-16:1)	trans-3-cis-9-hexadecenoyl-[acyl-carrier-protein] + NADPH + H+ =	fatty acid biosynthesis	1	1	0	0	-	-		1

R-0389	enoyl-[acyl-carrier-protein] reductase (n-18:1)	trans-3-cis-11-octadecenoyl-[acyl-carrier-protein] + NADPH + H+ =	fatty acid biosynthesis	1	1	0	0	-	-		1
R-0390	glutaminase	L-glutamine + H2O = L-glutamate + NH3	L-glutamate/L-glutamine degradatio	0	0	0	0	-	-		0
R-0391	L-glutamine:D-fructose-6-phosphate aminotransfera	L-glutamine + D-fructose-6-phosphate = L-glutamate + D-glucosar	aminosugar metabolism	1	1	0	0	-	-		1
R-0392	glutamate racemase	L-glutamate = D-glutamate	glutamate metabolism	1	1	0	0	-	-		1
R-0393	aspartate aminotransferase	L-aspartate + 2-oxoglutarate = oxaloacetate + L-glutamate	aspartate metabolism	1	1	0	0	-	-		1
R-0394	GMP synthetase	ATP + XMP + L-glutamine + H2O = AMP + diphosphate + GMP + L-g	purine biosynthesis	1	1	0	0	-	1		1
R-0395	GMP synthetase (ammonia dependent)	NH3 + XMP + ATP = AMP + diphosphate + GMP	purine biosynthesis	0	0	0	0	-	1		0
R-0396	glutamyl-tRNA synthetase	ATP + L-glutamate + tRNA(Glu) = AMP + diphosphate + L-glutamyl	glutamate metabolism	1	1	0	0	-	-		1
R-0397	glutamine synthetase	ATP + L-glutamate + NH3 = ADP + phosphate + L-glutamine	glutamate metabolism	1	1	0	0	-	-		1
R-0398	gamma-glutamylcysteine synthetase	ATP + L-glutamate + L-cysteine = ADP + phosphate + gamma-L-glu	glutathione metabolism	1	1	0	0	-	-		1
R-0399	glutathione synthetase	ATP + gamma-L-glutamyl-L-cysteine + glycine = ADP + phosphate	glutathione metabolism	1	1	0	0	-	-		1
R-0400	glutamine-dependent NAD(+) synthetase	ATP + nicotinate-adenine-dinucleotide + L-glutamine + H2O = AM	NAD biosynthesis	1	1	0	0	-	-		1
R-0401	amidophosphoribosyltransferase (PRPP amidotransf	L-glutamine + 5-phosphoribosyl-1-pyrophosphate + H2O = 5-phos	purine biosynthesis	1	1	0	0	-	-		1
R-0402	glutamate synthase 1	NH3 + 2-oxoglutarate + NADPH + H+ = L-glutamate + NADP+ + H2(	glutamate metabolism	0	0	0	0	-	1		0
R-0403	glutamate synthase 2	L-glutamine + 2-oxoglutarate + NADPH + H+ = 2 L-glutamate + NA	glutamate metabolism	1	1	0	0	-	1		1
R-0404	carbamoyl phosphate synthetase	2 ATP + L-glutamine + HCO3- + H+ + H2O = 2 ADP + phosphate + L-	pyrimidine biosynthesis	1	1	0	0	-	-		1
R-0405	cysteine synthase	O-acetyl-L-serine + H2S = L-cysteine + acetate	cysteine metabolism	1	1	0	0	-	-		1
R-0406	serine acetyltransferase	acetyl-CoA + L-serine = CoA + O-acetyl-L-serine	cysteine metabolism	1	1	0	0	-	-		1
R-0407	D-cysteine desulfhydrase	D-cysteine + H2O = H2S + NH3 + pyruvate	cysteine degradation	0	0	0	0	-	-		0
R-0408	cysteine tRNA synthetase	ATP + L-cysteine + tRNA(Cys) = AMP + diphosphate + L-cysteinyl-tl	cysteine metabolism	1	1	0	0	-	-		1
R-0409	3-mercaptopyruvate sulfurtransferase	3-mercaptopyruvate + cyanide = pyruvate + thiocyanate	cysteine degradation	0	0	0	0	-	-		0
R-0410	pyrroline-5-carboxylate reductase	(S)-1-pyrroline-5-carboxylate + NADPH + H+ = L-proline + NADP+	arginine and proline metabolism	1	1	0	0	-	-		1
R-0411	proline dehydrogenase	L-proline + FAD = (S)-1-pyrroline-5-carboxylate + FADH2	arginine and proline degradation	0	0	-	-	-	-		0
R-0412	1-pyrroline-5-carboxylate dehydrogenase	(S)-1-pyrroline-5-carboxylate + NAD+ + 2 H2O = L-glutamate + NAI	arginine and proline degradation	0	0	-	-	-	-		0
R-0413	ornithine cyclodeaminase	L-ornithine = L-proline + NH3	arginine and proline degradation	0	0	-	0	-	-		0
R-0414	arginase	L-arginine + H2O = L-ornithine + urea	arginine and proline degradation	0	0	-	0	-	-		0
R-0415	ornithine carbamoyltransferase	carbamoyl-phosphate + L-ornithine = phosphate + L-citrulline	arginine and proline metabolism	1	1	0	0	-	-		1
R-0416	proline-tRNA ligase	ATP + L-proline + tRNA(Pro) = AMP + diphosphate + L-prolyl-tRNA	arginine and proline metabolism	1	1	0	0	-	-		1
R-0417	arginyl-tRNA synthetase	ATP + L-arginine + tRNA(Arg) = AMP + diphosphate + L-arginyl-tRN	arginine and proline metabolism	1	1	0	0	-	-		1
R-0418	UDP-N-acetylenolpyruvoylglucosamine reductase	UDP-N-acetyl-3-O-(1-carboxyvinyl)-D-glucosamine + NADPH + H+	aminosugar metabolism	1	1	0	0	-	-		1
R-0419	N-acetylglucosamine-6-phosphate deacetylase	N-acetyl-D-glucosamine-6-phosphate + H2O = D-glucosamine-6-p	aminosugar degradation	0	0	0	0	-	-		0
R-0420	UDP-N-acetylglucosamine 1-carboxyvinyltransferase	phosphoenolpyruvate + UDP-N-acetyl-D-glucosamine = phosphat	aminosugar metabolism	1	1	0	0	-	-		1
R-0421	beta N-acetyl-glucosaminidase	GlcNAc-1,6-anhMurNAc + H2O = 1,6-anhydro-N-acetylmuramate	murein tripeptide recycling	0	0	0	0	-	-		0
R-0422	N-acetylglucosamine-1-phosphate uridyltransferase	N-acetyl-glucosamine-1-phosphate + UTP = UDP-N-acetyl-D-gluco	aminosugar metabolism	1	1	0	0	-	-		1
R-0423	glucosamine-1-phosphate acetyltransferase	D-glucosamine-1-phosphate + acetyl-CoA = N-acetyl-glucosamine	aminosugar metabolism	1	1	0	0	-	-		1
R-0424	phosphoglucosamine mutase	D-glucosamine-1-phosphate = D-glucosamine-6-phosphate	aminosugar metabolism	1	1	0	0	-	-		1
R-0425	aspartate ammonia-lyase	L-aspartate = fumarate + NH3 + H+	aspartate/asparagine degradation	0	0	-1	-1	-	-	kept set to 0 : degradation pathwa	0
R-0426	L-aspartate oxidase	L-aspartate + O2 = a-iminosuccinate + H2O2 + H+	NAD biosynthesis	1	1	0	0	-	-		1
R-0427	quinolinate synthetase	a-iminosuccinate + glycerone-phosphate = quinolinate + phospha	NAD biosynthesis	1	1	0	0	-	-		1
R-0428	quinolinate phosphoribosyltransferase	5-phosphoribosyl-1-pyrophosphate + quinolinate = CO2 + diphos	NAD biosynthesis	1	1	0	0	-	-		1
R-0429	L-asparaginase	L-asparagine + H2O = L-aspartate + NH3	aspartate/asparagine degradation	0	0	0	0	-	-		0
R-0430	alanine racemase	L-alanine = D-alanine	alanine metabolism	1	1	0	0	-	-		1
R-0431	adenylosuccinate synthetase	GTP + IMP + L-aspartate = GDP + phosphate + adenylo-succinate	purine biosynthesis	1	1	0	0	-	-		1
R-0432	aspartate carbamoyltransferase	carbamoyl-phosphate + L-aspartate = phosphate + N-carbamoyl-l	pyrimidine biosynthesis	1	1	0	0	-	-		1
R-0433	argininosuccinate synthase	ATP + L-citrulline + L-aspartate = AMP + diphosphate + L-arginino-	arginine and proline metabolism	1	1	0	0	-	-		1
R-0434	aspartate tRNA synthetase	ATP + L-aspartate + tRNA(Asp) = AMP + diphosphate + L-aspartyl-t	aspartate metabolism	1	1	0	0	-	-		1
R-0435	asparagine synthase	L-glutamine + L-aspartate + ATP + H2O = L-glutamate + L-asparagi	aspartate metabolism	1	1	0	0	-	-		1
R-0436	alanyl-tRNA synthetase	ATP + L-alanine + tRNA(Ala) = AMP + diphosphate + L-alanyl-tRNA	alanine metabolism	1	1	0	0	-	-		1
R-0437	argininosuccinate lyase	L-arginino-succinate = L-arginine + fumarate	arginine and proline metabolism	1	1	0	0	-	-		1
R-0438	adenylosuccinate lyase	adenylo-succinate = fumarate + AMP	purine biosynthesis	1	1	0	0	-	-		1
R-0439	5'-phosphoribosyl-4-(N-succinocarboxamide)-5-amin	5'-phosphoribosyl-4-(N-succinocarboxamide)-5-aminoimidazole =	purine biosynthesis	1	1	0	0	-	-		1
R-0440	succinate-semialdehyde dehydrogenase	succinate-semialdehyde + NADP+ + H2O = succinate + NADPH + H	4-hydroxyphenylacetate degradatio	0	0	0	0	-	-		0
R-0441	guanine phosphoribosyltransferase	5-phosphoribosyl-1-pyrophosphate + guanine = diphosphate + GI	purine biosynthesis	1	1	0	0	-	-		1
R-0442	hypoxanthine phosphoribosyltransferase	5-phosphoribosyl-1-pyrophosphate + hypoxanthine = diphosphat	purine biosynthesis	1	1	0	0	-	-		1
R-0443	ATP synthase	ADP + phosphate + 4 H+[p] = ATP + H2O + 4 H+	oxidative phosphorilation	1	1	0	0	-	-		1
R-0444	cytochrome c oxidase	2 reduced-cytochrome-c + 0.5 O2 + 4 H+ = 2 oxidized-cytochrome-	oxidative phosphorilation	1	1	0	0	-	-		1
R-0445	cytochrome o ubiquinol oxidase	1 UQH2 + 0.5 O2 + 4 H+ = 1 UQ + 1 H2O + 4 H+[p]	oxidative phosphorilation	1	1	0	0	-	-		1
R-0446	ubiquinol-cytochrome c reductase	2 oxidized-cytochrome-c + UQH2 = 2 reduced-cytochrome-c + UQ	oxidative phosphorilation	1	1	0	-1	-	-		1
R-0447	NADH:ubiquinone oxidoreductase I	NADH + UQ + 5 H+ = NAD+ + UQH2 + 4 H+[p]	oxidative phosphorilation	1	1	0	0	-	-		1
R-0448	NAD(P) transhydrogenase	NADP+ + NADH + H+[p] = NADPH + NAD+ + H+	oxidative phosphorilation	0	1	-1	-1	-	-	set usable during succiante growt	1
R-0449	phosphoribosylglycinamide synthetase	5-phospho-beta-D-ribosylamine + ATP + glycine = ADP + phospha	purine biosynthesis	1	1	0	0	-	-		1
R-0450	phosphoribosylglycinamide formyltransferase	5-phospho-ribosyl-glycineamide + 10-formyltetrahydrofolate = 5'	purine biosynthesis	1	1	-1	0	-	-		1
R-0451	phosphoribosylformylglycinamide synthetase	ATP + 5'-phosphoribosyl-N-formylglycineamide + L-glutamine + H	purine biosynthesis	1	1	0	0	-	-		1
R-0452	phosphoribosylaminoimidazole synthetase	ATP + 5-phosphoribosyl-N-formylglycineamidine = ADP + phospha	purine biosynthesis	1	1	0	0	-	-		1
R-0453	phosphoribosylaminoimidazole carboxylase	5-aminoimidazole-ribonucleotide + CO2 = 4-carboxyaminoimidaz	purine biosynthesis	1	1	0	0	-	-		1
R-0454	phosphoribosylaminoimidazole-succinocarboxamide	ATP + 4-carboxyaminoimidazole-ribonucleotide + L-aspartate = A	purine biosynthesis	1	1	0	0	-	-		1
R-0455	phosphoribosylaminoimidazolecarboxamide formylt	aminoimidazole-carboxamide-ribonucleotide + 10-formyltetrahy	purine biosynthesis	1	1	0	0	-	-		1
R-0456	inosine monophosphate cyclohydrolase	phosphoribosyl-formamido-carboxamide = IMP + H2O	purine biosynthesis	1	1	0	0	-	-		1
R-0457	IMP dehydrogenase	IMP + NAD+ + H2O = XMP + NADH + H+	purine biosynthesis	1	1	0	0	-	-		1
R-0458	guanylate kinase	GMP + ATP = GDP + ADP	purine biosynthesis	1	1	0	0	-	-		1
R-0459	deoxyguanylate kinase	dGMP + ATP = dGDP + ADP	purine biosynthesis	1	1	0	0	-	-		1
R-0460	UDP kinase	UDP + ATP = UTP + ADP	pyrimidine biosynthesis	1	1	0	0	-	-		1
R-0461	CDP kinase	CDP + ATP = CTP + ADP	pyrimidine biosynthesis	1	1	0	0	-	-		1
R-0462	dUDP kinase	dUDP + ATP = dUTP + ADP	pyrimidine biosynthesis	0	0	0	0	-	1		0
R-0463	dCDP kinase	dCDP + ATP = dCTP + ADP	pyrimidine biosynthesis	1	1	0	0	-	-		1
R-0464	dTDP kinase	dTDP + ATP = dTTP + ADP	pyrimidine biosynthesis	1	1	0	0	-	-		1
R-0465	dADP kinase	dADP + ATP = dATP + ADP	purine biosynthesis	1	1	0	0	-	-		1
R-0466	dGDP kinase	dGDP + ATP = dGTP + ADP	purine biosynthesis	1	1	0	0	-	-		1
R-0467	GDP kinase	GDP + ATP = GTP + ADP	purine biosynthesis	1	1	0	0	-	-		1
R-0469	CTP synthetase	ATP + UTP + L-glutamine + H2O = ADP + phosphate + CTP + L-gluta	pyrimidine biosynthesis	1	1	0	0	-	-		1
R-0470	UDP reductase	UDP + reduced-thioredoxin = dUDP + oxidized-thioredoxin + H2O	pyrimidine biosynthesis	0	0	0	0	-	1		0
R-0471	CDP reductase	CDP + reduced-thioredoxin = dCDP + oxidized-thioredoxin + H2O	pyrimidine biosynthesis	1	1	0	0	-	-		1
R-0472	ADP reductase	ADP + reduced-thioredoxin = dADP + oxidized-thioredoxin + H2O	purine biosynthesis	1	1	0	0	-	-		1
R-0474	GDP reductase	GDP + reduced-thioredoxin = dGDP + oxidized-thioredoxin + H2O	purine biosynthesis	1	1	0	0	-	-		1
R-0475	thioredoxin reductase	oxidized-thioredoxin + NADPH + H+ = reduced-thioredoxin + NAD	purine biosynthesis	1	1	0	0	-	-		1
R-0476	adenylate kinase	AMP + ATP = 2 ADP	purine biosynthesis	1	1	0	0	-	-		1
R-0477	dihydroorotase	N-carbamoyl-L-aspartate = dihydroorotate + H2O	pyrimidine biosynthesis	1	1	0	0	-	-		1
R-0478	dihydroorotate dehydrogenase	dihydroorotate + O2 = orotate + H2O2	pyrimidine biosynthesis	1	1	0	0	-	-		1
R-0479	orotate phosphoribosyltransferase	5-phosphoribosyl-1-pyrophosphate + orotate = orotidine-5'-phos	pyrimidine biosynthesis	1	1	0	0	-	-		1
R-0480	orotidine 5'-phosphate decarboxylase	orotidine-5'-phosphate = CO2 + UMP	pyrimidine biosynthesis	1	1	-	-	-	-		1
R-0481	cytidylate kinase	CMP + ATP = CDP + ADP	pyrimidine biosynthesis	1	1	0	0	-	-		1
R-0482	uridylate kinase	UMP + ATP = UDP + ADP	pyrimidine biosynthesis	1	1	-1	0	-	-		1
R-0483	dCTP deaminase	dCTP + H2O = NH3 + dUTP	pyrimidine biosynthesis	1	1	0	0	-	1		1
R-0484	thymidylate synthetase	dUMP + 5,10-methylenetetrahydrofolate = dTMP + 7,8-dihydrofo	pyrimidine biosynthesis	1	1	-1	0	-	-		1
R-0485	dihydrofolate reductase	NADPH + 7,8-dihydrofolate + H+ = NADP+ + tetrahydrofolate	folate biosynthesis	1	1	-	0	-	-		1
R-0486	deoxyuridine 5'-triphosphate nucleotidohydrolase	dUTP + H2O = dUMP + diphosphate	pyrimidine biosynthesis	1	1	0	0	-	-		1
R-0487	nucleoside triphosphate pyrophosphohydrolase (ATI	ATP + H2O = AMP + diphosphate	salvage pathways of purine and pyri	0	0	0	0	-	-		0
R-0489	nucleoside triphosphate pyrophosphohydrolase (GT	GTP + H2O = GMP + diphosphate	salvage pathways of purine and pyri	0	0	0	0	-	-		0
R-0490	nucleoside triphosphate pyrophosphohydrolase (CTI	CTP + H2O = CMP + diphosphate	salvage pathways of purine and pyri	0	0	0	0	-	-		0
R-0491	nucleoside triphosphate pyrophosphohydrolase (UT	UTP + H2O = UMP + diphosphate	salvage pathways of purine and pyri	0	0	0	0	-	-		0
R-0492	5'-nucleotidase (GMP)	GMP + H2O = guanosine + phosphate	salvage pathways of purine and pyri	0	0	0	0	-	-		0
R-0493	5'-nucleotidase (AMP)	AMP + H2O = adenosine + phosphate	salvage pathways of purine and pyri	0	0	0	0	-	-		0
R-0494	5'-nucleotidase (IMP)	IMP + H2O = inosine + phosphate	salvage pathways of purine and pyri	0	0	0	0	-	-		0
R-0495	5'-nucleotidase (XMP)	XMP + H2O = xanthosine + phosphate	salvage pathways of purine and pyri	0	0	0	0	-	-		0
R-0496	GTP cyclohydrolase I	GTP + H2O = formate + 7,8-dihydroneopterin-triphosphate	folate biosynthesis	1	1	0	0	-	-		1
R-0497	dihydroneopterin triphosphate pyrophosphohydrola	7,8-dihydroneopterin-triphosphate + H2O = dihydroneopterin-ph	folate biosynthesis	1	1	-	-	-	-		1
R-0498	dihydroneopterin aldolase	7,8-dihydro-D-neopterin = glycolaldehyde + 6-hydroxymethyl-7,8	folate biosynthesis	1	1	0	0	-	-		1
R-0499	dihydroneopterin monophosphate dephosphorylase	dihydroneopterin-phosphate + H2O = 7,8-dihydro-D-neopterin +	folate biosynthesis	1	1	-	-	-	-		1
R-0500	2-amino-4-hydroxy-6-hydroxymethyldihydropteridin	6-hydroxymethyl-7,8-dihydropterin + ATP = 6-hydroxymethyl-dih	folate biosynthesis	1	1	0	0	-	-		1
R-0501	dihydropteroate synthase	p-aminobenzoate + 6-hydroxymethyl-dihydropterin-diphosphate	folate biosynthesis	1	1	0	0	-	-		1
R-0502	folylpolyglutamate synthase 1	L-glutamate + ATP + methylene-tetrahydropteroyl-[gamma-Glu](r	folate biosynthesis	1	1	0	-1	-	-		1
R-0503	dihydrofolate synthetase 1	L-glutamate + ATP + 7,8-dihydropteroate = phosphate + ADP + 7,8	folate biosynthesis	1	1	0	-1	-	-		1
R-0504	folylpolyglutamate synthase 2	L-glutamate + ATP + 10-formyl-tetrahydropteroyl-[gamma-Glu](n)	folate biosynthesis	1	1	0	-1	-	-		1
R-0505	folylpolyglutamate synthase 3	L-glutamate + ATP + tetrahydropteroyl-[gamma-Glu](n) = ADP + p	folate biosynthesis	1	1	0	-1	-	-		1
R-0506	dihydromethanopterin reductase	NADPH + 7,8-dihydromethanopterin + H+ = NADP+ + tetrahydrom	folate biosynthesis	1	1	0	0	-	-		1
R-0507	1-deoxy-D-xylulose 5-phosphate reductoisomerase	1-deoxy-D-xylulose-5-phosphate + NADPH + H+ = 2-C-methyl-D-er	carotenoids biosynthesis	1	1	-	-	-	-		1
R-0508	4-diphosphocytidyl-2C- methyl-D-erythritol synthase	2-C-methyl-D-erythritol-4-phosphate + CTP = 4-(cytidine-5'-dipho	carotenoids biosynthesis	1	1	0	0	-	-		1
R-0509	2C-methyl-D-erythritol 2,4-cyclodiphosphate synthas	2-phospho-4-(cytidine-5'-diphospho)-2-C-methyl-D-erythritol = 2-	carotenoids biosynthesis	1	1	0	0	-	-		1
R-0510	4-diphosphocytidyl-2-C-methyl-D-erythritol kinase	4-(cytidine-5'-diphospho)-2-C-methyl-D-erythritol + ATP = 2-phos	carotenoids biosynthesis	1	1	0	0	-	-		1
R-0511	1-hydroxy-2-methyl-2-(E)-butenyl-4-diphosphate syn	2-C-methyl-D-erythritol-2,4-cyclodiphosphate + 2 reduced-ferred	carotenoids biosynthesis	1	1	0	0	-	-		1
R-0512	1-hydroxy-2-methyl-2-(E)-butenyl-4-diphosphate red	1-hydroxy-2-methyl-2-(E)-butenyl-4-diphosphate + NADPH + H+ =	carotenoids biosynthesis	1	1	0	0	-	-		1
R-0513	1-hydroxy-2-methyl-2-(E)-butenyl-4-diphosphate red	1-hydroxy-2-methyl-2-(E)-butenyl-4-diphosphate + NADPH + H+ =	carotenoids biosynthesis	1	1	0	0	-	-		1
R-0514	geranyl diphosphate synthase	dimethylallyl-diphosphate + isopentenyl-diphosphate = diphospł	carotenoids biosynthesis	1	1	0	0	-	-		1
R-0515	farnesyl-diphosphate synthase	geranyl-diphosphate + isopentenyl-diphosphate = diphosphate +	carotenoids biosynthesis	1	1	0	0	-	-		1
R-0516	geranylgeranyl pyrophosphate synthase	isopentenyl-diphosphate + all-trans-farnesyl-diphosphate = all-tr	carotenoids biosynthesis	1	1	0	0	-	-		1
R-0517	phytoene synthase 1	2 all-trans-geranyl-geranyl-diphosphate = prephytoene-diphosph	carotenoids biosynthesis	1	1	0	0	-	-		1
R-0518	phytoene synthase 2	prephytoene-diphosphate = phytoene + diphosphate	carotenoids biosynthesis	1	1	0	0	-	-		1
R-0519	phytoene dehydrogenase 1	phytoene + reduced-electron-acceptor + O2 = phytofluene + oxid	carotenoids biosynthesis	1	1	0	0	-	-		1
R-0520	phytoene dehydrogenase 2	phytofluene + reduced-electron-acceptor + O2 = zeta-carotene +	carotenoids biosynthesis	1	1	0	0	-	-		1
R-0521	zeta-carotene desaturase 1	zeta-carotene + reduced-electron-acceptor + O2 = neurosporene	carotenoids biosynthesis	1	1	0	0	-	-		1
R-0522	zeta-carotene desaturase 2	neurosporene + reduced-electron-acceptor + O2 = trans-lycopene	carotenoids biosynthesis	1	1	0	0	-	-		1
R-0523	prephenate dehydrogenase	prephenate + NAD+ = 4-hydroxyphenylpyruvate + CO2 + NADH +	phenylalanine, tyrosine, tryptophar	1	1	0	0	-	-		1
R-0524	aromatic-amino-acid transaminase (PHE)	phenylpyruvate + L-glutamate = L-phenylalanine + 2-oxoglutarate	phenylalanine, tyrosine, tryptophar	1	1	0	0	-	-		1
R-0525	aromatic-amino-acid transaminase (TYR)	4-hydroxyphenylpyruvate + L-glutamate = L-tyrosine + 2-oxogluta	phenylalanine, tyrosine, tryptophar	1	1	0	0	-	-		1
R-0526	D-alanine aminotransferase	pyruvate + D-glutamate = D-alanine + 2-oxoglutarate	alanine metabolism	1	1	0	0	-	-		1
R-0527	histidinol-phosphatase	L-histidinol-phosphate + H2O = L-histidinol + phosphate	histidine biosynthesis	1	1	-	-	-	-		1
R-0529	acyl phosphate synthase (n-16:0)	hexadecanoyl-[acyl-carrier-protein] + phosphate = hexadecanoyl-	glycerophospholipid metabolism	1	1	0	0	-	-		1
R-0530	acyl phosphate synthase (n-16:1)	hexadec-9-enoyl-[acyl-carrier-protein] + phosphate = hexadec-9-e	glycerophospholipid metabolism	1	1	0	0	-	-		1
R-0531	acyl phosphate synthase (n-18:0)	octadecanoyl-[acyl-carrier-protein] + phosphate = octadecanoyl-p	glycerophospholipid metabolism	1	1	0	0	-	-		1
R-0532	acyl phosphate synthase (n-18:1)	octadec-9-enoyl-[acyl-carrier-protein] + phosphate = octadec-9-e	glycerophospholipid metabolism	1	1	0	0	-	-		1
R-0534	glycerol-3-phosphate synthetase (n-16:0)	sn-glycerol-3-phosphate + hexadecanoyl-phosphate = 1-hexadeca	glycerophospholipid metabolism	1	1	1	0	-	-		1
R-0535	glycerol-3-phosphate synthetase (n-16:1)	sn-glycerol-3-phosphate + hexadec-9-enoyl-phosphate = 1-hexad	glycerophospholipid metabolism	1	1	1	0	-	-		1
R-0536	glycerol-3-phosphate synthetase (n-18:0)	sn-glycerol-3-phosphate + octadecanoyl-phosphate = 1-octadeca	glycerophospholipid metabolism	1	1	1	0	-	-		1
R-0537	glycerol-3-phosphate synthetase (n-18:1)	sn-glycerol-3-phosphate + octadec-9-enoyl-phosphate = 1-octade	glycerophospholipid metabolism	1	1	1	0	-	-		1
R-0538	thymidylate kinase	dTMP + ATP = dTDP + ADP	pyrimidine biosynthesis	1	1	0	0	-	-		1
R-0539	adenylate kinase 2	dAMP + ATP = ADP + dADP	purine biosynthesis	1	1	0	0	-	-		1
R-0540	glutamyl-tRNA amidotransferase	ATP + L-glutamyl-tRNA(Gln) + L-glutamine + H2O = ADP + phospha	glutamate metabolism	1	1	0	0	-	-		1
R-0541	nondiscriminating glutamyl-tRNA synthetase	ATP + L-glutamate + tRNA(Gln) = AMP + diphosphate + L-glutamyl	glutamate metabolism	1	1	0	0	-	-		1
R-0542	nondiscriminating aspartate tRNA synthetase	ATP + L-aspartate + tRNA(Asn) = AMP + diphosphate + L-aspartyl-t	aspartate metabolism	1	1	0	0	-	-		1
R-0543	aspartyl-tRNA amidotransferase	ATP + L-aspartyl-tRNA(Asn) + L-glutamine + H2O = ADP + phospha	aspartate metabolism	1	1	0	0	-	-		1
R-0544	gamma-glutamyl kinase	L-glutamate + ATP = L-glutamate-5-phosphate + ADP	arginine and proline metabolism	1	1	0	-1	-	-		1
R-0545	glutamate-5-semialdehyde dehydrogenase	L-glutamate-5-phosphate + NADPH + H+ = L-glutamate-gamma-se	arginine and proline metabolism	1	1	0	-1	-	-		1
R-0546	L-glutamate-gamma-semialdehyde (cyclase dehydrat	L-glutamate-gamma-semialdehyde = (S)-1-pyrroline-5-carboxylate	arginine and proline metabolism	1	1	-	-	-	-		1
R-0547	N-acetylglutamate synthase	L-glutamate + acetyl-CoA = N-acetyl-L-glutamate + CoA	arginine and proline metabolism	1	1	0	0	-	-		1
R-0548	acetylglutamate kinase	N-acetyl-L-glutamate + ATP = N-acetylglutamyl-phosphate + ADP	arginine and proline metabolism	1	1	0	0	-	-		1
R-0549	N-acetylglutamylphosphate reductase	N-acetylglutamyl-phosphate + NADPH + H+ = N-acetyl-L-glutamat	arginine and proline metabolism	1	1	0	0	-	-		1
R-0550	acetylornithine deacetylase	N-acetyl-L-ornithine + H2O = L-ornithine + acetate	arginine and proline metabolism	0	0	0	0	-	1		0
R-0551	glutamate N-acetyltransferase	L-glutamate + N-acetyl-L-ornithine = N-acetyl-L-glutamate + L-orn	arginine and proline metabolism	1	1	0	0	-	1		1
R-0552	methylation	(S)-adenosyl-L-methionine + demethylated-methyl-acceptor = (S)	methionine biosynthesis	1	1	-	-	-	-		1
R-0553	tryptophanyl tRNA synthetase	ATP + L-tryptophan + tRNA(Trp) = AMP + diphosphate + L-tryptop	phenylalanine, tyrosine, tryptophar	1	1	0	0	-	-		1
R-0554	phosphoglucose isomerase 2	b-D-glucose-6-phosphate = D-fructose-6-phosphate	starch and sucrose metabolism	1	1	0	0	-	-		1
R-0555	glucokinase 2	ATP + b-D-glucose = ADP + b-D-glucose-6-phosphate	starch and sucrose metabolism	1	1	1	0	-	-		1
R-0556	2'-(5''-triphosphoribosyl)-3'-dephosphocoenzyme-A s	dephospho-CoA + ATP = 2'-(5-triphosphoribosyl)-3'-dephospho-C	2'-(5'-phosphoribosyl)-3'-dephospho	1	1	0	0	-	-		1
R-0557	phosphoribosyl-dephospho-CoA transferase	2'-(5-triphosphoribosyl)-3'-dephospho-CoA + malonate-decarbox	2'-(5'-phosphoribosyl)-3'-dephospho	1	1	-	-	-	-		1
R-0558	Isoquinoline 1-oxidoreductase	isoquinoline + oxidized-electron-acceptor + H2O = isoquinolin-1(2	unassigned	1	1	1	0	-	-		1
R-0559	polysulphide reductase	S(n)2- + reduced-electron-acceptor = S(n-1)2- + H2S + oxidized-ele	sulfur metabolism	0	0	-	-	-	-		0
R-0560	potassium-transporting ATPase	K+[p] + H2O + ATP = K+ + phosphate + ADP	transport	1	1	0	0	-	-		1
R-0561	myo-inositol-1-phosphate synthase	b-D-glucose-6-phosphate = D-myo-inositol-3-monophosphate	inositol metabolism	1	1	0	0	-	-		1
R-0562	pyridoxamine 5'-phosphate oxidase	pyridoxamine-5'-phosphate + O2 + H2O = H2O2 + NH3 + pyridoxa	pyridoxal-5'-phosphate biosynthesis	0	0	0	0	-	-		0
R-0563	pyridoxine 5'-phosphate oxidase	pyridoxine-5'-phosphate + O2 = H2O2 + pyridoxal-5'-phosphate	pyridoxal-5'-phosphate biosynthesis	1	1	0	0	-	-		1
R-0565	3-deoxy-D-manno-octulosonate cytidylyltransferase	3-deoxy-D-manno-octulosonate + CTP = CMP-3-deoxy-D-manno-c	CMP-KDO biosynthesis	1	1	0	0	-	-		1
R-0566	3-deoxy-8-phosphooctulonate synthase	D-arabinose-5-phosphate + phosphoenolpyruvate + H2O = 3-deo	CMP-KDO biosynthesis	1	1	0	0	-	-		1
R-0567	3-deoxy-D-manno-octulosonate-8-phosphate phospl	3-deoxy-D-manno-octulosonate-8-P + H2O = 3-deoxy-D-manno-o	CMP-KDO biosynthesis	1	1	-	-	-	-		1
R-0568	D-arabinose-5-phosphate isomerase	D-arabinose-5-phosphate = D-ribulose-5-phosphate	CMP-KDO biosynthesis	1	1	0	0	-	-		1
R-0569	salicylate hydroxylase	salicylate + NADH + O2 + 2 H+ = catechol + CO2 + NAD+ + H2O	unassigned	1	1	-1	0	-	-		1
R-0570	adenine phosphoribosyltransferase	5-phosphoribosyl-1-pyrophosphate + adenine = AMP + diphosph	salvage pathways of purine and pyri	1	1	-	-	-	-		1
R-0571	inositol monophosphatase	D-myo-inositol-3-monophosphate + H2O = phosphate + myo-inos	inositol metabolism	1	1	-	-	-	-		1
R-0572	glycerol-2-phosphatase	glycerol-2-phosphate + H2O = glycerol + phosphate	glycerol degradation	0	0	-	-	-	-		0
R-0573	thiamine phosphate synthase	4-methyl-5-(b-hydroxyethyl)thiazole-phosphate + 4-amino-5-hyd	thiamine biosynthesis	1	1	0	0	-	-		1
R-0574	thiazole synthase 1	iminoglycine + L-tyrosine + thiS-thiF-acyl-disulfide = 4-methyl-5-(t	thiamine biosynthesis	1	1	0	0	-	-		1
R-0576	glycine oxidase 1	glycine + H2O + O2 = glyoxylate + NH3 + H2O2	glycine, serine, threonine degradati	0	0	-1	0	-	-	set usable during succiante growt	0
R-0577	glycine oxidase 2	D-alanine + H2O + O2 = pyruvate + NH3 + H2O2	alanine degradation	0	0	-1	0	-	-	set usable during succiante growt	0
R-0578	glycine oxidase 3	sarcosine + H2O + O2 = glyoxylate + methylamine + H2O2	sarcosine degradation	0	0	-1	0	-	-	set usable during succiante growt	0
R-0579	glycine oxidase 4	N-ethylglycine + H2O + O2 = glyoxylate + ethylamine + H2O2	glycine, serine, threonine degradati	0	0	-1	0	-	-	set usable during succiante growt	0
R-0580	gluconolactonase	glucono-d-lactone[p] + H2O[p] = D-gluconate[p]	sugar oxidation	0	0	0	0	-	-		0
R-0581	coproporphyrinogen III oxidase	coproporphyrinogen-III + O2 = protoporphyrinogen-IX + 2 CO2 + 2	heme biosynthesis	1	1	0	0	-	-		1
R-0582	AMP nucleosidase	AMP[p] + H2O[p] = D-ribose-5-phosphate[p] + adenine[p]	salvage pathways of purine and pyri	0	0	0	0	-	-		0
R-0583	ceramide glucosyltransferase	UDP-glucose + ceramide = UDP + D-glucosyl-ceramide	unassigned	1	1	0	0	-	-		1
R-0584	7,8-diaminopelargonic acid synthetase	(S)-adenosyl-L-methionine + 7-keto-8-aminopelargonate = (S)-ade	biotin biosynthesis	1	1	0	0	-	-		1
R-0585	7-keto-8-aminopelargonic acid synthetase	6-carboxyhexanoyl-CoA + L-alanine = CO2 + 7-keto-8-aminopelarg	biotin biosynthesis	1	1	0	0	-	-		1
R-0586	biotin biosynthesis hypothetical reaction	malonyl-CoA + ADP + phosphate = ATP + 6-carboxyhexanoyl-CoA	biotin biosynthesis	1	1	-	-	-	-		1
R-0587	cobalamin synthase	adenosylcobinamide-GDP + a-ribazole = coenzyme-B12 + GMP	adenosylcobalamin biosynthesis pa	0	0	0	0	-	1		0
R-0588	cobalamin-5'-phosphate synthase	adenosylcobinamide-GDP + a-ribazole-5'-P = adenosylcobalamin-	adenosylcobalamin biosynthesis pa	1	1	0	0	-	1		1
R-0589	nicotinate-nucleotide--dimethylbenzimidazole phos	nicotinate-mononucleotide + dimethylbenzimidazole = nicotinat	adenosylcobalamin biosynthesis pa	1	1	-	-	-	-		1
R-0590	3-demethylubiquinone-8 3-O-methyltransferase	(S)-adenosyl-L-methionine + 3-demethylubiquinol-8 = (S)-adenos	ubiquinone-8 biosynthesis	1	1	0	0	-	-		1
R-0591	2-octaprenyl-6-hydroxyphenol methylase	2-octaprenyl-6-hydroxyphenol + (S)-adenosyl-L-methionine = 2-o	ubiquinone-8 biosynthesis	1	1	0	0	-	-		1
R-0593	nitric oxide dioxygenase	2 NO + NADPH + 2 O2 = 2 NO3- + NADP+ + H+	unassigned	1	1	-1	0	-	-		1
R-0594	dihydropteridine reductase	5,6,7,8-tetrahydropteridine + NADP+ = 6,7-dihydropteridine + NA	dihydrobiopterin recycling	1	1	-1	0	-	-		1
R-0595	phosphoribulokinase	D-ribulose-5-phosphate + ATP = D-ribulose-1,5-bisphosphate + AI	Calvin-Benson-Bassham cycle	1	1	0	0	-	-		1
R-0596	ornithine decarboxylase	L-ornithine = CO2 + putrescine	putrescine biosynthesis	1	1	0	0	-	-		1
R-0597	molybdopterin guanine dinucleotide synthase	molybdenum-cofactor + GTP + 4 H+ = molybdopterin-guanine-dir	molybdenum cofactor biosynthesis	1	1	0	0	-	-		1
R-0598	precorrin-6Y C(5,15)-methyltransferase	precorrin-6B + 2 (S)-adenosyl-L-methionine = precorrin-8x + 2 (S)-	adenosylcobalamin biosynthesis pa	1	1	0	0	-	-		1
R-0599	precorrin-4 C(11)-methyltransferase	(S)-adenosyl-L-methionine + precorrin-4 = (S)-adenosyl-L-homocy	adenosylcobalamin biosynthesis pa	1	1	-	-	-	-		1
R-0600	cobyrinic acid a,c-diamide synthase	cobyrinate + 2 L-glutamine + 2 ATP + 2 H2O = cob(II)yrinate-a,c-di	adenosylcobalamin biosynthesis pa	0	0	0	0	-	1		0
R-0601	hydrogenobyrinate a,c-diamide synthase	hydrogenobyrinate + 2 L-glutamine + 2 ATP + 2 H2O = 2 L-glutama	adenosylcobalamin biosynthesis pa	1	1	0	0	-	1		1
R-0602	isonitrile hydratase	N-cyclohexylformamide = cyclohexyl-isocyanide + H2O	unassigned	1	1	0	0	-	-		1
R-0603	urease	urea + H2O = 2 NH3 + CO2	urea degradation	0	0	0	0	-	-		0
R-0604	6-pyruvoyl tetrahydrobiopterin synthase	7,8-dihydroneopterin-triphosphate = triphosphate + 6-pyruvoyl-t	tetrahydrobioprein biosynthesis	1	1	-1	0	-	-		1
R-0605	quinoprotein ethanol dehydrogenase	ethanol[p] + 2 oxidized-cytochrome-c = 2 reduced-cytochrome-c	oxidative ethanol degradation	0	0	0	0	-	-		0
R-0606	cytochrome d terminal oxidase	2 UQH2 + O2 + 4 H+ = 2 UQ + 2 H2O + 4 H+[p]	oxidative phosphorilation	1	1	1	0	-	-		1
R-0607	2-octaprenylphenol hydroxylase	2-octaprenylphenol + O2 + 2 H+ = 2-octaprenyl-6-hydroxyphenol	ubiquinone-8 biosynthesis	1	1	0	0	-	-		1
R-0608	NADPH-dependent FMN reductase	FMNH2 + NADP+ = FMN + NADPH + H+	oxidative phosphorilation	1	1	0	0	-	-		1

R-0609	precorrin-3B C(17)-methyltransferase	precorrin-3B + (S)-adenosyl-L-methionine = precorrin-4 + (S)-aden	adenosylcobalamin biosynthesis pa	1	1	0	0	-	-	1
R-0610	precorrin-2 C(20)-methyltransferase	precorrin-2 + (S)-adenosyl-L-methionine = (S)-adenosyl-L-homocy	adenosylcobalamin biosynthesis pa	1	1	0	0	-	-	1
R-0611	precorrin-8X methylmutase	precorrin-8x = hydrogenobyrinate	adenosylcobalamin biosynthesis pa	1	1	0	0	-	1	1
R-0612	precorrin-3B synthase	precorrin-3A + NADH + O2 + H+ = precorrin-3B + NAD+ + H2O	adenosylcobalamin biosynthesis pa	1	1	0	0	-	-	1
R-0613	cobinamide phosphate guanylyltransferase	adenosyl-cobinamide-phosphate + GTP = adenosylcobinamide-G	adenosylcobalamin biosynthesis pa	1	1	-	-	-	-	1
R-0614	cobinamide kinase	adenosylcobinamide + ATP = adenosyl-cobinamide-phosphate +	adenosylcobalamin salvage pathwa	0	0	-	-	-	-	0
R-0615	cobalamin adenosyltransferase	ATP + cob(I)alamin = coenzyme-B12 + triphosphate + H+	adenosylcobalamin salvage pathwa	0	0	0	0	-	-	0
R-0616	ferrochelatase	Fe2+ + protoporphyrin-IX = protoheme-IX + 2 H+	heme biosynthesis	1	1	0	0	-	-	1
R-0617	dephospho-CoA kinase	dephospho-CoA + ATP = ADP + CoA	coenzyme A biosynthesis	1	1	0	0	-	-	1
R-0618	uroporphyrinogen decarboxylase	uroporphyrinogen-III = 4 CO2 + coproporphyrinogen-III	heme biosynthesis	1	1	0	0	-	-	1
R-0619	molybdopterin synthase sulfurylase	precursor-Z + 2 sulfurated-molybdopterin-synthase = 2 molybdop	molybdenum cofactor biosynthesis	1	1	0	0	-	-	1
R-0620	ETF dehydrogenase	reduced-ETF + UQ = oxidized-ETF + UQH2	oxidative phosphorilation	1	1	0	0	-	-	1
R-0621	3-hydroxybutyryl-CoA dehydrogenase	(S)-3-hydroxybutanoyl-CoA + NADP+ = acetoacetyl-CoA + NADPH	Ethylmalonyl-CoA pathway	0	0	0	0	-	1	0
R-0622	uracil phosphoribosyltransferase	diphosphate + UMP = 5-phosphoribosyl-1-pyrophosphate + uraci	salvage pathways of purine and pyri	0	0	0	0	-	-	0
R-0623	glycogen phosphorylase-limit dextrin a-1,6-glucohyd	limit-dextrin + H2O = maltotetraose + debranched-limit-dextrin	starch and sucrose metabolism	1	1	0	0	-	-	1
R-0624	thiamine biosynthesis protein thiC	5-aminoimidazole-ribonucleotide + (S)-adenosyl-L-methionine =	thiamine biosynthesis	1	1	-1	0	-	-	1
R-0625	guanine deaminase	guanine + H2O = NH3 + xanthine	salvage pathways of purine and pyri	0	0	0	0	-	-	0
R-0626	ureidoglycolate amidohydrolase	(S)-ureidoglycolate + H2O = 2 NH3 + CO2 + glyoxylate	allantoin degradation pathway	0	0	-	0	-	-	0
R-0627	3-methyl-2-oxobutanoate hydroxymethyltransferase	3-methyl-2-oxobutanoate + 5,10-methylenetetrahydrofolate + H2	pantothenate biosynthesis	1	1	-1	0	-	-	1
R-0628	carboxymethylenebutenolidase	2-chloro-cis-dienelactone + H2O = 2-chloromaleylacetate + H+	1,4-dichlorobenzene degradation	0	0	0	0	-	-	0
R-0629	dienelactone hydrolase (cis)	cis-dienelactone + H2O = 2-maleylacetate + H+	4-chlorocatechol degradation	0	0	0	0	-	-	0
R-0630	dienelactone hydrolase (trans)	trans-dienelactone + H2O = 2-maleylacetate + H+	4-chlorocatechol degradation	0	0	0	0	-	-	0
R-0631	4-(b-D-ribofuranosyl)aminobenzene-5'-phosphate sy	p-aminobenzoate + 5-phosphoribosyl-1-pyrophosphate = 4-(b-D-	folate biosynthesis	1	1	0	0	-	-	1
R-0632	acyl carrier protein phosphodiesterase	[acyl-carrier-protein] + H2O = 4'-phosphopantetheine + apo-[acyl	acyl carrier protein metabolism	0	0	-	-	-	-	0
R-0633	homospermidine synthase 1	putrescine + spermidine = 1,3-diaminopropane + sym-homosperr	homospermidine biosynthesis	1	1	0	0	-	-	1
R-0634	homospermidine synthase 2	2 putrescine = NH3 + sym-homospermidine	homospermidine biosynthesis	1	1	0	0	-	-	1
R-0635	N-ethylmaleimide reductase	N-ethylmaleimide + 2 H+ = N-ethylsuccinimide	unassigned	1	1	0	0	-	-	1
R-0636	inorganic pyrophosphatase	diphosphate + H2O = 2 phosphate	oxidative phosphorilation	1	1	0	0	-	1	1
R-0637	UDP-3-O-[3-hydroxymyristoyl]glucosamine N-acetylt	(3R)-3-hydroxytetradecanoyl-[acyl-carrier-protein] + UDP-3-O-(3-I	lipid-A-precursor biosynthesis	1	1	0	0	-	-	1
R-0638	undecaprenyl diphosphate synthase 1	all-trans-farnesyl-diphosphate + isopentenyl-diphosphate = 2-cis,	peptidoglycan biosynthesis	1	1	0	0	-	-	1
R-0639	undecaprenyl diphosphate synthase 2	2-cis,6-trans,10-trans-geranylgeranyl-diphosphate + isopentenyl-	peptidoglycan biosynthesis	1	1	0	0	-	-	1
R-0640	undecaprenyl diphosphate synthase 3	di-trans,poly-cis-pentaprenyl-diphosphate + isopentenyl-diphosp	peptidoglycan biosynthesis	1	1	0	0	-	-	1
R-0641	undecaprenyl diphosphate synthase 4	di-trans,poly-cis-hexaprenyl-diphosphate + isopentenyl-diphosph	peptidoglycan biosynthesis	1	1	0	0	-	-	1
R-0642	undecaprenyl diphosphate synthase 5	di-trans,poly-cis-heptaprenyl-diphosphate + isopentenyl-diphosp	peptidoglycan biosynthesis	1	1	0	0	-	-	1
R-0643	undecaprenyl diphosphate synthase 6	di-trans,poly-cis-octaprenyl-diphosphate + isopentenyl-diphosph	peptidoglycan biosynthesis	1	1	0	0	-	-	1
R-0644	undecaprenyl diphosphate synthase 7	di-trans,poly-cis-nonaprenyldiphosphate + isopentenyl-diphosph	peptidoglycan biosynthesis	1	1	0	0	-	-	1
R-0645	undecaprenyl diphosphate synthase 8	di-trans,poly-cis-decaprenyldiphosphate + isopentenyl-diphosph	peptidoglycan biosynthesis	1	1	0	0	-	-	1
R-0646	acyl-CoA thioesterase II (n-h6:0)	(3R)-3-hydroxyhexanoyl-CoA + H2O = (3R)-3-hydroxyhexanoate +	fatty acid b-oxidation	0	0	0	0	-	-	0
R-0647	acyl-CoA thioesterase II (n-h8:0)	(3R)-3-hydroxyoctanoyl-CoA + H2O = (3R)-3-hydroxyoctanoate + (	fatty acid b-oxidation	0	0	0	0	-	-	0
R-0648	acyl-CoA thioesterase II (n-h10:0)	(3R)-3-hydroxydecanoyl-CoA + H2O = (3R)-3-hydroxydecanoate +	fatty acid b-oxidation	0	0	0	0	-	-	0
R-0649	acyl-CoA thioesterase II (n-h12:0)	(3R)-3-hydroxydodecanoyl-CoA + H2O = (3R)-3-hydroxydodecano	fatty acid b-oxidation	0	0	0	0	-	-	0
R-0650	acyl-CoA thioesterase II (n-h14:0)	(3R)-3-hydroxytetradecanoyl-CoA + H2O = (3R)-3-hydroxytetrade	fatty acid b-oxidation	0	0	0	0	-	-	0
R-0651	acyl-CoA thioesterase II (n-h16:0)	(3R)-3-hydroxyhexadecanoyl-CoA + H2O = (3R)-3-hydroxyhexadec	fatty acid b-oxidation	0	0	0	0	-	-	0
R-0652	acyl-CoA thioesterase II (n-h18:0)	(3R)-3-hydroxyoctadecanoyl-CoA + H2O = (3R)-3-hydroxyoctadeca	fatty acid b-oxidation	0	0	0	0	-	-	0
R-0653	acyl-CoA thioesterase II (n-6:0)	hexanoyl-CoA + H2O = hexanoate + CoA	fatty acid b-oxidation	0	0	0	0	-	-	0
R-0654	acyl-CoA thioesterase II (n-8:0)	octanoyl-CoA + H2O = octanoate + CoA	fatty acid b-oxidation	0	0	0	0	-	-	0
R-0655	acyl-CoA thioesterase II (n-10:0)	decanoyl-CoA + H2O = decanoate + CoA	fatty acid b-oxidation	0	0	0	0	-	-	0
R-0656	acyl-CoA thioesterase II (n-12:0)	dodecanoyl-CoA + H2O = dodecanoate + CoA	fatty acid b-oxidation	0	0	0	0	-	-	0
R-0657	acyl-CoA thioesterase II (n-14:0)	tetradecanoyl-CoA + H2O = tetradecanoate + CoA	fatty acid b-oxidation	0	0	0	0	-	-	0
R-0658	acyl-CoA thioesterase II (n-16:0)	hexadecanoyl-CoA + H2O = hexadecanoate + CoA	fatty acid b-oxidation	0	0	0	0	-	-	0
R-0659	acyl-CoA thioesterase II (n-18:0)	octadecanoyl-CoA + H2O = octadecanoate + CoA	fatty acid b-oxidation	0	0	0	0	-	-	0
R-0660	creatininase	creatinine + H2O = creatine	creatinine degradation	0	0	0	0	-	-	0
R-0661	fumarylacetoacetate hydrolase	4-fumaryl-acetoacetate + H2O = fumarate + acetoacetate	tyrosine degradation	0	0	1	0	-	-	0
R-0662	2-dehydropantoate 2-reductase	2-dehydropantoate + NADPH + H+ = L-pantoate + NADP+	pantothenate biosynthesis	1	1	1	0	-	-	1
R-0663	sulfate adenylyltransferase	SO42- + ATP + 2 H+ = adenosine-5'-phosphosulfate + diphosphate	sulfate reduction I (assimilatory)	1	1	0	0	-	-	1
R-0664	phosphoadenosine phosphosulfate reductase	phosphoadenosine-5'-phosphosulfate + reduced-thioredoxin = a	sulfate reduction I (assimilatory)	1	1	0	0	-	-	1
R-0665	sulfite reductase-(NADPH)	HSO3- + 3 NADPH + 4 H+ = H2S + 3 NADP+ + 3 H2O	sulfate reduction I (assimilatory)	1	1	0	0	-	-	1
R-0666	pyridoxine 5'-phosphate synthase	1-deoxy-D-xylulose-5-phosphate + 1-amino-propan-2-one-3-phos	pyridoxal-5'-phosphate biosynthesis	1	1	0	0	-	-	1
R-0667	holo-[acyl-carrier-protein] synthase	apo-[acyl-carrier-protein] + CoA = adenosine-3',5'-bisphosphate +	acyl carrier protein metabolism	1	1	0	0	-	-	1
R-0669	glutathione reductase	glutathione-disulfide + NADPH + H+ = 2 glutathione + NADP+	superoxide radicals degradation	0	0	0	0	-	-	0
R-0670	NADPH-ferredoxin oxidoreductase 1	2 oxidized-ferredoxin + NADPH = 2 reduced-ferredoxin + NADP+ +	oxidative phosphorilation	1	1	0	0	-	-	1
R-0671	dethiobiotin synthetase	CO2 + 7,8-diaminopelargonate + ATP = dethiobiotin + phosphate	biotin biosynthesis	1	1	0	1	-	-	1
R-0672	undecaprenyl pyrophosphate phosphatase	di-trans,poly-cis-undecaprenyl-diphosphate + H2O = di-trans,pol	peptidoglycan biosynthesis	1	1	-	0	-	-	1
R-0673	peptidoglycan glycosyltransferase	diphosphoundecaprenyl-[GlcNAc-MurNAc-pentapeptide](n) + pe	peptidoglycan biosynthesis	1	1	0	0	-	-	1
R-0674	pantothenate kinase	(R)-pantothenate + ATP = (D)-4'-phosphopantothenate + ADP	coenzyme A biosynthesis	1	1	-1	0	-	-	1
R-0675	methylamine dehydrogenase	methylamine[p] + oxidized-amicyanin[p] + H2O[p] = formaldehyd	methylamine oxidation to formalde	1	1	1	0	-	-	1
R-0676	maleylacetoacetate isomerase	4-maleyl-acetoacetate = 4-fumaryl-acetoacetate	tyrosine degradation	0	0	0	0	-	-	0
R-0677	NADH:ubiquinone oxidoreductase II	UQ + NADH + H+ = UQH2 + NAD+	oxidative phosphorilation	1	1	0	0	-	-	1
R-0679	pyridoxal kinase	ATP + pyridoxal = ADP + pyridoxal-5'-phosphate	pyridoxal-5'-phosphate salvage path	0	0	0	0	-	-	0
R-0680	pyridoxamine kinase	ATP + pyridoxamine = ADP + pyridoxamine-5'-phosphate	pyridoxal-5'-phosphate salvage path	0	0	0	0	-	-	0
R-0681	trans-2,3-dihydro-3-hydroxy-anthranilate isomerase	trans-2,3-dihydro-3-hydroxyanthranilate = 2,3-dihydro-3-hydroxy	phenazine biosynthesis	1	1	0	0	-	-	1
R-0682	L-Ala-D/L-Glu epimerase	L-Ala-γ-D-Glu = L-Ala-L-Glu	unassigned	1	1	-	-	-	-	1
R-0683	spermidine acetyltransferase	acetyl-CoA + spermidine = N-acetylspermidine + CoA	spermidine metabolism	1	1	0	0	-	-	1
R-0684	methoxyneurosporene dehydrogenase 1	hydroxyneurosporene + oxidized-electron-acceptor = demethylsp	carotenoids biosynthesis	1	1	0	1	-	-	1
R-0685	methoxyneurosporene dehydrogenase 2	rhodopin + oxidized-electron-acceptor = 3,4-dehydrorhodopin +	carotenoids biosynthesis	1	1	0	1	-	-	1
R-0686	methoxyneurosporene dehydrogenase 3	rhodovibrin + oxidized-electron-acceptor = hydroxyspirilloxanthi	carotenoids biosynthesis	1	1	0	1	-	-	1
R-0687	hydroxyneurosporene methyltransferase 1	demethylspheroidene + (S)-adenosyl-L-methionine = spheroiden	carotenoids biosynthesis	1	1	0	0	-	-	1
R-0688	hydroxyneurosporene methyltransferase 2	hydroxyneurosporene + (S)-adenosyl-L-methionine = methoxyne	carotenoids biosynthesis	1	1	0	0	-	-	1
R-0689	hydroxyneurosporene methyltransferase 3	rhodopin + (S)-adenosyl-L-methionine = 3,4-dihydroanhydrorhod	carotenoids biosynthesis	1	1	0	0	-	-	1
R-0690	hydroxyneurosporene methyltransferase 4	3',4'-dihydrorhodovibrin + (S)-adenosyl-L-methionine = 3,4,3',4'-t	carotenoids biosynthesis	1	1	0	0	-	-	1
R-0691	hydroxyneurosporene methyltransferase 5	3,4-dehydrorhodopin + (S)-adenosyl-L-methionine = anhydrorho	carotenoids biosynthesis	1	1	0	0	-	-	1
R-0692	hydroxyneurosporene methyltransferase 6	hydroxyspirilloxanthin + (S)-adenosyl-L-methionine = spirilloxant	carotenoids biosynthesis	1	1	0	0	-	-	1
R-0693	2-desacetyl-2-hydroxyethyl bacteriochlorophyllide a	2-desacetyl-2-hydroxyethyl-bacteriochlorophyllidea = bacterioch	bacteriochlorophyll a biosynthesis	1	1	0	1	-	-	1
R-0694	chlorophyllide a reductase	chlorophyllidea + ATP + H2O = 3-vinyl-bacteriochlorophyllidea +	bacteriochlorophyll a biosynthesis	1	1	1	0	-	-	1
R-0695	3-hydroxyethylchlorophyllide a reductase	3-hydroxyethylchlorophyllidea + 2 H+ = 2-desacetyl-2-hydroxyeth	bacteriochlorophyll a biosynthesis	1	1	1	0	-	-	1
R-0696	cyanase	cyanate + HCO3- + H+ = carbamate + CO2	cyanate degradation	0	0	0	0	-	-	0
R-0697	di-haem cytochrome c peroxidase	H2O2[p] + 2 reduced-cytochrome-c + 2 H+ = 2 oxidized-cytochrom	superoxide radicals degradation	0	0	1	0	-	-	0
R-0698	L-sorbosone dehydrogenase	L-sorbosone-2,6-lactone + reduced-electron-acceptor + H2O = 2-k	sugar metabolism	1	1	0	0	-	-	1
R-0699	xanthine dehydrogenase 1	xanthine + NAD+ + H2O = urate + NADH + H+	salvage pathways of purine and pyri	0	0	0	0	-	-	0
R-0700	carbonic anhydrase	CO2 + H2O = HCO3- + H+	unassigned	1	1	1	0	-	-	1
R-0701	molybdenum::molybdopterin ligase	molybdopterin-AMP + molybdate + H+ = molybdenum-cofactor +	molybdenum cofactor biosynthesis	1	1	0	0	-	-	1
R-0702	nicotinate nucleotide adenylyltransferase	ATP + nicotinate-mononucleotide = diphosphate + nicotinate-ad	NAD biosynthesis	1	1	-	0	-	-	1
R-0703	acetyl-CoA:acetoacetyl-CoA transferase	acetoacetate + acetyl-CoA = acetoacetyl-CoA + acetate	PHB degradation	0	0	0	0	-	1	0
R-0704	UDP-N-acetylglucosamine--N-acetylmuramyl-(penta	N-acetylmuramoyl-pentapeptide-diphosphoundecaprenol + UDP	peptidoglycan biosynthesis	1	1	-	-	-	-	1
R-0705	UDP-N-acetylglucosamine--N-acetylmuramyl-(penta	N-acetylmuramoyl-L-alanyl-D-glutamyl-meso-2,6-diaminopimely	peptidoglycan biosynthesis	1	1	-	-	-	-	1
R-0706	UDP-N-acetylmuramate-alanine ligase	L-alanine + UDP-N-acetylmuramate + ATP = UDP-N-acetylmuram	peptidoglycan biosynthesis	1	1	0	0	-	-	1
R-0707	D-alanine-D-alanine ligase B	2 D-alanine + ATP = D-alanyl-D-alanine + phosphate + ADP	peptidoglycan biosynthesis	1	1	0	0	-	-	1
R-0708	UDP-3-O-acyl-N-acetylglucosamine deacetylase	UDP-3-O-(3-hydroxymyristoyl)-N-acetylglucosamine + H2O = UDP	lipid-A-precursor biosynthesis	1	1	0	0	-	-	1
R-1033	adenosine phosphorylase	phosphate + adenosine = deoxyribose-1-phosphate + adenine	salvage pathways of purine and pyri	1	1	-	-	-	-	1
R-0709	thymidine phosphorylase	phosphate + thymidine = deoxyribose-1-phosphate + thymine	salvage pathways of purine and pyri	0	0	0	0	-	-	0
R-0710	uracil phosphorylase	phosphate + deoxyuridine = deoxyribose-1-phosphate + uracil	salvage pathways of purine and pyri	0	0	0	0	-	-	0
R-0711	xanthosine phosphorylase	xanthosine + phosphate = alpha-D-ribose-1-phosphate + xanthin	salvage pathways of purine and pyri	0	0	-	-	-	-	0
R-0712	cytidine deaminase 1	cytidine + H2O = uridine + NH3	salvage pathways of purine and pyri	0	0	-	-	-	-	0
R-0713	cytidine deaminase 2	deoxycytidine + H2O = deoxyuridine + NH3	salvage pathways of purine and pyri	0	0	-	-	-	-	0
R-0714	tetraacyldisaccharide 4'-kinase	lipid-A-disaccharide + ATP = lipid-IVA + ADP	lipid-A-precursor biosynthesis	1	1	-	-	-	-	1
R-0715	3-deoxy-D-manno-octulosonic acid transferase 1	(KDO)-lipid-IVA + CMP-3-deoxy-D-manno-octulosonate = KDO2-li	lipopolysaccharide biosynthesis	1	1	0	0	-	-	1
R-0716	3-deoxy-D-manno-octulosonic acid transferase 2	lipid-IVA + CMP-3-deoxy-D-manno-octulosonate = (KDO)-lipid-IV	lipopolysaccharide biosynthesis	1	1	0	0	-	-	1
R-0717	GTP cyclohydrolase II	GTP + 3 H2O = diphosphate + 2,5-diamino-6-(ribosylamino)-4-(3H	flavin biosynthesis	1	1	0	0	-	-	1
R-0718	acylphosphatase (n-16:0)	hexadecanoyl-phosphate + H2O = hexadecanoate + phosphate	fatty acid degradation	0	0	-	-	-	-	0
R-0719	acylphosphatase (n-16:1)	hexadec-9-enoyl-phosphate + H2O = 9-hexadecenoate + phospha	fatty acid degradation	0	0	-	-	-	-	0
R-0720	acylphosphatase (n-18:0)	octadecanoyl-phosphate + H2O = octadecanoate + phosphate	fatty acid degradation	0	0	-	-	-	-	0
R-0721	acylphosphatase (n-18:1)	octadec-9-enoyl-phosphate + H2O = 9-octadecenoate + phosphat	fatty acid degradation	0	0	-	-	-	-	0
R-0722	magnesium chelatase	ATP + protoporphyrin-IX + Mg2+ + H2O = Mg-protoporphyrin + ph	bacteriochlorophyll a biosynthesis	1	1	0	0	-	-	1
R-0724	H(+)-translocating pyrophosphatases	diphosphate + H2O + H+ = 2 phosphate + H+[p]	oxidative phosphorilation	1	1	0	0	-	1	1
R-0725	octaprenyl diphosphate synthase 1	isopentenyl-diphosphate + all-trans-geranyl-geranyl-diphosphate	ubiquinone-8 biosynthesis	1	1	0	0	-	-	1
R-0726	octaprenyl diphosphate synthase 2	geranylfarnesyl-diphosphate + isopentenyl-diphosphate = all-tra	ubiquinone-8 biosynthesis	1	1	0	0	-	-	1
R-0727	octaprenyl diphosphate synthase 3	all-trans-hexaprenyl-diphosphate + isopentenyl-diphosphate = al	ubiquinone-8 biosynthesis	1	1	0	0	-	-	1
R-0728	octaprenyl diphosphate synthase 4	all-trans-heptaprenyl-diphosphate + isopentenyl-diphosphate =	ubiquinone-8 biosynthesis	1	1	0	0	-	-	1
R-0729	thiamine monophosphate kinase	thiamine-phosphate + ATP = thiamine-diphosphate + ADP	thiamin biosynthesis	1	1	0	0	-	-	1
R-0730	6,7-dimethyl-8-ribityllumazine synthase	5-amino-6-ribitylamino-2,4(1H,3H)-pyrimidinedione + L-3,4-dihyc	flavin biosynthesis	1	1	0	0	-	-	1
R-0731	riboflavin synthase	2 6,7-dimethyl-8-(1-D-ribityl)lumazine = 5-amino-6-ribitylamino-2	flavin biosynthesis	1	1	0	0	-	-	1
R-0732	diaminohydroxyphosphoribosylaminopyrimidine de	2,5-diamino-6-(ribosylamino)-4-(3H)-pyrimidinone-5'-phosphate	flavin biosynthesis	1	1	0	0	-	-	1
R-0733	5-amino-6-(5-phosphoribosylamino)uracil reductase	5-amino-6-(5'-phosphoribosylamino)uracil + NADPH + H+ = 5-ami	flavin biosynthesis	1	1	0	0	-	-	1
R-0736	hydroperoxidase	2 H2O2 = 2 H2O + O2	superoxide radicals degradation	1	1	0	0	-	-	1
R-0737	heme d synthase	protoheme-IX + H2O2 = heme-d	heme biosynthesis	1	1	0	0	-	-	1
R-0738	nitrate reductase (periplasmic)	NO3-[p] + 2 reduced-cytochrome-c + 2 H+[p] = NO2-[p] + 2 oxidize	oxidative phosphorilation	0	0	0	0	-	-	0
R-0739	uroporphyrin III C-methyltransferase 2	(S)-adenosyl-L-methionine + precorrin-1 = (S)-adenosyl-L-homocy	siroheme biosynthesis	1	1	0	0	-	-	1
R-0740	uroporphyrin III C-methyltransferase 1	(S)-adenosyl-L-methionine + uroporphyrinogen-III = (S)-adenosyl-	siroheme biosynthesis	1	1	0	0	-	-	1
R-0741	precorrin-2 dehydrogenase	precorrin-2 + NAD+ = sirohydrochlorin + NADH + H+	siroheme biosynthesis	1	1	0	0	-	-	1
R-0742	sirohydrochlorin ferrochelatase	sirohydrochlorin + Fe2+ = siroheme + 2 H+	siroheme biosynthesis	1	1	0	0	-	-	1
R-0743	cobaltochelatase	hydrogenobyrinate-a,c-diamide + Co2+ + ATP + H2O = cob(II)yrin	adenosylcobalamin biosynthesis pa	1	1	0	0	-	-	1
R-0744	acyl-CoA dehydrogenase (n-4:0)	butyryl-CoA + FAD = crotonyl-CoA + FADH2	fatty acid b-oxidation	0	0	0	0	-	-	0
R-0745	acyl-CoA dehydrogenase (n-6:0)	hexanoyl-CoA + FAD = trans-hex-2-enoyl-CoA + FADH2	fatty acid b-oxidation	0	0	0	0	-	-	0
R-0746	acyl-CoA dehydrogenase (n-8:0)	octanoyl-CoA + FAD = trans-oct-2-enoyl-CoA + FADH2	fatty acid b-oxidation	0	0	0	0	-	-	0
R-0747	acyl-CoA dehydrogenase (n-10:0)	decanoyl-CoA + FAD = trans-dec-2-enoyl-CoA + FADH2	fatty acid b-oxidation	0	0	0	0	-	-	0
R-0748	acyl-CoA dehydrogenase (n-12:0)	dodecanoyl-CoA + FAD = trans-dodec-2-enoyl-CoA + FADH2	fatty acid b-oxidation	0	0	0	0	-	-	0
R-0749	acyl-CoA dehydrogenase (n-14:0)	tetradecanoyl-CoA + FAD = trans-tetradec-2-enoyl-CoA + FADH2	fatty acid b-oxidation	0	0	0	0	-	-	0
R-0750	acyl-CoA dehydrogenase (n-16:0)	hexadecanoyl-CoA + FAD = trans-hexadec-2-enoyl-CoA + FADH2	fatty acid b-oxidation	0	0	0	0	-	-	0
R-0751	acyl-CoA dehydrogenase (n-18:0)	octadecanoyl-CoA + FAD = trans-octadec-2-enoyl-CoA + FADH2	fatty acid b-oxidation	0	0	0	0	-	-	0
R-0752	acyl-CoA dehydrogenase (n-12:1)	cis-dodec-5-enoyl-CoA + FAD = trans-3-cis-5-dodecenoyl-CoA + FA	fatty acid b-oxidation	0	0	0	0	-	-	0
R-0753	acyl-CoA dehydrogenase (n-14:1)	cis-tetradec-7-enoyl-CoA + FAD = trans-3-cis-7-tetradecenoyl-CoA	fatty acid b-oxidation	0	0	0	0	-	-	0
R-0754	acyl-CoA dehydrogenase (n-16:1)	hexadec-9-enoyl-CoA + FAD = trans-3-cis-9-hexadecenoyl-CoA + F	fatty acid b-oxidation	0	0	0	0	-	-	0
R-0755	acyl-CoA dehydrogenase (n-18:1)	octadec-9-enoyl-CoA + FAD = trans-3-cis-11-octadecenoyl-CoA + F	fatty acid b-oxidation	0	0	0	0	-	-	0
R-0756	heme O synthase	protoheme-IX + all-trans-farnesyl-diphosphate + H2O = heme-o +	heme biosynthesis	1	1	-	0	-	-	1
R-0757	N-formylglutamate amidohydrolase	N-formyl-L-glutamate + H2O = L-glutamate + formate	histidine degradation	0	0	0	0	-	-	0
R-0758	haloacid dehalogenase	chloroacetate + H2O = glycolate + HCl	1,2-dichloroethane degradation	0	0	0	0	-	-	0
R-0759	superoxide dismutase	2 superoxide + 2 H+ = H2O2 + O2	superoxide radicals degradation	0	0	0	0	-	-	0
R-0760	sulfite oxidase	HSO3- + O2 + H2O = H2O2 + SO42- + H+	sulfur metabolism	0	0	0	0	-	-	0
R-0762	2-hydroxychromene-2-carboxylate isomerase	2-hydroxychromene-2-carboxylate = trans-O-hydroxybenzylidene	naphthalene degradation	0	0	0	0	-	-	0
R-0763	precorrin-5 (C1)-methyltransferase	precorrin-5 + (S)-adenosyl-L-methionine + H2O = precorrin-6A + (S	adenosylcobalamin biosynthesis pa	1	1	0	0	-	-	1
R-0764	fatty acyl-CoA synthetase (n-4:0)	butyrate + ATP + CoA = butyryl-CoA + AMP + diphosphate	fatty acid b-oxidation	0	0	0	0	-	-	0
R-0765	fatty acyl-CoA synthetase (n-6:0)	hexanoate + ATP + CoA = hexanoyl-CoA + AMP + diphosphate	fatty acid b-oxidation	0	0	0	0	-	-	0
R-0766	fatty acyl-CoA synthetase (n-8:0)	octanoate + ATP + CoA = octanoyl-CoA + AMP + diphosphate	fatty acid b-oxidation	0	0	0	0	-	-	0
R-0767	fatty acyl-CoA synthetase (n-10:0)	decanoate + ATP + CoA = decanoyl-CoA + AMP + diphosphate	fatty acid b-oxidation	0	0	0	0	-	-	0
R-0768	fatty acyl-CoA synthetase (n-12:0)	dodecanoate + ATP + CoA = dodecanoyl-CoA + AMP + diphosphat	fatty acid b-oxidation	0	0	0	0	-	-	0
R-0769	fatty acyl-CoA synthetase (n-14:0)	tetradecanoate + ATP + CoA = tetradecanoyl-CoA + AMP + diphos	fatty acid b-oxidation	0	0	0	0	-	-	0
R-0770	fatty acyl-CoA synthetase (n-16:0)	hexadecanoate + ATP + CoA = hexadecanoyl-CoA + AMP + diphos	fatty acid b-oxidation	0	0	0	0	-	-	0
R-0771	fatty acyl-CoA synthetase (n-18:0)	octadecanoate + ATP + CoA = octadecanoyl-CoA + AMP + diphos	fatty acid b-oxidation	0	0	0	0	-	-	0
R-0772	fatty acyl-CoA synthetase (n-12:1)	5-dodecenoate + ATP + CoA = cis-dodec-5-enoyl-CoA + AMP + dip	fatty acid b-oxidation	0	0	0	0	-	-	0
R-0773	fatty acyl-CoA synthetase (n-14:1)	7-tetradecenoate + ATP + CoA = cis-tetradec-7-enoyl-CoA + AMP +	fatty acid b-oxidation	0	0	0	0	-	-	0
R-0774	fatty acyl-CoA synthetase (n-16:1)	9-hexadecenoate + ATP + CoA = hexadec-9-enoyl-CoA + AMP + di	fatty acid b-oxidation	0	0	0	0	-	-	0
R-0775	fatty acyl-CoA synthetase (n-18:1)	9-octadecenoate + ATP + CoA = octadec-9-enoyl-CoA + AMP + dip	fatty acid b-oxidation	0	0	0	0	-	-	0
R-0776	adenine deaminase	adenine + H2O = NH3 + hypoxanthine	salvage pathways of purine and pyri	1	1	0	0	-	-	1
R-0777	exopolyphosphatase	polyphosphate(n) + H2O = polyphosphate(n-1) + phosphate	phosphorous metabolism	0	0	0	0	-	-	0
R-0778	lauroyl acyltransferase	dodecanoyl-[acyl-carrier-protein] + KDO2-lipid-IVA = KDO2-(lauro	lipopolysaccharide biosynthesis	1	1	0	0	-	-	1
R-0779	alcohol dehydrogenase (isopropanol)	isopropanol + NAD+ = acetone + NADH + H+	acetone degradation	0	0	0	0	-	-	0
R-0780	alcohol dehydrogenase (acetaldehyde)	acetaldehyde + NADH + H+ = ethanol + NAD+	oxidative ethanol degradation	0	0	0	0	-	-	0
R-0781	acetoin reductase	acetoin + NADH + H+ = 2,3-butanediol + NAD+	diacetyl, acetoin, 2,3-butanediol bic	0	0	0	0	-	-	0
R-0782	diacetyl reductase	diacetyl + NADH + H+ = acetoin + NAD+	diacetyl, acetoin, 2,3-butanediol bic	0	0	0	0	-	-	0
R-0783	3,4-dihydroxy-2-butanone-4-phoshate synthase	D-ribulose-5-phosphate = formate + L-3,4-dihydroxybutan-2-one-	flavin biosynthesis	1	1	0	0	-	-	1
R-0784	pyrimidine phosphatase	5-amino-6-ribitylamino-2,4(1H,3H)-pyrimidinedione-5'-phosphat	flavin biosynthesis	1	1	-	-	-	-	1
R-0785	2,3-dihydro-3-hydroxy-anthranilate oxidation (spont	2,3-dihydro-3-hydroxy-anthranilate = 2,3-dihydro-3-oxo-anthrani	phenazine biosynthesis	1	1	-	-	-	-	1
R-0786	2,3-dihydro-3-oxo-anthranilate condensation (hypot	2 2,3-dihydro-3-oxo-anthranilate = phenazine-1-carboxylate + CO	phenazine biosynthesis	1	1	-	-	-	-	1
R-0787	NH3-dependent NAD(+) synthetase	ATP + nicotinate-adenine-dinucleotide + NH3 = AMP + diphospha	NAD biosynthesis	1	1	0	0	-	-	1
R-0788	anhydro-N-acetylmuramoyl-L-alanine amidase	GlcNAc-1,6-anhMurNAc-L-Ala-gamma-D-Glu-DAP-D-Ala + H2O = (	murein tripeptide recycling	0	0	0	0	-	-	0
R-0789	4-hydroxybenzoate octaprenyltransferase	all-trans-octaprenyl-diphosphate + 4-hydroxybenzoate = 3-octapi	ubiquinone-8 biosynthesis	1	1	0	0	-	-	1
R-0790	chorismate lyase	chorismate = 4-hydroxybenzoate + pyruvate	ubiquinone-8 biosynthesis	1	1	-	-	-	-	1
R-0791	3-octaprenyl-4-hydroxybenzoate carboxy-lyase	3-octaprenyl-4-hydroxybenzoate = 2-octaprenylphenol + CO2	ubiquinone-8 biosynthesis	1	1	-	-	-	-	1
R-0792	molybderin biosynthesis protein	GTP + H2O = precursor-Z + diphosphate	molybdenum cofactor biosynthesis	1	1	0	0	-	-	1
R-0793	4-hydroxy-L-threonine phosphate dehydrogenase, N	4-(phosphonooxy)-threonine + NAD+ = (2S)-2-amino-3-oxo-4-pho	pyridoxal-5'-phosphate biosynthesis	1	1	0	0	-	-	1
R-0794	xanthine dehydrogenase 2	hypoxanthine + H2O = xanthine + 2 H+	salvage pathways of purine and pyri	0	0	0	0	-	-	0
R-0795	urea carboxylase	ATP + urea + CO2 + H2O = ADP + phosphate + urea-1-carboxylate	urea degradation	0	0	-	-	-	-	0
R-0796	allophanate hydrolase	urea-1-carboxylate + H2O = 2 NH3 + 2 CO2	urea degradation	0	0	-	-	-	-	0
R-0797	putative n-methylproline demethylase	monomethyl-proline + demethylated-methyl-acceptor = L-proline	stachydrine degradation	0	0	1	0	-	-	0
R-0798	choline kinase	choline + ATP = phosphoryl-choline + ADP + 2 H+	phosphatidylcholine biosynthesis	1	1	-	-	-	-	1
R-0799	ethanolamine kinase	ethanolamine + ATP = phosphoryl-ethanolamine + ADP	phosphatidylcholine biosynthesis	1	1	-	-	-	-	1
R-0800	hydantoinase	dihydrouracil + H2O = 3-ureidopropionate	uracil degradation (reductive)	0	0	0	0	-	-	0
R-0801	UDP-N-acetylglucosamine 2-epimerase	UDP-N-acetyl-D-glucosamine + H2O = N-acetyl-D-mannosamine +	sugar nucleotide biosynthesis	1	1	-	0	-	-	1
R-0802	nigerose hydrolase	nigerose + H2O = 2 D-glucose	sugar degradation	0	0	0	0	-	-	0
R-0803	2-octaprenyl-6-methoxyphenol hydroxylase	2-octaprenyl-6-methoxyphenol + O2 + 4 H+ = 2-octaprenyl-6-meth	ubiquinone-8 biosynthesis	1	1	0	0	-	-	1
R-0804	nicotinamidase	nicotinamide + H2O = NH3 + nicotinate	NAD biosynthesis	0	0	0	0	-	-	0
R-0805	agmatinase	agmatine + H2O = urea + putrescine	putrescine biosynthesis	1	1	0	0	-	-	1
R-0806	phosphopantothenoylcysteine decarboxylase	(R)-4'-phosphopantothenoyl-L-cysteine = 4'-phosphopantetheine	coenzyme A biosynthesis	1	1	0	0	-	-	1
R-0807	phosphopantothenoylcysteine synthetase	(D)-4'-phosphopantothenate + L-cysteine + CTP = (R)-4'-phospho	coenzyme A biosynthesis	1	1	0	0	-	-	1
R-0808	(S)-adenosylmethionine:2-DMK methyltransferase	(S)-adenosyl-L-methionine + demethylmenaquinone-8 = (S)-aden	menaquinone-8 biosynthesis	1	1	0	0	-	-	1
R-0809	2-octaprenyl-6-methoxy-1,4-benzoquinone methyla	(S)-adenosyl-L-methionine + 2-octaprenyl-6-methoxy-1,4-benzoq	ubiquinone-8 biosynthesis	1	1	0	0	-	-	1
R-0810	biotin synthase	sulfurated-sulfur-donor + dethiobiotin + 2 (S)-adenosyl-L-methio	biotin biosynthesis	1	1	0	0	-	-	1
R-0811	5-carboxymethyl-2-oxo-hex-3-ene-1,7-dioate decarb	5-carboxy-2-oxohept-3-enedioate = 2-hydroxyhepta-2,4-dienedio	4-hydroxyphenylacetate degradatio	0	0	0	0	-	-	0
R-0812	5-carboxymethyl-2-hydroxymuconate isomerase	5-carboxymethyl-2-hydroxymuconate = 5-carboxy-2-oxohept-3-e	4-hydroxyphenylacetate degradatio	0	0	0	0	-	-	0
R-0813	rubredoxin NAD+ reductase	oxidized-rubredoxin + NADH = reduced-rubredoxin + NAD+	oxidative phosphorilation	1	1	0	0	-	-	1
R-0814	pyridine nucleotide-disulphide oxidoreductase	oxidized-electron-acceptor + NADH + H+ = reduced-electron-acce	oxidative phosphorilation	1	1	0	0	-	-	1
R-0815	catalase	ethanol + H2O2 = acetaldehyde + 2 H2O	oxidative ethanol degradation	0	0	0	0	-	-	0
R-0816	maltose acetyltransferase	maltose + acetyl-CoA = acetylmaltose + CoA	starch and sucrose metabolism	1	1	0	0	-	-	1
R-0817	D-lactate dehydrogenase	D-lactate + NAD+ = pyruvate + NADH + H+	methylglyoxal degradation	0	0	-	0	-	-	0
R-0819	enoyl-CoA hydratase (n-6:0)	trans-hex-2-enoyl-CoA + H2O = (3S)-3-hydroxyhexanoyl-CoA	fatty acid b-oxidation	0	0	0	0	-	-	0
R-0820	enoyl-CoA hydratase (n-8:0)	trans-oct-2-enoyl-CoA + H2O = (3S)-3-hydroxyoctanoyl-CoA	fatty acid b-oxidation	0	0	0	0	-	-	0
R-0821	enoyl-CoA hydratase (n-12:0)	trans-dodec-2-enoyl-CoA + H2O = (3S)-3-hydroxydodecanoyl-CoA	fatty acid b-oxidation	0	0	0	0	-	-	0
R-0822	enoyl-CoA hydratase (n-14:0)	trans-tetradec-2-enoyl-CoA + H2O = (3S)-3-hydroxytetradecanoyl-	fatty acid b-oxidation	0	0	0	0	-	-	0
R-0823	enoyl-CoA hydratase (n-16:0)	trans-hexadec-2-enoyl-CoA + H2O = (3S)-3-hydroxyhexadecanoyl-	fatty acid b-oxidation	0	0	0	0	-	-	0
R-0824	enoyl-CoA hydratase (n-18:0)	trans-octadec-2-enoyl-CoA + H2O = (3S)-3-hydroxyoctadecanoyl-(	fatty acid b-oxidation	0	0	0	0	-	-	0
R-0825	enoyl-CoA hydratase (n-12:1)	trans-3-cis-5-dodecenoyl-CoA + H2O = (3S)-3-hydroxy-cis-dodec-5	fatty acid b-oxidation	0	0	0	0	-	-	0
R-0826	enoyl-CoA hydratase (n-14:1)	trans-3-cis-7-tetradecenoyl-CoA + H2O = (3S)-3-hydroxy-cis-tetrad	fatty acid b-oxidation	0	0	0	0	-	-	0

R-0827	enoyl-CoA hydratase (n-16:1)	trans-3-cis-9-hexadecenoyl-CoA + H2O = (3S)-3-hydroxy-cis-hexad	fatty acid b-oxidation	0	0	0	0	-	-		0	
R-0828	enoyl-CoA hydratase (n-18:1)	trans-3-cis-11-octadecenoyl-CoA + H2O = (3S)-3-hydroxy-cis-octac	fatty acid b-oxidation	0	0	0	0	-	-		0	
R-0829	cardiolipin synthase (n-16:0)	phosphatidylglycerol(dihexadecanoyl) = cardiolipin(dihexadecan	cardiolipin biosynthesis	1	1	0	0	-	-		1	
R-0830	cardiolipin synthase (n-16:1)	phosphatidylglycerol(dihexadec-9-enoyl) = cardiolipin(dihexadec	cardiolipin biosynthesis	1	1	0	0	-	-		1	
R-0831	cardiolipin synthase (n-18:0)	phosphatidylglycerol(dioctadecanoyl) = cardiolipin(dioctadecanc	cardiolipin biosynthesis	1	1	0	0	-	-		1	
R-0832	cardiolipin synthase (n-18:1)	phosphatidylglycerol(dioctadec-9-enoyl) = cardiolipin(dioctadec-	cardiolipin biosynthesis	1	1	0	0	-	-		1	
R-0833	hydroxymethylpyrimidine kinase	ATP + hydroxymethylpyrimidine = ADP + hydroxymethylpyrimidir	thiamin biosynthesis	1	1	0	0	-	-		1	
R-0834	phosphohydroxymethylpyrimidine kinase	hydroxymethylpyrimidine-phosphate + ATP = 4-amino-5-hydroxy	thiamin biosynthesis	1	1	0	0	-	-		1	
R-0835	ferredoxin--nitrite reductase	NO2- + 6 reduced-ferredoxin + 7 H+ = NH3 + 6 oxidized-ferredoxin	nitrate reduction	0	0	0	0	-	-		0	
R-0837	saccharopine dehydrogenase (NADP+, L-glutamate-f	saccharopine + NADP+ + H2O = L-glutamate + 2-aminoadipate-6-s	lysine biosynthesis	1	1	-	-	-	-		1	
R-0838	nicotinate phosphoribosyltransferase	nicotinate-mononucleotide + diphosphate = nicotinate + 5-phosp	NAD salvage pathway	1	1	0	0	-	-		1	
R-0839	pantetheine-phosphate adenylyltransferase	4'-phosphopantetheine + ATP = dephospho-CoA + diphosphate	coenzyme A biosynthesis	1	1	0	0	-	-		1	
R-0840	UDP-N-acetylmuramoylalanine--D-glutamate ligase	UDP-N-acetylmuramoyl-L-alanine + D-glutamate + ATP = UDP-N-a	peptidoglycan biosynthesis	1	1	0	0	-	-		1	
R-0841	UDP-N-acetylmuramoylalanyl-D-glutamyl-2,6-diamir	UDP-N-acetylmuramoyl-L-alanyl-D-glutamyl-meso-2,6-diaminopi	peptidoglycan biosynthesis	1	1	0	0	-	-		1	
R-0842	phospho-N-acetylmuramoyl-pentapeptide-transfera	UDP-N-acetylmuramoyl-L-alanyl-D-glutamyl-meso-2,6-diaminopi	peptidoglycan biosynthesis	1	1	0	0	-	-		1	
R-0843	phospho-N-acetylmuramoyl-pentapeptide-transfera	UDP-N-acetylmuramoyl-L-alanyl-D-glutamyl-L-lysyl-D-alanyl-D-ala	peptidoglycan biosynthesis	1	1	0	0	-	-		1	
R-0844	UDP-N-acetylmuramoylalanyl-D-glutamate 2,6-diam	UDP-N-acetylmuramoyl-L-alanyl-D-glutamate + meso-diaminohe	peptidoglycan biosynthesis	1	1	0	0	-	-		1	
R-0845	UDP-N-acetylglucosamine acyltransferase	(3R)-3-hydroxytetradecanoyl-[acyl-carrier-protein] + UDP-N-acety	lipid-A-precursor biosynthesis	1	1	0	0	-	-		1	
R-0846	lipid-A-disaccharide synthase	2,3-bis(3-hydroxymyristoyl)-b-D-glucosaminyl-1-phosphate + UDF	lipid-A-precursor biosynthesis	1	1	-	-	-	-		1	
R-0847	tartrate dehydrogenase	L-tartrate + NAD+ = 2-hydroxy-3-oxosuccinate + NADH + H+	tartrate metabolism	0	0	0	0	-	-		0	
R-0848	tartrate decarboxylase	L-tartrate = D-glycerate + CO2	tartrate metabolism	0	0	0	0	-	-		0	
R-0849	D-malate dehydrogenase [decarboxylating]	(R)-malate + NAD+ = CO2 + pyruvate + NADH + H+	D-malate degradation	0	0	0	0	-	-		0	
R-0850	precorrin-6A reductase	precorrin-6A + NADPH + H+ = precorrin-6B + NADP+	adenosylcobalamin biosynthesis pa	1	1	0	0	-	-		1	
R-0851	adenosyl-cobyric acid synthase	adenosyl-cobyrinate-a,c-diamide + 4 L-glutamine + 4 ATP + 4 H2O	adenosylcobalamin biosynthesis pa	1	1	0	0	-	-		1	
R-0852	adenosylcobinamide-phosphate synthase	ATP + adenosyl-cobyrate + (R)-1-amino-2-propanol-O-2-phosphat	adenosylcobalamin biosynthesis pa	1	1	0	0	-	-		1	
R-0853	L-threonine-O-3-phosphate decarboxylase	L-threonine-O-3-phosphate = (R)-1-amino-2-propanol-O-2-phospl	adenosylcobalamin biosynthesis pa	1	1	0	0	-	-		1	
R-0854	2-nitropropane dioxygenase	2 2-nitropropane + O2 = 2 NO2- + 2 acetone + 2 H+	2-nitropropane degradation	0	0	0	0	-	-		0	
R-0855	aerobic magnesium-protoporphyrin IX monomethyl	131-oxo-magnesium-protoporphyrin-IX-13-monomethyl-ester + N	bacteriochlorophyll a biosynthesis	1	1	1	1	-	-		1	
R-0856	aerobic magnesium-protoporphyrin IX monomethyl	131-hydroxy-magnesium-protoporphyrin-IX-13-monomethyl-este	bacteriochlorophyll a biosynthesis	1	1	1	1	-	-		1	
R-0857	magnesium protoporphyrin IX methyltransferase	Mg-protoporphyrin + (S)-adenosyl-L-methionine = (S)-adenosyl-L-	bacteriochlorophyll a biosynthesis	1	1	-	-	-	-		1	
R-0858	protochlorophyllide reductase	monovinyl-protochlorophyllidea + NADPH + H+ = chlorophyllidea	bacteriochlorophyll a biosynthesis	1	1	1	0	-	-		1	
R-0859	3-vinyl bacteriochlorophyllide a hydratase	3-vinyl-bacteriochlorophyllidea + H2O = 2-desacetyl-2-hydroxyetl	bacteriochlorophyll a biosynthesis	1	1	-	1	-	-		1	
R-0860	chlorophyllide hydratase	chlorophyllidea + H2O = 3-hydroxyethylchlorophyllidea	bacteriochlorophyll a biosynthesis	1	1	-	1	-	-		1	
R-0861	bacteriochlorophyll a synthase	bacteriochlorophyllidea + all-trans-geranyl-geranyl-diphosphate	bacteriochlorophyll a biosynthesis	1	1	-	0	-	-		1	
R-0862	geranylgeranyl reductase 1	geranylgeranyl-bacteriochlorophylla + NADPH + H+ = dihydrogera	bacteriochlorophyll a biosynthesis	1	1	0	1	-	-		1	
R-0863	geranylgeranyl reductase 2	dihydrogeranylgeranyl-bacteriochlorophylla + NADPH + H+ = tetr	bacteriochlorophyll a biosynthesis	1	1	0	1	-	-		1	
R-0864	geranylgeranyl reductase 3	tetrahydrogeranylgeranyl-bacteriochlorophylla + NADPH + H+ = b	bacteriochlorophyll a biosynthesis	1	1	0	1	-	-		1	
R-0865	arsenate reductase	arsenate + reduced-glutaredoxin = arsenite + oxidized-glutaredox	arsenate detoxification	0	0	-	0	-	-		0	
R-0866	phosphonate dehydrogenase	phosphonate + NAD+ + H2O = phosphate + NADH + H+	unassigned	1	1	0	0	-	-		1	
R-0867	methylglyoxal reductase	methylglyoxal + NADPH + H+ = acetol + NADP+	methylglyoxal degradation	0	0	0	0	-	-		0	
R-0868	b-keto ester reductase	ethyl-2-methylacetoacetate + NADPH + H+ = ethyl-(2R)-methyl-(3	unassigned	1	1	0	0	-	-		1	
R-0869	2,5-diketo-D-gluconate reductase A	2,5-didehydro-D-gluconate + NADPH + H+ = 2-keto-L-gluconate +	ketogluconate metabolism	0	0	0	0	-	-		0	
R-0870	chromate reductase	Cr6+ + 2 NADPH + O2 = Cr3+ + H2O2 + 2 NADP+	chromium metabolism	0	0	0	0	-	-		0	
R-0871	nitrilase	indole-3-acetonitrile + 2 H2O = NH3 + indole-3-acetate + H+	IAA biosynthesis	1	1	-	0	-	-		1	
R-0872	neurosporene dehydratase	neurosporene + H2O = hydroxyneurosporene	carotenoids biosynthesis	1	1	0	0	-	-		1	
R-0873	hydroxyneurosporene synthase 1	trans-lycopene + H2O = rhodopin	carotenoids biosynthesis	1	1	0	0	-	-		1	
R-0874	hydroxyneurosporene synthase 2	anhydrorhodovibrin + H2O = rhodovibrin	carotenoids biosynthesis	1	1	0	0	-	-		1	
R-0875	hydroxyneurosporene synthase 3	3,4-dihydroanhydrorhodovibrin + H2O = 3',4'-dihydrorhodovibrin	carotenoids biosynthesis	1	1	0	0	-	-		1	
R-0876	aerobic magnesium-protoporphyrin IX monomethyl	Mg-protoporphyrin-monomethyl-ester + NADPH + O2 + H+ = 131-	bacteriochlorophyll a biosynthesis	1	1	1	1	-	-		1	
R-0877	3,8-divinyl protochlorophyllide 8-vinyl reductase	divinyl-protochlorophyllidea + NADPH + H+ = monovinyl-protoch	bacteriochlorophyll a biosynthesis	1	1	-	-	-	-		1	
R-0878	molybdopterin adenylyltransferase	ATP + molybdopterin = molybdopterin-AMP + diphosphate	molybdenum cofactor biosynthesis	1	1	-	-	-	-		1	
R-0879	3-hydroxyacyl-CoA epimerase (n-4:0)	(S)-3-hydroxybutanoyl-CoA = (R)-3-hydroxybutanoyl-CoA	fatty acid b-oxidation	0	0	0	0	-	1		0	
R-0880	3-hydroxyacyl-CoA epimerase (n-6:0)	(3S)-3-hydroxyhexanoyl-CoA = (3R)-3-hydroxyhexanoyl-CoA	fatty acid b-oxidation	0	0	0	0	-	-		0	
R-0881	3-hydroxyacyl-CoA epimerase (n-8:0)	(3S)-3-hydroxyoctanoyl-CoA = (3R)-3-hydroxyoctanoyl-CoA	fatty acid b-oxidation	0	0	0	0	-	-		0	
R-0882	3-hydroxyacyl-CoA epimerase (n-12:0)	(3S)-3-hydroxydodecanoyl-CoA = (3R)-3-hydroxydodecanoyl-CoA	fatty acid b-oxidation	0	0	0	0	-	-		0	
R-0883	3-hydroxyacyl-CoA epimerase (n-14:0)	(3S)-3-hydroxytetradecanoyl-CoA = (3R)-3-hydroxytetradecanoyl-	fatty acid b-oxidation	0	0	0	0	-	-		0	
R-0884	3-hydroxyacyl-CoA epimerase (n-16:0)	(3S)-3-hydroxyhexadecanoyl-CoA = (3R)-3-hydroxyhexadecanoyl-	fatty acid b-oxidation	0	0	0	0	-	-		0	
R-0885	3-hydroxyacyl-CoA epimerase (n-18:0)	(3S)-3-hydroxyoctadecanoyl-CoA = (3R)-3-hydroxyoctadecanoyl-C	fatty acid b-oxidation	0	0	0	0	-	-		0	
R-0886	3-hydroxyacyl-CoA epimerase (n-12:1)	(3S)-3-hydroxy-cis-dodec-5-enoyl-CoA = (3R)-3-hydroxy-cis-dodec	fatty acid b-oxidation	0	0	0	0	-	-		0	
R-0887	3-hydroxyacyl-CoA epimerase (n-14:1)	(3S)-3-hydroxy-cis-tetradec-7-enoyl-CoA = (3R)-3-hydroxy-cis-tetra	fatty acid b-oxidation	0	0	0	0	-	-		0	
R-0888	3-hydroxyacyl-CoA epimerase (n-16:1)	(3S)-3-hydroxy-cis-hexadec-9-enoyl-CoA = (3R)-3-hydroxy-cis-hexa	fatty acid b-oxidation	0	0	0	0	-	-		0	
R-0889	3-hydroxyacyl-CoA epimerase (n-18:1)	(3S)-3-hydroxy-cis-octadec-11-enoyl-CoA = (3R)-3-hydroxy-cis-octa	fatty acid b-oxidation	0	0	0	0	-	-		0	
R-0890	3-hydroxyacyl-CoA dehydrogenase (n-4:0)	(S)-3-hydroxybutanoyl-CoA + NAD+ = acetoacetyl-CoA + NADH + H	fatty acid b-oxidation	0	0	0	0	-	1		0	
R-0891	3-hydroxyacyl-CoA dehydrogenase (n-6:0)	(3S)-3-hydroxyhexanoyl-CoA + NAD+ = 3-oxohexanoyl-CoA + NAD	fatty acid b-oxidation	0	0	0	0	-	-		0	
R-0892	3-hydroxyacyl-CoA dehydrogenase (n-8:0)	(3S)-3-hydroxyoctanoyl-CoA + NAD+ = 3-oxooctanoyl-CoA + NADI	fatty acid b-oxidation	0	0	0	0	-	-		0	
R-0893	3-hydroxyacyl-CoA dehydrogenase (n-12:0)	(3S)-3-hydroxydodecanoyl-CoA + NAD+ = 3-oxododecanoyl-CoA +	fatty acid b-oxidation	0	0	0	0	-	-		0	
R-0894	3-hydroxyacyl-CoA dehydrogenase (n-14:0)	(3S)-3-hydroxytetradecanoyl-CoA + NAD+ = 3-oxotetradecanoyl-C	fatty acid b-oxidation	0	0	0	0	-	-		0	
R-0895	3-hydroxyacyl-CoA dehydrogenase (n-16:0)	(3S)-3-hydroxyhexadecanoyl-CoA + NAD+ = 3-oxohexadecanoyl-C	fatty acid b-oxidation	0	0	0	0	-	-		0	
R-0896	3-hydroxyacyl-CoA dehydrogenase (n-18:0)	(3S)-3-hydroxyoctadecanoyl-CoA + NAD+ = 3-oxooctadecanoyl-Co	fatty acid b-oxidation	0	0	0	0	-	-		0	
R-0897	3-hydroxyacyl-CoA dehydrogenase (n-12:1)	(3S)-3-hydroxy-cis-dodec-5-enoyl-CoA + NAD+ = 3-oxo-cis-dodec-5	fatty acid b-oxidation	0	0	0	0	-	-		0	
R-0898	3-hydroxyacyl-CoA dehydrogenase (n-14:1)	(3S)-3-hydroxy-cis-tetradec-7-enoyl-CoA + NAD+ = 3-oxo-cis-tetra	fatty acid b-oxidation	0	0	0	0	-	-		0	
R-0899	3-hydroxyacyl-CoA dehydrogenase (n-16:1)	(3S)-3-hydroxy-cis-hexadec-9-enoyl-CoA + NAD+ = 3-oxo-cis-hexa	fatty acid b-oxidation	0	0	0	0	-	-		0	
R-0900	3-hydroxyacyl-CoA dehydrogenase (n-18:1)	(3S)-3-hydroxy-cis-octadec-11-enoyl-CoA + NAD+ = 3-oxo-cis-octa	fatty acid b-oxidation	0	0	0	0	-	-		0	
R-0901	enoyl-CoA isomerase (n-6:0)	trans-hex-2-enoyl-CoA = cis-hex-3-enoyl-CoA	fatty acid b-oxidation	0	0	0	0	-	-		0	
R-0902	enoyl-CoA isomerase (n-8:0)	trans-oct-2-enoyl-CoA = cis-oct-3-enoyl-CoA	fatty acid b-oxidation	0	0	0	0	-	-		0	
R-0903	enoyl-CoA isomerase (n-12:0)	trans-dodec-2-enoyl-CoA = cis-dodec-3-enoyl-CoA	fatty acid b-oxidation	0	0	0	0	-	-		0	
R-0904	enoyl-CoA isomerase (n-14:0)	trans-tetradec-2-enoyl-CoA = cis-tetradec-3-enoyl-CoA	fatty acid b-oxidation	0	0	0	0	-	-		0	
R-0905	enoyl-CoA isomerase (n-16:0)	trans-hexadec-2-enoyl-CoA = cis-hexadec-3-enoyl-CoA	fatty acid b-oxidation	0	0	0	0	-	-		0	
R-0906	enoyl-CoA isomerase (n-18:0)	trans-octadec-2-enoyl-CoA = cis-octadec-3-enoyl-CoA	fatty acid b-oxidation	0	0	0	0	-	-		0	
R-0907	enoyl-CoA isomerase (n-12:1)	trans-2-cis-5-dodecenoyl-CoA = cis-3-cis-5-dodecenoyl-CoA	fatty acid b-oxidation	0	0	0	0	-	-		0	
R-0908	enoyl-CoA isomerase (n-14:1)	trans-2-cis-7-tetradecenoyl-CoA = cis-3-cis-7-tetradecenoyl-CoA	fatty acid b-oxidation	0	0	0	0	-	-		0	
R-0909	enoyl-CoA isomerase (n-16:1)	trans-2-cis-9-hexadecenoyl-CoA = cis-3-cis-9-hexadecenoyl-CoA	fatty acid b-oxidation	0	0	0	0	-	-		0	
R-0910	enoyl-CoA isomerase (n-18:1)	trans-2-cis-11-octadecenoyl-CoA = cis-3-cis-11-octadecenoyl-CoA	fatty acid b-oxidation	0	0	0	0	-	-		0	
R-0911	enoyl-CoA isomerase (n-4:0)	crotonyl-CoA = vinylacetyl-CoA	fatty acid b-oxidation	0	0	0	0	-	-		0	
R-0913	3-ketoacyl-CoA thiolase (n-6:0)	3-oxohexanoyl-CoA + CoA = acetyl-CoA + butyryl-CoA	fatty acid b-oxidation	0	0	0	0	-	-		0	
R-0914	3-ketoacyl-CoA thiolase (n-8:0)	3-oxooctanoyl-CoA + CoA = acetyl-CoA + hexanoyl-CoA	fatty acid b-oxidation	0	0	0	0	-	-		0	
R-0915	3-ketoacyl-CoA thiolase (n-10:0)	3-oxodecanoyl-CoA + CoA = acetyl-CoA + octanoyl-CoA	fatty acid b-oxidation	0	0	0	0	-	-		0	
R-0916	3-ketoacyl-CoA thiolase (n-12:0)	3-oxododecanoyl-CoA + CoA = acetyl-CoA + decanoyl-CoA	fatty acid b-oxidation	0	0	0	0	-	-		0	
R-0917	3-ketoacyl-CoA thiolase (n-14:0)	3-oxotetradecanoyl-CoA + CoA = acetyl-CoA + dodecanoyl-CoA	fatty acid b-oxidation	0	0	0	0	-	-		0	
R-0918	3-ketoacyl-CoA thiolase (n-16:0)	3-oxohexadecanoyl-CoA + CoA = acetyl-CoA + tetradecanoyl-CoA	fatty acid b-oxidation	0	0	0	0	-	-		0	
R-0919	3-ketoacyl-CoA thiolase (n-18:0)	3-oxooctadecanoyl-CoA + CoA = acetyl-CoA + hexadecanoyl-CoA	fatty acid b-oxidation	0	0	0	0	-	-		0	
R-0920	3-ketoacyl-CoA thiolase (n-12:1)	3-oxo-cis-dodec-5-enoyl-CoA + CoA = acetyl-CoA + cis-dec-3-enoy	fatty acid b-oxidation	0	0	0	0	-	-		0	
R-0921	3-ketoacyl-CoA thiolase (n-14:1)	3-oxo-cis-tetradec-7-enoyl-CoA + CoA = acetyl-CoA + cis-dodec-5-	fatty acid b-oxidation	0	0	0	0	-	-		0	
R-0922	3-ketoacyl-CoA thiolase (n-16:1)	3-oxo-cis-hexadec-9-enoyl-CoA + CoA = acetyl-CoA + cis-tetradec-	fatty acid b-oxidation	0	0	0	0	-	-		0	
R-0923	3-ketoacyl-CoA thiolase (n-18:1)	3-oxo-cis-octadec-11-enoyl-CoA + CoA = acetyl-CoA + hexadec-9-e	fatty acid b-oxidation	0	0	0	0	-	-		0	
R-0924	enoyl-CoA isomerase (n-10:0)	trans-dec-2-enoyl-CoA = cis-dec-2-enoyl-CoA	fatty acid b-oxidation	0	0	0	0	-	-		0	
R-0925	3-hydroxyacyl-CoA dehydrogenase (n-10:0)	(3S)-3-hydroxydecanoyl-CoA + NAD+ = 3-oxodecanoyl-CoA + NAD	fatty acid b-oxidation	0	0	0	0	-	-		0	
R-0926	3-hydroxyacyl-CoA epimerase (n-10:0)	(3S)-3-hydroxydecanoyl-CoA = (3R)-3-hydroxydecanoyl-CoA	fatty acid b-oxidation	0	0	0	0	-	-		0	
R-0927	enoyl-CoA hydratase (n-10:0)	trans-dec-2-enoyl-CoA + H2O = (3S)-3-hydroxydecanoyl-CoA	fatty acid b-oxidation	0	0	0	0	-	-		0	
R-0928	2-octaprenyl-3-methyl-6-methoxy-1,4-benzoquinone	2-octaprenyl-3-methyl-6-methoxy-1,4-benzoquinol + O2 + H+ = 3-	ubiquinone-8 biosynthesis	1	1	-	-	-	-		1	
R-0929	L-sorbosone to L-sorbosone-2,6-lactone (spontaneou	L-sorbosone = L-sorbosone-2,6-lactone	sugar metabolism	1	1	-	-	-	-		1	
R-0930	pantothenate synthetase	b-alanine + L-pantoate + ATP = (R)-pantothenate + diphosphate +	pantothenate biosynthesis	1	1	0	0	-	-		1	
R-0933	phosphatidylglycerophosphatase (n-16:0)	phosphatidylglycerolphosphate(dihexadecanoyl) + H2O = phospl	glycerophospholipid metabolism	1	1	-	-	-	-		1	
R-0934	phosphatidylglycerophosphatase (n-16:1)	phosphatidylglycerolphosphate(dihexadec-9-enoyl) + H2O = phos	glycerophospholipid metabolism	1	1	-	-	-	-		1	
R-0935	phosphatidylglycerophosphatase (n-18:0)	phosphatidylglycerolphosphate(dioctadecanoyl) + H2O = phosph	glycerophospholipid metabolism	1	1	-	-	-	-		1	
R-0936	phosphatidylglycerophosphatase (n-18:1)	phosphatidylglycerolphosphate(dioctadec-9-enoyl) + H2O = phos	glycerophospholipid metabolism	1	1	-	-	-	-		1	
R-0937	protoporphyrinogen IX oxidase	protoporphyrinogen-IX + 3 O2 = protoporphyrin-IX + 3 H2O2	bacteriochlorophyll a biosynthesis	1	1	-	-	-	-		1	
R-0938	1-amino-propan-2-one-3-phosphate synthesis	(2S)-2-amino-3-oxo-4-phosphonooxybutanoate = 1-amino-propai	pyridoxal-5'-phosphate biosynthesis	1	1	-	-	-	-		1	
R-0939	riboflavin kinase	riboflavin + ATP = ADP + FMN	flavin biosynthesis	1	1	0	0	-	-		1	
R-0940	FMN adenylyltransferase	ATP + FMN = diphosphate + FAD	flavin biosynthesis	1	1	0	0	-	-		1	
R-0941	UDP-2,3-diacylglucosamine hydrolase	UDP-2,3-bis(3-hydroxymyristoyl)glucosamine + H2O = 2,3-bis(3-hy	peptidoglycan biosynthesis	1	1	-	-	-	-		1	
R-0942	cob(I)yrinic acid a,c-diamide adenosyltransferase	cob(I)yrinate-a,c-diamide + ATP = adenosyl-cobyrinate-a,c-diamic	adenosylcobalamin biosynthesis pa	1	1	0	0	-	-		1	
R-0943	cob(II)yrinate a,c-diamide reductase	cob(II)yrinate-a,c-diamide + FMNH2 = cob(I)yrinate-a,c-diamide +	adenosylcobalamin biosynthesis pa	1	1	0	0	-	-		1	
R-0944	a-ribazole-5'-phosphate phosphatase	a-ribazole-5'-P + H2O = a-ribazole + phosphate	adenosylcobalamin biosynthesis pa	0	0	-	-	-	1		0	
R-0945	adenosylcobalamine-5'-phosphate phosphatase	adenosylcobalamin-5'-phosphate + H2O = coenzyme-B12 + phosp	adenosylcobalamin biosynthesis pa	1	1	-	-	-	1		1	
R-0946	L-threonine kinase	L-threonine + ATP = L-threonine-O-3-phosphate + ADP	adenosylcobalamin biosynthesis pa	1	1	-	-	-	-		1	
R-0947	flavin destructase	FMNH2 + O2 = 2 dimethylbenzimidazole + D-erythrose-4-phospha	adenosylcobalamin biosynthesis pa	1	1	-	-	-	-		1	
R-0965	N-succinyldiaminopimelate-aminotransferase	L-glutamate + N-succinyl-2-amino-6-ketopimelate = 2-oxoglutarat	lysine biosynthesis	1	1	-	-	-	-		1	
R-0966	L-asparaginase (periplasm)	L-asparagine[p] + H2O[p] = L-aspartate[p] + NH3[p]	aspartate/asparagine degradation	0	0	0	0	-	-		0	
R-0967	superoxide dismutase (periplasm)	2 superoxide[p] + 2 H+[p] = H2O2[p] + O2[p]	superoxide radicals degradation	0	0	0	0	-	-		0	
R-0968	galactose oxidase (extracellular)	D-galactose[e] + O2[e] = D-galacto-hexodialdose[e] + H2O2[e]	sugar oxidation	0	0	-	0	-	-		0	
R-0969	phytoene dehydrogenase 1 (extracellular)	phytoene[e] + reduced-electron-acceptor[e] + O2[e] = phytofluen	carotenoids biosynthesis	1	1	0	0	-	-		1	
R-0970	phytoene dehydrogenase 2 (extracellular)	phytofluene[e] + reduced-electron-acceptor[e] + O2[e] = zeta-carc	carotenoids biosynthesis	1	1	0	0	-	-		1	
R-0971	zeta-carotene desaturase 1 (extracullular)	zeta-carotene[e] + reduced-electron-acceptor[e] + O2[e] = neuros	carotenoids biosynthesis	1	1	0	0	-	-		1	
R-0972	zeta-carotene desaturase 2 (extracellular)	neurosporene[e] + reduced-electron-acceptor[e] + O2[e] = trans-l	carotenoids biosynthesis	1	1	0	0	-	-		1	
R-0973	deoxynucleoside triphosphate pyrophosphohydrola:	dATP + H2O = dAMP + diphosphate	salvage pathways of purine and pyri	0	0	0	0	-	-		0	
R-0974	deoxynucleoside triphosphate pyrophosphohydrola:	dTTP + H2O = dTMP + diphosphate	salvage pathways of purine and pyri	0	0	0	0	-	-		0	
R-0975	deoxynucleoside triphosphate pyrophosphohydrola:	dGTP + H2O = dGMP + diphosphate	salvage pathways of purine and pyri	0	0	0	0	-	-		0	
R-0976	deoxynucleoside triphosphate pyrophosphohydrola:	dCTP + H2O = dCMP + diphosphate	salvage pathways of purine and pyri	0	0	0	0	-	-		0	
R-0977	NADH-azoreductase	methyl-red + 2 NADH + 2 H+ = anthranilate + N,N'-dimethyl-p-phe	unassigned	1	1	0	0	-	-		1	
R-0978	acetyldehyde dehydrogenase	acetaldehyde + NADP+ + H2O = acetate + NADPH + H+	oxidative ethanol degradation	1	1	0	1	-	-		1	
R-0979	beta-glucosidase	short-glucan(y) + y H2O = y b-D-glucose	starch and sucrose metabolism	1	1	0	0	-	-		1	
R-0980	2-acetolactate decarboxylation	2-acetolactate = CO2 + 2 H+ + diacetyl	diacetyl, acetoin, 2,3-butanediol bio	0	0	-	-	-	-		0	
R-0981	5-methylthioribose-1-phosphate isomerase	(S)-methyl-5-thio-alpha-D-ribose-1-phosphate = (S)-methyl-5-thio	methionine salvage pathway	1	1	0	0	-	-		1	
R-0982	S-adenosylmethionine decarboxylase	(S)-adenosyl-L-methionine = CO2 + (S)-adenosylmethioninamine	polyamine biosynthesis	1	1	-	-	-	-		1	
R-0983	adenylylsulfate kinase	adenosine-5'-phosphosulfate + ATP = phosphoadenosine-5'-phos	sulfate reduction I (assimilatory)	1	1	-	-	-	-		1	
R-0984	nitrate reductase assimilatory	NO3- + NADPH + H+ = NO2- + NADP+ + H2O	nitrate reduction	0	0	-	-	-	-		0	
R-0985	adenosine-3'(2'),5'-bisphosphate nucleotidase	adenosine-3',5'-bisphosphate + H2O = phosphate + AMP	sulfate reduction I (assimilatory)	1	1	0	0	-	-		1	
R-0988	5-keto-D-gluconate 5-reductase (inferred from physi	NADPH + 5-dehydro-D-gluconate + H+ = NADP+ + D-gluconate	ketogluconate metabolism	0	0	-	-	-	-		0	
R-0989	malyl-CoA thioesterase	(S)-malyl-CoA + H2O = (S)-malate + CoA	acetate metabolism	1	1	0	0	-1	-		1	
R-0990	triphosphate phosphohydrolase (inferred from gap)	triphosphate + H2O = diphosphate + phosphate	phosphorous metabolism	1	1	-	-	-	-		1	
R-0991	NADPH oxidoreductase (OYE)	NADPH + H+ + O2 = NADP+ + H2O2	NADPH oxidation	1	1	0	0	-	-		1	0
R-0992	glutathione peroxidase (periplasmic)	H2O2[p] + 2 glutathione = glutathione-disulfide + 2 H2O[p]	superoxide radicals degradation	0	0	0	0	-	-		0	
R-0993	5'-deoxyadenosine phosphorylase	5'-deoxyadenosine + phosphate = adenine + 5'-deoxyribose-1-ph	unassigned	1	1	-1	0	-	-		1	
R-0994	aminodeoxychorismate lyase	4-amino-4-deoxychorismate = p-aminobenzoate + pyruvate	folate biosynthesis	1	1	-	-	-	-		1	
R-0995	sulfur donor activation	unsulfurated-sulfur-acceptor + H2S = sulfurated-sulfur-donor	sulfur metabolism	1	1	-	-	-	-		1	
R-0996	aspartate 1-decarboxylase	L-aspartate = b-alanine + CO2	coenzyme A biosynthesis	1	1	-	-	-	-		1	
R-0997	molybdopterin-synthase activations	sulfurated-sulfur-donor + molybdopterin-synthase = sulfurated-m	molybdenum cofactor biosynthesis	1	1	-	-	-	-		1	
R-0998	erythronate-4-phosphate dehydrogenase	erythronate-4-phosphate + NAD+ = 2-oxo-3-hydroxy-4-phosphob	pyridoxal-5'-phosphate biosynthesis	1	1	-	-	-	-		1	
R-0999	erythrose 4-phosphate dehydrogenase	D-erythrose-4-phosphate + NAD+ + H2O = erythronate-4-phospha	pyridoxal-5'-phosphate biosynthesis	1	1	-	-	-	-		1	
R-1000	glycine oxidase 5	glycine + O2 = iminoglycine + H2O2	thiamine biosynthesis	1	1	-1	0	-	-		1	
R-1001	thiamin biosynthesis 1	thiS + ATP = adenylated-thiS + diphosphate	thiamine biosynthesis	1	1	-	-	-	-		1	
R-1002	thiamin biosynthesis 2	thiI + L-cysteine = thiI-persulfide + L-alanine	thiamine biosynthesis	1	1	0	0	-	-		1	
R-1003	thiamin biosynthesis 3	adenylated-thiS + thiI-persulfide = thiS-thiI-acyl-disulfide + AMP	thiamine biosynthesis	1	1	-	-	-	-		1	
R-1004	thiamin biosynthesis 4	thiS-thiI-acyl-disulfide + thiF = thiS-thiF-acyl-disulfide + thiI	thiamine biosynthesis	1	1	-	-	-	-		1	
R-1005	debranched limit dextrin phosphorylase	debranched-limit-dextrin + n phosphate = n D-glucose-1-phospha	starch and sucrose metabolism	0	0	-	-	-	-		0	
R-1006	NAD kinase	NAD+ + ATP = NADP+ + ADP	NAD biosynthesis	1	1	0	0	-	-		1	
R-1007	photosystem I	oxidized-ferredoxin + 2 reduced-cytochrome-c + photon = reduce	photosynthesis light reactions	0	0	1	1	-	-		0	
R-1008	sulfur oxidation enzyme system	thiosulfate[p] + 5 H2O[p] + oxidized-cytochrome-c = 2 SO42-[p] +	sulfur metabolism	0	0	1	0	-	-		0	
R-1009	formaldehyde dehydrogenase	formaldehyde[p] + 2 oxidized-cytochrome-c = formate[p] + 2 H+[p	formaldehyde oxidation to formate	1	1	0	0	-	-		1	
R-1010	aldose sugar dehydrogenase (D-arabinose)	D-arabinose[p] + 2 oxidized-cytochrome-c = D-arabinono-1,4-lact	sugar oxidation	0	0	0	0	-	-		0	
R-1011	aldose sugar dehydrogenase (L-arabinose)	L-arabinose[p] + 2 oxidized-cytochrome-c = L-arabinono-1,4-lacto	sugar oxidation	0	0	0	0	-	-		0	
R-1012	D-arabinolactonase	D-arabinono-1,4-lactone[p] + H2O[p] = D-arabinonate[p]	sugar oxidation	0	0	-	-	-	-		0	
R-1013	L-arabinolactonase	L-arabinono-1,4-lactone[p] + H2O[p] = L-arabinonate[p]	sugar oxidation	0	0	-	-	-	-		0	
R-1014	aldose sugar dehydrogenase (L-lyxose)	L-lyxose[p] + 2 oxidized-cytochrome-c = L-lyxono-1,4-lactone[p] +	sugar oxidation	0	0	0	0	-	-		0	
R-1015	L-lyxolactonase	L-lyxono-1,4-lactone[p] + H2O[p] = L-lyxonate[p]	sugar oxidation	0	0	-	-	-	-		0	
R-1016	aldose sugar dehydrogenase (D-xylose)	D-xylose[p] + 2 oxidized-cytochrome-c = D-xylono-1,4-lactone[p] +	sugar oxidation	0	0	0	0	-	-		0	
R-1017	D-xylolactonase	D-xylono-1,4-lactone[p] + H2O[p] = D-xylonate[p]	sugar oxidation	0	0	-	-	-	-		0	
R-1018	aldose sugar dehydrogenase (D-ribose)	D-ribose[p] + 2 oxidized-cytochrome-c = D-ribono-1,4-lactone[p] +	sugar oxidation	0	0	0	0	-	-		0	
R-1019	D-ribolactonase	D-ribono-1,4-lactone[p] + H2O[p] = D-ribonate[p]	sugar oxidation	0	0	-	-	-	-		0	
R-1020	aldose sugar dehydrogenase (2-deoxy-D-ribose)	2-deoxy-D-ribose[p] + 2 oxidized-cytochrome-c = 2-deoxy-D-ribon	sugar oxidation	0	0	0	0	-	-		0	
R-1021	2-deoxy-D-ribolactonase	2-deoxy-D-ribono-1,4-lactone[p] + H2O[p] = 2-deoxy-D-ribonate[p	sugar oxidation	0	0	-	-	-	-		0	
R-1022	aldose sugar dehydrogenase (3-O-b-D-galactopyranc	3-O-b-D-galactopyranosyl-D-arabinose[p] + 2 oxidized-cytochrom	sugar oxidation	0	0	0	0	-	-		0	
R-1023	3-O-b-D-galactopyranosyl-D-arabinolactonase	3-O-b-D-galactopyranosyl-D-arabinono-1,4-lactone[p] + H2O[p] =	sugar oxidation	0	0	-	-	-	-		0	
R-1024	ethylamine dehydrogenase	ethylamine[p] + oxidized-amicyanin[p] + H2O[p] = acetaldehyde[	ethylamine oxidation to acetaldehy	0	0	1	0	-	-		0	
R-1025	amicyanin oxidation	2 oxidized-cytochrome-c + reduced-amicyanin[p] = 2 reduced-cyt	methylamine oxidation to formalde	0	0	1	0	-	-		0	
R-1026	photosystem I cyclic	2 reduced-ferredoxin + 2 H+ + UQ = 2 oxidized-ferredoxin + UQH2	photosynthesis light reactions	0	0	-	-	-	-		0	
R-1027	2-amino-2-deoxyisochorismate synthase	chorismate + L-glutamine = 2-amino-2-deoxy-isochorismate + L-gl	phenazine biosynthesis	1	1	-	-	-	-		1	
R-1028	2-amino-2-deoxyisochorismate hydrolase	2-amino-2-deoxy-isochorismate + H2O = trans-2,3-dihydro-3-hydr	phenazine biosynthesis	1	1	-	-	-	-		1	
R-1029	carbamate degradation (spontaneous)	carbamate + H+ = NH3 + CO2	cyanate degradation	0	0	-	-	-	-		0	
R-5001	methanol transport via diffusion (extracellular to pe	methanol[e] = methanol[p]	transport : one carbon compound	1	1	-	-	-	-		1	
R-5002	formaldehyde transport via diffusion (periplasm to c	formaldehyde[p] = formaldehyde	transport : one carbon compound	1	1	-	-	-	-		1	
R-5003	CO2 transport via diffusion (extracellular to periplasr	CO2[e] = CO2[p]	transport : one carbon compound	1	1	-	-	-	-		1	
R-5004	CO2 transport via diffusion (periplasm to cytoplasm)	CO2[p] = CO2	transport : one carbon compound	1	1	-	-	-	-		1	
R-5005	O2 transport via diffusion (extracellular to periplasm	O2[e] = O2[p]	transport : gaz	1	1	-	-	-	-		1	
R-5006	O2 transport via diffusion (periplasm to cytoplasm)	O2[p] = O2	transport : gaz	1	1	-	-	-	-		1	
R-5007	formaldehyde transport via diffusion (extracellular t	formaldehyde[e] = formaldehyde[p]	transport : one carbon compound	0	0	-	-	-	-		0	
R-5008	phosphate transport via diffusion (extracellular to pe	phosphate[e] = phosphate[p]	transport : ion	1	1	-	-	-	-		1	
R-5009	phosphate ABC transporter	phosphate[p] + ATP + H2O = 2 phosphate + ADP	transport : ion	1	1	0	0	-	-		1	
R-5010	NH3 transport via diffusion (extracellular to periplas	NH3[e] = NH3[p]	transport : ion	1	1	-	-	-	-		1	
R-5011	NH3 transport (periplasm to cytoplasm)	NH3[p] + H+[p] = NH3 + H+	transport : ion	1	1	0	0	-	-		1	
R-5012	succinate transport via diffusion (extracellular to per	succinate[e] = succinate[p]	transport : organic acid	0	1	-	-	-	-	succinate uptake	1	
R-5013	succinate symport (periplasm to cytoplasm)	succinate[p] + 2 H+[p] = succinate + 2 H+	transport : organic acid	0	1	-	0	-	-	succinate uptake	1	
R-5014	fumarate transport via diffusion (extracellular to per	fumarate[e] = fumarate[p]	transport : organic acid	0	0	-	-	-	-		0	
R-5015	fumarate symport (periplasm to cytoplasm)	fumarate[p] + 2 H+[p] = fumarate + 2 H+	transport : organic acid	0	0	-	0	-	-		0	
R-5016	citrate transport via diffusion (extracellular to peripl	citrate[e] = citrate[p]	transport : organic acid	0	0	-	-	-	-		0	
R-5017	citrate symport (periplasm to cytoplasm)	citrate[p] + 2 H+[p] = citrate + 2 H+	transport : organic acid	0	0	-	0	-	-		0	
R-5018	glycolate transport via diffusion (extracellular to peri	glycolate[e] = glycolate[p]	transport : organic acid	0	0	-	-	-	-		0	
R-5019	glycolate symport (periplasm to cytoplasm)	glycolate[p] + H+[p] = glycolate + H+	transport : organic acid	0	0	-1	0	-	-		0	
R-5020	formate transport via diffusion (extracellular to peri	formate[e] = formate[p]	transport : one carbon compound	0	0	-	-	-	-		0	
R-5021	formate symport (periplasm to cytoplasm)	formate[p] + H+[p] = formate + H+	transport : one carbon compound	1	1	-	0	-	-		1	
R-5022	oxalate transport via diffusion (extracellular to perip	oxalate[e] = oxalate[p]	transport : organic acid	0	0	-	-	-	-		0	
R-5023	oxalate/formate antiporter (periplasm to cytoplasm)	oxalate[p] + formate = oxalate + formate[p]	transport : organic acid	0	0	0	0	-	-		0	
R-5024	acetate transport via diffusion (extracellular to perip	acetate[e] = acetate[p]	transport : organic acid	0	0	-	-	-	-		0	
R-5025	sodium/acetate symporter (periplasm to cytoplasm)	acetate[p] + H+[p] = acetate + H+	transport : organic acid	0	0	-1	0	-	-		0	
R-5026	D-gluconate transport via diffusion (extracellular to	D-gluconate[e] = D-gluconate[p]	transport : sugar	0	0	-	-	-	-		0	
R-5027	H+/gluconate symporter	D-gluconate[p] + H+[p] = D-gluconate + H+	transport : sugar	0	0	-	0	-	-		0	
R-5028	putrescine transport via diffusion (extracellular to pe	putrescine[e] = putrescine[p]	transport : polyamine	0	0	-	-	-	-		0	
R-5029	putrescine ABC transporter	putrescine[p] + ATP + H2O = putrescine + ADP + phosphate	transport : polyamine	0	0	0	0	-	-		0	
R-5030	a-ketoglutarate transport via diffusion (extracellular	2-oxoglutarate[e] = 2-oxoglutarate[p]	transport : organic acid	0	0	-	-	-	-		0	
R-5031	a-ketoglutarate MFS transporter	2-oxoglutarate[p] + H+[p] = 2-oxoglutarate + H+	transport : organic acid	0	0	-1	0	-	-		0	
R-5032	PiT phosphate transporter	phosphate[p] + H+[p] = phosphate + H+	transport : ion	0	0	-	0	-	1		0	

R-5033	sulfate transport via diffusion (extracellular to peripl	SO42-[e] = SO42-[p]	transport : ion	1	1	-	-	-	-	1
R-5034	sodium/sulphate symporter	SO42-[p] + Na+[p] = SO42- + Na+	transport : ion	1	1	-	0	-	-	1
R-5035	sodium transport via diffusion (extracellular to perip	Na+[e] = Na+[p]	transport : ion	1	1	-	-	-	-	1
R-5036	thiosulfate transport via diffusion (extracellular to pe	thiosulfate[e] = thiosulfate[p]	transport : ion	0	0	-	-	-	-	0
R-5037	sulfate ABC transporter	SO42-[p] + ATP + H2O = SO42- + phosphate + ADP	transport : ion	1	1	0	0	-	-	1
R-5038	thiosulfate ABC transporter	thiosulfate[p] + ATP + H2O = thiosulfate + phosphate + ADP	transport : ion	0	0	0	0	-	-	0
R-5039	sodium-proton antiporter	Na+ + 2 H+[p] = Na+[p] + 2 H+	transport : ion	0	0	-	0	-	-	0
R-5040	nitrate ABC transporter	NO3-[p] + ATP + H2O = NO3- + phosphate + ADP	transport : ion	0	0	0	0	-	-	0
R-5041	nitrate transport via diffusion (extracellular to peripl	NO3-[e] = NO3-[p]	transport : ion	0	0	-	-	-	-	0
R-5042	glutamate transport via diffusion (extracellular to pe	L-glutamate[e] = L-glutamate[p]	transport : amino acid	0	0	-	-	-	-	0
R-5043	aspartate transport via diffusion (extracellular to per	L-aspartate[e] = L-aspartate[p]	transport : amino acid	0	0	-	-	-	-	0
R-5044	aspartate ABC transporter	L-aspartate[p] + ATP + H2O = L-aspartate + phosphate + ADP	transport : amino acid	0	0	0	0	-	-	0
R-5045	glutamate ABC transporter	L-glutamate[p] + ATP + H2O = L-glutamate + phosphate + ADP	transport : amino acid	0	0	0	0	-	-	0
R-5046	H2O transport via diffusion (extracellular to periplasi	H2O[e] = H2O[p]	transport : water	1	1	-	-	-	-	1
R-5047	H2O transport via aquaporin	H2O[p] = H2O	transport : water	1	1	-	-	-	-	1
R-5048	ethanolamine transport via diffusion (extracellular to	ethanolamine[e] = ethanolamine[p]	transport : ethanolamine	0	0	-	-	-	-	0
R-5049	ethanolamine transporter (permease)	ethanolamine[p] = ethanolamine	transport : ethanolamine	0	0	-	0	-	-	0
R-5050	malate transport via diffusion (extracellular to peripl	(S)-malate[e] = (S)-malate[p]	transport : organic acid	0	0	-	-	-	-	0
R-5051	malate symport (periplasm to cytoplasm)	(S)-malate[p] + 2 H+[p] = (S)-malate + 2 H+	transport : organic acid	0	0	-	0	-	-	0
R-5052	pyruvate transport via diffusion (extracellular to peri	pyruvate[e] = pyruvate[p]	transport : organic acid	0	0	-	-	-	-	0
R-5053	pyruvate symport (periplasm to cytoplasm)	pyruvate[p] + H+[p] = pyruvate + H+	transport : organic acid	0	0	-	-	-	-	0
R-5055	glucose porin (extracellular to periplasm)	D-glucose[e] = D-glucose[p]	transport : sugar	0	0	-	0	-	-	0
R-5056	D-ribose transport via diffusion (extracellular to peri	D-ribose[e] = D-ribose[p]	transport : pentose	0	0	-	-	-	-	0
R-5058	2-deoxy-D-ribose transport via diffusion (extracellula	2-deoxy-D-ribose[e] = 2-deoxy-D-ribose[p]	transport : pentose	0	0	-	-	-	-	0
R-5060	5-dehydro-D-gluconate transporter (inferred from ph	5-dehydro-D-gluconate[p] + H+[p] = 5-dehydro-D-gluconate + H+	transport : sugar	0	0	-	-	-	-	0
R-5061	5-dehydro-D-gluconate transport via diffusion (extra	5-dehydro-D-gluconate[e] = 5-dehydro-D-gluconate[p]	transport : sugar	0	0	-	-	-	-	0
R-5062	Co2+ transport via diffusion (extracellular to periplas	Co2+[e] = Co2+[p]	transport : ion	1	1	-	-	-	-	1
R-5063	Cobalt transporter (periplasm to cytoplasm)	Co2+[p] = Co2+	transport : ion	1	1	0	0	-	-	1
R-5064	H+ transport via diffusion (extracellular to periplasm	H+[e] = H+[p]	transport : ion	1	1	-	-	-	-	1
R-5065	H2O2 transport via diffusion (extracellular to peripla	H2O2[e] = H2O2[p]	transport : H2O2	1	1	-	-	-	-	1
R-5066	H2O2 transport via diffusion (periplasm to cytoplasm	H2O2[p] = H2O2	transport : H2O2	1	1	-	-	-	-	1
R-5067	iron ABC transporter	Fe2+[p] + ATP + H2O = Fe2+ + phosphate + ADP	transport : ion	1	1	0	0	-	1	1
R-5068	iron transport via diffusion (extracellular to periplasr	Fe2+[e] = Fe2+[p]	transport : ion	1	1	0	0	-	-	1
R-5069	molybdate ABC transporter	molybdate[p] + ATP + H2O = molybdate + phosphate + ADP	transport : ion	1	1	0	0	-	-	1
R-5070	molybdate transport via diffusion (extracellular to pe	molybdate[e] = molybdate[p]	transport : ion	1	1	-	-	-	-	1
R-5071	potassium:proton antiporter	K+ + H+[p] = K+[p] + H+	transport : ion	0	0	0	0	-	-	0
R-5072	potassium transport via diffusion (extracellular to pe	K+[e] = K+[p]	transport : ion	1	1	-	-	-	-	1
R-5073	magnesium transport via diffusion (extracellular to p	Mg2+[e] = Mg2+[p]	transport : ion	1	1	-	-	-	-	1
R-5074	magnesium ABC transporter	Mg2+[p] + ATP + H2O = Mg2+ + phosphate + ADP	transport : ion	1	1	0	0	-	-	1
R-5075	calcium transport via diffusion (extracellular to perip	Ca2+[e] = Ca2+[p]	transport : ion	1	1	-	-	-	-	1
R-5076	calcium ABC transporter	Ca2+[p] + ATP + H2O = Ca2+ + phosphate + ADP	transport : ion	1	1	-	-	-	-	1
R-5077	copper transport via diffusion (extracellular to peripl	Cu2+[e] = Cu2+[p]	transport : ion	1	1	-	-	-	-	1
R-5078	copper ABC transporter	Cu2+[p] + ATP + H2O = Cu2+ + phosphate + ADP	transport : ion	1	1	0	0	-	-	1
R-5079	copper-exporting ATPase	Cu2+ + ATP + H2O = Cu2+[p] + phosphate + ADP	transport : ion	0	0	0	0	-	-	0
R-5080	manganese transport via diffusion (extracellular to p	Mn2+[e] = Mn2+[p]	transport : ion	1	1	-	-	-	-	1
R-5081	manganese:proton symport	Mn2+[p] + H+[p] = Mn2+ + H+	transport : ion	1	1	0	0	-	-	1
R-5082	iron:proton symport	Fe2+[p] + H+[p] = Fe2+ + H+	transport : ion	0	0	0	0	-	1	0
R-5083	cobalt-exporting ATPase	Co2+ + ATP + H2O = Co2+[p] + phosphate + ADP	transport : ion	0	0	0	0	-	-	0
R-5084	zinc-exporting ATPase	Zn2+ + ATP + H2O = Zn2+[p] + phosphate + ADP	transport : ion	0	0	0	0	-	-	0
R-5085	cadmium-exporting ATPase	Cd2+ + ATP + H2O = Cd2+[p] + phosphate + ADP	transport : ion	0	0	0	0	-	-	0
R-5086	zinc transport via diffusion (extracellular to periplasr	Zn2+[e] = Zn2+[p]	transport : ion	1	1	-	-	-	-	1
R-5087	zinc ABC transporter	Zn2+[p] + ATP + H2O = Zn2+ + phosphate + ADP	transport : ion	1	1	-	-	-	-	1
R-5088	chloride transport via diffusion (extracellular to peri	Cl-[e] = Cl-[p]	transport : ion	1	1	-	-	-	-	1
R-5089	chloride channel	Cl-[p] = Cl-	transport : ion	1	1	0	0	-	-	1
R-5090	sodium/calcium exchanger	Na+[p] + Ca2+ = Na+ + Ca2+[p]	transport : ion	1	1	-	-	-	-	1
R-5091	D-arabinose transport via diffusion (extracellular to	D-arabinose[e] = D-arabinose[p]	transport : pentose	0	0	-	-	-	-	0
R-5092	L-arabinose transport via diffusion (extracellular to p	L-arabinose[e] = L-arabinose[p]	transport : pentose	0	0	-	-	-	-	0
R-5093	D-arabinonate transport via diffusion (extracellular t	D-arabinonate[e] = D-arabinonate[p]	transport : pentose	0	0	-	-	-	-	0
R-5094	L-arabinonate transport via diffusion (extracellular to	L-arabinonate[e] = L-arabinonate[p]	transport : pentose	0	0	-	-	-	-	0
R-5095	L-lyxose transport via diffusion (extracellular to perip	L-lyxose[e] = L-lyxose[p]	transport : pentose	0	0	-	-	-	-	0
R-5096	D-xylose transport via diffusion (extracellular to peri	D-xylose[e] = D-xylose[p]	transport : pentose	0	0	-	-	-	-	0
R-5097	D-xylonate transport via diffusion (extracellular to pe	D-xylonate[e] = D-xylonate[p]	transport : pentose	0	0	-	-	-	-	0
R-5098	L-lyxonate transport via diffusion (extracellular to pe	L-lyxonate[e] = L-lyxonate[p]	transport : pentose	0	0	-	-	-	-	0
R-5099	D-ribonate transport via diffusion (extracellular to pe	D-ribonate[e] = D-ribonate[p]	transport : pentose	0	0	-	-	-	-	0
R-5100	2-deoxy-D-ribonate transport via diffusion (extracellu	2-deoxy-D-ribonate[e] = 2-deoxy-D-ribonate[p]	transport : pentose	0	0	-	-	-	-	0
R-5101	acetoacetate symport (periplasm to cytoplasm)	acetoacetate[p] + H+[p] = acetoacetate + H+	transport : organic acid	0	0	-	-	-	-	0
R-5102	acetoacetate transport via diffusion (extracellular to	acetoacetate[e] = acetoacetate[p]	transport : organic acid	0	0	-	-	-	-	0
R-5103	propanoate symport (periplasm to cytoplasm)	propanoate[p] + H+[p] = propanoate + H+	transport : organic acid	0	0	-	-	-	-	0
R-5104	propanoate transport via diffusion (extracellular to p	propanoate[e] = propanoate[p]	transport : organic acid	0	0	-	-	-	-	0
R-5105	3-O-b-D-galactopyranosyl-D-arabinose transport via	3-O-b-D-galactopyranosyl-D-arabinose[e] = 3-O-b-D-galactopyran	transport : polysugar	0	0	-	-	-	-	0
R-5106	3-O-b-D-galactopyranosyl-D-arabinonate transport v	3-O-b-D-galactopyranosyl-D-arabinonate[e] = 3-O-b-D-galactopyr	transport : polysugar	0	0	-	-	-	-	0
R-5107	short-chain fatty acid (decanoate) transporter (perip	decanoate[p] + H+[p] = decanoate + H+	transport : fatty acid	0	0	-	-	-	-	0
R-5108	decanoate transport via diffusion (extracellular to pe	decanoate[e] = decanoate[p]	transport : fatty acid	0	0	-	-	-	-	0
R-5109	methylamine transport via diffusion (extracellular to	methylamine[e] = methylamine[p]	transport : one carbon compound	0	0	-	-	-	-	0
R-5110	ethylamine transport via diffusion (extracellular to p	ethylamine[e] = ethylamine[p]	transport : ethylamine	0	0	-	-	-	-	0
R-5111	acetaldehyde transport via diffusion (periplasm to cy	acetaldehyde[p] = acetaldehyde	transport : aldehyde	0	0	-	-	-	-	0
R-5112	ethanol transport via diffusion (extracellular to perip	ethanol[e] = ethanol[p]	transport : alchool	0	0	-	-	-	-	0
R-5113	L-tartrate transport via diffusion (extracellular to per	L-tartrate[e] = L-tartrate[p]	transport : organic acid	0	0	-	-	-	-	0
R-1030	D-lactate dehydrogenase (cytochrome)	D-lactate + 2 oxidized-cytochrome-c = pyruvate + 2 H+ + 2 reduce	lactate metabolism	0	0	-1	0	-	-	0
R-1031	S-lactate dehydrogenase	(S)-lactate + NAD+ = pyruvate + NADH + H+	lactate metabolism	0	0	-	-	-	-	0
R-1032	1,2-propanediol dehydrogenase	1,2-propanediol[p] + 4 oxidized-cytochrome-c = (S)-lactate[p] + 4	1,2-propanediol metabolism	0	0	0	0	-	-	0
EX-0069	exchange flux : (S)-lactate	= (S)-lactate[e]	exchange with environment	0	0	-	-	-	-	0
EX-0070	exchange flux : 1,2-propanediol	= 1,2-propanediol[e]	exchange with environment	0	0	-	-	-	-	0
R-5117	1,2-propanediol transport via diffusion (extracellular	1,2-propanediol[e] = 1,2-propanediol[p]	transport : alchool	0	0	-	-	-	-	0
R-5118	(S)-lactate transport via diffusion (extracellular to pe	(S)-lactate[e] = (S)-lactate[p]	transport : organic acid	0	0	-	-	-	-	0
R-5119	(S)-lactate transport (periplasm to cytoplasm)	(S)-lactate[p] + H+[p] = (S)-lactate	transport : organic acid	0	0	-	-	-	-	0
R-5114	L-tartrate transporter	L-tartrate[p] + H+[p] = L-tartrate + H+	transport : organic acid	0	0	0	0	-	-	0
R-5115	glycerone transport via diffusion (extracellular to per	glycerone[e] = glycerone[p]	transport : triose	0	0	-	-	-	-	0
R-5116	glycerone transport via diffusion (periplasm to cytop	glycerone[p] = glycerone	transport : triose	0	0	-	-	-	-	0
				**717**	**723**					**724**
