# Supplementary material for: Co-Consumption of Methanol and Succinate by Methylobacterium extorquens AM1
Source: PLoS One. 2012 Nov 1;7(11):e48271. doi: 10.1371/journal.pone.0048271 (PMC3486813; doi:10.1371/journal.pone.0048271)
Supplement: Table S3 — Fluxes solution of the feasibility analysis. (PDF) [file pone.0048271.s008.pdf]

Results of the Flux Feasability analysis
****

A-GAM	9.666000
EX-0001	5.203600
EX-0002	9.716900
EX-0003	1.730800
EX-0004	-8.644100
A-13CCO2	5.100000
EX-0007	0.000000
EX-0008	0.218700
EX-0009	2.671900
EX-0010	0.000000
EX-0011	0.000000
EX-0012	0.000000
EX-0013	0.000000
EX-0014	0.000000
EX-0015	0.000000
EX-0016	0.000000
EX-0017	0.000000
EX-0019	0.000000
EX-0020	0.014100
EX-0021	0.000700
EX-0022	0.000000
EX-0023	0.000000
EX-0024	0.000000
EX-0025	0.000000
EX-0026	-15.252300
EX-0027	0.000000
EX-0028	0.000000
EX-0029	0.000000
EX-0030	0.000000
EX-0031	0.000000
EX-0032	0.000000
EX-0033	0.000000
EX-0034	0.109800
EX-0035	0.006000
EX-0036	0.024800
EX-0037	0.000000
EX-0038	0.001400
EX-0039	0.000600
EX-0040	0.033200
EX-0041	0.001500
EX-0042	0.000900
EX-0043	0.000600
EX-0044	0.000600
EX-0045	0.000600
EX-0046	0.000900
EX-0047	0.000000
EX-0048	0.000000
EX-0049	0.000000
EX-0050	0.000000
EX-0051	0.000000
EX-0052	0.000000
EX-0053	0.000000
EX-0054	0.000000
EX-0055	0.000000
EX-0056	0.000000
EX-0057	0.000000
EX-0058	0.000000
EX-0059	0.000000
EX-0060	0.000000
EX-0061	0.000000
EX-0062	0.000000
EX-0063	0.000000
EX-0064	0.000000
EX-0065	0.000000
EX-0066	0.000000
EX-0067	0.000000
EX-0068	0.000000
EX-0069	0.000000
EX-0070	0.000000
mue	0.180000
NGAM	9.500000

R-0001	5.203600
R-0002	0.000000
R-0003	5.203600
R-0004	2.283500
R-0005	2.920100
R-0006	5.203600
R-0007	5.203600
R-0008	5.203600
R-0011	5.100000
R-0012	0.103900
R-0013	0.016400
R-0014	0.016400
R-0015	0.000000
R-0016	0.252500
R-0017	0.252500
R-0018	0.252500
R-0019	-0.657700
R-0020	0.000000
R-0021	2.781800
R-0022	0.254400
R-0023	0.254400
R-0024	0.050000
R-0025	0.050000
R-0026	0.000000
R-0027	0.049900
R-0028	0.000000
R-0029	0.000000
R-0031	0.000000
R-0032	0.000000
R-0033	0.000000
R-0034	0.000000
R-0035	0.000000
R-0036	0.000000
R-0037	0.000000
R-0038	0.000000
R-0044	0.000000
R-0045	-0.000000
R-0046	-0.000000
R-0047	0.477000
R-0048	0.476800
R-0049	0.476800
R-0050	0.257700
R-0051	0.210000
R-0052	2.927900
R-0053	3.036600
R-0054	0.000000
R-0055	0.601000
R-0056	1.920300
R-0057	0.000000
R-0058	0.910000
R-0059	0.590500
R-0060	-0.590500
R-0061	0.276200
R-0062	0.260400
R-0063	0.260400
R-0064	0.185700
R-0065	0.000000
R-0066	0.000100
R-0067	0.000100
R-0068	0.000000
R-0069	-0.068700
R-0070	-0.068500
R-0071	-0.053500
R-0072	0.053500
R-0073	-0.015200
R-0074	0.015200
R-0075	0.083600
R-0076	0.000000
R-0077	0.001200
R-0078	0.000700
R-0079	0.000000
R-0080	0.000000
R-0081	0.000000

R-0082	0.000000
R-0083	0.000000
R-0086	-0.000000
R-0089	0.000000
R-0090	0.000000
R-0094	0.000000
R-0095	0.000000
R-0097	0.000000
R-0098	0.000000
R-0099	0.734500
R-0101	0.014000
R-0102	0.000000
R-0105	0.000000
R-0106	0.000000
R-0107	0.000000
R-0108	0.000000
R-0109	0.000000
R-0110	0.000000
R-0111	0.000000
R-0112	0.000700
R-0113	0.071600
R-0114	0.075600
R-0115	0.037700
R-0116	0.075600
R-0117	0.071500
R-0118	0.037500
R-0119	0.071600
R-0120	0.071600
R-0121	0.071600
R-0122	0.150000
R-0123	0.037700
R-0124	0.150000
R-0125	0.037700
R-0126	0.150000
R-0127	0.037700
R-0128	0.071600
R-0129	-0.071600
R-0130	-0.178600
R-0131	0.000000
R-0132	0.000000
R-0133	0.068800
R-0134	0.000000
R-0135	0.000000
R-0136	0.000000
R-0137	0.000000
R-0138	0.000000
R-0139	0.000000
R-0140	0.000000
R-0141	0.000000
R-0142	0.000000
R-0143	0.000000
R-0145	0.000000
R-0146	0.000000
R-0147	0.000000
R-0148	0.000000
R-0149	0.006400
R-0150	0.006400
R-0151	0.000000
R-0152	0.000000
R-0153	0.062400
R-0154	0.000000
R-0155	0.000000
R-0156	0.000000
R-0157	0.000000
R-0158	0.000000
R-0159	0.000000
R-0160	0.000000
R-0161	0.000000
R-0162	0.000000
R-0163	0.109800
R-0164	0.109800
R-0165	0.109800
R-0166	0.109800

R-0167	0.000000
R-0168	-0.038300
R-0169	0.024500
R-0170	0.000000
R-0171	0.176600
R-0174	0.000000
R-0175	0.000000
R-0176	0.000000
R-0177	0.038300
R-0178	0.038300
R-0179	0.000100
R-0180	0.000100
R-0181	0.000100
R-0182	0.000000
R-0183	0.000100
R-0184	0.000100
R-0185	0.038100
R-0186	0.000000
R-0187	0.013600
R-0188	0.024500
R-0189	0.038300
R-0190	0.038300
R-0191	0.000100
R-0192	0.000000
R-0193	0.012200
R-0194	0.038300
R-0195	0.038300
R-0196	0.000000
R-0197	0.000000
R-0198	0.000000
R-0199	0.000000
R-0200	0.000700
R-0201	0.010200
R-0202	0.000000
R-0203	0.000000
R-0204	0.010100
R-0205	0.000900
R-0206	0.010200
R-0207	0.010900
R-0208	0.010900
R-0209	0.000000
R-0210	0.046400
R-0211	0.046400
R-0212	0.046400
R-0213	0.046400
R-0214	0.046400
R-0215	0.046400
R-0216	0.046400
R-0217	-0.051500
R-0218	0.000000
R-0219	0.012200
R-0220	0.012200
R-0221	0.012200
R-0222	0.012200
R-0223	0.012200
R-0224	0.012200
R-0225	0.012200
R-0226	0.012200
R-0227	0.012200
R-0228	0.000000
R-0229	0.000000
R-0230	0.000000
R-0231	0.000000
R-0232	0.000000
R-0233	0.000000
R-0234	0.000000
R-0235	0.000000
R-0236	0.001300
R-0237	0.000600
R-0238	0.000200
R-0239	0.000200
R-0240	0.000000
R-0241	0.150200

R-0242	0.000000
R-0243	0.000000
R-0244	0.000000
R-0245	0.000000
R-0249	0.093600
R-0251	0.000000
R-0252	0.000000
R-0253	0.000000
R-0254	0.000000
R-0255	0.037700
R-0256	0.000000
R-0257	0.103900
R-0258	0.055400
R-0259	0.055800
R-0260	0.093600
R-0261	0.150200
R-0262	0.211000
R-0263	0.319500
R-0264	0.319500
R-0265	0.319500
R-0266	0.000000
R-0267	0.000000
R-0268	0.014500
R-0270	0.000000
R-0272	0.000600
R-0273	0.000600
R-0274	0.000600
R-0275	0.009400
R-0276	0.000000
R-0277	0.000000
R-0278	0.000000
R-0279	0.000000
R-0280	0.000400
R-0281	0.000400
R-0282	0.000400
R-0283	0.006400
R-0284	0.000400
R-0285	0.000400
R-0286	0.000400
R-0287	0.006600
R-0289	0.000200
R-0290	0.000200
R-0291	0.000200
R-0292	0.002800
R-0294	0.000000
R-0295	0.000000
R-0298	0.000000
R-0299	0.000000
R-0300	0.000000
R-0301	0.000000
R-0302	0.000000
R-0303	0.000000
R-0304	0.000000
R-0305	0.000000
R-0308	0.000000
R-0309	0.000000
R-0310	0.000000
R-0311	0.000000
R-0312	0.000000
R-0313	0.000000
R-0314	0.000000
R-0315	0.000000
R-0317	0.000000
R-0318	0.000000
R-0319	0.000000
R-0320	0.000000
R-0322	0.000000
R-0323	0.000000
R-0324	0.000000
R-0325	0.000000
R-0327	0.000000
R-0328	0.000000
R-0329	0.000000

R-0330	0.000000
R-0331	0.000000
R-0332	0.000000
R-0334	0.000600
R-0335	0.000600
R-0336	0.000600
R-0337	0.009400
R-0338	0.176500
R-0339	0.022400
R-0340	0.022400
R-0341	0.022400
R-0342	0.022400
R-0343	0.022400
R-0344	0.002400
R-0345	0.002400
R-0346	0.002300
R-0347	0.001200
R-0348	0.020000
R-0349	0.020000
R-0350	0.020000
R-0351	0.018700
R-0352	0.022400
R-0353	0.022400
R-0354	0.022400
R-0355	0.022400
R-0356	0.002400
R-0358	0.002400
R-0359	0.002300
R-0360	0.001200
R-0361	0.020000
R-0362	0.020000
R-0363	0.020000
R-0364	0.018700
R-0365	0.022400
R-0366	0.020000
R-0367	0.022400
R-0368	0.022400
R-0369	0.022400
R-0370	0.002400
R-0371	0.002300
R-0372	0.002300
R-0373	0.001200
R-0374	0.020000
R-0375	0.020000
R-0376	0.020000
R-0377	0.018700
R-0378	0.022400
R-0379	0.022400
R-0380	0.022400
R-0381	0.002400
R-0382	0.002400
R-0383	0.002300
R-0384	0.002300
R-0385	0.001200
R-0386	0.020000
R-0387	0.020000
R-0388	0.020000
R-0389	0.018700
R-0390	0.000000
R-0391	0.006000
R-0392	0.199800
R-0393	-0.384400
R-0394	0.021800
R-0395	0.000000
R-0396	0.049600
R-0397	1.768500
R-0398	0.000000
R-0399	0.000000
R-0400	0.000000
R-0401	0.037700
R-0402	0.000000
R-0403	1.455900
R-0404	0.075000

R-0405	0.003000
R-0406	0.003000
R-0407	0.000000
R-0408	0.000200
R-0409	0.000000
R-0410	0.056200
R-0411	0.000000
R-0412	0.000000
R-0413	0.000000
R-0414	0.000000
R-0415	0.043400
R-0416	0.056000
R-0417	0.043400
R-0418	0.000000
R-0419	0.000000
R-0420	0.000000
R-0421	0.000000
R-0422	0.006000
R-0423	0.006000
R-0424	-0.006000
R-0425	0.000000
R-0426	0.001200
R-0427	0.001200
R-0428	0.001200
R-0429	0.000000
R-0430	-0.199800
R-0431	0.028000
R-0432	0.031600
R-0433	0.043400
R-0434	0.044600
R-0435	0.000000
R-0436	0.198600
R-0437	0.043400
R-0438	0.028000
R-0439	0.037600
R-0440	0.000000
R-0441	0.000000
R-0442	0.000000
R-0443	27.123200
R-0444	5.203600
R-0445	7.960200
R-0446	0.000000
R-0447	11.268100
R-0448	0.000000
R-0449	0.037700
R-0450	0.037700
R-0451	0.037700
R-0452	0.037700
R-0453	0.037600
R-0454	0.037600
R-0455	0.049800
R-0456	0.049800
R-0457	0.021800
R-0458	0.021900
R-0459	0.000000
R-0460	0.106400
R-0461	0.006300
R-0462	0.000000
R-0463	0.008700
R-0464	0.112600
R-0465	0.002800
R-0466	0.006000
R-0467	0.043900
R-0469	0.024400
R-0470	0.000000
R-0471	0.008700
R-0472	0.002800
R-0474	0.006000
R-0475	0.030800
R-0476	1.309700
R-0477	0.031600
R-0478	0.031600
R-0479	0.031600

R-0480	0.031600
R-0481	0.015000
R-0482	0.031600
R-0483	0.002800
R-0484	0.002800
R-0485	0.002800
R-0486	0.002800
R-0487	0.000000
R-0489	0.000000
R-0490	0.000000
R-0491	0.000000
R-0492	0.000000
R-0493	0.000000
R-0494	0.000000
R-0495	0.000000
R-0496	0.000000
R-0497	0.000000
R-0498	0.000000
R-0499	0.000000
R-0500	0.000000
R-0501	0.000000
R-0502	0.000000
R-0503	0.000000
R-0504	0.000000
R-0505	0.000000
R-0506	0.000100
R-0507	0.001100
R-0508	0.001100
R-0509	0.001100
R-0510	0.001100
R-0511	0.001100
R-0512	0.000200
R-0513	0.000900
R-0514	0.000200
R-0515	0.000200
R-0516	0.000200
R-0517	0.000100
R-0518	0.000100
R-0519	0.000100
R-0520	0.000100
R-0521	0.000100
R-0522	0.000100
R-0523	0.013600
R-0524	0.024500
R-0525	0.013600
R-0526	0.199800
R-0527	0.012200
R-0529	0.000600
R-0530	0.000600
R-0531	0.000600
R-0532	0.009400
R-0534	0.000600
R-0535	0.000600
R-0536	0.000600
R-0537	0.009400
R-0538	0.002800
R-0539	0.000000
R-0540	0.049900
R-0541	0.049900
R-0542	0.044900
R-0543	0.044900
R-0544	0.056200
R-0545	0.056200
R-0546	0.056200
R-0547	0.000000
R-0548	0.051500
R-0549	0.051500
R-0550	0.000000
R-0551	0.051500
R-0552	0.000000
R-0553	0.000100
R-0554	0.000000
R-0555	0.000000

R-0556	0.000000
R-0557	0.000000
R-0558	0.000000
R-0559	0.000000
R-0560	0.033200
R-0561	0.000000
R-0562	0.000000
R-0563	0.000000
R-0565	0.000000
R-0566	0.000000
R-0567	0.000000
R-0568	-0.000000
R-0569	0.000000
R-0570	0.000800
R-0571	0.000000
R-0572	0.000000
R-0573	0.000000
R-0574	0.000000
R-0576	0.000000
R-0577	0.000000
R-0578	0.000000
R-0579	0.000000
R-0580	0.000000
R-0581	0.000100
R-0582	0.000000
R-0583	0.000000
R-0584	0.000000
R-0585	0.000000
R-0586	0.000000
R-0587	0.000000
R-0588	0.000000
R-0589	0.000000
R-0590	0.000000
R-0591	0.000000
R-0593	0.000000
R-0594	0.000000
R-0595	0.000000
R-0596	0.008200
R-0597	0.000000
R-0598	0.000000
R-0599	0.000000
R-0600	0.000000
R-0601	0.000000
R-0602	0.000000
R-0603	0.000000
R-0604	0.000000
R-0605	0.000000
R-0606	3.117900
R-0607	0.000000
R-0608	-0.000100
R-0609	0.000000
R-0610	0.000000
R-0611	0.000000
R-0612	0.000000
R-0613	0.000000
R-0614	0.000000
R-0615	0.000000
R-0616	0.000100
R-0617	0.002800
R-0618	0.000100
R-0619	0.000000
R-0620	0.000000
R-0621	0.000000
R-0622	0.000000
R-0623	0.000000
R-0624	0.000000
R-0625	0.000000
R-0626	0.000000
R-0627	0.002800
R-0628	0.000000
R-0629	0.000000
R-0630	0.000000
R-0631	0.000000

R-0632	0.000000
R-0633	0.000000
R-0634	0.000000
R-0635	0.000000
R-0636	1.502100
R-0637	0.000000
R-0638	0.000000
R-0639	0.000000
R-0640	0.000000
R-0641	0.000000
R-0642	0.000000
R-0643	0.000000
R-0644	0.000000
R-0645	0.000000
R-0646	0.000000
R-0647	0.000000
R-0648	0.000000
R-0649	0.000000
R-0650	0.000000
R-0651	0.000000
R-0652	0.000000
R-0653	0.000000
R-0654	0.000000
R-0655	0.000000
R-0656	0.000000
R-0657	0.000000
R-0658	0.000000
R-0659	0.000000
R-0660	0.000000
R-0661	0.000000
R-0662	0.002800
R-0663	0.013300
R-0664	0.013300
R-0665	0.013300
R-0666	0.000000
R-0667	0.000000
R-0669	0.000000
R-0670	0.001100
R-0671	0.000000
R-0672	0.000000
R-0673	0.000000
R-0674	0.002800
R-0675	0.000000
R-0676	0.000000
R-0677	0.000000
R-0679	0.000000
R-0680	0.000000
R-0681	0.000000
R-0682	0.000000
R-0683	0.000000
R-0684	0.000000
R-0685	0.000100
R-0686	0.000100
R-0687	0.000000
R-0688	0.000000
R-0689	0.000000
R-0690	0.000000
R-0691	0.000100
R-0692	0.000100
R-0693	0.000000
R-0694	0.000000
R-0695	0.000000
R-0696	0.000000
R-0697	0.000000
R-0698	0.000000
R-0699	0.000000
R-0700	0.251500
R-0701	0.000000
R-0702	0.001200
R-0703	0.000000
R-0704	0.000000
R-0705	0.000000
R-0706	0.000000

R-0707	0.000000
R-0708	0.000000
R-0709	0.000000
R-0710	0.000000
R-0711	0.000000
R-0712	0.000000
R-0713	0.000000
R-0714	0.000000
R-0715	0.000000
R-0716	0.000000
R-0717	0.000100
R-0718	0.000000
R-0719	0.000000
R-0720	0.000000
R-0721	0.000000
R-0722	0.000000
R-0724	0.000000
R-0725	0.000000
R-0726	0.000000
R-0727	0.000000
R-0728	0.000000
R-0729	0.000000
R-0730	0.000200
R-0731	0.000100
R-0732	0.000100
R-0733	0.000100
R-0736	0.016600
R-0737	0.000000
R-0738	0.000000
R-0739	0.000100
R-0740	0.000100
R-0741	0.000000
R-0742	0.000000
R-0743	0.000000
R-0744	0.000000
R-0745	0.000000
R-0746	0.000000
R-0747	0.000000
R-0748	0.000000
R-0749	0.000000
R-0750	0.000000
R-0751	0.000000
R-0752	0.000000
R-0753	0.000000
R-0754	0.000000
R-0755	0.000000
R-0756	0.000000
R-0757	0.000000
R-0758	0.000000
R-0759	0.000000
R-0760	0.000000
R-0762	0.000000
R-0763	0.000000
R-0764	0.000000
R-0765	0.000000
R-0766	0.000000
R-0767	0.000000
R-0768	0.000000
R-0769	0.000000
R-0770	0.000000
R-0771	0.000000
R-0772	0.000000
R-0773	0.000000
R-0774	0.000000
R-0775	0.000000
R-0776	-0.000000
R-0777	0.000000
R-0778	0.000000
R-0779	0.000000
R-0780	0.000000
R-0781	0.000000
R-0782	0.000000
R-0783	0.000200

R-0784	0.000100
R-0785	0.000000
R-0786	0.000000
R-0787	0.001200
R-0788	0.000000
R-0789	0.000000
R-0790	0.000000
R-0791	0.000000
R-0792	0.000000
R-0793	0.000000
R-0794	0.000000
R-0795	0.000000
R-0796	0.000000
R-0797	0.000000
R-0798	0.000000
R-0799	0.000000
R-0800	0.000000
R-0801	0.000000
R-0802	0.000000
R-0803	0.000000
R-0804	0.000000
R-0805	0.000000
R-0806	0.002800
R-0807	0.002800
R-0808	0.000000
R-0809	0.000000
R-0810	0.000000
R-0811	0.000000
R-0812	0.000000
R-0813	0.000000
R-0814	0.000100
R-0815	0.000000
R-0816	0.000000
R-0817	0.000000
R-0819	0.000000
R-0820	0.000000
R-0821	0.000000
R-0822	0.000000
R-0823	0.000000
R-0824	0.000000
R-0825	0.000000
R-0826	0.000000
R-0827	0.000000
R-0828	0.000000
R-0829	0.000000
R-0830	0.000000
R-0831	0.000000
R-0832	0.000000
R-0833	0.000000
R-0834	0.000000
R-0835	0.000000
R-0837	0.000000
R-0838	-0.000000
R-0839	0.002800
R-0840	0.000000
R-0841	0.000000
R-0842	0.000000
R-0843	0.000000
R-0844	0.000000
R-0845	0.000000
R-0846	0.000000
R-0847	0.000000
R-0848	0.000000
R-0849	0.000000
R-0850	0.000000
R-0851	0.000000
R-0852	0.000000
R-0853	0.000000
R-0854	0.000000
R-0855	0.000000
R-0856	0.000000
R-0857	0.000000
R-0858	0.000000

R-0859	0.000000
R-0860	0.000000
R-0861	0.000000
R-0862	0.000000
R-0863	0.000000
R-0864	0.000000
R-0865	0.000000
R-0866	0.000000
R-0867	0.000000
R-0868	0.000000
R-0869	0.000000
R-0870	0.000000
R-0871	0.000000
R-0872	0.000000
R-0873	0.000100
R-0874	0.000100
R-0875	0.000000
R-0876	0.000000
R-0877	0.000000
R-0878	0.000000
R-0879	0.000000
R-0880	0.000000
R-0881	0.000000
R-0882	0.000000
R-0883	0.000000
R-0884	0.000000
R-0885	0.000000
R-0886	0.000000
R-0887	0.000000
R-0888	0.000000
R-0889	0.000000
R-0890	0.000000
R-0891	0.000000
R-0892	0.000000
R-0893	0.000000
R-0894	0.000000
R-0895	0.000000
R-0896	0.000000
R-0897	0.000000
R-0898	0.000000
R-0899	0.000000
R-0900	0.000000
R-0901	0.000000
R-0902	0.000000
R-0903	0.000000
R-0904	0.000000
R-0905	0.000000
R-0906	0.000000
R-0907	0.000000
R-0908	0.000000
R-0909	0.000000
R-0910	0.000000
R-0911	0.000000
R-0913	0.000000
R-0914	0.000000
R-0915	0.000000
R-0916	0.000000
R-0917	0.000000
R-0918	0.000000
R-0919	0.000000
R-0920	0.000000
R-0921	0.000000
R-0922	0.000000
R-0923	0.000000
R-0924	0.000000
R-0925	0.000000
R-0926	0.000000
R-0927	0.000000
R-0928	0.000000
R-0929	0.000000
R-0930	0.002800
R-0933	0.000200
R-0934	0.000200

R-0935	0.000200
R-0936	0.002800
R-0937	0.000100
R-0938	0.000000
R-0939	0.000100
R-0940	0.000000
R-0941	0.000000
R-0942	0.000000
R-0943	0.000000
R-0944	0.000000
R-0945	0.000000
R-0946	0.000000
R-0947	0.000000
R-0965	0.046400
R-0966	0.000000
R-0967	0.000000
R-0968	0.000000
R-0969	0.000000
R-0970	0.000000
R-0971	0.000000
R-0972	0.000000
R-0973	0.000000
R-0974	0.000000
R-0975	0.000000
R-0976	0.000000
R-0977	0.000000
R-0978	0.000700
R-0979	0.000000
R-0980	0.000000
R-0981	0.000000
R-0982	0.000000
R-0983	0.013300
R-0984	0.000000
R-0985	0.013300
R-0988	0.000000
R-0989	0.000000
R-0990	0.000000
R-0991	0.000000
R-0992	0.000000
R-0993	0.000100
R-0994	0.000000
R-0995	0.000100
R-0996	0.002800
R-0997	0.000100
R-0998	0.000000
R-0999	0.000000
R-1000	0.000000
R-1001	0.000000
R-1002	0.000000
R-1003	0.000000
R-1004	0.000000
R-1005	0.000000
R-1006	0.000400
R-1007	0.000000
R-1008	0.000000
R-1009	0.000000
R-1010	0.000000
R-1011	0.000000
R-1012	0.000000
R-1013	0.000000
R-1014	0.000000
R-1015	0.000000
R-1016	0.000000
R-1017	0.000000
R-1018	0.000000
R-1019	0.000000
R-1020	0.000000
R-1021	0.000000
R-1022	0.000000
R-1023	0.000000
R-1024	0.000000
R-1025	0.000000
R-1026	0.000000

R-1027	0.000000
R-1028	0.000000
R-1029	0.000000
R-1030	0.000000
R-1031	0.000000
R-1032	0.000000
R-1033	0.000700
R-5001	5.203600
R-5002	5.203600
R-5003	-8.644100
R-5004	-8.644100
R-5005	9.716900
R-5006	9.716900
R-5007	0.000000
R-5008	0.218700
R-5009	0.218700
R-5010	1.730800
R-5011	1.730800
R-5012	2.671900
R-5013	2.671900
R-5014	0.000000
R-5015	0.000000
R-5016	0.000000
R-5017	0.000000
R-5018	0.000000
R-5019	0.000000
R-5020	0.000000
R-5021	0.000000
R-5022	0.000000
R-5023	0.000000
R-5024	0.000000
R-5025	0.000000
R-5026	0.000000
R-5027	0.000000
R-5028	0.000000
R-5029	0.000000
R-5030	0.000000
R-5031	0.000000
R-5032	0.000000
R-5033	0.014100
R-5034	0.000700
R-5035	0.000700
R-5036	0.000000
R-5037	0.013300
R-5038	0.000000
R-5039	0.000000
R-5040	0.000000
R-5041	0.000000
R-5042	0.000000
R-5043	0.000000
R-5044	0.000000
R-5045	0.000000
R-5046	-15.252300
R-5047	-15.252300
R-5048	0.000000
R-5049	0.000000
R-5050	0.000000
R-5051	0.000000
R-5052	0.000000
R-5053	0.000000
R-5055	0.000000
R-5056	0.000000
R-5058	0.000000
R-5060	0.000000
R-5061	0.000000
R-5062	0.000000
R-5063	0.000000
R-5064	0.024800
R-5065	0.000000
R-5066	0.000000
R-5067	0.001400
R-5068	0.001400
R-5069	0.000600

R-5070	0.000600
R-5071	0.000000
R-5072	0.033200
R-5073	0.001500
R-5074	0.001500
R-5075	0.000900
R-5076	0.000900
R-5077	0.000600
R-5078	0.000600
R-5079	0.000000
R-5080	0.000600
R-5081	0.000600
R-5082	0.000000
R-5083	0.000000
R-5084	0.000000
R-5085	0.000000
R-5086	0.000600
R-5087	0.000600
R-5088	0.000900
R-5089	0.000900
R-5090	0.000000
R-5091	0.000000
R-5092	0.000000
R-5093	0.000000
R-5094	0.000000
R-5095	0.000000
R-5096	0.000000
R-5097	0.000000
R-5098	0.000000
R-5099	0.000000
R-5100	0.000000
R-5101	0.000000
R-5102	0.000000
R-5103	0.000000
R-5104	0.000000
R-5105	0.000000
R-5106	0.000000
R-5107	0.000000
R-5108	0.000000
R-5109	0.000000
R-5110	0.000000
R-5111	0.000000
R-5112	0.000000
R-5113	0.000000
R-5114	0.000000
R-5115	0.000000
R-5116	0.000000
R-5117	0.000000
R-5118	0.000000
R-5119	0.000000
