# Supplementary material for: Co-Consumption of Methanol and Succinate by Methylobacterium extorquens AM1
Source: PLoS One. 2012 Nov 1;7(11):e48271. doi: 10.1371/journal.pone.0048271 (PMC3486813; doi:10.1371/journal.pone.0048271)
Supplement: Table S4 — Fluxes solution of the flux variability analysis. (PDF) [file pone.0048271.s009.pdf]

Results of Flux Variability Analysis:
****

A-GAM	MinFlux:	9.666	MaxFlux:	9.666
EX-0001	MinFlux:	5.07737	MaxFlux:	5.2913
EX-0002	MinFlux:	9.4509	MaxFlux:	9.7718
EX-0003	MinFlux:	1.7308	MaxFlux:	1.7308
EX-0004	MinFlux:	-8.6441	MaxFlux:	-8.4301
A-13CCO2-bis	MinFlux:	4.9738	MaxFlux:	5.1
EX-0007	MinFlux:	0	MaxFlux:	0
EX-0008	MinFlux:	0.21866	MaxFlux:	0.21866
EX-0009	MinFlux:	2.65	MaxFlux:	2.671927
EX-0010	MinFlux:	0	MaxFlux:	0
EX-0011	MinFlux:	0	MaxFlux:	0
EX-0012	MinFlux:	0	MaxFlux:	0
EX-0013	MinFlux:	0	MaxFlux:	0
EX-0014	MinFlux:	0	MaxFlux:	0
EX-0015	MinFlux:	0	MaxFlux:	0
EX-0016	MinFlux:	0	MaxFlux:	0
EX-0017	MinFlux:	0	MaxFlux:	0
EX-0019	MinFlux:	0	MaxFlux:	0
EX-0020	MinFlux:	0.014074	MaxFlux:	0.014074
EX-0021	MinFlux:	0.0007326	MaxFlux:	0.0007326
EX-0022	MinFlux:	0	MaxFlux:	0
EX-0023	MinFlux:	0	MaxFlux:	0
EX-0024	MinFlux:	0	MaxFlux:	0
EX-0025	MinFlux:	0	MaxFlux:	0
EX-0026	MinFlux:	-18.6977	MaxFlux:	-14.9341
EX-0027	MinFlux:	0	MaxFlux:	0
EX-0028	MinFlux:	0	MaxFlux:	0
EX-0029	MinFlux:	0	MaxFlux:	0
EX-0030	MinFlux:	0	MaxFlux:	0
EX-0031	MinFlux:	0	MaxFlux:	0
EX-0032	MinFlux:	0	MaxFlux:	0
EX-0033	MinFlux:	3.9528e-005	MaxFlux:	3.9528e-005
EX-0034	MinFlux:	0.10984	MaxFlux:	0.10984
EX-0035	MinFlux:	0.0059739	MaxFlux:	0.0059739
EX-0036	MinFlux:	0.024836	MaxFlux:	0.024836
EX-0037	MinFlux:	0	MaxFlux:	0
EX-0038	MinFlux:	0.0014452	MaxFlux:	0.0014452
EX-0039	MinFlux:	0.00063353	MaxFlux:	0.00063353
EX-0040	MinFlux:	0.033205	MaxFlux:	0.033205
EX-0041	MinFlux:	0.001485	MaxFlux:	0.001485
EX-0042	MinFlux:	0.000891	MaxFlux:	0.000891
EX-0043	MinFlux:	0.000594	MaxFlux:	0.000594
EX-0044	MinFlux:	0.000594	MaxFlux:	0.000594
EX-0045	MinFlux:	0.000594	MaxFlux:	0.000594
EX-0046	MinFlux:	0.000891	MaxFlux:	0.000891
EX-0047	MinFlux:	0	MaxFlux:	0
EX-0048	MinFlux:	0	MaxFlux:	0
EX-0049	MinFlux:	0	MaxFlux:	0
EX-0050	MinFlux:	0	MaxFlux:	0
EX-0051	MinFlux:	0	MaxFlux:	0
EX-0052	MinFlux:	0	MaxFlux:	0
EX-0053	MinFlux:	0	MaxFlux:	0
EX-0054	MinFlux:	0	MaxFlux:	0
EX-0055	MinFlux:	0	MaxFlux:	0
EX-0056	MinFlux:	0	MaxFlux:	0
EX-0057	MinFlux:	0	MaxFlux:	0
EX-0058	MinFlux:	0	MaxFlux:	0
EX-0059	MinFlux:	0	MaxFlux:	0
EX-0060	MinFlux:	0	MaxFlux:	0
EX-0061	MinFlux:	0	MaxFlux:	0
EX-0062	MinFlux:	0	MaxFlux:	0
EX-0063	MinFlux:	0	MaxFlux:	0
EX-0064	MinFlux:	0	MaxFlux:	0
EX-0065	MinFlux:	0	MaxFlux:	0
EX-0066	MinFlux:	0	MaxFlux:	0
EX-0067	MinFlux:	0	MaxFlux:	0
EX-0068	MinFlux:	0	MaxFlux:	0
EX-0069	MinFlux:	0	MaxFlux:	0
EX-0070	MinFlux:	0	MaxFlux:	0
mue	MinFlux:	0.18	MaxFlux:	0.18
NGAM	MinFlux:	9.5	MaxFlux:	9.5

R-0001	MinFlux:	5.0774	MaxFlux:	5.2913
R-0002	MinFlux:	0	MaxFlux:	0
R-0003	MinFlux:	1.8057	MaxFlux:	5.2913
R-0004	MinFlux:	0	MaxFlux:	3.2766
R-0005	MinFlux:	0	MaxFlux:	5.2913
R-0006	MinFlux:	1.8057	MaxFlux:	5.2913
R-0007	MinFlux:	1.8057	MaxFlux:	5.2913
R-0008	MinFlux:	1.8057	MaxFlux:	5.2913
R-0011	MinFlux:	3.3533	MaxFlux:	5.1
R-0012	MinFlux:	0.1039	MaxFlux:	0.19161
R-0013	MinFlux:	0.016373	MaxFlux:	0.10408
R-0014	MinFlux:	0.016373	MaxFlux:	0.10408
R-0015	MinFlux:	0	MaxFlux:	0.087709
R-0016	MinFlux:	0.25253	MaxFlux:	0.34024
R-0017	MinFlux:	0.25253	MaxFlux:	0.34024
R-0018	MinFlux:	0.25253	MaxFlux:	0.34024
R-0019	MinFlux:	-2.464	MaxFlux:	-0.56998
R-0020	MinFlux:	0	MaxFlux:	3.4934
R-0021	MinFlux:	-2.632	MaxFlux:	3.0621
R-0022	MinFlux:	0	MaxFlux:	3.7479
R-0023	MinFlux:	-0.0032258	MaxFlux:	0.34216
R-0024	MinFlux:	0.049987	MaxFlux:	0.37344
R-0025	MinFlux:	0.049987	MaxFlux:	0.37344
R-0026	MinFlux:	0	MaxFlux:	0
R-0027	MinFlux:	0.049929	MaxFlux:	0.049929
R-0028	MinFlux:	0	MaxFlux:	0
R-0029	MinFlux:	0	MaxFlux:	0
R-0031	MinFlux:	0	MaxFlux:	0
R-0032	MinFlux:	4.6094e-005	MaxFlux:	0.3235
R-0033	MinFlux:	0	MaxFlux:	0
R-0034	MinFlux:	0	MaxFlux:	0
R-0035	MinFlux:	4.6094e-005	MaxFlux:	0.3235
R-0036	MinFlux:	4.752e-006	MaxFlux:	0.32346
R-0037	MinFlux:	4.752e-006	MaxFlux:	0.32346
R-0038	MinFlux:	4.752e-006	MaxFlux:	0.32346
R-0044	MinFlux:	0	MaxFlux:	0.32346
R-0045	MinFlux:	-3.5165e-005	MaxFlux:	0.32342
R-0046	MinFlux:	-3.5165e-005	MaxFlux:	0.32342
R-0047	MinFlux:	0.21935	MaxFlux:	0.54281
R-0048	MinFlux:	0.21912	MaxFlux:	0.54258
R-0049	MinFlux:	0.21912	MaxFlux:	0.54258
R-0050	MinFlux:	0	MaxFlux:	0.32346
R-0051	MinFlux:	0	MaxFlux:	0.27576
R-0052	MinFlux:	2.696	MaxFlux:	2.9718
R-0053	MinFlux:	2.8047	MaxFlux:	3.0813
R-0054	MinFlux:	0	MaxFlux:	5.6682
R-0055	MinFlux:	0	MaxFlux:	1.226
R-0056	MinFlux:	0	MaxFlux:	5.6682
R-0057	MinFlux:	0	MaxFlux:	5.6682
R-0058	MinFlux:	0.91	MaxFlux:	2.7164
R-0059	MinFlux:	0.59052	MaxFlux:	2.3969
R-0060	MinFlux:	-2.3969	MaxFlux:	-0.59052
R-0061	MinFlux:	0.27618	MaxFlux:	2.0825
R-0062	MinFlux:	0.26045	MaxFlux:	2.0668
R-0063	MinFlux:	0.26045	MaxFlux:	2.0668
R-0064	MinFlux:	0.18572	MaxFlux:	2.3774
R-0065	MinFlux:	0	MaxFlux:	0
R-0066	MinFlux:	0.00011167	MaxFlux:	2.1918
R-0067	MinFlux:	0.00011167	MaxFlux:	2.1918
R-0068	MinFlux:	0	MaxFlux:	0.82728
R-0069	MinFlux:	-0.068686	MaxFlux:	0.48284
R-0070	MinFlux:	-0.34421	MaxFlux:	-0.068452
R-0071	MinFlux:	-0.053504	MaxFlux:	0.22226
R-0072	MinFlux:	-0.22226	MaxFlux:	0.053504
R-0073	MinFlux:	-0.015182	MaxFlux:	0.26058
R-0074	MinFlux:	-0.26058	MaxFlux:	0.015182
R-0075	MinFlux:	0.083634	MaxFlux:	0.083634
R-0076	MinFlux:	0	MaxFlux:	0
R-0077	MinFlux:	0.0011503	MaxFlux:	0.0011503
R-0078	MinFlux:	0.00065297	MaxFlux:	0.00065297
R-0079	MinFlux:	0	MaxFlux:	1.7467
R-0080	MinFlux:	0	MaxFlux:	1.7467
R-0081	MinFlux:	0	MaxFlux:	0

R-0082	MinFlux:	0	MaxFlux:	0
R-0083	MinFlux:	0	MaxFlux:	0
R-0086	MinFlux:	-1.4731e-005	MaxFlux:	-1.4731e-005
R-0089	MinFlux:	0	MaxFlux:	0
R-0090	MinFlux:	0	MaxFlux:	3.4934
R-0094	MinFlux:	0	MaxFlux:	4.6579
R-0095	MinFlux:	0	MaxFlux:	1.7467
R-0097	MinFlux:	4.6094e-005	MaxFlux:	0.3235
R-0098	MinFlux:	4.6094e-005	MaxFlux:	0.3235
R-0099	MinFlux:	-6.0134	MaxFlux:	4.2279
R-0101	MinFlux:	0.013951	MaxFlux:	1.0257
R-0102	MinFlux:	0	MaxFlux:	0
R-0105	MinFlux:	0	MaxFlux:	1.0117
R-0106	MinFlux:	0	MaxFlux:	0
R-0107	MinFlux:	0	MaxFlux:	0
R-0108	MinFlux:	0	MaxFlux:	0
R-0109	MinFlux:	0	MaxFlux:	0
R-0110	MinFlux:	1.4731e-005	MaxFlux:	1.4731e-005
R-0111	MinFlux:	0	MaxFlux:	0
R-0112	MinFlux:	0.00065297	MaxFlux:	0.00065297
R-0113	MinFlux:	0.071592	MaxFlux:	0.071592
R-0114	MinFlux:	0.075605	MaxFlux:	0.075605
R-0115	MinFlux:	0.037684	MaxFlux:	0.037684
R-0116	MinFlux:	0.075605	MaxFlux:	0.075605
R-0117	MinFlux:	0.07145	MaxFlux:	0.07145
R-0118	MinFlux:	0.037542	MaxFlux:	0.037542
R-0119	MinFlux:	0.071592	MaxFlux:	0.071592
R-0120	MinFlux:	0.071592	MaxFlux:	0.071592
R-0121	MinFlux:	0.071592	MaxFlux:	0.071592
R-0122	MinFlux:	0.14995	MaxFlux:	0.14995
R-0123	MinFlux:	0.037684	MaxFlux:	0.037684
R-0124	MinFlux:	0.14995	MaxFlux:	0.14995
R-0125	MinFlux:	0.037684	MaxFlux:	0.037684
R-0126	MinFlux:	0.14995	MaxFlux:	0.14995
R-0127	MinFlux:	0.037684	MaxFlux:	0.037684
R-0128	MinFlux:	0.071592	MaxFlux:	0.071592
R-0129	MinFlux:	-0.071592	MaxFlux:	-0.071592
R-0130	MinFlux:	-0.17864	MaxFlux:	-0.17864
R-0131	MinFlux:	0	MaxFlux:	0
R-0132	MinFlux:	0	MaxFlux:	0
R-0133	MinFlux:	0.068801	MaxFlux:	0.068801
R-0134	MinFlux:	0	MaxFlux:	0
R-0135	MinFlux:	0	MaxFlux:	0
R-0136	MinFlux:	0	MaxFlux:	0
R-0137	MinFlux:	0	MaxFlux:	0
R-0138	MinFlux:	0	MaxFlux:	0
R-0139	MinFlux:	0	MaxFlux:	0
R-0140	MinFlux:	0	MaxFlux:	0
R-0141	MinFlux:	0	MaxFlux:	0
R-0142	MinFlux:	0	MaxFlux:	0
R-0143	MinFlux:	0	MaxFlux:	0
R-0145	MinFlux:	0	MaxFlux:	0
R-0146	MinFlux:	0	MaxFlux:	0
R-0147	MinFlux:	0	MaxFlux:	0
R-0148	MinFlux:	0	MaxFlux:	0
R-0149	MinFlux:	0.0063939	MaxFlux:	0.0063939
R-0150	MinFlux:	0.0063939	MaxFlux:	0.0063939
R-0151	MinFlux:	0	MaxFlux:	0
R-0152	MinFlux:	0	MaxFlux:	0
R-0153	MinFlux:	0.062407	MaxFlux:	0.062407
R-0154	MinFlux:	0	MaxFlux:	0
R-0155	MinFlux:	0	MaxFlux:	0
R-0156	MinFlux:	0	MaxFlux:	0
R-0157	MinFlux:	0	MaxFlux:	0
R-0158	MinFlux:	0	MaxFlux:	0
R-0159	MinFlux:	0	MaxFlux:	0
R-0160	MinFlux:	0	MaxFlux:	0
R-0161	MinFlux:	0	MaxFlux:	0
R-0162	MinFlux:	0	MaxFlux:	0
R-0163	MinFlux:	0.10984	MaxFlux:	0.10984
R-0164	MinFlux:	0.10984	MaxFlux:	0.10984
R-0165	MinFlux:	0.10984	MaxFlux:	0.10984
R-0166	MinFlux:	0.10984	MaxFlux:	0.10984

R-0167	MinFlux:	0	MaxFlux:	0
R-0168	MinFlux:	-0.038302	MaxFlux:	-0.038302
R-0169	MinFlux:	0.024472	MaxFlux:	0.024472
R-0170	MinFlux:	0	MaxFlux:	0
R-0171	MinFlux:	0.17655	MaxFlux:	0.17655
R-0174	MinFlux:	0	MaxFlux:	0
R-0175	MinFlux:	0	MaxFlux:	0
R-0176	MinFlux:	0	MaxFlux:	0
R-0177	MinFlux:	0.038302	MaxFlux:	0.038302
R-0178	MinFlux:	0.038302	MaxFlux:	0.038302
R-0179	MinFlux:	0.00010872	MaxFlux:	0.00010872
R-0180	MinFlux:	0.00010872	MaxFlux:	0.00010872
R-0181	MinFlux:	0.00010872	MaxFlux:	0.00010872
R-0182	MinFlux:	0	MaxFlux:	0
R-0183	MinFlux:	0.00010872	MaxFlux:	0.00010872
R-0184	MinFlux:	0.00010872	MaxFlux:	0.00010872
R-0185	MinFlux:	0.038099	MaxFlux:	0.038099
R-0186	MinFlux:	1.4731e-005	MaxFlux:	1.4731e-005
R-0187	MinFlux:	0.013587	MaxFlux:	0.013587
R-0188	MinFlux:	0.024472	MaxFlux:	0.024472
R-0189	MinFlux:	0.038302	MaxFlux:	0.038302
R-0190	MinFlux:	0.038302	MaxFlux:	0.038302
R-0191	MinFlux:	0.00010872	MaxFlux:	0.00010872
R-0192	MinFlux:	0	MaxFlux:	0
R-0193	MinFlux:	0.012197	MaxFlux:	0.012197
R-0194	MinFlux:	0.038302	MaxFlux:	0.038302
R-0195	MinFlux:	0.038302	MaxFlux:	0.038302
R-0196	MinFlux:	0	MaxFlux:	0
R-0197	MinFlux:	0	MaxFlux:	0
R-0198	MinFlux:	0	MaxFlux:	0
R-0199	MinFlux:	0	MaxFlux:	0
R-0200	MinFlux:	0.00065297	MaxFlux:	0.00065297
R-0201	MinFlux:	0.010215	MaxFlux:	0.010215
R-0202	MinFlux:	0	MaxFlux:	0
R-0203	MinFlux:	0	MaxFlux:	0
R-0204	MinFlux:	0.010136	MaxFlux:	0.010136
R-0205	MinFlux:	0.00085061	MaxFlux:	0.00085061
R-0206	MinFlux:	0.010215	MaxFlux:	0.010215
R-0207	MinFlux:	0.010868	MaxFlux:	0.010868
R-0208	MinFlux:	0.010868	MaxFlux:	0.010868
R-0209	MinFlux:	0	MaxFlux:	0
R-0210	MinFlux:	0.046375	MaxFlux:	0.046375
R-0211	MinFlux:	0.046375	MaxFlux:	0.046375
R-0212	MinFlux:	0.046357	MaxFlux:	0.046357
R-0213	MinFlux:	0.046375	MaxFlux:	0.046375
R-0214	MinFlux:	0.046375	MaxFlux:	0.046375
R-0215	MinFlux:	0.046357	MaxFlux:	0.046357
R-0216	MinFlux:	0.046375	MaxFlux:	0.046375
R-0217	MinFlux:	-0.051549	MaxFlux:	-0.051549
R-0218	MinFlux:	0	MaxFlux:	0
R-0219	MinFlux:	0.012197	MaxFlux:	0.012197
R-0220	MinFlux:	0.012197	MaxFlux:	0.012197
R-0221	MinFlux:	0.012197	MaxFlux:	0.012197
R-0222	MinFlux:	0.012197	MaxFlux:	0.012197
R-0223	MinFlux:	0.012197	MaxFlux:	0.012197
R-0224	MinFlux:	0.012197	MaxFlux:	0.012197
R-0225	MinFlux:	0.012197	MaxFlux:	0.012197
R-0226	MinFlux:	0.012197	MaxFlux:	0.012197
R-0227	MinFlux:	0.012197	MaxFlux:	0.012197
R-0228	MinFlux:	0	MaxFlux:	0
R-0229	MinFlux:	0	MaxFlux:	0
R-0230	MinFlux:	0	MaxFlux:	0
R-0231	MinFlux:	0	MaxFlux:	0
R-0232	MinFlux:	0	MaxFlux:	0
R-0233	MinFlux:	0	MaxFlux:	0
R-0234	MinFlux:	0	MaxFlux:	0
R-0235	MinFlux:	0	MaxFlux:	0
R-0236	MinFlux:	0.0012649	MaxFlux:	0.0012649
R-0237	MinFlux:	0.00063245	MaxFlux:	0.00063245
R-0238	MinFlux:	0.00015811	MaxFlux:	0.00015811
R-0239	MinFlux:	0.00015811	MaxFlux:	0.00015811
R-0240	MinFlux:	0	MaxFlux:	0
R-0241	MinFlux:	0.15024	MaxFlux:	0.15024

R-0242	MinFlux:	0	MaxFlux:	0
R-0243	MinFlux:	0	MaxFlux:	0
R-0244	MinFlux:	0	MaxFlux:	0
R-0245	MinFlux:	0	MaxFlux:	0
R-0249	MinFlux:	0.09365	MaxFlux:	0.09365
R-0251	MinFlux:	0	MaxFlux:	0
R-0252	MinFlux:	0	MaxFlux:	0
R-0253	MinFlux:	0	MaxFlux:	0
R-0254	MinFlux:	0	MaxFlux:	0
R-0255	MinFlux:	0.037684	MaxFlux:	0.037684
R-0256	MinFlux:	0	MaxFlux:	0
R-0257	MinFlux:	0.10386	MaxFlux:	0.10386
R-0258	MinFlux:	0.055378	MaxFlux:	0.055378
R-0259	MinFlux:	0.055817	MaxFlux:	0.055817
R-0260	MinFlux:	0.09365	MaxFlux:	0.09365
R-0261	MinFlux:	0.15024	MaxFlux:	0.15024
R-0262	MinFlux:	0.21105	MaxFlux:	0.21105
R-0263	MinFlux:	0.31949	MaxFlux:	0.31949
R-0264	MinFlux:	0.31949	MaxFlux:	0.31949
R-0265	MinFlux:	0.31949	MaxFlux:	0.31949
R-0266	MinFlux:	3.9528e-005	MaxFlux:	3.9528e-005
R-0267	MinFlux:	0	MaxFlux:	0
R-0268	MinFlux:	0.014494	MaxFlux:	0.014494
R-0270	MinFlux:	0	MaxFlux:	0
R-0272	MinFlux:	0.00059721	MaxFlux:	0.00059721
R-0273	MinFlux:	0.00060194	MaxFlux:	0.00060194
R-0274	MinFlux:	0.00057638	MaxFlux:	0.00057638
R-0275	MinFlux:	0.0093739	MaxFlux:	0.0093739
R-0276	MinFlux:	0	MaxFlux:	0
R-0277	MinFlux:	0	MaxFlux:	0
R-0278	MinFlux:	0	MaxFlux:	0
R-0279	MinFlux:	0	MaxFlux:	0
R-0280	MinFlux:	0.0004061	MaxFlux:	0.0004061
R-0281	MinFlux:	0.00040932	MaxFlux:	0.00040932
R-0282	MinFlux:	0.00039194	MaxFlux:	0.00039194
R-0283	MinFlux:	0.0063742	MaxFlux:	0.0063742
R-0284	MinFlux:	0.00041805	MaxFlux:	0.00041805
R-0285	MinFlux:	0.00042136	MaxFlux:	0.00042136
R-0286	MinFlux:	0.00040346	MaxFlux:	0.00040346
R-0287	MinFlux:	0.0065617	MaxFlux:	0.0065617
R-0289	MinFlux:	0.00017916	MaxFlux:	0.00017916
R-0290	MinFlux:	0.00018058	MaxFlux:	0.00018058
R-0291	MinFlux:	0.00017292	MaxFlux:	0.00017292
R-0292	MinFlux:	0.0028122	MaxFlux:	0.0028122
R-0294	MinFlux:	0	MaxFlux:	0
R-0295	MinFlux:	0	MaxFlux:	0
R-0298	MinFlux:	0	MaxFlux:	0
R-0299	MinFlux:	0	MaxFlux:	0
R-0300	MinFlux:	0	MaxFlux:	0
R-0301	MinFlux:	0	MaxFlux:	0
R-0302	MinFlux:	0	MaxFlux:	0
R-0303	MinFlux:	0	MaxFlux:	0
R-0304	MinFlux:	0	MaxFlux:	0
R-0305	MinFlux:	0	MaxFlux:	0
R-0308	MinFlux:	0	MaxFlux:	0
R-0309	MinFlux:	0	MaxFlux:	0
R-0310	MinFlux:	0	MaxFlux:	0
R-0311	MinFlux:	0	MaxFlux:	0
R-0312	MinFlux:	0	MaxFlux:	0
R-0313	MinFlux:	0	MaxFlux:	0
R-0314	MinFlux:	0	MaxFlux:	0
R-0315	MinFlux:	0	MaxFlux:	0
R-0317	MinFlux:	0	MaxFlux:	0
R-0318	MinFlux:	0	MaxFlux:	0
R-0319	MinFlux:	0	MaxFlux:	0
R-0320	MinFlux:	0	MaxFlux:	0
R-0322	MinFlux:	0	MaxFlux:	0
R-0323	MinFlux:	0	MaxFlux:	0
R-0324	MinFlux:	0	MaxFlux:	0
R-0325	MinFlux:	0	MaxFlux:	0
R-0327	MinFlux:	0	MaxFlux:	0
R-0328	MinFlux:	0	MaxFlux:	0
R-0329	MinFlux:	0	MaxFlux:	0

R-0330	MinFlux:	0	MaxFlux:	0
R-0331	MinFlux:	0	MaxFlux:	0
R-0332	MinFlux:	0	MaxFlux:	0
R-0334	MinFlux:	0.00059721	MaxFlux:	0.00059721
R-0335	MinFlux:	0.00060194	MaxFlux:	0.00060194
R-0336	MinFlux:	0.00057638	MaxFlux:	0.00057638
R-0337	MinFlux:	0.0093739	MaxFlux:	0.0093739
R-0338	MinFlux:	0.17651	MaxFlux:	0.17651
R-0339	MinFlux:	0.022389	MaxFlux:	0.022389
R-0340	MinFlux:	0.022389	MaxFlux:	0.022389
R-0341	MinFlux:	0.022389	MaxFlux:	0.022389
R-0342	MinFlux:	0.022389	MaxFlux:	0.022389
R-0343	MinFlux:	0.022389	MaxFlux:	0.022389
R-0344	MinFlux:	0.0024372	MaxFlux:	0.0024372
R-0345	MinFlux:	0.0024192	MaxFlux:	0.0024192
R-0346	MinFlux:	0.0023472	MaxFlux:	0.0023472
R-0347	MinFlux:	0.0011528	MaxFlux:	0.0011528
R-0348	MinFlux:	0.019952	MaxFlux:	0.019952
R-0349	MinFlux:	0.019952	MaxFlux:	0.019952
R-0350	MinFlux:	0.019952	MaxFlux:	0.019952
R-0351	MinFlux:	0.018748	MaxFlux:	0.018748
R-0352	MinFlux:	0.022389	MaxFlux:	0.022389
R-0353	MinFlux:	0.022389	MaxFlux:	0.022389
R-0354	MinFlux:	0.022389	MaxFlux:	0.022389
R-0355	MinFlux:	0.022389	MaxFlux:	0.022389
R-0356	MinFlux:	0.0024372	MaxFlux:	0.0024372
R-0358	MinFlux:	0.0024192	MaxFlux:	0.0024192
R-0359	MinFlux:	0.0023472	MaxFlux:	0.0023472
R-0360	MinFlux:	0.0011528	MaxFlux:	0.0011528
R-0361	MinFlux:	0.019952	MaxFlux:	0.019952
R-0362	MinFlux:	0.019952	MaxFlux:	0.019952
R-0363	MinFlux:	0.019952	MaxFlux:	0.019952
R-0364	MinFlux:	0.018748	MaxFlux:	0.018748
R-0365	MinFlux:	0.022389	MaxFlux:	0.022389
R-0366	MinFlux:	0.019952	MaxFlux:	0.019952
R-0367	MinFlux:	0.022389	MaxFlux:	0.022389
R-0368	MinFlux:	0.022389	MaxFlux:	0.022389
R-0369	MinFlux:	0.022389	MaxFlux:	0.022389
R-0370	MinFlux:	0.0024372	MaxFlux:	0.0024372
R-0371	MinFlux:	0.0023472	MaxFlux:	0.0023472
R-0372	MinFlux:	0.0023472	MaxFlux:	0.0023472
R-0373	MinFlux:	0.0011528	MaxFlux:	0.0011528
R-0374	MinFlux:	0.019952	MaxFlux:	0.019952
R-0375	MinFlux:	0.019952	MaxFlux:	0.019952
R-0376	MinFlux:	0.019952	MaxFlux:	0.019952
R-0377	MinFlux:	0.018748	MaxFlux:	0.018748
R-0378	MinFlux:	0.022389	MaxFlux:	0.022389
R-0379	MinFlux:	0.022389	MaxFlux:	0.022389
R-0380	MinFlux:	0.022389	MaxFlux:	0.022389
R-0381	MinFlux:	0.0024372	MaxFlux:	0.0024372
R-0382	MinFlux:	0.0024372	MaxFlux:	0.0024372
R-0383	MinFlux:	0.0023472	MaxFlux:	0.0023472
R-0384	MinFlux:	0.0023472	MaxFlux:	0.0023472
R-0385	MinFlux:	0.0011528	MaxFlux:	0.0011528
R-0386	MinFlux:	0.019952	MaxFlux:	0.019952
R-0387	MinFlux:	0.019952	MaxFlux:	0.019952
R-0388	MinFlux:	0.019952	MaxFlux:	0.019952
R-0389	MinFlux:	0.018748	MaxFlux:	0.018748
R-0390	MinFlux:	0	MaxFlux:	0
R-0391	MinFlux:	0.0060459	MaxFlux:	0.0060459
R-0392	MinFlux:	0.19984	MaxFlux:	0.19984
R-0393	MinFlux:	-0.38518	MaxFlux:	-0.38441
R-0394	MinFlux:	0.021836	MaxFlux:	0.021836
R-0395	MinFlux:	0	MaxFlux:	0
R-0396	MinFlux:	0.04955	MaxFlux:	0.04955
R-0397	MinFlux:	1.7685	MaxFlux:	1.7705
R-0398	MinFlux:	3.9528e-005	MaxFlux:	3.9528e-005
R-0399	MinFlux:	3.9528e-005	MaxFlux:	3.9528e-005
R-0400	MinFlux:	0	MaxFlux:	0.001236
R-0401	MinFlux:	0.037684	MaxFlux:	0.037684
R-0402	MinFlux:	0	MaxFlux:	0
R-0403	MinFlux:	1.4559	MaxFlux:	1.4566
R-0404	MinFlux:	0.074978	MaxFlux:	0.074978

R-0405	MinFlux:	0.0030081	MaxFlux:	0.0030081
R-0406	MinFlux:	0.0030081	MaxFlux:	0.0030081
R-0407	MinFlux:	0	MaxFlux:	0
R-0408	MinFlux:	0.00017567	MaxFlux:	0.00017567
R-0409	MinFlux:	0	MaxFlux:	0
R-0410	MinFlux:	0.056218	MaxFlux:	0.056218
R-0411	MinFlux:	0	MaxFlux:	0
R-0412	MinFlux:	0	MaxFlux:	0
R-0413	MinFlux:	0	MaxFlux:	0
R-0414	MinFlux:	0	MaxFlux:	0
R-0415	MinFlux:	0.043381	MaxFlux:	0.043381
R-0416	MinFlux:	0.055951	MaxFlux:	0.055951
R-0417	MinFlux:	0.043381	MaxFlux:	0.043381
R-0418	MinFlux:	1.8e-005	MaxFlux:	1.8e-005
R-0419	MinFlux:	0	MaxFlux:	0
R-0420	MinFlux:	1.8e-005	MaxFlux:	1.8e-005
R-0421	MinFlux:	0	MaxFlux:	0
R-0422	MinFlux:	0.0060459	MaxFlux:	0.0060459
R-0423	MinFlux:	0.0060459	MaxFlux:	0.0060459
R-0424	MinFlux:	-0.0060459	MaxFlux:	-0.0060459
R-0425	MinFlux:	0	MaxFlux:	0
R-0426	MinFlux:	0.001236	MaxFlux:	0.001236
R-0427	MinFlux:	0.001236	MaxFlux:	0.001236
R-0428	MinFlux:	0.001236	MaxFlux:	0.001236
R-0429	MinFlux:	0	MaxFlux:	0
R-0430	MinFlux:	-0.19978	MaxFlux:	-0.19978
R-0431	MinFlux:	0.028005	MaxFlux:	0.028777
R-0432	MinFlux:	0.031597	MaxFlux:	0.031597
R-0433	MinFlux:	0.043381	MaxFlux:	0.043381
R-0434	MinFlux:	0.044602	MaxFlux:	0.044602
R-0435	MinFlux:	0	MaxFlux:	0
R-0436	MinFlux:	0.19862	MaxFlux:	0.19862
R-0437	MinFlux:	0.043381	MaxFlux:	0.043381
R-0438	MinFlux:	0.028005	MaxFlux:	0.028777
R-0439	MinFlux:	0.037644	MaxFlux:	0.037644
R-0440	MinFlux:	0	MaxFlux:	0
R-0441	MinFlux:	0	MaxFlux:	0
R-0442	MinFlux:	0	MaxFlux:	0.00077155
R-0443	MinFlux:	27.1232	MaxFlux:	32.0234
R-0444	MinFlux:	5.0774	MaxFlux:	19.5093
R-0445	MinFlux:	0	MaxFlux:	14.218
R-0446	MinFlux:	0	MaxFlux:	14.218
R-0447	MinFlux:	7.7747	MaxFlux:	11.5219
R-0448	MinFlux:	0	MaxFlux:	3.2766
R-0449	MinFlux:	0.037684	MaxFlux:	0.037684
R-0450	MinFlux:	0.037684	MaxFlux:	0.037684
R-0451	MinFlux:	0.037684	MaxFlux:	0.037684
R-0452	MinFlux:	0.037684	MaxFlux:	0.037684
R-0453	MinFlux:	0.037644	MaxFlux:	0.037644
R-0454	MinFlux:	0.037644	MaxFlux:	0.037644
R-0455	MinFlux:	0.049842	MaxFlux:	0.049842
R-0456	MinFlux:	0.049842	MaxFlux:	0.049842
R-0457	MinFlux:	0.021836	MaxFlux:	0.021836
R-0458	MinFlux:	0.021876	MaxFlux:	0.021876
R-0459	MinFlux:	0	MaxFlux:	0
R-0460	MinFlux:	0.10644	MaxFlux:	0.10644
R-0461	MinFlux:	0.0063129	MaxFlux:	0.0063129
R-0462	MinFlux:	0	MaxFlux:	0
R-0463	MinFlux:	0.0087367	MaxFlux:	0.0087367
R-0464	MinFlux:	0.11259	MaxFlux:	0.11259
R-0465	MinFlux:	0.0027518	MaxFlux:	0.0027518
R-0466	MinFlux:	0.0059848	MaxFlux:	0.0059848
R-0467	MinFlux:	0.043896	MaxFlux:	0.044668
R-0469	MinFlux:	0.024396	MaxFlux:	0.024396
R-0470	MinFlux:	0	MaxFlux:	0
R-0471	MinFlux:	0.0087367	MaxFlux:	0.0087367
R-0472	MinFlux:	0.0027518	MaxFlux:	0.0027518
R-0474	MinFlux:	0.0059848	MaxFlux:	0.0059848
R-0475	MinFlux:	0.030815	MaxFlux:	0.030815
R-0476	MinFlux:	1.3097	MaxFlux:	5.9676
R-0477	MinFlux:	0.031597	MaxFlux:	0.031597
R-0478	MinFlux:	0.031597	MaxFlux:	0.031597
R-0479	MinFlux:	0.031597	MaxFlux:	0.031597

R-0480	MinFlux:	0.031597	MaxFlux:	0.031597
R-0481	MinFlux:	0.01505	MaxFlux:	0.01505
R-0482	MinFlux:	0.031633	MaxFlux:	0.031633
R-0483	MinFlux:	0.0027518	MaxFlux:	0.0027518
R-0484	MinFlux:	0.0027518	MaxFlux:	0.0027518
R-0485	MinFlux:	0.0027666	MaxFlux:	0.0027666
R-0486	MinFlux:	0.0027518	MaxFlux:	0.0027518
R-0487	MinFlux:	0	MaxFlux:	0
R-0489	MinFlux:	0	MaxFlux:	0
R-0490	MinFlux:	0	MaxFlux:	0
R-0491	MinFlux:	0	MaxFlux:	0
R-0492	MinFlux:	0	MaxFlux:	0
R-0493	MinFlux:	0	MaxFlux:	0
R-0494	MinFlux:	0	MaxFlux:	0
R-0495	MinFlux:	0	MaxFlux:	0
R-0496	MinFlux:	1.4731e-005	MaxFlux:	1.4731e-005
R-0497	MinFlux:	1.4731e-005	MaxFlux:	1.4731e-005
R-0498	MinFlux:	1.4731e-005	MaxFlux:	1.4731e-005
R-0499	MinFlux:	1.4731e-005	MaxFlux:	1.4731e-005
R-0500	MinFlux:	1.4731e-005	MaxFlux:	1.4731e-005
R-0501	MinFlux:	1.4731e-005	MaxFlux:	1.4731e-005
R-0502	MinFlux:	0	MaxFlux:	0
R-0503	MinFlux:	1.4731e-005	MaxFlux:	1.4731e-005
R-0504	MinFlux:	0	MaxFlux:	0
R-0505	MinFlux:	0	MaxFlux:	0
R-0506	MinFlux:	5.94e-005	MaxFlux:	5.94e-005
R-0507	MinFlux:	0.0011107	MaxFlux:	0.0011107
R-0508	MinFlux:	0.0011107	MaxFlux:	0.0011107
R-0509	MinFlux:	0.0011107	MaxFlux:	0.0011107
R-0510	MinFlux:	0.0011107	MaxFlux:	0.0011107
R-0511	MinFlux:	0.0011107	MaxFlux:	0.0011107
R-0512	MinFlux:	0.00022902	MaxFlux:	0.00022902
R-0513	MinFlux:	0.00088171	MaxFlux:	0.00088171
R-0514	MinFlux:	0.00022902	MaxFlux:	0.00022902
R-0515	MinFlux:	0.00022902	MaxFlux:	0.00022902
R-0516	MinFlux:	0.00017863	MaxFlux:	0.00017863
R-0517	MinFlux:	6.9552e-005	MaxFlux:	6.9552e-005
R-0518	MinFlux:	6.9552e-005	MaxFlux:	6.9552e-005
R-0519	MinFlux:	6.9552e-005	MaxFlux:	6.9552e-005
R-0520	MinFlux:	6.9552e-005	MaxFlux:	6.9552e-005
R-0521	MinFlux:	6.9552e-005	MaxFlux:	6.9552e-005
R-0522	MinFlux:	6.9552e-005	MaxFlux:	6.9552e-005
R-0523	MinFlux:	0.013627	MaxFlux:	0.013627
R-0524	MinFlux:	0.024472	MaxFlux:	0.024472
R-0525	MinFlux:	0.013627	MaxFlux:	0.013627
R-0526	MinFlux:	0.19982	MaxFlux:	0.19982
R-0527	MinFlux:	0.012197	MaxFlux:	0.012197
R-0529	MinFlux:	0.00059721	MaxFlux:	0.00059721
R-0530	MinFlux:	0.00060194	MaxFlux:	0.00060194
R-0531	MinFlux:	0.00057638	MaxFlux:	0.00057638
R-0532	MinFlux:	0.0093739	MaxFlux:	0.0093739
R-0534	MinFlux:	0.00059721	MaxFlux:	0.00059721
R-0535	MinFlux:	0.00060194	MaxFlux:	0.00060194
R-0536	MinFlux:	0.00057638	MaxFlux:	0.00057638
R-0537	MinFlux:	0.0093739	MaxFlux:	0.0093739
R-0538	MinFlux:	0.0027518	MaxFlux:	0.0027518
R-0539	MinFlux:	0	MaxFlux:	0
R-0540	MinFlux:	0.049931	MaxFlux:	0.049931
R-0541	MinFlux:	0.049931	MaxFlux:	0.049931
R-0542	MinFlux:	0.044947	MaxFlux:	0.044947
R-0543	MinFlux:	0.044947	MaxFlux:	0.044947
R-0544	MinFlux:	0.056218	MaxFlux:	0.056218
R-0545	MinFlux:	0.056218	MaxFlux:	0.056218
R-0546	MinFlux:	0.056218	MaxFlux:	0.056218
R-0547	MinFlux:	0	MaxFlux:	0
R-0548	MinFlux:	0.051549	MaxFlux:	0.051549
R-0549	MinFlux:	0.051549	MaxFlux:	0.051549
R-0550	MinFlux:	0	MaxFlux:	0
R-0551	MinFlux:	0.051549	MaxFlux:	0.051549
R-0552	MinFlux:	0	MaxFlux:	0
R-0553	MinFlux:	0.00010872	MaxFlux:	0.00010872
R-0554	MinFlux:	0	MaxFlux:	0
R-0555	MinFlux:	0	MaxFlux:	0

R-0556	MinFlux:	0	MaxFlux:	0
R-0557	MinFlux:	0	MaxFlux:	0
R-0558	MinFlux:	0	MaxFlux:	0
R-0559	MinFlux:	0	MaxFlux:	0
R-0560	MinFlux:	0.033205	MaxFlux:	0.033205
R-0561	MinFlux:	0	MaxFlux:	0
R-0562	MinFlux:	0	MaxFlux:	0
R-0563	MinFlux:	3.9528e-005	MaxFlux:	3.9528e-005
R-0565	MinFlux:	3.6e-005	MaxFlux:	3.6e-005
R-0566	MinFlux:	3.6e-005	MaxFlux:	3.6e-005
R-0567	MinFlux:	3.6e-005	MaxFlux:	3.6e-005
R-0568	MinFlux:	-3.6e-005	MaxFlux:	-3.6e-005
R-0569	MinFlux:	0	MaxFlux:	0
R-0570	MinFlux:	0	MaxFlux:	0.00077155
R-0571	MinFlux:	0	MaxFlux:	0
R-0572	MinFlux:	0	MaxFlux:	0
R-0573	MinFlux:	3.9528e-005	MaxFlux:	3.9528e-005
R-0574	MinFlux:	3.9528e-005	MaxFlux:	3.9528e-005
R-0576	MinFlux:	0	MaxFlux:	0
R-0577	MinFlux:	0	MaxFlux:	0
R-0578	MinFlux:	0	MaxFlux:	0
R-0579	MinFlux:	0	MaxFlux:	0
R-0580	MinFlux:	0	MaxFlux:	0
R-0581	MinFlux:	7.9056e-005	MaxFlux:	7.9056e-005
R-0582	MinFlux:	0	MaxFlux:	0
R-0583	MinFlux:	0	MaxFlux:	0
R-0584	MinFlux:	3.9528e-005	MaxFlux:	3.9528e-005
R-0585	MinFlux:	3.9528e-005	MaxFlux:	3.9528e-005
R-0586	MinFlux:	3.9528e-005	MaxFlux:	3.9528e-005
R-0587	MinFlux:	0	MaxFlux:	0
R-0588	MinFlux:	3.9528e-005	MaxFlux:	3.9528e-005
R-0589	MinFlux:	3.9528e-005	MaxFlux:	3.9528e-005
R-0590	MinFlux:	3.9528e-005	MaxFlux:	3.9528e-005
R-0591	MinFlux:	3.9528e-005	MaxFlux:	3.9528e-005
R-0593	MinFlux:	0	MaxFlux:	0
R-0594	MinFlux:	0	MaxFlux:	0
R-0595	MinFlux:	0	MaxFlux:	0
R-0596	MinFlux:	0.0081678	MaxFlux:	0.0081678
R-0597	MinFlux:	3.9528e-005	MaxFlux:	3.9528e-005
R-0598	MinFlux:	3.9528e-005	MaxFlux:	3.9528e-005
R-0599	MinFlux:	3.9528e-005	MaxFlux:	3.9528e-005
R-0600	MinFlux:	0	MaxFlux:	0
R-0601	MinFlux:	3.9528e-005	MaxFlux:	3.9528e-005
R-0602	MinFlux:	0	MaxFlux:	0
R-0603	MinFlux:	0	MaxFlux:	0
R-0604	MinFlux:	0	MaxFlux:	0
R-0605	MinFlux:	0	MaxFlux:	0
R-0606	MinFlux:	0	MaxFlux:	3.4934
R-0607	MinFlux:	3.9528e-005	MaxFlux:	3.9528e-005
R-0608	MinFlux:	-5.9292e-005	MaxFlux:	-5.9292e-005
R-0609	MinFlux:	3.9528e-005	MaxFlux:	3.9528e-005
R-0610	MinFlux:	3.9528e-005	MaxFlux:	3.9528e-005
R-0611	MinFlux:	3.9528e-005	MaxFlux:	3.9528e-005
R-0612	MinFlux:	3.9528e-005	MaxFlux:	3.9528e-005
R-0613	MinFlux:	3.9528e-005	MaxFlux:	3.9528e-005
R-0614	MinFlux:	0	MaxFlux:	0
R-0615	MinFlux:	0	MaxFlux:	0
R-0616	MinFlux:	7.9056e-005	MaxFlux:	7.9056e-005
R-0617	MinFlux:	0.0027534	MaxFlux:	0.0027534
R-0618	MinFlux:	7.9056e-005	MaxFlux:	7.9056e-005
R-0619	MinFlux:	3.9528e-005	MaxFlux:	3.9528e-005
R-0620	MinFlux:	4.752e-006	MaxFlux:	0.32346
R-0621	MinFlux:	0	MaxFlux:	0
R-0622	MinFlux:	0	MaxFlux:	0
R-0623	MinFlux:	0	MaxFlux:	0
R-0624	MinFlux:	3.9528e-005	MaxFlux:	3.9528e-005
R-0625	MinFlux:	0	MaxFlux:	0
R-0626	MinFlux:	0	MaxFlux:	0
R-0627	MinFlux:	0.0027534	MaxFlux:	0.0027534
R-0628	MinFlux:	0	MaxFlux:	0
R-0629	MinFlux:	0	MaxFlux:	0
R-0630	MinFlux:	0	MaxFlux:	0
R-0631	MinFlux:	0	MaxFlux:	0

R-0632	MinFlux:	0	MaxFlux:	0
R-0633	MinFlux:	0	MaxFlux:	0
R-0634	MinFlux:	0	MaxFlux:	0
R-0635	MinFlux:	0	MaxFlux:	0
R-0636	MinFlux:	0	MaxFlux:	4.62
R-0637	MinFlux:	3.6e-005	MaxFlux:	3.6e-005
R-0638	MinFlux:	1.0865e-005	MaxFlux:	1.0865e-005
R-0639	MinFlux:	1.0865e-005	MaxFlux:	1.0865e-005
R-0640	MinFlux:	1.0865e-005	MaxFlux:	1.0865e-005
R-0641	MinFlux:	1.0865e-005	MaxFlux:	1.0865e-005
R-0642	MinFlux:	1.0865e-005	MaxFlux:	1.0865e-005
R-0643	MinFlux:	1.0865e-005	MaxFlux:	1.0865e-005
R-0644	MinFlux:	1.0865e-005	MaxFlux:	1.0865e-005
R-0645	MinFlux:	1.0865e-005	MaxFlux:	1.0865e-005
R-0646	MinFlux:	0	MaxFlux:	0
R-0647	MinFlux:	0	MaxFlux:	0
R-0648	MinFlux:	0	MaxFlux:	0
R-0649	MinFlux:	0	MaxFlux:	0
R-0650	MinFlux:	0	MaxFlux:	0
R-0651	MinFlux:	0	MaxFlux:	0
R-0652	MinFlux:	0	MaxFlux:	0
R-0653	MinFlux:	0	MaxFlux:	0
R-0654	MinFlux:	0	MaxFlux:	0
R-0655	MinFlux:	0	MaxFlux:	0
R-0656	MinFlux:	0	MaxFlux:	0
R-0657	MinFlux:	0	MaxFlux:	0
R-0658	MinFlux:	0	MaxFlux:	0
R-0659	MinFlux:	0	MaxFlux:	0
R-0660	MinFlux:	0	MaxFlux:	0
R-0661	MinFlux:	0	MaxFlux:	0
R-0662	MinFlux:	0.0027534	MaxFlux:	0.0027534
R-0663	MinFlux:	0.013341	MaxFlux:	0.013341
R-0664	MinFlux:	0.013341	MaxFlux:	0.013341
R-0665	MinFlux:	0.013341	MaxFlux:	0.013341
R-0666	MinFlux:	3.9528e-005	MaxFlux:	3.9528e-005
R-0667	MinFlux:	0	MaxFlux:	0
R-0669	MinFlux:	0	MaxFlux:	0
R-0670	MinFlux:	0.0011107	MaxFlux:	0.0011107
R-0671	MinFlux:	3.9528e-005	MaxFlux:	3.9528e-005
R-0672	MinFlux:	1.8e-005	MaxFlux:	1.8e-005
R-0673	MinFlux:	1.8e-005	MaxFlux:	1.8e-005
R-0674	MinFlux:	0.0027534	MaxFlux:	0.0027534
R-0675	MinFlux:	0	MaxFlux:	0
R-0676	MinFlux:	0	MaxFlux:	0
R-0677	MinFlux:	0	MaxFlux:	3.4934
R-0679	MinFlux:	0	MaxFlux:	0
R-0680	MinFlux:	0	MaxFlux:	0
R-0681	MinFlux:	0	MaxFlux:	0
R-0682	MinFlux:	0	MaxFlux:	0
R-0683	MinFlux:	0	MaxFlux:	0
R-0684	MinFlux:	0	MaxFlux:	0
R-0685	MinFlux:	6.9552e-005	MaxFlux:	6.9552e-005
R-0686	MinFlux:	6.9552e-005	MaxFlux:	6.9552e-005
R-0687	MinFlux:	0	MaxFlux:	0
R-0688	MinFlux:	0	MaxFlux:	0
R-0689	MinFlux:	0	MaxFlux:	0
R-0690	MinFlux:	0	MaxFlux:	0
R-0691	MinFlux:	6.9552e-005	MaxFlux:	6.9552e-005
R-0692	MinFlux:	6.9552e-005	MaxFlux:	6.9552e-005
R-0693	MinFlux:	0	MaxFlux:	0
R-0694	MinFlux:	0	MaxFlux:	0
R-0695	MinFlux:	0	MaxFlux:	0
R-0696	MinFlux:	0	MaxFlux:	0
R-0697	MinFlux:	0	MaxFlux:	0
R-0698	MinFlux:	0	MaxFlux:	0
R-0699	MinFlux:	0	MaxFlux:	0
R-0700	MinFlux:	0.25149	MaxFlux:	0.57495
R-0701	MinFlux:	3.9528e-005	MaxFlux:	3.9528e-005
R-0702	MinFlux:	0.001236	MaxFlux:	0.001236
R-0703	MinFlux:	0	MaxFlux:	0
R-0704	MinFlux:	0	MaxFlux:	0
R-0705	MinFlux:	1.8e-005	MaxFlux:	1.8e-005
R-0706	MinFlux:	1.8e-005	MaxFlux:	1.8e-005

R-0707	MinFlux:	1.8e-005	MaxFlux:	1.8e-005
R-0708	MinFlux:	3.6e-005	MaxFlux:	3.6e-005
R-0709	MinFlux:	0	MaxFlux:	0
R-0710	MinFlux:	0	MaxFlux:	0
R-0711	MinFlux:	0	MaxFlux:	0
R-0712	MinFlux:	0	MaxFlux:	0
R-0713	MinFlux:	0	MaxFlux:	0
R-0714	MinFlux:	1.8e-005	MaxFlux:	1.8e-005
R-0715	MinFlux:	1.8e-005	MaxFlux:	1.8e-005
R-0716	MinFlux:	1.8e-005	MaxFlux:	1.8e-005
R-0717	MinFlux:	9.882e-005	MaxFlux:	9.882e-005
R-0718	MinFlux:	0	MaxFlux:	0
R-0719	MinFlux:	0	MaxFlux:	0
R-0720	MinFlux:	0	MaxFlux:	0
R-0721	MinFlux:	0	MaxFlux:	0
R-0722	MinFlux:	0	MaxFlux:	0
R-0724	MinFlux:	0	MaxFlux:	6.16
R-0725	MinFlux:	3.9528e-005	MaxFlux:	3.9528e-005
R-0726	MinFlux:	3.9528e-005	MaxFlux:	3.9528e-005
R-0727	MinFlux:	3.9528e-005	MaxFlux:	3.9528e-005
R-0728	MinFlux:	3.9528e-005	MaxFlux:	3.9528e-005
R-0729	MinFlux:	3.9528e-005	MaxFlux:	3.9528e-005
R-0730	MinFlux:	0.00019764	MaxFlux:	0.00019764
R-0731	MinFlux:	9.882e-005	MaxFlux:	9.882e-005
R-0732	MinFlux:	9.882e-005	MaxFlux:	9.882e-005
R-0733	MinFlux:	9.882e-005	MaxFlux:	9.882e-005
R-0736	MinFlux:	0.016555	MaxFlux:	0.016555
R-0737	MinFlux:	3.9528e-005	MaxFlux:	3.9528e-005
R-0738	MinFlux:	0	MaxFlux:	0
R-0739	MinFlux:	7.9056e-005	MaxFlux:	7.9056e-005
R-0740	MinFlux:	7.9056e-005	MaxFlux:	7.9056e-005
R-0741	MinFlux:	3.9528e-005	MaxFlux:	3.9528e-005
R-0742	MinFlux:	3.9528e-005	MaxFlux:	3.9528e-005
R-0743	MinFlux:	3.9528e-005	MaxFlux:	3.9528e-005
R-0744	MinFlux:	0	MaxFlux:	0
R-0745	MinFlux:	0	MaxFlux:	0
R-0746	MinFlux:	0	MaxFlux:	0
R-0747	MinFlux:	0	MaxFlux:	0
R-0748	MinFlux:	0	MaxFlux:	0
R-0749	MinFlux:	0	MaxFlux:	0
R-0750	MinFlux:	0	MaxFlux:	0
R-0751	MinFlux:	0	MaxFlux:	0
R-0752	MinFlux:	0	MaxFlux:	0
R-0753	MinFlux:	0	MaxFlux:	0
R-0754	MinFlux:	0	MaxFlux:	0
R-0755	MinFlux:	0	MaxFlux:	0
R-0756	MinFlux:	3.9528e-005	MaxFlux:	3.9528e-005
R-0757	MinFlux:	0	MaxFlux:	0
R-0758	MinFlux:	0	MaxFlux:	0
R-0759	MinFlux:	0	MaxFlux:	0
R-0760	MinFlux:	0	MaxFlux:	0
R-0762	MinFlux:	0	MaxFlux:	0
R-0763	MinFlux:	3.9528e-005	MaxFlux:	3.9528e-005
R-0764	MinFlux:	0	MaxFlux:	0
R-0765	MinFlux:	0	MaxFlux:	0
R-0766	MinFlux:	0	MaxFlux:	0
R-0767	MinFlux:	0	MaxFlux:	0
R-0768	MinFlux:	0	MaxFlux:	0
R-0769	MinFlux:	0	MaxFlux:	0
R-0770	MinFlux:	0	MaxFlux:	0
R-0771	MinFlux:	0	MaxFlux:	0
R-0772	MinFlux:	0	MaxFlux:	0
R-0773	MinFlux:	0	MaxFlux:	0
R-0774	MinFlux:	0	MaxFlux:	0
R-0775	MinFlux:	0	MaxFlux:	0
R-0776	MinFlux:	0	MaxFlux:	0.00077155
R-0777	MinFlux:	0	MaxFlux:	0
R-0778	MinFlux:	1.8e-005	MaxFlux:	1.8e-005
R-0779	MinFlux:	0	MaxFlux:	0
R-0780	MinFlux:	0	MaxFlux:	0
R-0781	MinFlux:	0	MaxFlux:	0
R-0782	MinFlux:	0	MaxFlux:	0
R-0783	MinFlux:	0.00019764	MaxFlux:	0.00019764

R-0784	MinFlux:	9.882e-005	MaxFlux:	9.882e-005
R-0785	MinFlux:	0	MaxFlux:	0
R-0786	MinFlux:	0	MaxFlux:	0
R-0787	MinFlux:	0	MaxFlux:	0.001236
R-0788	MinFlux:	0	MaxFlux:	0
R-0789	MinFlux:	3.9528e-005	MaxFlux:	3.9528e-005
R-0790	MinFlux:	3.9528e-005	MaxFlux:	3.9528e-005
R-0791	MinFlux:	3.9528e-005	MaxFlux:	3.9528e-005
R-0792	MinFlux:	3.9528e-005	MaxFlux:	3.9528e-005
R-0793	MinFlux:	3.9528e-005	MaxFlux:	3.9528e-005
R-0794	MinFlux:	0	MaxFlux:	0
R-0795	MinFlux:	0	MaxFlux:	0
R-0796	MinFlux:	0	MaxFlux:	0
R-0797	MinFlux:	0	MaxFlux:	0
R-0798	MinFlux:	0	MaxFlux:	0
R-0799	MinFlux:	0	MaxFlux:	0
R-0800	MinFlux:	0	MaxFlux:	0
R-0801	MinFlux:	0	MaxFlux:	0
R-0802	MinFlux:	0	MaxFlux:	0
R-0803	MinFlux:	3.9528e-005	MaxFlux:	3.9528e-005
R-0804	MinFlux:	0	MaxFlux:	0
R-0805	MinFlux:	0	MaxFlux:	0
R-0806	MinFlux:	0.0027534	MaxFlux:	0.0027534
R-0807	MinFlux:	0.0027534	MaxFlux:	0.0027534
R-0808	MinFlux:	0	MaxFlux:	0
R-0809	MinFlux:	3.9528e-005	MaxFlux:	3.9528e-005
R-0810	MinFlux:	3.9528e-005	MaxFlux:	3.9528e-005
R-0811	MinFlux:	0	MaxFlux:	0
R-0812	MinFlux:	0	MaxFlux:	0
R-0813	MinFlux:	0	MaxFlux:	0
R-0814	MinFlux:	0.0001391	MaxFlux:	0.0001391
R-0815	MinFlux:	0	MaxFlux:	0
R-0816	MinFlux:	0	MaxFlux:	0
R-0817	MinFlux:	0	MaxFlux:	0
R-0819	MinFlux:	0	MaxFlux:	0
R-0820	MinFlux:	0	MaxFlux:	0
R-0821	MinFlux:	0	MaxFlux:	0
R-0822	MinFlux:	0	MaxFlux:	0
R-0823	MinFlux:	0	MaxFlux:	0
R-0824	MinFlux:	0	MaxFlux:	0
R-0825	MinFlux:	0	MaxFlux:	0
R-0826	MinFlux:	0	MaxFlux:	0
R-0827	MinFlux:	0	MaxFlux:	0
R-0828	MinFlux:	0	MaxFlux:	0
R-0829	MinFlux:	0	MaxFlux:	0
R-0830	MinFlux:	0	MaxFlux:	0
R-0831	MinFlux:	0	MaxFlux:	0
R-0832	MinFlux:	0	MaxFlux:	0
R-0833	MinFlux:	0	MaxFlux:	0
R-0834	MinFlux:	3.9528e-005	MaxFlux:	3.9528e-005
R-0835	MinFlux:	0	MaxFlux:	0
R-0837	MinFlux:	0	MaxFlux:	0
R-0838	MinFlux:	-3.9528e-005	MaxFlux:	-3.9528e-005
R-0839	MinFlux:	0.0027534	MaxFlux:	0.0027534
R-0840	MinFlux:	1.8e-005	MaxFlux:	1.8e-005
R-0841	MinFlux:	1.8e-005	MaxFlux:	1.8e-005
R-0842	MinFlux:	1.8e-005	MaxFlux:	1.8e-005
R-0843	MinFlux:	0	MaxFlux:	0
R-0844	MinFlux:	1.8e-005	MaxFlux:	1.8e-005
R-0845	MinFlux:	3.6e-005	MaxFlux:	3.6e-005
R-0846	MinFlux:	1.8e-005	MaxFlux:	1.8e-005
R-0847	MinFlux:	0	MaxFlux:	0
R-0848	MinFlux:	0	MaxFlux:	0
R-0849	MinFlux:	0	MaxFlux:	0
R-0850	MinFlux:	3.9528e-005	MaxFlux:	3.9528e-005
R-0851	MinFlux:	3.9528e-005	MaxFlux:	3.9528e-005
R-0852	MinFlux:	3.9528e-005	MaxFlux:	3.9528e-005
R-0853	MinFlux:	3.9528e-005	MaxFlux:	3.9528e-005
R-0854	MinFlux:	0	MaxFlux:	0
R-0855	MinFlux:	0	MaxFlux:	0
R-0856	MinFlux:	0	MaxFlux:	0
R-0857	MinFlux:	0	MaxFlux:	0
R-0858	MinFlux:	0	MaxFlux:	0

R-0859	MinFlux:	0	MaxFlux:	0
R-0860	MinFlux:	0	MaxFlux:	0
R-0861	MinFlux:	0	MaxFlux:	0
R-0862	MinFlux:	0	MaxFlux:	0
R-0863	MinFlux:	0	MaxFlux:	0
R-0864	MinFlux:	0	MaxFlux:	0
R-0865	MinFlux:	0	MaxFlux:	0
R-0866	MinFlux:	0	MaxFlux:	0
R-0867	MinFlux:	0	MaxFlux:	0
R-0868	MinFlux:	0	MaxFlux:	0
R-0869	MinFlux:	0	MaxFlux:	0
R-0870	MinFlux:	0	MaxFlux:	0
R-0871	MinFlux:	0	MaxFlux:	0
R-0872	MinFlux:	0	MaxFlux:	0
R-0873	MinFlux:	6.9552e-005	MaxFlux:	6.9552e-005
R-0874	MinFlux:	6.9552e-005	MaxFlux:	6.9552e-005
R-0875	MinFlux:	0	MaxFlux:	0
R-0876	MinFlux:	0	MaxFlux:	0
R-0877	MinFlux:	0	MaxFlux:	0
R-0878	MinFlux:	3.9528e-005	MaxFlux:	3.9528e-005
R-0879	MinFlux:	0	MaxFlux:	0
R-0880	MinFlux:	0	MaxFlux:	0
R-0881	MinFlux:	0	MaxFlux:	0
R-0882	MinFlux:	0	MaxFlux:	0
R-0883	MinFlux:	0	MaxFlux:	0
R-0884	MinFlux:	0	MaxFlux:	0
R-0885	MinFlux:	0	MaxFlux:	0
R-0886	MinFlux:	0	MaxFlux:	0
R-0887	MinFlux:	0	MaxFlux:	0
R-0888	MinFlux:	0	MaxFlux:	0
R-0889	MinFlux:	0	MaxFlux:	0
R-0890	MinFlux:	0	MaxFlux:	0
R-0891	MinFlux:	0	MaxFlux:	0
R-0892	MinFlux:	0	MaxFlux:	0
R-0893	MinFlux:	0	MaxFlux:	0
R-0894	MinFlux:	0	MaxFlux:	0
R-0895	MinFlux:	0	MaxFlux:	0
R-0896	MinFlux:	0	MaxFlux:	0
R-0897	MinFlux:	0	MaxFlux:	0
R-0898	MinFlux:	0	MaxFlux:	0
R-0899	MinFlux:	0	MaxFlux:	0
R-0900	MinFlux:	0	MaxFlux:	0
R-0901	MinFlux:	0	MaxFlux:	0
R-0902	MinFlux:	0	MaxFlux:	0
R-0903	MinFlux:	0	MaxFlux:	0
R-0904	MinFlux:	0	MaxFlux:	0
R-0905	MinFlux:	0	MaxFlux:	0
R-0906	MinFlux:	0	MaxFlux:	0
R-0907	MinFlux:	0	MaxFlux:	0
R-0908	MinFlux:	0	MaxFlux:	0
R-0909	MinFlux:	0	MaxFlux:	0
R-0910	MinFlux:	0	MaxFlux:	0
R-0911	MinFlux:	0	MaxFlux:	0
R-0913	MinFlux:	0	MaxFlux:	0
R-0914	MinFlux:	0	MaxFlux:	0
R-0915	MinFlux:	0	MaxFlux:	0
R-0916	MinFlux:	0	MaxFlux:	0
R-0917	MinFlux:	0	MaxFlux:	0
R-0918	MinFlux:	0	MaxFlux:	0
R-0919	MinFlux:	0	MaxFlux:	0
R-0920	MinFlux:	0	MaxFlux:	0
R-0921	MinFlux:	0	MaxFlux:	0
R-0922	MinFlux:	0	MaxFlux:	0
R-0923	MinFlux:	0	MaxFlux:	0
R-0924	MinFlux:	0	MaxFlux:	0
R-0925	MinFlux:	0	MaxFlux:	0
R-0926	MinFlux:	0	MaxFlux:	0
R-0927	MinFlux:	0	MaxFlux:	0
R-0928	MinFlux:	3.9528e-005	MaxFlux:	3.9528e-005
R-0929	MinFlux:	0	MaxFlux:	0
R-0930	MinFlux:	0.0027534	MaxFlux:	0.0027534
R-0933	MinFlux:	0.00017916	MaxFlux:	0.00017916
R-0934	MinFlux:	0.00018058	MaxFlux:	0.00018058

R-0935	MinFlux:	0.00017292	MaxFlux:	0.00017292
R-0936	MinFlux:	0.0028122	MaxFlux:	0.0028122
R-0937	MinFlux:	7.9056e-005	MaxFlux:	7.9056e-005
R-0938	MinFlux:	3.9528e-005	MaxFlux:	3.9528e-005
R-0939	MinFlux:	9.882e-005	MaxFlux:	9.882e-005
R-0940	MinFlux:	3.9528e-005	MaxFlux:	3.9528e-005
R-0941	MinFlux:	1.8e-005	MaxFlux:	1.8e-005
R-0942	MinFlux:	3.9528e-005	MaxFlux:	3.9528e-005
R-0943	MinFlux:	3.9528e-005	MaxFlux:	3.9528e-005
R-0944	MinFlux:	0	MaxFlux:	0
R-0945	MinFlux:	3.9528e-005	MaxFlux:	3.9528e-005
R-0946	MinFlux:	3.9528e-005	MaxFlux:	3.9528e-005
R-0947	MinFlux:	1.9764e-005	MaxFlux:	1.9764e-005
R-0965	MinFlux:	0.046375	MaxFlux:	0.046375
R-0966	MinFlux:	0	MaxFlux:	0
R-0967	MinFlux:	0	MaxFlux:	0
R-0968	MinFlux:	0	MaxFlux:	0
R-0969	MinFlux:	0	MaxFlux:	0
R-0970	MinFlux:	0	MaxFlux:	0
R-0971	MinFlux:	0	MaxFlux:	0
R-0972	MinFlux:	0	MaxFlux:	0
R-0973	MinFlux:	0	MaxFlux:	0
R-0974	MinFlux:	0	MaxFlux:	0
R-0975	MinFlux:	0	MaxFlux:	0
R-0976	MinFlux:	0	MaxFlux:	0
R-0977	MinFlux:	0	MaxFlux:	0
R-0978	MinFlux:	0.00065297	MaxFlux:	0.00065297
R-0979	MinFlux:	0	MaxFlux:	0
R-0980	MinFlux:	0	MaxFlux:	0
R-0981	MinFlux:	0	MaxFlux:	0
R-0982	MinFlux:	0	MaxFlux:	0
R-0983	MinFlux:	0.013341	MaxFlux:	0.013341
R-0984	MinFlux:	0	MaxFlux:	0
R-0985	MinFlux:	0.013341	MaxFlux:	0.013341
R-0988	MinFlux:	0	MaxFlux:	0
R-0989	MinFlux:	0	MaxFlux:	3.4934
R-0990	MinFlux:	3.9528e-005	MaxFlux:	3.9528e-005
R-0991	MinFlux:	0	MaxFlux:	0
R-0992	MinFlux:	0	MaxFlux:	0
R-0993	MinFlux:	0.00011858	MaxFlux:	0.00011858
R-0994	MinFlux:	1.4731e-005	MaxFlux:	1.4731e-005
R-0995	MinFlux:	0.00011858	MaxFlux:	0.00011858
R-0996	MinFlux:	0.0027534	MaxFlux:	0.0027534
R-0997	MinFlux:	7.9056e-005	MaxFlux:	7.9056e-005
R-0998	MinFlux:	3.9528e-005	MaxFlux:	3.9528e-005
R-0999	MinFlux:	3.9528e-005	MaxFlux:	3.9528e-005
R-1000	MinFlux:	3.9528e-005	MaxFlux:	3.9528e-005
R-1001	MinFlux:	3.9528e-005	MaxFlux:	3.9528e-005
R-1002	MinFlux:	3.9528e-005	MaxFlux:	3.9528e-005
R-1003	MinFlux:	3.9528e-005	MaxFlux:	3.9528e-005
R-1004	MinFlux:	3.9528e-005	MaxFlux:	3.9528e-005
R-1005	MinFlux:	0	MaxFlux:	0
R-1006	MinFlux:	0.00043956	MaxFlux:	0.00043956
R-1007	MinFlux:	0	MaxFlux:	0
R-1008	MinFlux:	0	MaxFlux:	0
R-1009	MinFlux:	0	MaxFlux:	3.3979
R-1010	MinFlux:	0	MaxFlux:	0
R-1011	MinFlux:	0	MaxFlux:	0
R-1012	MinFlux:	0	MaxFlux:	0
R-1013	MinFlux:	0	MaxFlux:	0
R-1014	MinFlux:	0	MaxFlux:	0
R-1015	MinFlux:	0	MaxFlux:	0
R-1016	MinFlux:	0	MaxFlux:	0
R-1017	MinFlux:	0	MaxFlux:	0
R-1018	MinFlux:	0	MaxFlux:	0
R-1019	MinFlux:	0	MaxFlux:	0
R-1020	MinFlux:	0	MaxFlux:	0
R-1021	MinFlux:	0	MaxFlux:	0
R-1022	MinFlux:	0	MaxFlux:	0
R-1023	MinFlux:	0	MaxFlux:	0
R-1024	MinFlux:	0	MaxFlux:	0
R-1025	MinFlux:	0	MaxFlux:	0
R-1026	MinFlux:	0	MaxFlux:	0

R-1027	MinFlux:	0	MaxFlux:	0
R-1028	MinFlux:	0	MaxFlux:	0
R-1029	MinFlux:	0	MaxFlux:	0
R-1030	MinFlux:	0	MaxFlux:	0
R-1031	MinFlux:	0	MaxFlux:	0
R-1032	MinFlux:	0	MaxFlux:	0
R-1033	MinFlux:	0.00065297	MaxFlux:	0.00065297
R-5001	MinFlux:	5.0774	MaxFlux:	5.2913
R-5002	MinFlux:	1.8057	MaxFlux:	5.2913
R-5003	MinFlux:	-8.6441	MaxFlux:	-8.4301
R-5004	MinFlux:	-8.6441	MaxFlux:	-8.4301
R-5005	MinFlux:	9.4509	MaxFlux:	9.7718
R-5006	MinFlux:	9.4509	MaxFlux:	9.7718
R-5007	MinFlux:	0	MaxFlux:	0
R-5008	MinFlux:	0.21866	MaxFlux:	0.21866
R-5009	MinFlux:	0.21866	MaxFlux:	0.21866
R-5010	MinFlux:	1.7308	MaxFlux:	1.7308
R-5011	MinFlux:	1.7308	MaxFlux:	1.7308
R-5012	MinFlux:	2.65	MaxFlux:	2.6719
R-5013	MinFlux:	2.65	MaxFlux:	2.6719
R-5014	MinFlux:	0	MaxFlux:	0
R-5015	MinFlux:	0	MaxFlux:	0
R-5016	MinFlux:	0	MaxFlux:	0
R-5017	MinFlux:	0	MaxFlux:	0
R-5018	MinFlux:	0	MaxFlux:	0
R-5019	MinFlux:	0	MaxFlux:	0
R-5020	MinFlux:	0	MaxFlux:	0
R-5021	MinFlux:	0	MaxFlux:	3.3979
R-5022	MinFlux:	0	MaxFlux:	0
R-5023	MinFlux:	0	MaxFlux:	0
R-5024	MinFlux:	0	MaxFlux:	0
R-5025	MinFlux:	0	MaxFlux:	0
R-5026	MinFlux:	0	MaxFlux:	0
R-5027	MinFlux:	0	MaxFlux:	0
R-5028	MinFlux:	0	MaxFlux:	0
R-5029	MinFlux:	0	MaxFlux:	0
R-5030	MinFlux:	0	MaxFlux:	0
R-5031	MinFlux:	0	MaxFlux:	0
R-5032	MinFlux:	0	MaxFlux:	0
R-5033	MinFlux:	0.014074	MaxFlux:	0.014074
R-5034	MinFlux:	0	MaxFlux:	0.0007326
R-5035	MinFlux:	0.0007326	MaxFlux:	0.0007326
R-5036	MinFlux:	0	MaxFlux:	0
R-5037	MinFlux:	0.013341	MaxFlux:	0.014074
R-5038	MinFlux:	0	MaxFlux:	0
R-5039	MinFlux:	0	MaxFlux:	0
R-5040	MinFlux:	0	MaxFlux:	0
R-5041	MinFlux:	0	MaxFlux:	0
R-5042	MinFlux:	0	MaxFlux:	0
R-5043	MinFlux:	0	MaxFlux:	0
R-5044	MinFlux:	0	MaxFlux:	0
R-5045	MinFlux:	0	MaxFlux:	0
R-5046	MinFlux:	-18.6977	MaxFlux:	-14.9341
R-5047	MinFlux:	-18.6977	MaxFlux:	-14.9341
R-5048	MinFlux:	0	MaxFlux:	0
R-5049	MinFlux:	0	MaxFlux:	0
R-5050	MinFlux:	0	MaxFlux:	0
R-5051	MinFlux:	0	MaxFlux:	0
R-5052	MinFlux:	0	MaxFlux:	0
R-5053	MinFlux:	0	MaxFlux:	0
R-5055	MinFlux:	0	MaxFlux:	0
R-5056	MinFlux:	0	MaxFlux:	0
R-5058	MinFlux:	0	MaxFlux:	0
R-5060	MinFlux:	0	MaxFlux:	0
R-5061	MinFlux:	0	MaxFlux:	0
R-5062	MinFlux:	3.9528e-005	MaxFlux:	3.9528e-005
R-5063	MinFlux:	3.9528e-005	MaxFlux:	3.9528e-005
R-5064	MinFlux:	0.024836	MaxFlux:	0.024836
R-5065	MinFlux:	0	MaxFlux:	0
R-5066	MinFlux:	0	MaxFlux:	0
R-5067	MinFlux:	0.0014452	MaxFlux:	0.0014452
R-5068	MinFlux:	0.0014452	MaxFlux:	0.0014452
R-5069	MinFlux:	0.00063353	MaxFlux:	0.00063353

R-5070	MinFlux:	0.00063353	MaxFlux:	0.00063353
R-5071	MinFlux:	0	MaxFlux:	0
R-5072	MinFlux:	0.033205	MaxFlux:	0.033205
R-5073	MinFlux:	0.001485	MaxFlux:	0.001485
R-5074	MinFlux:	0.001485	MaxFlux:	0.001485
R-5075	MinFlux:	0.000891	MaxFlux:	0.0016236
R-5076	MinFlux:	0.000891	MaxFlux:	0.0016236
R-5077	MinFlux:	0.000594	MaxFlux:	0.000594
R-5078	MinFlux:	0.000594	MaxFlux:	0.000594
R-5079	MinFlux:	0	MaxFlux:	0
R-5080	MinFlux:	0.000594	MaxFlux:	0.000594
R-5081	MinFlux:	0.000594	MaxFlux:	0.000594
R-5082	MinFlux:	0	MaxFlux:	0
R-5083	MinFlux:	0	MaxFlux:	0
R-5084	MinFlux:	0	MaxFlux:	0
R-5085	MinFlux:	0	MaxFlux:	0
R-5086	MinFlux:	0.000594	MaxFlux:	0.000594
R-5087	MinFlux:	0.000594	MaxFlux:	0.000594
R-5088	MinFlux:	0.000891	MaxFlux:	0.000891
R-5089	MinFlux:	0.000891	MaxFlux:	0.000891
R-5090	MinFlux:	0	MaxFlux:	0.0007326
R-5091	MinFlux:	0	MaxFlux:	0
R-5092	MinFlux:	0	MaxFlux:	0
R-5093	MinFlux:	0	MaxFlux:	0
R-5094	MinFlux:	0	MaxFlux:	0
R-5095	MinFlux:	0	MaxFlux:	0
R-5096	MinFlux:	0	MaxFlux:	0
R-5097	MinFlux:	0	MaxFlux:	0
R-5098	MinFlux:	0	MaxFlux:	0
R-5099	MinFlux:	0	MaxFlux:	0
R-5100	MinFlux:	0	MaxFlux:	0
R-5101	MinFlux:	0	MaxFlux:	0
R-5102	MinFlux:	0	MaxFlux:	0
R-5103	MinFlux:	0	MaxFlux:	0
R-5104	MinFlux:	0	MaxFlux:	0
R-5105	MinFlux:	0	MaxFlux:	0
R-5106	MinFlux:	0	MaxFlux:	0
R-5107	MinFlux:	0	MaxFlux:	0
R-5108	MinFlux:	0	MaxFlux:	0
R-5109	MinFlux:	0	MaxFlux:	0
R-5110	MinFlux:	0	MaxFlux:	0
R-5111	MinFlux:	0	MaxFlux:	0
R-5112	MinFlux:	0	MaxFlux:	0
R-5113	MinFlux:	0	MaxFlux:	0
R-5114	MinFlux:	0	MaxFlux:	0
R-5115	MinFlux:	0	MaxFlux:	0
R-5116	MinFlux:	0	MaxFlux:	0
R-5117	MinFlux:	0	MaxFlux:	0
R-5118	MinFlux:	0	MaxFlux:	0
R-5119	MinFlux:	0	MaxFlux:	0
