# Supplementary material for: Co-Consumption of Methanol and Succinate by Methylobacterium extorquens AM1
Source: PLoS One. 2012 Nov 1;7(11):e48271. doi: 10.1371/journal.pone.0048271 (PMC3486813; doi:10.1371/journal.pone.0048271)
Supplement: Table S5 — Details of energetics calculations from flux balance analysis. (PDF) [file pone.0048271.s010.pdf]

ATP production

	Feasible solution		FVA		FBA			
**reaction**	**flux**	**%**	**min**	**max**				
R-0443 (ATP synthase)	27.1	100.0%	27.1232	32.0234				
**SUM**	**27.1**							

ATP production (by origine in respiratory chain)

	Feasible solution		FVA		FBA			
**reaction**	**flux**	**%**	**min**	**max**	**min ATP from meoh**		**max ATP from meoh**	
NDHI (meoh)*	13.2	48.7%			7.2197217	26.6%	16.8	56.5%
cyt c oxidase (meoh)	4.6	17.0%			8.4138634	31.0%	5.1	17.1%
**sum : methanol**	**17.8**	**65.6%**			**15.6**	**57.6%**	**21.9**	**73.6%**
NDHI (succ)*	6.7	24.8%			8.31963	30.6%	5.3	17.8%
succinate dehydrogenase (succ)	2.6	9.5%			3.20	11.8%	2.6	8.7%
**sum : succinate**	**9.3**	**34.4%**			**11.5**	**42.4%**	**7.9**	**26.4%**
**SUM**	**27.1**				**27.1**		**29.7**	

*asignment of NDHI usage to methanol or succinate where calculated from their % in NADH generation

ATP consumption

	Feasible solution		FVA		FBA
**reaction**	**flux**	**%**	**min**	**max**	
precursor biosynthesis	2.0	7.5%			
biomass biosynthesis	5.9	21.8%			
NGAM	9.5	35.0%			
A-GAM	9.7	35.6%			
**SUM**	**27.1**				

NADH production

	Feasible solution		FVA		FBA			
**reaction**	**flux**	**%**	**min**	**max**	**min NADH from meoh**		**max NADH from meoh**	
Mtd A/B (meoh)	2.9	24.1%	0.0	5.3	0.5	6.0%	5.3	38.7%
formate dehydrogenase (meoh)	5.1	42.1%	3.4	5.1	3.5	40.4%	5.1	37.3%
**sum : methanol oxidation**	**8.0**	**66.2%**			**4.0**	**46.5%**	**10.4**	**76.1%**
akg dehydrogenase TCA (succ)	0.3	2.1%	0.0	0.3	0.0	0.0%	0.0	0.3%
pyruvate dehydrogenase (succ)	0.6	5.0%	0.0	1.2	1.1	13.1%	0.3	2.2%
malic enzyme (succ)*	0.0	0.0%	0.0	1.2	0.0	0.0%	0.0	0.0%
malate dehydrogenase (succ)*	0.3	2.1%	0.0	0.3	0.0	0.0%	0.0	0.3%
**sum : succinate oxidation**	**1.1**	9.2%			**1.1**	**13.1%**	**0.4**	**2.9%**
**precursor biosynthesis***	**3.0**	24.6%			3.5	40.4%	2.9	21.0%
**sum : succinate**	**4.1**	**33.8%**			**4.6**	**53.5%**	**3.3**	**23.9%**
**SUM**	**12.1**				**8.6**		**13.7**	

* fluxes through mae and mdh corresponding to the oxidation flux of the TCA cycle, rest of flux through mae and mdh for biosynthesis are taken into account in "prec

NADH consumption

	Feasible solution		FVA		FBA
**reaction**	**flux**	**%**	**min**	**max**	
respiratory chain	11.27	93.61%			
transhydrogenase (succ)	0.00	0.00%	0.00	3.28	
precursor biosynthesis	0.84	7.00%			
biomass biosynthesis	0.00	0.00%			
**SUM**	**12.04**				

NADPH production

	Feasible solution		FVA		FBA			
**reaction**	**flux**	**%**	**min**	**max**	**min NADPH from meoh**		**max NADPH from meoh**	
mtd A (meoh)	2.3	82.7%	0.0	3.3	0.0	0.0%	3.1	93.4%
transhydrogenase (meoh)	0.0	0.0%			0.8	30.3%	0.0	0.0%
**sum : methanol**	**2.3**	**82.7%**			**0.8**	**30.3%**	**3.1**	**93.4%**
isocitrate dehydrogenase (succ)	0.5	17.3%	0.2	0.5	0.3	9.7%	0.2	6.6%
transhydrogenase (succ)	0.0	0.0%			0.4	14.3%	0.0	0.0%
oxidative pentose phosphate pathway (succ)	0.0	0.0%	0.0	3.0	1.3	45.6%	0.0	0.0%
biosynthesis	0.0	0.0%	0.0	0.0	0.0	0.0%	0.0	0.0%
**sum : succinate**	**0.5**	**17.3%**			**1.9**	**69.7%**	**0.2**	**6.6%**
**SUM**	**2.8**				**2.8**		**3.3**	

NADPH consumption

	Feasible solution		FVA		FBA
**reaction**	**flux**	**%**	**min**	**max**	
precursor biosynthesis	0.07	2.40%			
biomass biosynthesis	2.69	97.59%			
**SUM**	**2.8**				

:ursor biosynthesis"
